# Phylogenetic treatment and taxonomic revision of the trapdoor spider genus *Aptostichus* Simon (Araneae, Mygalomorphae, Euctenizidae)

**DOI:** 10.3897/zookeys.252.3588

**Published:** 2012-12-19

**Authors:** Jason E. Bond

**Affiliations:** 1 Department of Biological Sciences, Auburn University Museum of Natural History, Auburn, Alabama, United States of America

**Keywords:** Biodiversity, Biodiversity hotspot, Cladistics, California Floristic Province, Conservation, DNA taxonomy, DNA barcoding, New species, Spider taxonomy, *Aptostichus*, Euctenizidae, Mygalomorphae

## Abstract

This systematic study documents the taxonomy, diversity, and distribution of 40 species of the predominately Californian trapdoor spider genus *Aptostichus* Simon, 1891. Thirty-three of these species are newly described: *Aptostichus dantrippi*, *Aptostichus cabrillo*, *Aptostichus pennjillettei*, *Aptostichus asmodaeus*, *Aptostichus nateevansi*, *Aptostichus chiricahua*, *Aptostichus icenoglei*, *Aptostichus isabella*, *Aptostichus muiri*, *Aptostichus barackobamai*, *Aptostichus sinnombre*, *Aptostichus hedinorum*, *Aptostichus aguacaliente*, *Aptostichus chemehuevi*, *Aptostichus sarlacc*, *Aptostichus derhamgiulianii*, *Aptostichus anzaborrego*, *Aptostichus serrano*, *Aptostichus mikeradtkei*, *Aptostichus edwardabbeyi*, *Aptostichus killerdana*, *Aptostichus cahuilla*, *Aptostichus satleri*, *Aptostichus elisabethae*, *Aptostichus fornax*, *Aptostichus lucerne*, *Aptostichus fisheri*, *Aptostichus bonoi*, *Aptostichus cajalco*, *Aptostichus sierra*, *Aptostichus huntington*, *Aptostichus dorothealangeae*, and *Aptostichus chavezi*. Most of these species are restricted to the California Floristic Province, a known biodiversity hotspot. Of the 40 recognized species, over half are considered to be imperiled or vulnerable and two have likely gone extinct over the past half-century; the conservation status of only 11 species is considered to be secure. Using 73 quantitative and qualitative morphological characters I propose a preliminary phylogeny for the genus that recognizes four major lineages: the *Atomarius*, *Simus*, *Hesperus*, and *Sierra* species groups. Additionally, the phylogenetic analysis indicates that adaptations favoring the invasion of the arid desert habitats of southern California have evolved multiple times across the group. The existence of both desert and non - desert species in three of the four species groups makes this genus an ideal candidate for the study of the evolutionary ecology of desert arthropods. A set of molecular characters based on the contiguous mitochondrial DNA genes 16S-tRNA valine-12S is used in an independent analysis to assist in placement of specimens into species. The taxonomy section explicitly identifies the concept employed in species delimitation. Niche based distribution models are constructed to predict the ranges of species for which an adequate number of sampling sites were known.

## Introduction

The mygalomorph family Euctenizidae is a geographically widespread group of fossorial spiders most of whom capture prey at the entrance of a burrow covered by a silken - soil trapdoor. Raven (1985) originally established this group as a subfamily of the Cyrtaucheniidae, however a number of phylogenetic treatments of the infraorder Mygalomorphae (Bond and Opell 2002, Bond and Hedin 2006, Hedin and Bond 2006, and Bond et al. 2012b) indicated that the family was a classical dumping ground, comprising a number of genera and species that were difficult to place. A recent phylogenetic treatment by Bond et al. (2012b) concluded that Raven’s subfamily Euctenizinae, a group that included all of the North American cyrtaucheniids, was a monophyletic taxon that is closely related to idiopids and should be elevated to the family rank. This group comprises the eastern North American genus *Myrmekiaphila* Atkinson, 1886 (recently revised by Bond and Platnick 2007, also see Bailey et al. 2010 and Bond et al. 2012a), and the southwestern genera *Neoapachella*, Bond & Opell 2002, *Eucteniza* Ausserer, 1875, *Promyrmekiaphila* Schenkel, 1950 (recently revised by Stockman and Bond 2008, but also see Stockman and Bond 2007), *Entychides* Simon, 1888, *Apomastus* Bond & Opell, 2002, and *Aptostichus* Simon, 1891. Although the basal euctenizid lineages are probably relatively old (Bond and Hedin 2006) most of the genera are depauperate with respect to morphological and species diversity. Many consist of very few species and two, *Neopachella* and *Promyrmekiaphila*, are either monotypic (Bond and Opell 2002) or comprise only two morphological species (Stockman and Bond 2008). That said, morphological assessments likely underestimate the evolutionary diversity contained within these groups (Bailey et al. 2010, Bond et al. 2001, Bond and Stockman 2008, Starrett and Hedin 2006, Stockman and Bond 2007).

**Figures 1–6.**
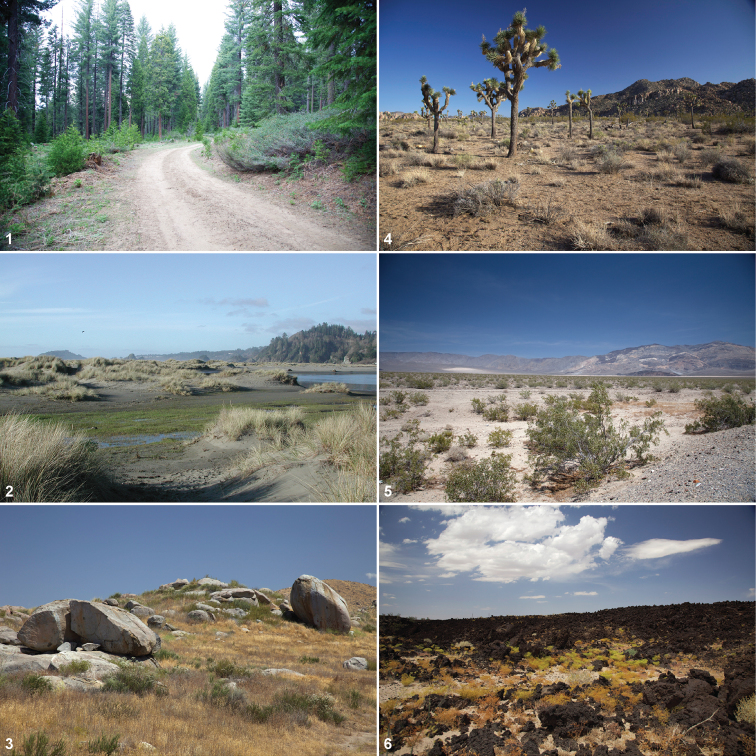
Breadth of diversity of *Aptostichus* species habitat types in the California Floristic Province. **1** alpine habitat, Sierra Nevada Mountain Range, Fresno County **2** northern coastal dunes, Humboldt County **3** chaparral, Riverside County **4** Joshua Tree National Park, Riverside County **5** Panamint Valley, Inyo County **6** Pisgah Crater, San Bernardino County.

In terms of diversity the trapdoor spider genus *Aptostichus*, the subject of this revision, is an anomaly with respect to high species diversity relative to the other euctenizid genera and many other mygalomorph groups. Itcomprises > 40 species restricted primarily to the state of California, with additional, species, in the states of Nevada and Arizona (one and two respectively). Among southwestern mygalomorph genera (save theraphosids, tarantulas), this diversity is rivaled only by the antrodiaetid genus *Aliatypus* Smith, 1908 that contains a third as many species. *Aptostichus* species range widely in size (carapace length 3 - 7.5 mm), coloration, and habitat type. These features, and others (described below) make the genus very interesting from an evolutionary and ecological perspective. Although relatively restricted geographically its species are found in disparate habitats (Figs 1–6), ranging from Mediterranean climates to the arid Mojave and Colorado deserts. Their apparent ecological specialization coupled with high species diversity makes these spiders ideal for investigations of the evolution of characters associated with desert adaptations. The “trapdoor spider desert adaptation paradigm” has been addressed by others (Main 1978, Coyle and Icenogle 1994) but never in an explicit phylogenetic context. Additionally, the distribution of this genus across the unique taxonomically and geologically diverse Californian Floristic Province (Myers et al. 2000), a region recognized as a biodiversity hotspot, provides an important and well-studied system in which to consider questions about the geography of speciation and adaptation and makes this group of high conservation interest.

Although *Aptostichus* may be noteworthy from an evolutionary and conservation perspective, its taxonomy has been largely neglected. Since the original description of the genus by Simon (1891) only three species of *Aptostichus* were subsequently described during the 20^th^ Century (Smith 1908; Chamberlin 1917, 1919). Largely through the efforts of Mr. Wendell Icenogle and Dr. Willis Gertsch during the late 1960’s through the 1970’s, many *Aptostichus* specimens were collected and the high diversity in this group began to come to light. It is apparent from letters and preliminary taxonomic worksheets created during the 1970’s that Gertsch had intended to revise the genus, a project that never reached fruition. More recently, molecular studies focusing on a speciation pattern and process within the *Aptostichus atomarius* species complex, a widespread, morphologically homogenous species distributed broadly throughout southern and western California (Bond and Stockman 2008), have resulted in the description of an additional three species (*Aptostichus stephencolberti*, *Aptostichus miwok*, and *Aptostichus angelinajolieae*). These results were consistent with earlier molecular studies of the coastal dune endemic species *Aptostichus simus* (Bond et al. 2001) that likewise seemed to indicate species crypsis.

**Figures 7–10.**
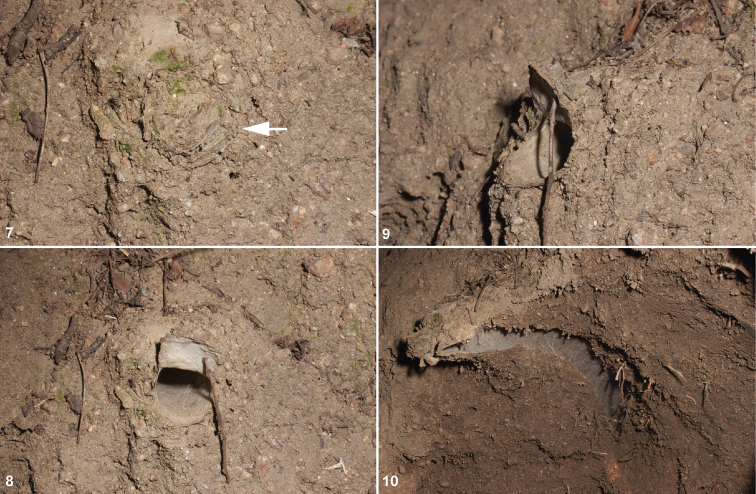
*Aptostichus icenoglei* sp. n. burrow; specimen MY2465, adult female, excavated from roadside bank north of Fallbrook on DeLuz Road (San Diego County). **7** burrow with trapdoor closed **8, 9** trapdoor propped open frontal and side views **10** burrow excavated.

The covert behavior and simple morphology of many mygalomorph groups (Coyle 1971), particularly when compared to many other more “advanced” araneomorph spider groups, is probably why *Aptostichus* has been overlooked by other spider workers. Like many mygalomorph groups, itis perhaps even more difficult to study because females of many species lack obvious distinguishing morphological features altogether (humorously characterized as “hopeless” by Gertsch *in lit*.). Additionally, many species can be collected only during certain times of the year and collecting typically requires that the burrows be excavated, an activity that is often very time-consuming. Because *Aptostichus* species construct flimsy, thin wafer trapdoors (Figs 7–10) that often have plant debri attached rendering them cryptic, these doors cannot easily be detected by simply searching a substrate for a thin door outline. Therefore, one must sometimes use a “scraping” technique to find burrows by removing the first few centimeters of topsoil, thereby exposing the silk lined burrow. This technique however, is not very effective in sandy desert habitats. The only way to find desert *Aptostichus* females appears to be after winter rains when the spiders extend, or clean out their burrows, leaving a small mound of sand at the burrow entrance. In contrast, males are much easier to distinguish than females and have been widely collected in standard pitfall traps. This sex-specific disparity is reflected herein in that some female specimens were impossible to accurately assign to a species on the basis of morphology alone; that is, molecular data were sometimes necessary to assign specimens to a taxon.

The objectives of this systematic study are two-fold. First, I aim to provide a first taxonomic treatment of the genus *Aptostichus*. This taxonomic study seeks to answer basic questions about species delineation and distribution, thereby providing the information necessary for future studies of speciation pattern and process, character evolution, adaptation, and biogeography in this incredibly diverse and interesting group of trapdoor spiders. Second, I propose an interspecific phylogeny for *Aptostichus*; however, caution that this phylogeny should be considered as preliminary. Although over 70 morphological characters are used, many of these are thought to be homoplasic a priori(e.g., features like carapace and abdomen coloration are obvious psammophilic characteristics). And, many of the characters are single sex genitalic features; both sexes are unknown for about one-third of the species. This introduces a suite of characters for which the states cannot be assessed for a large proportion of species, undoubtedly affecting the results of phylogenetic analysis presented herein. All caveats aside, this phylogeny and taxonomic revision describes 33 new species of *Aptostichus*, establishes four monophyletic species groups and provides the phylogenetic framework needed to guide future studies of this group’s taxonomy and evolution.

## Abbreviations, materials and methods

The following institutional and quantitative morphological abbreviations used in this paper are defined as follows:

### Institutional

**AMNH** (American Museum of Natural History; New York, New York), **AUMNH** (Auburn University Museum of Natural History; Auburn, Alabama), **CAS** (California Academy of Sciences; San Francisco, California), **DUB** (personal collection of Darrell Ubick; San Francisco, California), **ICE** (personal collection of Wendell Icenogle; Winchester, California), **MCZ** (Museum of Comparative Zoology, Harvard; Boston, Massachusetts), **MEL** (personal collection of Mel Thompson), **MNHN** (Muséum National D’Histoire Naturelle, Paris), **SCW** (Personal collection of Scott C. Williams, deposited in the AMNH), **UCR** (University of California Riverside; Riverside, California).

### Quantitative morphological

**ANTd**: number of teeth on the anterior margin of the female cheliceral fang furrow.

**Figures 11–16.**
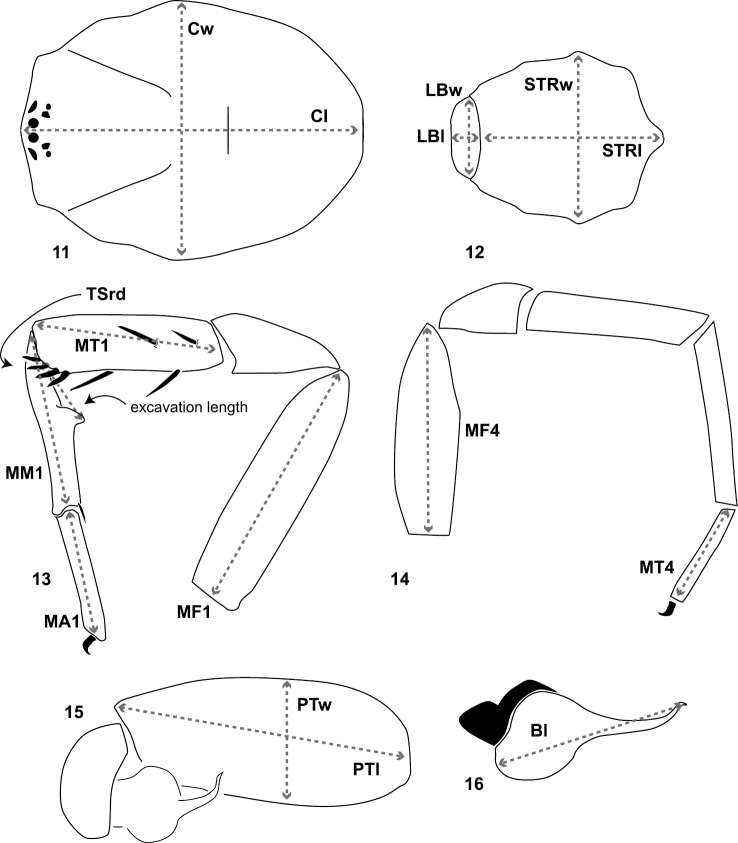
Diagrammatic representation of quantitative measurements of morphological features and leg articles. **11, 12** carapace, labium, sternum length and width **13** lengths of leg I femur, tibia, metatarsus, tarsus, metatarsal proximal/ventral excavated region, number of tibia distal retrolateral spines **14 **lengths of leg IV femur and tarsus **15** male palpal tibia length and width **16** male copulatory bulb length.

**Cl, Cw**: carapace length and width (Fig. 11). Carapace length taken along the midline dorsal most posterior position to the anterior front edge of the carapace (chelicerae are not included in length). Carapace width taken at the widest point.

**LBl**, **LBw**: labium length and width taken from the longest and widest points, respectively (Fig. 12).

**MF1**, **MT1**, **MM1, MA1**: lengths of male leg I femur, tibia, metatarsus, and tarsus (Fig. 13).

**MF4**, **MT4**: length of male leg IV femur and tarsus (Fig. 14) taken from the prolateral aspect.

**PTl, PTw**: male palpal tibia length and width (Fig. 15).

**Bl**: palpal bulb length from embolus tip to the bulb base, taken in the ventral plane at its longest point (Fig. 16).

**PTLs, TBs**: number of female prolateral patella and tibial spines leg III.

**STRl, STRw**: sternum length and width. Sternum length from the base of the labium to its most posterior point. Width taken across the widest point, usually between legs II and III (Fig. 12).

**TSrd**, **TSp**, **TSr**: number of tibia I spines on the distal most retrolateral, prolateral, and midline retrolateral positions.

### Measurement, characterization, and illustration of morphological features

Unique voucher numbers were assigned to all specimens (alphanumeric designations beginning with AP or MY); these data were added to each vial and can be used to cross-reference all images, measurement, and locality data. All measurements are given in millimeters and were made with a Wild M8 or Leica M9.5Z dissecting microscope equipped with an ocular micrometer scale. Appendage measurements, quantitative and meristic, were based on left appendages in the retrolateral (unless otherwise stated) view using the highest magnification possible. Measurements of large structures (e.g., leg article lengths, carapace and sternum dimensions, etc.) are accurate to 0.03–0.015 mm. Measurements of smaller structures (e.g., palpal bulb and labial dimensions) are accurate to 0.0075 mm. Lengths of leg articles were taken from the mid–proximal point of articulation to the mid–distal point of the article (*sensu* Coyle 1995 Figure 1; Figs 11–16). Leg I article measurements are listed in the species descriptions in the following order: femur, tibia, metatarsus, tarsus and metatarsus ventral excavation (Fig. 13); leg IV measurements are femur and tarsus only (given in that order). Carapace and leg coloration are described semi-quantitatively using Munsell® Color Charts (Windsor, NY) and are given using the color name and color notation (hue value/chroma).

**Figures 17–20.**
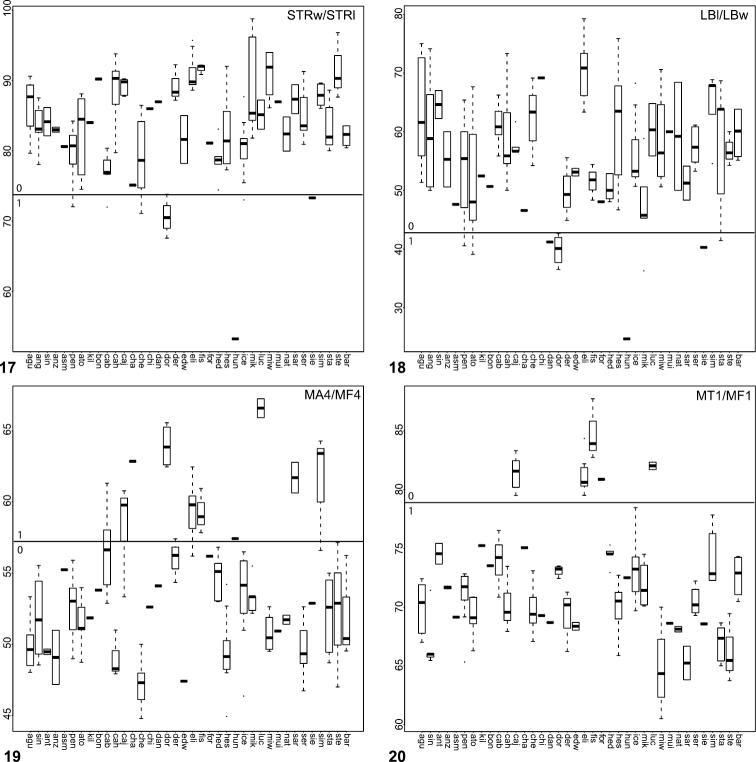
Box plots for quantitative measurements used in phylogenetic analysis and species diagnosis; ratios are given in values multiplied by 100 (y-axis); characters states are indicated above and below horizontal line bisecting graph; species abbreviations (x-axis) defined in Table 1. **17** sternum length to width **18** labium length to width **19** leg IV tarsus length to femur length **20** leg I tibia length to femur length.

**Figures 21–24.**
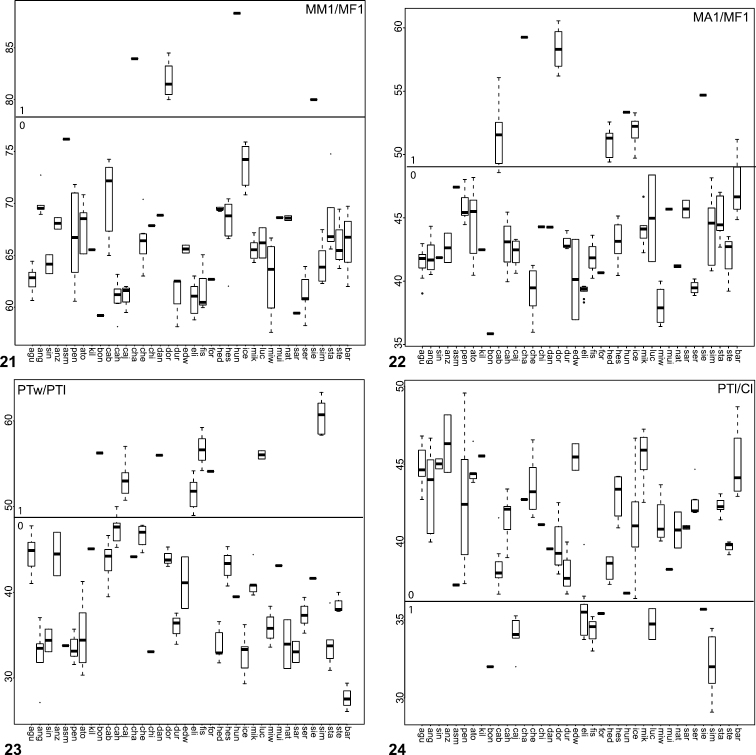
Box plots for quantitative measurements used in phylogenetic analysis and species diagnosis; ratios are given in values multiplied by 100 (y-axis); characters states are indicated above and below horizontal line bisecting graph; species abbreviations (x-axis) defined in Table 1. **21** leg I metatarsus length to femur length **22** leg I tarsus length to femur length **23** male palpal tibia width to length **24  **palpal tibia length to carapace length.

**Figures 25–27.**
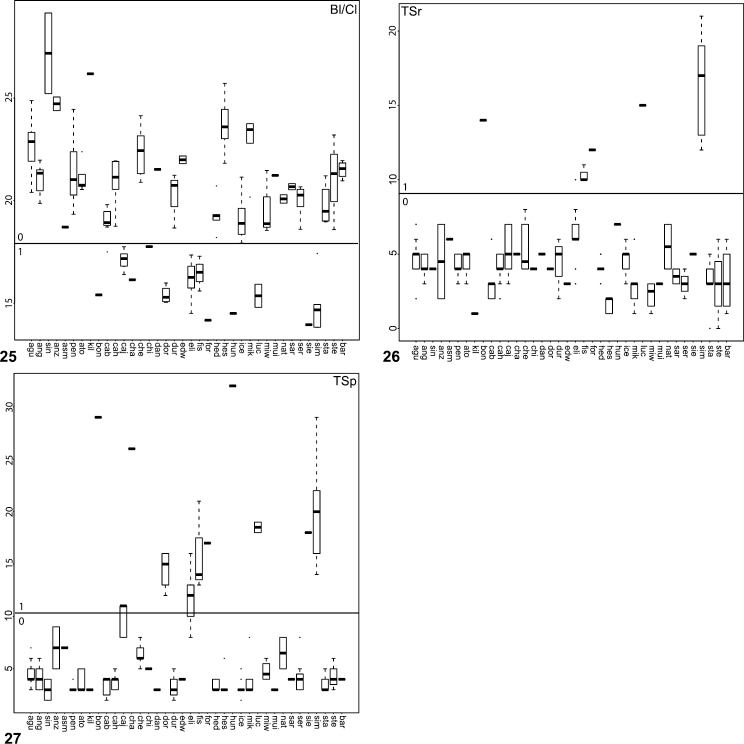
Box plot and counts for quantitative and meristic measurements used in phylogenetic analysis and species diagnosis; ratio given in values multiplied by 100 (y-axis); characters states are indicated above and below horizontal line bisecting graph; species abbreviations (x-axis) defined in Table 1. **25** male copulatory bulb length to carapace length **26** number of male, leg I tibia retrolateral spines **27 **number of male, leg I tibia prolateral spines.

Quantitative measurements are based on a minimum of five individuals of each sex when a sufficient number of specimens were available. When more than five specimens were available individuals were sampled from across the species’ geographical and size distribution (i.e., every attempt was made to select specimens that represented the range of sizes available in the collection). Species descriptions and material examined sections were generated using two simple Python scripts written specifically for generating homogenous species accounts [SPEEDM and MATex (Brewer and Bond (2012) available for download from the Dryad Data Repository at doi: 10.5061/dryad.3b95n)]. Characters for the phylogenetic analysis were scored from the type specimens, with the exception of the quantitative characters, which were scored on the basis of multiple specimens. Outgroup taxa were scored using the euctenizid specimens listed in Bond and Opell (2002) and in Bond and Hedin (2006) as exemplars. Quantitative values were taken from each of these exemplar taxa.

Mating clasper and palpal line drawings were made for some specimens (when needed to further clarify spination patterns) with the aid of a dissecting scope equipped with a camera lucida. Line drawings were scanned as digital images and converted to vector drawing objects in Adobe Illustrator (Adobe Systems Inc.). Digital images of specimens were made using a Visionary Digital Imaging System (Visionary Digital^TM^, Richmond, VA) where images were recorded at multiple focal planes and then assembled into a single focused image using the computer program Helicon Focus (Helicon Soft, Ltd., Ukraine). The female genital region was removed from the abdominal wall and tissues dissolved using trypsin; spermathecae were examined and photographed in the manner described above. Habitus illustrations were constructed from whole body images that were bisected, copied, and reflected in Adobe Photoshop (Adobe Systems, Inc.) to produce a roughly symmetrical image; the actual raw image on which the habitus illustration is based has been deposited in Morphbank and its record number noted in the figure legend (value in square [ ] brackets). For scanning electron microscopy, specimens were air-dried and sputter-coated with gold prior to viewing on an FEI Quanta 200 scanning electron microscope.

### Evaluation of quantitative morphological characters for diagnosis and phylogenetic analyses

Quantitative morphological features that were determined to have discrete, non-overlapping ranges for individual subsets of species were scored as phylogenetic characters and were used as features for morphological diagnosis of species. The criterion that employs only non-overlapping features limits the number of quantitative features and character states available to our analysis; these non-overlapping characters were chosen from a much larger suite of morphological measurements, many of which lacked discrete non-overlapping ranges (but may have differed statistically). Coyle (e.g., 1994, 1995) has frequently used an analysis of variance method to delineate states of quantitative characters for mygalomorph phylogenetic analyses. Mean values that statistically differ are scored as discrete states, regardless of potentially overlapping ranges. This approach is not uncommon and a number of other authors have likewise proposed methods for scoring overlapping quantitative characters (e.g., Chappill 1989, Goldman 1988, Thiele 1993, Wiens 2001, Goloboff et al. 2006). The use of these quantitative data has received some “conceptual” scrutiny, both negative (Farris 1990) and positive (e.g., Goloboff et al. 2006; see Garcia-Cruz and Sosa 2006 for detailed assessment of various approaches). However, the utility of overlapping quantitative character states has not been adequately tested (but see Hendrixson and Bond 2009). For this reason and others, I am hesitant to use these data as such in this present study. However, acknowledge that the approach I have employed here is not without certain problems. For example, in the case of species represented by only a few specimens, additional collecting could add specimens whose features expand the range of some characters and possibly negate, or change the scoring of said characters.

### Morphological characters scored

**General morphological and spinning features**

1. Thorax: flat = 0; sloping = 1 (see Bond and Beamer 2006; figs 1A, 1C).

2. Carapace pubescence: absent = 0; light = 1; heavy = 2 (Fig. 96).

3. Posterior edge of male carapace: aspinose = 0; with a distinct fringe of heavy spines and/or setae = 1 (Fig. 53).

4. Posterior thorax sclerotization: normal = 0; light = 1. See Bond and Opell (2002) for a detailed explanation of this character and its states.

5. AME and PME: subequal in diameter = 0; AME diameter greater = 1.

6. Eye tubercle: absent = 0; present, low = 1; present, high = 2.

7. Male thoracic groove: transverse = 0; recurved = 1; procurved = 2.

8. Sternal shape (Fig. 17): normal (STRw/STRl > 74.0) = 0; rounded and raised in the ventral plane = 1; long (STRw/STRl < 73.8) = 2.

9. Rastellum: on a distinct process = 0; consisting of large spines not on a process = 1.

10. Rastellar spines: normal = 0; enlarged = 1.

11. Rastellum retrolaterally offset spine (Fig. 189): absent = 0; present = 1.

12. Endite cuspules: restricted to medial posterior aspect = 0 (Fig. 256); widespread = 1.

13. Male labial cuspules: absent = 0; present = 1.

14. Male palpal endite cuspules: absent = 0; present = 1.

15. Labium shape (Fig. 18): wider than long/subquadrate (LBw/LBl > 42.5) = 0; very wide (LBw/LBl < 42.5).

16. Sternal sigilla: large = 0 (Fig. 188); small = 1 (Fig. 256).

17. Sternal sigilla: widely spaced = 0; closely spaced or contiguous = 1 (Fig. 188).

18. Carapace coloration: light = 0 (Figs 88–90); dark = 1 (Figs 79–81).

19. Abdominal color pattern: solid or with solid striping = 0; mottled striping = 1 (Fig. 80).

20. Abdomen coloration: light = 0 (Fig. 105); dark = 1 (Fig. 95).

21. Cheliceral dentition: single promarginal row of large teeth with retromarginal row of small denticles = 0; both margins with larger teeth = 1.

22. Pumpkiniform spigots (see Bond and Opell 2002): absent = 0; present = 1.

23. Spigot bases: with invaginations = 0; without invaginations = 1.

**Male leg and microstructural characters**

24. Tarsus IV length (Fig. 19): short (MA4/MF4 < 60.0) = 0; long (MA4/MF4 > 60) = 1.

25. Tarsus I pseudosegmentation (Fig. 279): absent = 0; present = 1.

26. Tarsus I: straight = 0; curved = 1 (Fig. 308).

27. Tarsus IV pseudosegmentation: absent = 0; present = 1.

28. Tarsus IV: straight = 0; curved = 1.

29. Tarsus I: stout (diameter equal to or greater than metatarsus) = 0; slender (diameter less than metatarsus) = 1.

30. Tarsus ventral spines: absent = 0; present = 1 (Fig. 311).

31. Leg I coloration: uniform = 0; distal 1/2 metatarsus and tarsus light in color = 1.

32. Tibia I length (Fig. 20): short (MTI/MFI < 79.53) = 0; long (MTI/MFI > 79.53).

33. Metatarsus I length (Fig. 21): short (MMI/MFI < 77.5) = 0; long (MMI/MFI > 77.5) = 1.

34. Tarsus I length (Fig. 22): short (MAI/MFI < 49.72) = 0; long (MAI/MFI > 49.72) = 1.

35. Tarsal scopulae: thin = 0; thick = 1.

36. Tarsal scopulae on leg IV: present = 0; absent = 1.

37. Bifid STCI basal tooth: absent = 0; present = 1.

**Female leg and microstructural characters**

38. Female tarsal scopulae: light = 0; dense = 1.

39. Metatarsus IV preening comb: absent = 0; present = 1 (Figs 45, 62).

40. Metatarsus III preening comb: absent = 0; present = 1.

**Secondary sexual and genitalic characters**

41. Palpal tibia (Fig. 23): stout (PTw/PTl > 50) = 0; slender (PTw/PTl < 50) = 1.

42. Palpal tibia (Fig. 24): short (PTl/Cl < 36) = 0; long (PTl/Cl > 36) = 1.

43. Palpal tibia spines: long and ventrally positioned = 0 (Fig. 71); short and retrolaterally positioned = 1 (Fig. 278).

44. Palpal tibia megaspines: present = 0 (Figs 341, 349); absent = 1.

45. Male tibia I ventral megaspine: absent = 0; present = 1.

46. Male metatarsus I mating apophysis: absent/non - distinct = 0 (Fig. 333); rectangular = 1 (Figs 149, 160); triangular = 2 (Fig. 192); triangular and hooked = 3 (Fig. 69).

47. Male metatarsus I mating apophysis spine: absent = 0; present = 1 (Fig. 160).

48. Male metatarsus I: straight = 0; anteverted when viewed in retrolateral aspect = 1 (Fig. 266).

49. Male metatarsus I proximal excavation: absent = 0 (Fig. 333); present = 1 (Fig. 107).

50. Embolus serration: absent = 0 (Figs 68, 72); present = 1 (Figs 49, 50, 277).

51. Embolus shape: single bend = 0 (Fig. 126); sigmoidal = 1 (Fig. 72).

52. Embolus: thin = 0 (Fig. 72); stout = 1 (Fig. 277).

53. Embolus shape: cylindrical = 0 (Fig. 72); dorsal - ventrally compressed = 1 (Fig. 277).

54. Sperm duct directly below bulb embolus junction: straight = 0; looped = 1.

55. Tip of embolus: normal, gradual taper = 0; tapers sharply into a very thin terminus = 1 (Fig. 335).

56. Male pedipalp distal prolateral tibial spine: absent = 0; present = 1 (Fig. 271).

57. Palpal bulb (Fig. 25): short (Bl/Cl < 17) = 0; long (Bl/Cl > 17) = 1.

58. Prolateral cymbial lobe: normal = 0; extended = 1.

60. Retrolateral, distal most aspect of cymbium forms a distinct process: no (normal) = 0; yes = 1 (Fig. 278).

61. Retrolateral cymbium spine row: absent = 0; present = 1 (Figs 246, 248).

62. Retrolateral distal tibial spines: absent = 0 (Fig. 165); present = 1 (Fig. 55).

63. Retrolateral distal tibial spines: absent = 0; short = 1 (Fig. 194); long = 2 (Fig. 123).

64. Retrolateral distal spines: absent = 0; arranged distally = 1 (Figs 73–75); offset behind distal margin = 2 (Fig. 167).

65. Retrolateral distal tibial spines: uniform, non-overlapping = 0 (Figs 73–75); uniform = 1 (Fig. 160); absent = 2.

66. Tibia I, 1 - 1 - 1 spination pattern: absent = 0; present = 1 (Fig. 70).

67. TSr (Fig. 26): few (TSr < 10) = 0; many (TSr > 10) = 1.

68. TSp (Fig. 27): few (TSp < 7) = 0; many (TSp > 7) = 1.

69. Spines on prolateral surface of male patella I: few (<7); many large spines (>7; Figs 139, 144).

70. Spermathecal lateral base: absent = 0; present = 1 (Figs 76–78).

71. Secondary spermathecal bulb: absent = 0; present = 1.

72. Median spermathecal stalk: short, approximately as long as wide = 0 (Fig. 76); long, much longer than wide = 1 (Fig. 202).

73. Median spermathecal bulb: large (exceeds diameter of median stalk) = 0 (Fig. 76); small (diameter of bulb and median stalk subequal) = 1 (Fig. 202).

74. Median spermathecal stalk: straight = 0 (Fig. 76); sinuous = 1 (Fig. 203).

### Morphological and molecular character matrix construction and phylogenetic analyses

Phylogenetic analyses of *Aptostichus* relationships were conducted using molecular and morphological data sets employing parsimony, Bayesian, and likelihood optimality criteria. Molecular data sets analyzed include data drawn from previous smaller studies (Bond et al. 2001, Bond and Stockman 2008, Bailey et al. 2010) and from newly collected data. Handling of tissues, DNA preparation, and sequencing followed procedures outlined in Bond and Stockman (2008). Legs were removed from specimens and preserved in RNAlater (Qiagen, Valencia, CA) and stored at −80C; whole specimens were preserved for morphological studies in 80% ethanol. Genomic DNA was extracted using the Qiagen DNeasy Tissue Kit. Standard PCR protocols were used to amplify an approximately 1500–base pair region of the mitochondrial genome spanning the region coding for the 12S rRNA, val-tRNA, and 16S rRNA genes. Amplification products were produced using the primers LR-J-12887 CCGCTCTGAACTCAGATCACGT and SR-N-14612spid AAGACAAGGATTAGATACCCT. After agarose gel verification and purification using ExoSAP-IT (USB, Cleveland, Ohio), products were sequenced on an ABI 3130 automated sequencer (Applied Biosystems, Foster City, CA) directly using the PCR primers and an additional internal sequencing primer (LR-J-13XXXa GGCAAATGATTATGCTACC). Sequence data were edited using the computer program Sequencher (Genecodes, Madison, WI) and then aligned using the software package Muscle (Edgar 2004) with default opening and gap extension penalties; minor adjustments to the alignment were made manually using the computer program Mesquite (Maddison and Maddison 2010). Molecular analyses primarily focused on identification of specimens, particularly juveniles, to species by phylogenetic association; that is, evidence of common ancestry with specimens identified by other means (e.g., morphological or cohesion species criteria). Given the paucity of species for which molecular data were available (less than half), it would be unwise to infer intra-generic relationships among species from these analyses.

Phylogenetic analyses of the molecular data comprised Bayesian and likelihood analyses. For Bayesian analyses the appropriate model of DNA substitution for each of the mtDNA data partitions was chosen using the computer program Kakusan 3 (Tanabe 2007) by Bayesian Information Criterion (BIC). MrBayes ver. 3.1.2 (Ronquist and Huelsenbeck 2003, Huelsenbeck and Ronquist 2001) was used to implement a Bayesian inference of phylogeny using the substitutions models indicated by BIC. Analyses comprised four concurrent Markov Chain Monte Carlo (MCMC) chains run for 40,000,000 generations with trees saved every 1,000 generations. The two independent runs were considered to have converged when the standard deviation of split frequencies value was < 0.01. Topologies were discarded as burn-in following visual inspection in the program Tracer (Rambaut and Drummond 2007); clade posterior probabilities were computed from the remaining trees. The reported likelihood score and post burn-in tree topology was computed using the sump and sumtcommand with the option contype=allcompat, respectively. Likelihood analyses were conducted using the computer program RAxML ver. 7.2.8 (Stamatakis 2006). Parameters employed in the analysis included 1000 random addition sequence replicates with the general time reversible model and gamma model of site heterogeneity (-m = GTRGAMMA, -# = 1000). Branch support values were computed via 1000 non-parametric bootstrap replicates. Bootstrap bipartitions were drawn onto the best tree topology obtained in the previous analysis.

Phylogenetic analyses of the morphological data set were performed using PAUP* version 4.0b10 (Swofford 2002). All binary characters were treated as reversible, multistate characters and were treated as unordered, and all characters were initially weighted equally. Heuristic searches were performed using random stepwise addition (1000 replicates) of taxa followed by TBR (tree bisection-reconnection) branch swapping held to one million rearrangements per replicate. Branches with a maximum length of zero were collapsed. The preferred tree topology (presented herein) is based on the search conducted in PAUP* using the “Goloboff Fit Criterion” (Goloboff 1993a, b, c, 1995) with the search parameters described above. Solutions using an array of concavity function constants (k= 3-12) were explored.

For the purposes of evaluating the evolution of habitat type, species were scored for seven habitat type character states: (0) mixed forest and coastal range; (1) chaparral; (2) alpine meadow; (3) desert; (4) coastal dune; (5) mixed redwood; (6) dry steppe. Character scorings were based on personal observations and ecoregion types assessed in the computer program ArcGIS (ESRI, Redlands, CA) using the 2007 EcoRegionsCalifornia07_3 GIS data set (Cleland et al. 2007) downloaded from http://www.fs.fed.us/r5/rsl/projects/gis/data/calcovs/EcoregionsCalifornia07_3.html . Taxa found in multiple habitat types were scored as polymorphic. Character state reconstructions using parsimony were evaluated on the preferred tree topology (Fig. 29) using the computer program MacClade ver. 4.05 (Maddison and Maddison 2000).

**Figure 28.**
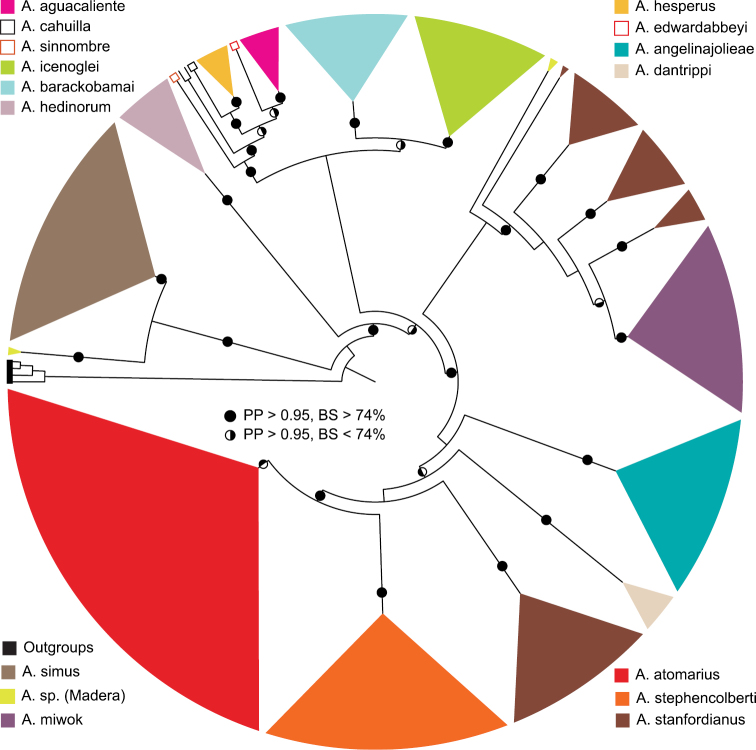
Summary tree based on an analysis of 337 individuals scored for the 12S-tRNA valine-16S mtDNA gene region comprising 1618 base pairs. Solid dots on internal branches denote strong posterior probability (PP) and bootstrap support (BS); half-shaded dots are nodes with bootstrap values < 74%.

**Figure 29.**
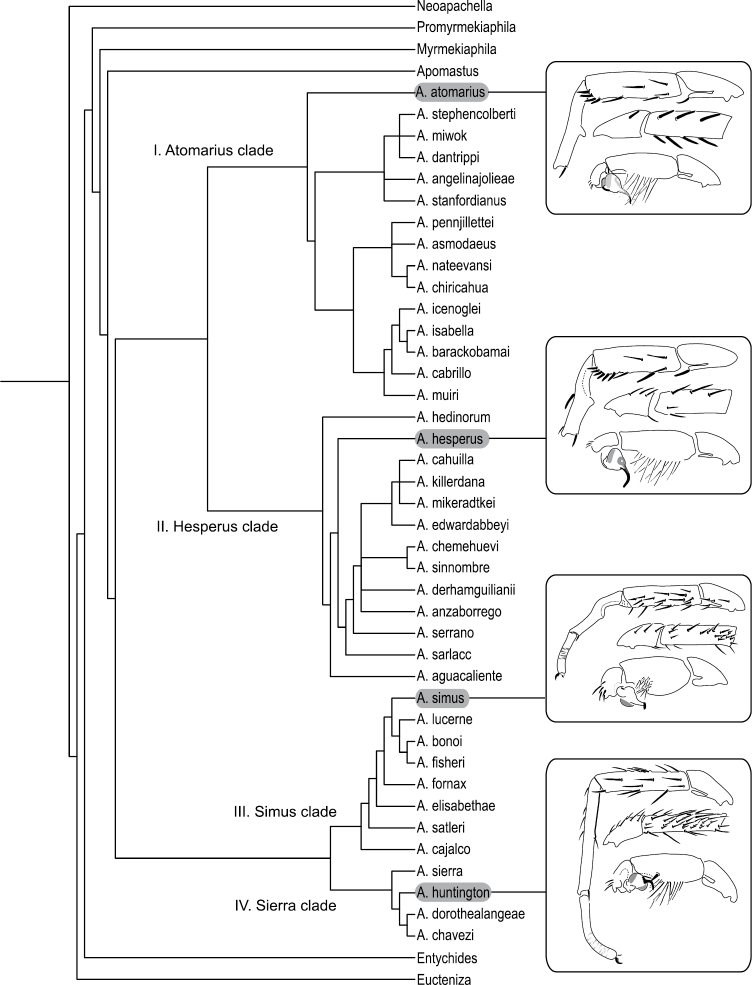
Preferred tree topology based on the analysis of 72 morphological characters employing implied weights (k = 7). The inferred tree recovers four monophyletic species groups: *Atomarius*, *Hesperus*, *Simus*, and *Sierra* clades. Inset figures illustrate exemplar mating clasper and pedipalp morphologies for each of the four major clades.

### Locality data, georeferencing, generation of niche-based distribution models, and conservation status

Latitude and longitude for all collecting localities were recorded in the field using a Garmin® Global Positioning System receiver (Garmin International Ltd., Olathe, KS) using WGS84 map datum. For previously collected specimens (e.g., loaned museum specimens) locality data were georeferenced by hand by finding the approximate locality on United States Geological Survey topographic maps (NAD83 map datum) or Google Earth (WGS84 datum). All georeferenced and field recorded locality data (latitude, longitude, elevation) were crosschecked by hand in Google Earth prior to generating distribution map illustrations and database entry. Distribution maps were constructed using ArcGIS using NAD83 map datum. Because many older collecting labels lack sufficient locality information, many georeferenced values are imprecise and should be used with caution. Data for labels that document only county and/or town information were georeferenced to the approximate geographic center of the locality given. Precision for each georeferenced point is annotated as a superscript in each material examined section of the species’ taxonomy using the confidence value scheme employed by Murphey et al. (2004): 1 = exact coordinates given; 2 = amended exact coordinates (i.e., exact coordinates given but were emended on validation); 3 = public land survey system (or herein geographic place name); 4 = within 1km radius; 5 = within 5km radius; 6 = within 10km radius; 7 = to county or > 10km; 8 = to state; 9 = to project region. Latitude and longitude are recorded to the 4^th^ decimal place as an indication of the precision in the point assigned by us (i.e., where I have assigned the locality place-holder for the specimen in question), not precision in the recording of the value or to specify the exact point of collection. Actual precision of the record is to be inferred from the numbering system described above. Detailed locality and associated GIS data as supplemental data files in spreadsheet and KML file format can be downloaded online from the Dryad Data Repository at doi: 10.5061/dryad.3b95n.

As an approach to facilitating species discovery and determination, niche-based distribution models (DM’s) were constructed for species for which sufficient locality data were available (> 5 points). Niche-based DM’s provide estimates for the probability of finding a species at a location on the landscape given the set of correlate ecological and climatic parameters used to construct the model. Locality coordinates for each specimen were imported into ArcMap (ESRI, Redlands, CA) and converted into shape files. Following the procedure outlined in Bond and Stockman (2008), DM’s were constructed using environmental layers thought to “likely influence the suitability of the environment” (Phillips et al. 2006) based on our previous analyses of *Aptostichus atomarius* species complex distributions (see Stockman and Bond 2007, for further justification of layer choice). Seven climatic layers were obtained from the WORLDCLIM data set (Hijmans et al. 2005): annual precipitation, precipitation seasonality, annual maximum temperature, annual minimum temperature, temperature seasonality, and mean precipitation during the driest and wettest quarters. A seventh layer, elevation, was constructed from a mosaic of Digital Elevation Models (DEMs) derived from the National Elevation Dataset (USGS). DEMs were converted to Raster format in ArcMap and resampled from 30-m resolution to 1-km resolution using bilinear interpolation. All seven layers were clipped to the same extent, cell size, and projection. Niche-based (DMs) were created using the computer program Maxent (Phillips et al. 2006). Maxent employs a maximum likelihood method that estimates a species’ distribution that has maximum entropy subject to the constraint that the environmental variables for the predicted distribution must match the empirical average (Elith et al. 2006; Phillips et al. 2006). Parameters for all Maxent analyses used the default values: convergence threshold = 10−5, maximum iterations = 500, regularization multiplier = 1, and auto features selected. Additional larger values of the regularization multiplier were used to ensure that models were not overfitting the data.

A hypothesized conservation status of all species has been included with each description. I have used the NatureServe ranking scheme–secure, apparently secure, vulnerable, imperiled, critically imperiled, and extinct–to describe perceived status. The designations provided are not based on any formal calculations (see Faber-Langendoen et al. 2009 for explicit criteria) and thus should not be viewed as formal status declarations. Many *Aptostichus* species are rare in collections and very difficult to collect, consequently parameters like rarity and abundance are impossible to accurately assess at this time. As such, I have based conservation status designations on extent of distribution and apparent threats to habitat (e.g., is the species known only from a highly impacted area?); a brief rationale is provided with each determination. These designations are likely to be very conservative and may belie the imperiled nature of some species. Future studies will seek to formally evaluate conservation status.

### Species delimitation and conceptualization

Although often not discussed, any taxonomic revision contains an implicit concept of a species. The general convention within spider taxonomy is a “diagnosable” species concept (Nelson and Platnick 1981, Nixon and Wheeler 1990) wherein populations are delineated as species as a consequence of sharing a set of qualitative, fixed differences. For the vast majority of spider species described these differences are essentially morphological, usually features related to differences in genitalia (male pedipalps, female spermathecae). Alternatively, one could simply think of this as a “morphological” species construct–if populations differ in a set of morphological characters they are delimited as separate species.

As discussed in number of papers related to species delineation in mygalomorph taxa, morphological stasis seems to be more the rule rather than the exception. That is, the prevailing hypothesis is that extreme geographic structuring due to limited dispersal capability may lead to speciation in the absence of morphological or apparent ecological divergence (Bond et al. 2001). As such a number of studies (e.g., Stockman and Bond 2007, Bond and Stockman 2008, Bailey et al. 2010) advocate that an integrative approach to mygalomorph spider species delimitation must be employed if an accurate representation of evolutionary diversity is to be achieved.

Ideally an integrative approach to species delimitation would use data from many sources (e.g., genetic, ecological, and traditional morphological) taken together to formulate species hypotheses. Such an approach serves to more thoroughly document evolutionary diversity whereas an approach that focuses on a single character system (e.g., genitalic features) may overlook species diversity but is easier to implement and is pragmatic both in terms of species documentation and discovery, and subsequent identification by non-specialists. Reflected in the amount of data available for any given set of specimens/populations, the species reported herein generally represent one of three construct classes–morphological, or traditionally delineated species, phylogenetic species, or “cohesion species”. Traditional morphologically delineated species are defined as those populations that represent qualitative differences in phenotype that differ in a discrete manner from other populations groups. Cohesion species follow Templeton’s (1989) concept wherein a species is defined as lineages that are genetically or demographically interchangeable. Cohesion species are those for which genetic and or ecological data has been considered in concert with the distribution of species (see Bond and Stockman 2008) and morphological characteristics. Each species’ account notes the species concept that has been applied. In a number of instances additional phylogenetic information, based on molecular analyses was available and considered as corroborative support for the species hypothesis being put forth. As such I have applied a phylogenetic species concept wherein individuals (populations) share common ancestry in a molecular phylogenetic analysis and thus are mutually exclusive and diagnosably distinct (Cracraft 1989). Operationally, I simply employ the phylogenetic information from this single gene analysis as corrabotive support for hypothesized morphological species when they are recovered as a clade on the gene tree; that is, these data are not necessarily used exclusively to delineate species.

### Data resources

The data underpinning the analysis reported in this paper (see below) were deposited on 19 November 2012 in the Dryad Data Repository at doi: 10.5061/dryad.3b95n and at GBIF, the Global Biodiversity Information Facility, http://ipt.pensoft.net/ipt/resource.do?r=aptostichus_locality_data . Images associated with species descriptions have been deposited in Morphbank (http://www.morphbank.net ); Morphbank image record numbers are noted in brackets by each figure in the figure legend.

## Results and discussion

### Summary of taxodiversity

At present the genus *Aptostichus* comprises 40 species, 33 of which are newly recognized herein. Table 1 summarizes species, type localities, and material available for each species described; >2000 specimens in total were examined. Of these 33 new species, 12 are known only from male specimens; of these, three are described on the basis of a single specimen. Such a sex-based disparity and lack of material for rare taxa has been noted in taxonomic revisions of other mygalomorph groups (e.g., the migid genus *Moggridgea* O. P. Cambridge, 1875; Griswold 1987) and is not uncommon in systematic works in general (Huber 2003, Lim et al. 2011). As already discussed the number of species will likely increase dramatically as more is learned about this group of spiders; molecular studies to date suggest that “morphological” species, particularly those that are widely distributed, likely disguise a great deal of evolutionary and ecological diversity. Moreover, some underestimation may in part be due to the lack of males collected for populations of unplaced female specimens.

**Table 1.** Summary of *Aptostichus* species diversity. Columns summarize nominal species and conservation status (see footnote); three-letter identifier; US state and county of type locality; latitude and longitude of type locality; characterization of (sex of specimens) and amount of material available for examination.

**Species**	**id**	**State: Co.**	**Lat/Long**	**Material**
*Aptostichus atomarius*^√^	ato	CA: San Bernardino	34.1774, -117.2736	>10; ♂♀
*Aptostichus stephencolberti*^√^	ste	CA: San Mateo	37.2659, -122.4121	>10; ♂♀
*Aptostichus angelinajolieae*^√^	ang	CA: Monterey	36.29045, -121.4659	>10; ♂♀
*Aptostichus miwok*^√^	miw	CA: Humboldt	41.01333, -124.1092	>10; ♂♀
*Aptostichus stanfordianus*^√^	sta	CA: San Mateo	37.4845, -122.3992	>10; ♂♀
*Aptostichus dantrippi*^§^	dan	CA: Kern	35.3947, -119.0313	>10; ♂♀
*Aptostichus pennjillettei*^‡^	pen	NV: Clark	37.0890, -116.0618	>10; ♂
*Aptostichus asmodaeus*^‡^	asm	CA: Contra Costa	37.8530, -121.9291	8; ♂♀
*Aptostichus nateevansi*^‡^	nat	CA: Los Angeles	33.3707, -118.3496	7; ♂♀
*Aptostichus chiricahua*^‡^	chi	AZ: Cochise	31.9136, -109.1408	1♂
*Aptostichus icenoglei*^√^	ice	AZ: Riverside	33.7149, -117.0922	>10; ♂♀
*Aptostichus cabrillo*^§^	cab	CA: San Diego	32.6681, -117.2423	>10; ♂♀
*Aptostichus isabella*^µ^	isa	CA: Kern	35.5689, -118.4383	1♂
*Aptostichus muiri*^µ^	mui	CA: Mariposa	37.4668, -119.9384	1♂, 1♀
*Aptostichus barackobamai*^§^	bar	CA: Mendocino	40.3167, -122.3499	>10; ♂♀
*Aptostichus hesperus*^√^	hes	CA: Los Angeles	34.0968, -117.7195	>10; ♂♀
*Aptostichus hedinorum*^‡^	hed	CA: San Diego	32.7104, -116.1170	>10; ♂♀
*Aptostichus cahuilla*^‡^	cah	CA: Riverside	33.7149, -117.0922	>10; ♂♀
*Aptostichus killerdana*^†^	kil	CA: Orange	33.4819, -117.7206	1♂; 4♀
*Aptostichus serrano*^√^	ser	CA: Riverside	33.9102, -115.9931	>10; ♂♀
*Aptostichus aguacaliente*^√^	agu	CA: Riverside	33.8964, -116.6251	>10; ♂♀
*Aptostichus chemehuevi*^‡^	che	CA: San Bernardino	34.7465, -116.3755	>10♂
*Aptostichus sarlacc*^‡^	sar	CA: San Bernardino	35.7553, -117.5006	2♂
*Aptostichus derhamgiulianii*^‡^	der	CA: Inyo	37.3333, -118.0167	3♂
*Aptostichus mikeradtkei*^√^	mik	CA: San Diego	32.64195, -117.03608	>10; ♂♀
*Aptostichus edwardabbeyi*^§‡^	edw	AZ: Cochise	32.0044, -109.3561	2♂; 1♀
*Aptostichus anzaborrego*^‡^	anz	CA: San Diego	32.86852, -116.23807	2♂
*Aptostichus sinnombre*^‡^	sin	CA: San Diego	32.86923, -116.23740	2♂
*Aptostichus simus*^§‡^	sim	CA: San Diego	32.6346, -117.1400	>10; ♂♀
*Aptostichus satleri*^µ^	sat	CA: Kern	35.5689, -118.4383	4♂
*Aptostichus elisabethae*^§‡^	eli	CA: San Bernardino	34.7465, -116.3755	>10; ♂♀
*Aptostichus fornax*^‡^	for	CA: Inyo	36.09167, -117.2591	1♂, 1♀
*Aptostichus lucerne*^†^	luc	CA: San Bernardino	34.47221, -117.122	2♂
*Aptostichus bonoi*^‡^	bon	CA: San Bernardino	34.0401, -116.3102	1♂, 1♀
*Aptostichus fisheri*^‡^	fis	CA: Kern	35.39752, -117.99797	3♂
*Aptostichus cajalco*^‡^	caj	CA: Riverside	33.8256, -117.4957	>10; ♂♀
*Aptostichus sierra*^‡^	sie	CA: Fresno	37.1129, -119.3095	1♂
*Aptostichus huntington*^‡^	hun	CA: Fresno	37.2379, -119.2295	3♂
*Aptostichus dorothealangeae*^§‡^	dor	CA: Kern	35.3947, -119.0313	>10; ♂♀
*Aptostichus chavezi*^√^	cha	CA: Tulare	36.488, -118.837	>10; ♂♀

Conservation status: secure √; vulnerable §; imperiled ‡; vulnerable/imperiled §‡; presumed to be extinct †; undertermined µ.

The genus *Aptostichus* has diversified within an extensive area that spans the California Floristic Province. Species are found in virtually every habitat type (see discussion of ecological evolution below) including extreme arid desert environments, mesic montane and coastal habitats, to high elevation alpine habitats of the Sierra Nevada Mountains. Without question the genus represents a classical adaptive radiation where lineages have diversified and apparently adapted to inhabit a set of disparate environments, climates, and habitat types. Ecological factors that may influence *Aptostichus* diversity and distributions include climatic suitability during dispersal, prey type and availability, water and temperature, and soil type (to name a few). Given their close ties to the substrate, as fossorial organisms, parameters associated with burrow architecture and design (depth, thickness of silk lining, trap door design, etc.), it is not surprising that many of these features vary from species to species.

Understanding of *Aptostichus* ecology and behavior is severely limited at this time. To date approximately 15 of the 40 species have been collected only from pitfall traps thus female burrows have never been observed. Of these about half are known from only a few specimens and thus are quite rare in collections. Moreover, some species like *Aptostichus derhamgiulianii* and *Aptostichus sierra* have not been collected since they were first discovered over 40 years ago and at least two species are now presumed extinct (*Aptostichus killerdana*, *Aptostichus lucerne*). Alternatively, recent collecting efforts have uncovered a number of new morphologically distinct species (e.g., *Aptostichus satleri*, *Aptostichus isabella*, *Aptostichus cajalco*). Given how narrowly endemic many species are there is likely to be considerable diversity that both awaits discovery but is also threatened due to development and habitat destruction throughout the California Floristic Province biodiversity hotspot.

### *Aptostichus* phylogeny

Figure 28 summarizes the maximum likelihood and Bayesian inference analyses of the mtDNA data set. The data set comprises 337 individual specimens representing 15 of the 40 species documented herein, sequenced for the mitochondrial region spanning the 16S-tRNA-valine-12S genes; of these 206 were newly generated sequences (GenBank Accession numbers: JX103235-JX103440). The matrix is an aligned 1618 base pairs; Kakusan chose the GTRGAMMA model for each of the three partitions. The –ln likelihood value for the best tree from the RAxML analysis is -59821.7255. The Bayesian analysis was run for 40,000,000 generations with half of the trees discarded as burnin. The harmonic and arithmetic means of the post-burnin tree topology likelihoods were -60532.81 and -60373.61, respectively. The summary tree (Fig. 28) supports the basal placement of *Aptostichus simus*, the monophyly of the *Atomarius* Sibling Species Complex, and general placement of other species into species groups delineated on the basis of the morphological analysis (see below). However, as discussed earlier these data were used principally to place undetermined individuals into species rather than formulate hypotheses of deeper relationships across the tree; that is, many of the internal nodes are not strongly supported. Using the results from this analysis in combination with specimens, particularly males that could be identified to species, many female and juvenile *Aptostichus atomarius*, *Aptostichus stanfordianus*, *Aptostichus angelinajolieae*, *Aptostichus icenoglei*, and *Aptostichus barackobamai* specimens were determined. This analysis indicates that *Aptostichus stanfordianus* likely comprises more than one species (previously noted by Bond and Stockman 2008) and that specimens collected from Madera County may be an undescribed species related the *Aptostichus simus* (*A*. sp. Madera, Fig. 28). However, mature males and females are not currently available and thus the status of these specimens remains unanswered.

The morphological matrix comprised 72 characters scored for all 40 *Aptostichus* species. Figures 17–27 summarize the quantitative character scorings for all taxa but *Aptostichus satleri*, *Aptostichus isabella*, and *Aptostichus sinnombre*. Table 2 summarizes the results for the parsimony analysis based on characters equally weighted (EW) and the implied weighting analyses (IW) using a range of concavity function constants (k=3-12). The EW analysis resulted in > 440,000 trees comprising 238 steps whereas the implied weights analyses resulted in considerably fewer trees with tree lengths ranging from 238–241 steps.

**Table 2.** Summary of results from phylogenetic analyses of the morphological data set scored for all 40 *Aptostichus* and six outgroup taxa. Table summarizes the number of trees, steps, consistency (CI) and retention indices (RI) and Goloboff fit (Gfit) values recovered from parsimony analyses employing equal weights (wts.) and the Goloboff fit criterion (with array of concavity function constants).

**Analysis**	**# trees**	**Steps**	**CI**	**RI**	**Gfit**
Equal wts.	446684	238	0.353	0.764	---------
k=3	17222	241	0.349	0.760	-52.564
k=4	17210	241	0.349	0.760	-55.071
k=5	17168	241	0.349	0.760	-57.007
k=6	453	239	0.351	0.763	-58.562
k=7	824	239	0.351	0.763	-59.836
k=8	493	239	0.351	0.763	-60.898
k=9	294	239	0.351	0.763	-61.798
k=10	296	238	0.353	0.764	-62.574
k=11	245	238	0.353	0.764	-63.248
k=12	228	238	0.353	0.764	-63.839

The preferred tree topology is based on the analysis using IW with a concavity function constant of k=7 (Fig. 29). The EW and IW analyses were moderately incongruent with respect to the tree topologies. The EW analysis failed to recover an *Aptostichus* clade that was monophyletic with respect to *Apomastus*. A strict consensus of the >440,000 resulted in a largely unresolved tree (towards the tips) that recovered the *Sierra*, *Simus*, and *Hesperus* clades, or species groups (Fig. 29); the *Atomarius* clade was paraphyletic with respect to the *Hesperus* clade. Implied weighting analyses that employed concavity function constants k=3-5 resulted in trees that recovered a monophyletic *Aptostichus*, *Sierra*, *Simus*, and *Hesperus* clades but like the EW analysis the *Atomarius* clade was paraphyletic. Tree topology stabilized for IW analyses where k=6-12 recovering a pattern similar to that illustrated in Figure 29 where all four major species groups are monophyletic. These clades are largely delineated on the basis of shared male mating clasper and pedipalp differences (illustrated for each species group in Fig. 29).

Both molecular and morphological data matrices and associated trees (all trees recovered and consensus), formatted as Nexus and Phylip files can be downloaded from the Dryad Data Repository at doi: 10.5061/dryad.3b95n..

### Character support of major clades and *Aptostichus* species groups

Table 3 summarizes the unambiguous character state support for each of the major nodes in the preferred tree topology (Fig. 29). I summarize below the support for only the major nodes in the analysis and formally diagnose the four nominal *Aptostichus* species groups. At this time it would be premature to overemphasize resolution among all of the terminal relationships within *Aptostichus* because of the incomplete nature of the data set due to missing taxa and few female representatives of some species.

**Table 3.** List of unambiguous character state changes for major clades recovered in the preferred tree topology using implied weights (k=7).

**Clades**	**Characters and state changes**
*Aptostichus* monophyly	**19:** 0→1; **31:** 0→1; **58:** 0→1; **59:** 0→1
*Simus* + *Sierra*	**42:** 1→0; **51:** 1→0
*Sierra*	**8:** 0→2; **33:** 0→1;
*Simus*	**14:** 1→0; **41:** 1→0; **43:** 0→1; **52:** 0→1; **53:** 0→1; **60:** 0→1
*Atomarius* + *Hesperus*	**48:** 0→1; **57:** 0→1; **62:** 0→1; **63:** 0→2; **64:** 0→1; **65:** 2→0
*Atomarius*	**2:** 1→2; **38:** 0→1; **71:** 0→1
*Hesperus*	**11:** 0→1; **18:** 1→0; **20:** 1→0; **72:** 0→1; **74:** 0→1

Four characters (given parenthetically following the description of the state) provide unambiguous support for the monophyly of *Aptostichus*: a mottled, striped abdominal color pattern (19), distal 1/2 of the male metatarsus I lighter in color (31), extended prolateral cymbial lobe (58), and a cymbium with spines (59). Two characters support the monophyly of the clade that comprises the *Sierra* and *Simus* species groups: a short male palpal tibia (42), and an embolus with a single distinct bend (51). Six synapomorphies support the node that unites the *Hesperus* and *Atomarius* species groups: a anteverted male metatarsus I (48), a long palpal bulb (57), the presence of a male retrolateral distal tibial spine (62), long male retrolateral distal tibial spines (63), a triangular male mating apophysis (64), and uniform, non-overlapping male retrolateral distal tibial spines (65).

*Sierra* *Species Group*.Four species comprise the *Sierra* species group, which is supported by two synapomorphies: long sternum (8) and a long male metatarsus I (33).

*Sierra* *Species Group*.Eight species comprise the *Simus* species group, the monophyly of which is supported by six synapomorphies: absence of cuspules on male endites (14), male palpal tibia stout (41), male palpal tibia spines short and positioned retrolaterally (43), stout embolus that is dorsal - ventrally compressed (52, 53), and retrolateral, distal most aspect of the cymbium formed as a distinct process (61).

*Hesperus* *Species Group*.Thirteen species comprise this diverse species group. The key distinguishing feature of this group is the presence of an offset retrolateral rastellar spine (character 11). Additionally, four other characters support the monophyly of this species group: lighter carapace and abdominal coloration (18, 20) and a long and sinuous spermathecal stalk (72, 74).

*Atomarius* *Species Group*.Fifteen species comprise the *Atomarius* species group, the monophyly of which is supported weakly by three synapomorphies: heavy carapace pubescence (2), dense female tarsal scopulae (38) and a distinct secondary spermathecal bulb (71).

### Desert adaptation in *Aptostichus*

**Figure 30.**
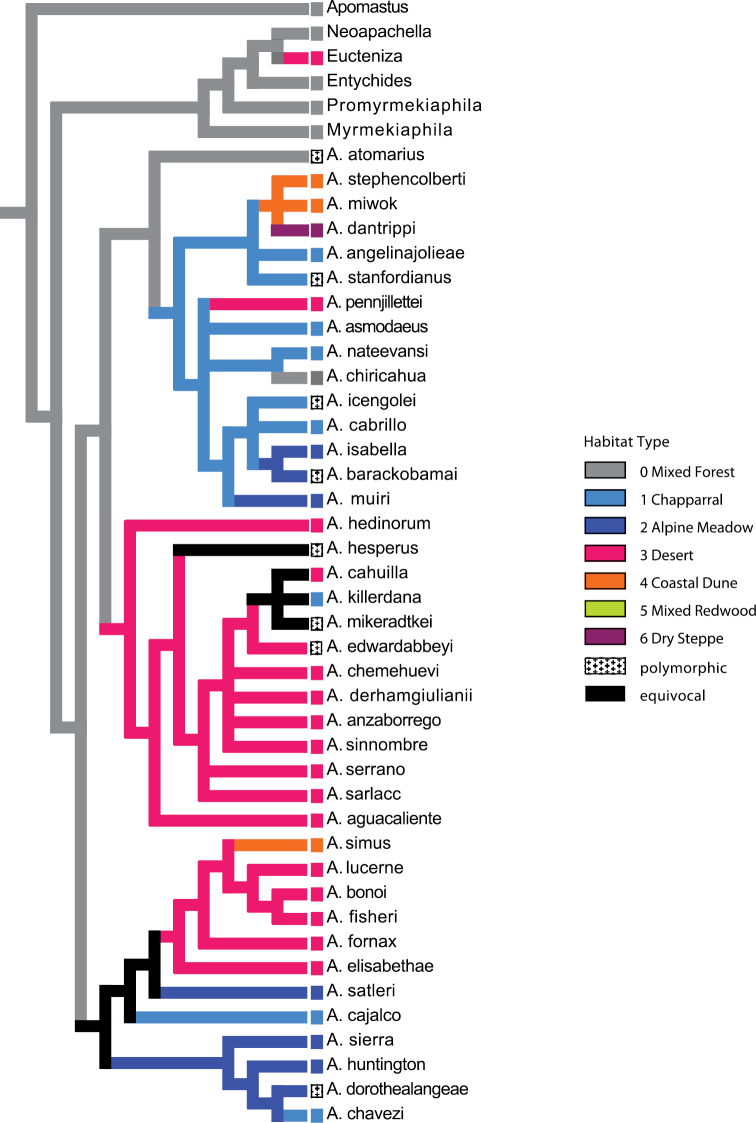
Optimization of habitat type on preferred tree topology (morphology, implied weighting, k=7).

*Aptostichus* is an ideal group for evaluating changes in spider morphology and behaviors associated with invasions of arid, desert and other habitat types (e.g., coastal dunes). Figure 30 maps habitat type on the preferred tree topology (Fig. 29) using parsimony; alternative optimization criteria were not possible due to the polytomies in the preferred tree. The current phylogeny requires at least three independent derivations of strictly desert habitation for 15 species occurring in three of the four species groups. Additional independent derivations of arid habitat are required if chaparral is classified similarly to desert habitats (i.e., all arid habitats are grouped together).

Main (1978) suggested eight adaptations that may allow trapdoor spiders to survive in very arid habitats: 1) larger body size, 2) deeper burrows, 3) increasing foraging area achieved by burrow rim modifications, 4) differential timing of breeding and dispersal, 5) the tendency of brooding females to plug their burrows, presumably for water retention, 6) aestivation of young in sealed burrows, 7) mature non-brooding females that do not plug burrows and are therefore able to feed sporadically, and 8) increased longevity of females. Concentrated efforts to obtain female specimens and additional natural history data will be required to address these questions more thoroughly for this group. Moreover, a complete molecular phylogeny for the genus will likely go further to resolve relationships among the species and will help to abrogate any confounding issues that morphological characters and attendant homoplasy associated with habitat may have.

## Taxonomy

### Family Euctenizidae

Raven, 1985

urn:lsid:zoobank.org:act:C27FB688-5D8E-4E77-ABCC-FD108DC4C22D

http://species-id.net/wiki/Euctenizidae

#### Type genus:

*Eucteniza* Ausserer, 1875

### Subfamily Apomastinae

Bond & Hedin subfam. n.

urn:lsid:zoobank.org:act:5C533E5D-0359-45F8-B37E-3BA34CC66303

#### Type genus.

*Apomastus* Bond & Opell, 2002

#### Note.

Defined as a euctenizid subfamily comprising the genera *Myrmekiaphila*, *Apomastus*, and *Aptostichus* in Bond et al. (2012b), the new designation, despite considerable phylogenetic support was dismissed as a nomen nudum in an online catalog (Platnick 2012) due to the absence of a formal diagnosis. To correct this oversight, I formally diagnose below the newsubfamily Apomastinae and again provide a list of included genera. As was originally intended, authorship is to be attributed to Bond and Hedin.

#### Diagnosis.

Apomastinae, a lineage defined in extensive phylogenetic analyses that include multiple lines of evidence that comprises genes and morphology (Bond and Hedin 2006; Bond et al. 2012b) can be morphologically distinguished from all other euctenizids by having a patch of endite cuspules that is restricted to the proximal inner margin (Fig. 38; rather than being uniformly distributed across the endite face, see Stockman and Bond 2008, fig. 10) and by having two distinct posterior median spinneret spigot types (as opposed to a single type, Stockman and Bond 2008, fig. 24).

**Figures 31–38.**
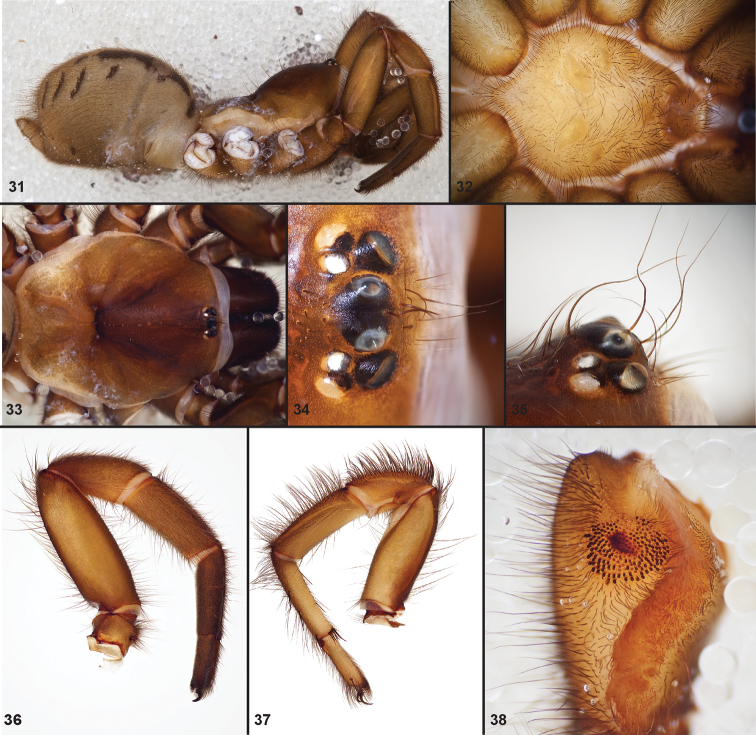
Standard light microscopy views of female an *Aptostichus simus* specimen (MY3432). **31 **side view **32** ventral view, sternum **33** dorsal view, carapace **34–35** eye group, dorsal and lateral views **36** leg I prolateral view **37** leg IV retrolateral view **38** palpal endite.

#### Included genera.

*Myrmekiaphila* Atkinson, 1886

*Aptostichus* Simon, 1891

*Apomastus* Bond and Opell, 2002

### Genus Aptostichus

Simon, 1891

urn:lsid:zoobank.org:act:4AE50840-DF3D-4EF9-B7F9-5E12286E4FB2

http://species-id.net/wiki/Aptostichus

**Map 1.**
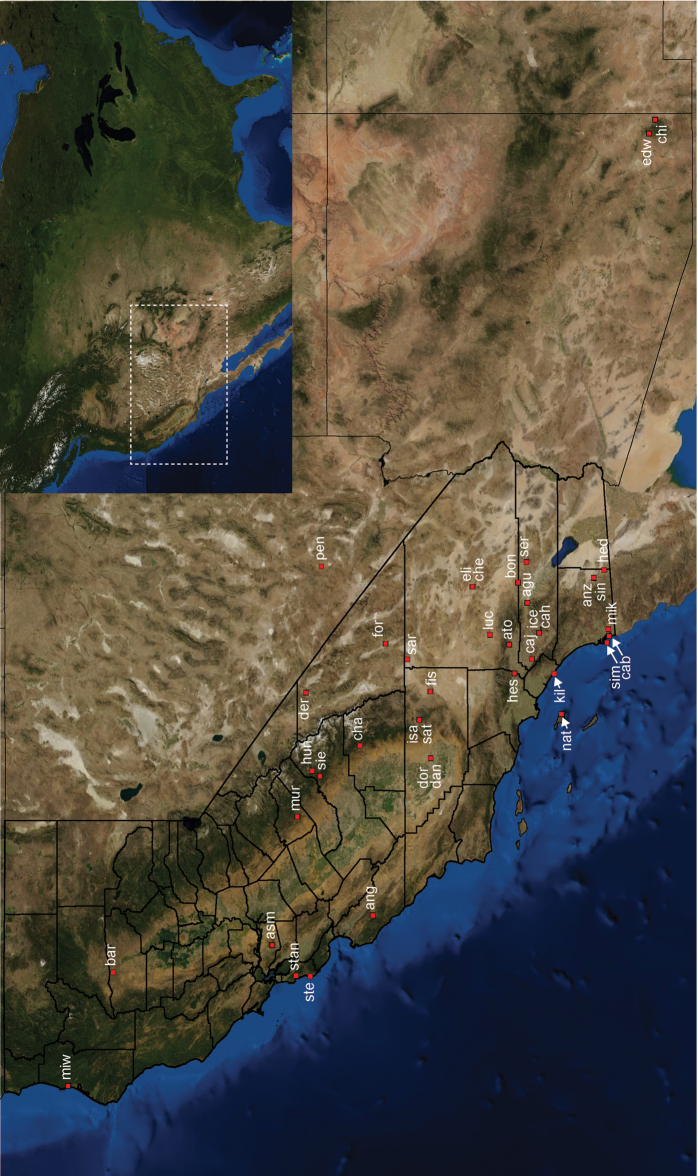
Distribution of type localities for all known species of *Aptostichus*. Three letter identifiers are defined in Table 1; inset shows approximate scope of study area.

Figs 31 [Fig F11] [Fig F12] –68 Map 1

Aptostichus Simon, 1891: 317 (type species by monotypy *Aptostichus atomarius* female lectotype from CA, San Bernardino; specimen AR4263 in MNHP, examined).–E. Simon 1892: 108.–E. Simon 1903: 901.–P. Smith 1908: 220–221–Bond & Opell 2002.
Actinoxia Simon, 1891: 318 (type species by monotypy *Actinoxia versicolor* Simon juvenile holotype in MNHP, examined).–E. Simon 1892: 109. P. Smith 1908: 214 (Smith considered *Actinoxia* to be a junior synonym of *Entychides* Simon).–R. Chamberlin 1937: 9.–Synonymized by Bond & Opell 2002: 518.
Nemesoides Chamberlin, 1919: 1–2 (*Nemesoides hespera* Chamberlin female holotype in MCZ, examined).–Synonymized by Bond & Opell 2002: 518.

#### Diagnosis.

**Figures 39–50. F11:**
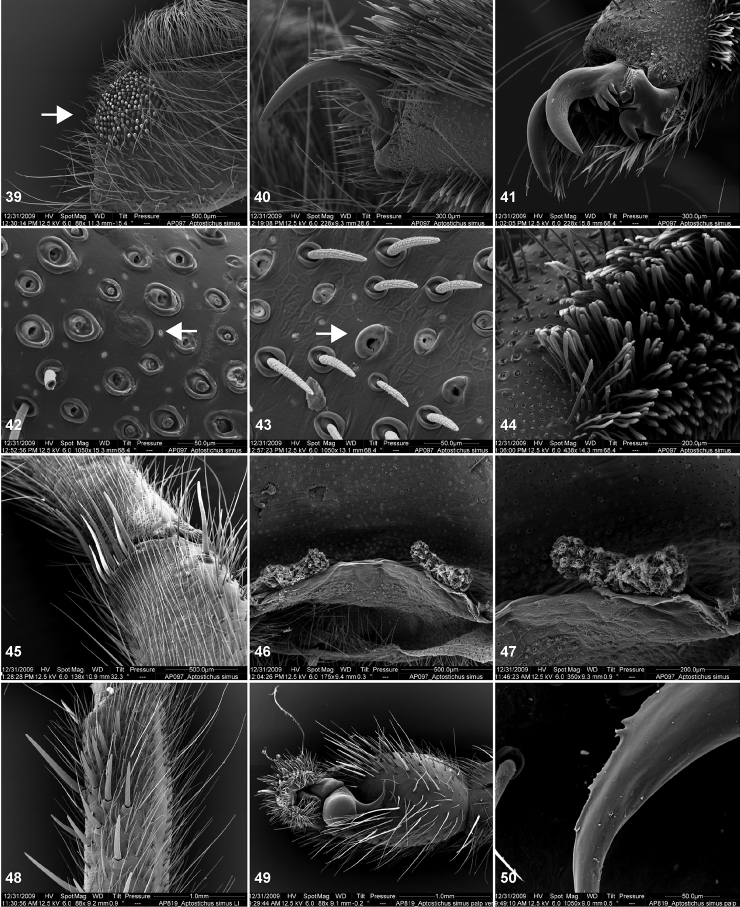
Scanning electron micrographs of *Aptostichus simus* specimens (female AP097, **39–47**; male AP819, **48–50**). **39** palpal endite, arrow indicates cuspule pattern **40** left pedipalp claw, retrolateral aspect **41** leg I, tarsal claws, retrolateral aspect **42** leg I, tarsus, dorsal aspect, tarsal organ (arrow) **43** leg I, tarsus, dorsal aspect, trichobothrial base (arrow) **44** leg I, tarsal scopulae **45** leg IV, ventral junction of tarsus and metatarsus, preening comb **46, 47** cleared spermathecae and close up of left spermathecal lobe **48** leg I, retrolateral distal aspect of tibia **49** pedipalp, ventral aspect **50** serrated edge of palpal embolus.

**Figures 51–56. F12:**
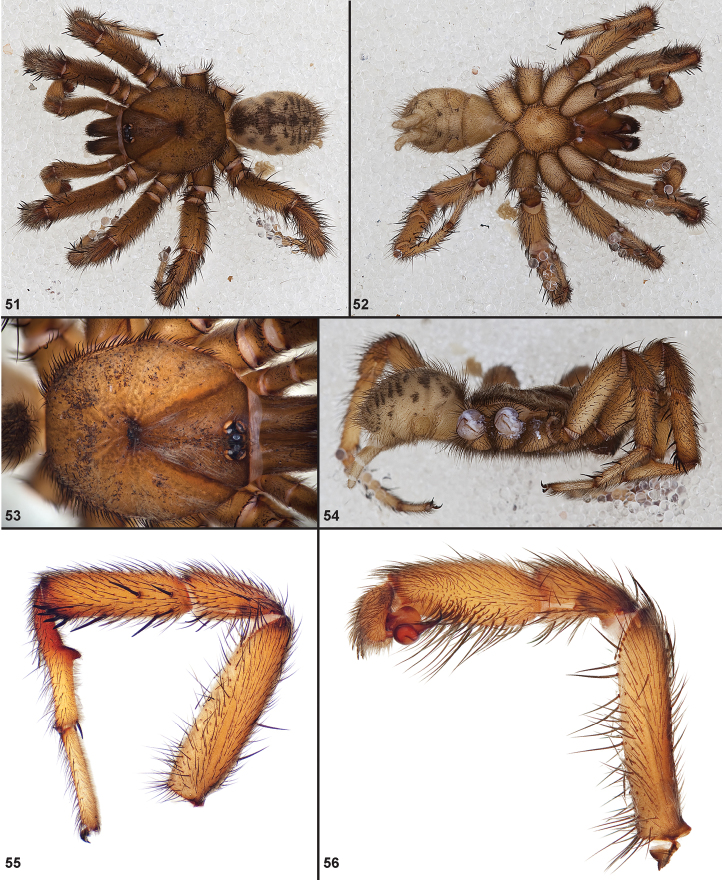
Standard light microscopy views of *Aptostichus atomarius* male specimens (**51–54** MY2979; **55, 56** AP357). **51** dorsal habitus view **52** ventral habitus view **53** carapace **54** lateral habitus view **55** leg I, retrolateral aspect **56** pedipalp, retrolateral aspect.

Males of this genus can be recognized by the presence of three or more spines on the distal most surface of the palpal cymbium (Figs 49, 56, 68) and a number of large, very thick spines on the distal-prolateral aspect of tibia I (Figs 48, 55). Tibia I spines are more offset proximally in the *Simus* and *Sierra* group species (Fig. 55). *Entychides* males have similar spination, however their spines are borne on a low apophysis whereas those of *Aptostichus* are not (Bond and Opell 2002). *Aptostichus* females have cuspules on both the labium and palpal endites; labial cuspules are generally few and/or restricted to the inner margins of the endites (Figs 38, 39). This condition is similar to that for *Apomastus*, however *Apomastus* species appear to lack labial cuspules and the distinctive *Aptostichus* abdominal coloration, which consists of a mottled chevron pattern (Fig. 51).

#### General description.

Small to medium sized trapdoor spiders. Cephalothorax longer than wide, sloping posteriorly, moderate pubescence in most species (Figs 31, 54). Carapace sclerotization equal across its length. Thoracic groove intermediate to wide, procurved or straight (Fig. 33) and deep. In some males the thoracic groove is transverse or recurved (Fig. 51). Carapace of males fringed in stout black setae (Fig. 53). Eyes on a low tubercle (Fig. 35). AME and PME subequal diameter, except in a few species, particularly in some *Simus* group species where the PME diameter is noticeably less than that of AME. PME row slightly procurved or straight, AME row slightly recurved (Fig. 34). Caput moderately high (Fig. 31). Carapace of ethanol preserved specimens appears orangish-yellow. The coloration of living spiders tends to be a darker brown, however there is considerable variation in the intensity of coloration. Male coloration in most specimens is dark reddish-brown. Female and male abdominal coloration very distinctive consisting of light brown or gray background with a dark mottled chevron like pattern (Fig. 51). This pattern is less distinctive in *Aptostichus simus*, closely related species and is reduced in most desert-adapted species.

**Figures 57–68.**
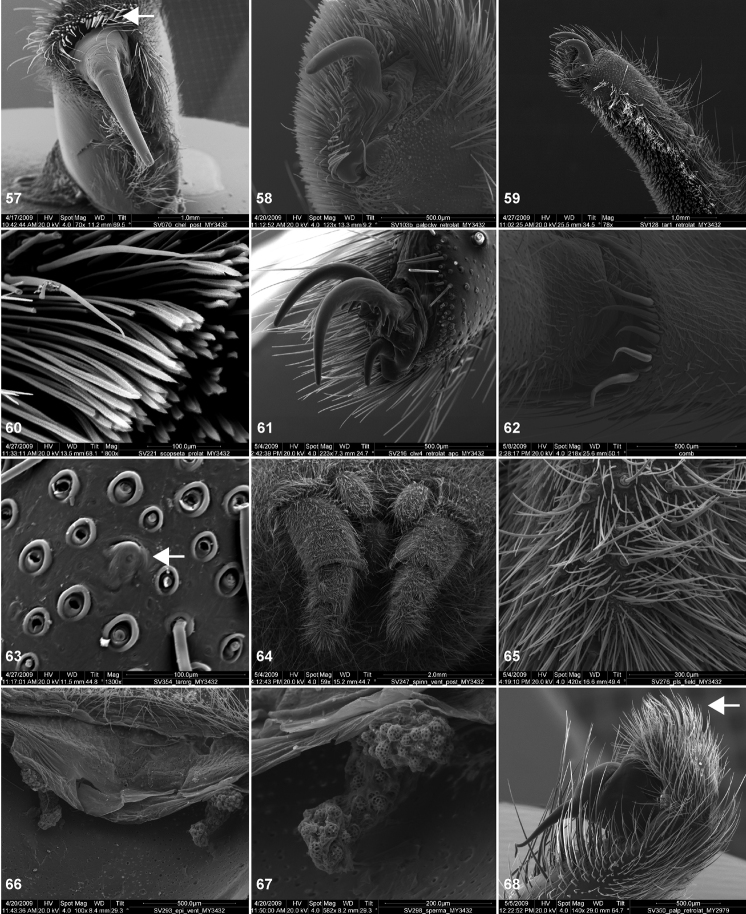
Scanning electron micrographs of *Aptostichus atomarius* specimens (female MY3432, **57–67**; male MY2979 **68**). **57** left chelicerae, anterior ventral aspect **58** left pedipalp claw, retrolateral aspect **59** leg I, tarsal claw, retrolateral aspect **60** tarsal scopulae, leg I **61** leg IV tarsal claws, retrolateral aspect **62** leg IV preening comb, tarsus/metatarsus junction **63** leg I, tarsus, dorsal aspect, tarsal organ **64 **spinnerets **65** spinning field, distal aspect of PLS **66, 67** cleared spermathecae and close-up of left lobe **68** pedipalp, retrolateral aspect, arrow indicates position of spines on cymbium.

Sternum wider posteriorly, sometimes wider than in other euctenizids, tapering anteriorly (Figs 32, 52). Posterior sigilla large and positioned mid-posteriorly in most species (Fig. 32), in some species contiguous (e.g., *Aptostichus hesperus*). Anterior margin of sigilla has a rounded margin. Palpal endites longer than wide often with only a few cuspules, which are restricted to the posterior margin, except in *Aptostichus simus* that has many cuspules arranged in a characteristic pattern (Figs 38–39). Labium wider than long, with a few, to a moderate number of cuspules (Fig. 32). Chelicerae dark brown. Rastellum consists of numerous spines not borne on a distinctive mound (Fig. 57). Fangs long and slender. Cheliceral furrow promargin with row of very large teeth. Retromarginal row consists of a patch of denticles.

Apical PLS article short, digitiform (Fig. 64). Spinnerets mostly with pumpkiniform spigots with several articulated spigots interspersed on apical and median articles of PLS and the PMS (Fig. 65). Two to three large, articulated spigots on apical most aspect of the PLS. PMS article robust. See Bond and Opell (2002) for more detailed descriptions of these spigot types.

Anterior leg articles slender relative to posterior. Tarsi short and robust (Fig. 36). Female scopulae long, dense, asymmetrical, extending full length of tarsus, no further than the metatarsus (Figs 36, 44, 60). Scopulae extend no further than the tarsus of the pedipalp. Posterior legs lack distinct scopulae. Pedipalp claw with a few (Fig. 40) to many teeth (Fig. 58). Male tarsi I and II with short sparse scopulae that are restricted to the ventral surface. In some species male tarsi are slightly bent, elongate and pseudosegmented (e.g., *Aptostichus simus*). Basal palpal tooth and STC I–IV basal tooth elongate and positioned on the median keel but not bifid (Figs 41, 59, 61). STC IV with 5 or more teeth (Fig. 61). Female anterior legs with very few ventral spines (Fig. 36). Prolateral surface of female patella III covered in numerous thick spines. Distal ventral aspect of tarsus IV with short, sparse spine patch. Preening combs on distal most retrolateral surface of metatarsus IV (Figs 37, 45, 62). Tarsal trichobothria arranged in a zigzag pattern with typical base (Fig. 43); low tarsal organ with central pit (Figs 42, 63). Spermathecae with an elongate base that forms a secondary spermathecal bulb in some species (Figs 46, 47, 66, 67).

Male mating clasper morphology is distinctive. Articles of leg I bear a number of large, thickened spines positioned retrolaterally on the distal aspect of the tibia (Fig. 55), except members of the *Sierra* and *Simus* species groups whose tibial spines are more concentrated proximally (Fig. 55). In most species, metatarsus I with proximal ventral to prolateral excavation bordered distally by a low mound (Fig. 55). Tibia I with 3-5 elongate spines distributed retrolaterally except in some species which have denser spine patches. Palpal cymbium with four or more dorsal spines (Figs 49, 56, 68). Palpal bulb normal (Fig. 68), embolus of some *Simus* group species with serrations (Fig. 50). Palpal femur short with a dorsal row of thin spines, tibia short and robust in some species (e.g., *Aptostichus simus*) there is a distinctive prolateral spine patch on the palpal tibia.

#### Distribution.

Distributed primarily throughout the California Floristic Province with the greatest number of species known from Southern California; a few species are recorded from Nevada and Arizona (Table 1, Map 1).

#### Key to species groups and to males

*Note*. Like many mygalomorph taxa, species identification is a non-trivial task. These spiders generally lack distinctive somatic differences that render development of a key to females virtually impossible and a key to males difficult at best. Although, I have attempted to provide a key to male specimens, I would caution that it is far from perfect and thus suggest that the key be used in conjunction with careful examination of specimens, the species description, knowledge of from where the specimen was collected (many species are narrowly endemic), and molecular characters (if available). Generally speaking no single characteristic should be taken as definitive evidence of a species’ determination. Because species placed in the *Atomarius* Sibling Species Complex have been delineated on the basis of a combination of biogeographic, general somatic, ecological, and molecular characteristics, the provided key to these taxa relies heavily on data taken from the geographic location and habitat from which the specimen was collected.

**Table:** 

1	Mid - ventral apophysis of metatarsus I triangular (Fig. 69), knob-like (Fig. 128), or absent (Fig. 333)	4
–	Mid - ventral apophysis of metatarsus I rectangular (Fig. 147)	*Atomarius* species group, in part- 2
2	Tibia I retrolateral spine(s) arranged along the distal most retrolateral aspect of the article (Fig. 147)	3
–	Tibia I retrolateral spines slightly behind (proximal) the distal most aspect of the article (Fig. 165)	*Aptostichus isabella*
3	TSrd > 4	*Aptostichus cabrillo*
–	TSrd≤ 3	*Aptostichus icenoglei*
4	Tibia I spines arranged along the distal most retrolateral aspect of the article, and/or with prolateral spines arranged in one or two rows along the prolateral surface of tibia I (Fig. 69)	16
–	Tibia I spines absent along the most distal retrolateral aspect with the prolateral spines not arranged in such a fashion (Fig. 275)	5
–	Tibia I spines slightly behind (proximal) the distal most retrolateral aspect of the article, prolateral spines arranged in a single row along the medial prolateral surface of tibia I. Numerous distal tibia I spines (TSrd) offset slightly proximal from the distal margin (Fig. 175)	*Aptostichus barackobamai*
5	Palpal endites lack cuspules, PTw/PTl > 0.5, palpal tibia spines short and prolaterally positioned, embolus short, thick and appears to be compressed in the dorsal/ventral plane (Figs 277, 278)	*Simus* species groups- 6
–	Palpal endites with cuspules, PTw/PTl < 0.5, palpal tibia spines long and ventrally positioned, palpal tibia with very distinct megaspines on the mid - retrolateral region, embolus thin (Figs 339, 341)	*Sierra* species group- 13
6	Sternum longer than wide, widest point usually between coxae III and IV (Fig. 283)	7
–	Sternum as wide as it is long, generally appears to be round in shape	*Aptostichus cajalco*
7	TSr < 10	8
–	TSr > 10	9
8	AME and PME subequal in diameter	*Aptostichus satleri*
–	PME less than AME in diameter	*Aptostichus elisabethae*
9	Embolus lacks serrations	*Aptostichus fornax*
–	Embolus serrated (Fig. 306)	10
10	Tarsus I lacks ventral spines, also lacks elongate ventral tibia I spines (Fig. 275)	*Aptostichus simus*
–	Tarsus I with short ventral spines (Figs 317, 322)	11
11	AME & PME diameter subequal, MA4/MF4 ≥ 60	*Aptostichus lucerne*
–	AME diameter less than PME diameter, MA4/MF4 < 60	12
12	MT1/MF1 > 79	*Aptostichus fisheri*
–	MT1/MF1 < 75	*Aptostichus bonoi*
13	TSr > 18	*Aptostichus chavezi*
–	TSr ≤ 18	14
14	Sternum noticeably long and thin (Fig. 340)	15
–	Sternum shape normal	*Aptostichus sierra*
15	Tarsus I and IV straight, PTl/Cl > 3	*Aptostichus dorothealangeae*
–	Tarsus I and IV curved, PTl/Cl < 3.6	*Aptostichus huntington*
16	Rastellum with a single spine offset retrolaterally (Fig. 189)	*Hesperus* species group- 17
–	Rastellum lacking an offset retrolateral spine	*Atomarius* species group- 29
17	Coloration darker, non psammophilic form, mottled abdominal striping (Fig. 191)	18
–	Coloration lighter, psammophilic form, abdominal striping reduced to just a few stripes or mottled blotches (Fig. 197)	22
18	Sternal sigilla contiguous (Fig. 188)	*Aptostichus hesperus*
–	Sternal sigilla separated	19
19	Sternum as wide as it is long, appears round and raised (Fig. 256)	*Aptostichus mikeradtkei*
–	Sternum longer than wide with widest point between coxae III and IV	20
20	Leg I retrolateral distal tibia spine pattern comprises < 4 spines, non-overlapping	*Aptostichus killerdana*
–	Leg I retrolateral distal tibial spine pattern comprises > 3 spines that overlap	21
21	Cl < 4.4, collected from southern California	*Aptostichus cahuilla*
–	Cl > 4.8, collected from southeastern Arizona	*Aptostichus edwardabbeyi*
22	Retrolateral surface of cymbium with spines (Figs 246, 248)	23
–	Retrolateral surface of cymbium lacks spines	25
23	AME and PME subequal in diameter	*Aptostichus sarlacc*
–	PME less than AME in diameter	24
24	Metatarsus mating apophysis armed with a distinct spine, tarsus convex in shape (Fig. 266)	*Aptostichus sinnombre*
–	Metatarsus mating apophysis not armed with a distinct spine, tarsus straight	*Aptostichus derhamgiulianii*
25	4 or more spines arranged along dorsal/prolateral surface of leg I metatarsus, spines form a distinct row	26
–	3 or fewer spines arranged along dorsal/prolateral surface of leg I metatarsus, not forming a distinct row	27
26	Distinct spine patch on prolateral surface of leg I patella, comprising multiple heavy spines (> 8) (Fig. 264)	*Aptostichus anzaborrego*
–	Leg I patella armed with only a few thin spines (usual condition, < 3)	*Aptostichus chemehuevi*
27	Innermost rastellar spines are much larger than those more prolaterally positioned	*Aptostichus serrano*
–	Innermost and prolateral spines are equal in size	28
28	Tibia I retrolateral distal spination pattern consists of multiple spines (> 3), often overlapping and a metatarsal mating apophysis armed with a single small spine	*Aptostichus aguacaliente*
–	Tibia I retrolateral distal spination pattern comprises 3 or fewer spines, metatarsal mating apophysis not armed with a spine	*Aptostichus hedinorum*
29	Metatarsus mating apophysis triangular in shape	30
–	Metatarsus mating apophysis rounded or knob-like (Fig. 128)	*Aptostichus asmodaeus*
30	Dense, heavy spination on leg I prolateral surface of tibia and patella (Fig. 139)	31
–	Spination on prolateral surface of leg I patella and tibia comprises only a few spines	32
31	Metatarsus leg I with a distinct row of heavy dorsal spines, collected from southeastern Arizona	*Aptostichus chiricahua*
–	Metatarsus leg I lacks a distinct row of heavy dorsal spines, collected from central California	*Aptostichus nateevansi*
32	Lightly colored carapace and abdomen, abdominal striping reduced comprising a set of light distinct bands, found in western Nevada (Fig. 120)	*Aptostichus pennjillettei*
–	Carapace and abdomen usually darkly pigmented, abdomen with distinct heavy, mottled pattern of stripes (e.g., Fig. 106)	33
33	3 or fewer distal retrolateral tibial spines on leg I, palpal tibia slender, distributed further to the north in California, proximity of the Yosemite Valley	*Aptostichus muiri*
–	More than three distal retrolateral tibial spines on leg I, palpal tibia more robust, distributed throughout southern California and the coastal ranges to the west	*Atomarius* Sibling species complex- 34
34	Collected from inland habitats, usually (but not always) darker in color with distinct mottled abdominal striping pattern (Fig. 81)	35
–	Collected from coastal dune habitat, always lighter in coloration and generally lacking distinct mottled abdominal striping pattern (Fig. 82)	37
35	Restricted to the Monterey Bay area, distributed west of the Salinas River Valley	*Aptostichus angelinajolieae*
–	Not restricted to the Monterey Bay area, found east and south of the Salinas River Valley	36
36	Known from along the banks of the Kern River (Kern Co., Bakersfield, California) and the southern extent of the Transverse Ranges. Specimens from the Bakersfield area often much lighter in coloration with significantly reduced abdominal patterning	*Aptostichus dantrippi*
–	Distributed throughout the coastal ranges of central California, bounded to the east by the Central Valley	*Aptostichus stanfordianus*
–	Widely distributed throughout southern California	*Aptostichus atomarius*
37	Distributed in California coastal dune habitat from San Luis Obispo County northward to San Francisco County	*Aptostichus stephencolberti*
–	Distributed in California coastal dune habitats from Marin County northward to Humboldt County and on Farallon Island	*Aptostichus miwok*

#### The *Atomarius* species group

**Included species.**

*Aptostichus atomarius* Simon, 1891

*Aptostichus stephencolberti* Bond, 2008

*Aptostichus angelinajolieae* Bond, 2008

*Aptostichus stanfordianus* Smith, 1908

*Aptostichus miwok* Bond, 2008

*Aptostichus dantrippi* Bond sp. n.

*Aptostichus pennjillettei* Bond sp. n.

*Aptostichus asmodaeus* Bond sp. n.

*Aptostichus nateevansi* Bond sp. n.

*Aptostichus chiricahua* Bond sp. n.

*Aptostichus icenoglei* Bond sp. n.

*Aptostichus cabrillo* Bond sp. n.

*Aptostichus isabella* Bond sp. n.

*Aptostichus muiri* Bond sp. n.

*Aptostichus barackobamai* Bond sp. n.

**The *Atomarius* Sibling Species Comple*x***

The *Atomarius* Sibling Species Complex (Bond and Stockman 2008) comprises six closely related sibling species: *Aptostichus atomarius*, *Aptostichus stephencolberti*, *Aptostichus angelinajolieae*, *Aptostichus stanfordianus*, *Aptostichus miwok* Bond, and *Aptostichus dantrippi* sp. n. With the exception of a number of coastal dune and desert-adapted lineages these species are relatively morphologically homogenous (see analysis of morphological variation in Bond and Stockman 2008) and are difficult to distinguish; modifications to the male leg I tibia and metatarsus are indistinguishable among species. However, all species in this complex have non-overlapping ranges and thus can be ascribed to species on the basis of collecting locality and habitat; examination of diagnostic molecular markers should be employed to definitively confirm any determinations made solely on the basis of geography. Molecular studies to date further indicate *Aptostichus atomarius*, as defined below, may actually comprise 2–3 species; sampling at the time was insufficient to formulate these additional hypotheses with rigor.

### Aptostichus atomarius

Simon, 1891

‘The San Bernardino Hills Trapdoor Spider’

urn:lsid:zoobank.org:act:CCA6EC4B-4050-4B06-B056-DF8D666F93DC

http://species-id.net/wiki/Aptostichus_atomarius

**Figures 69–75.**
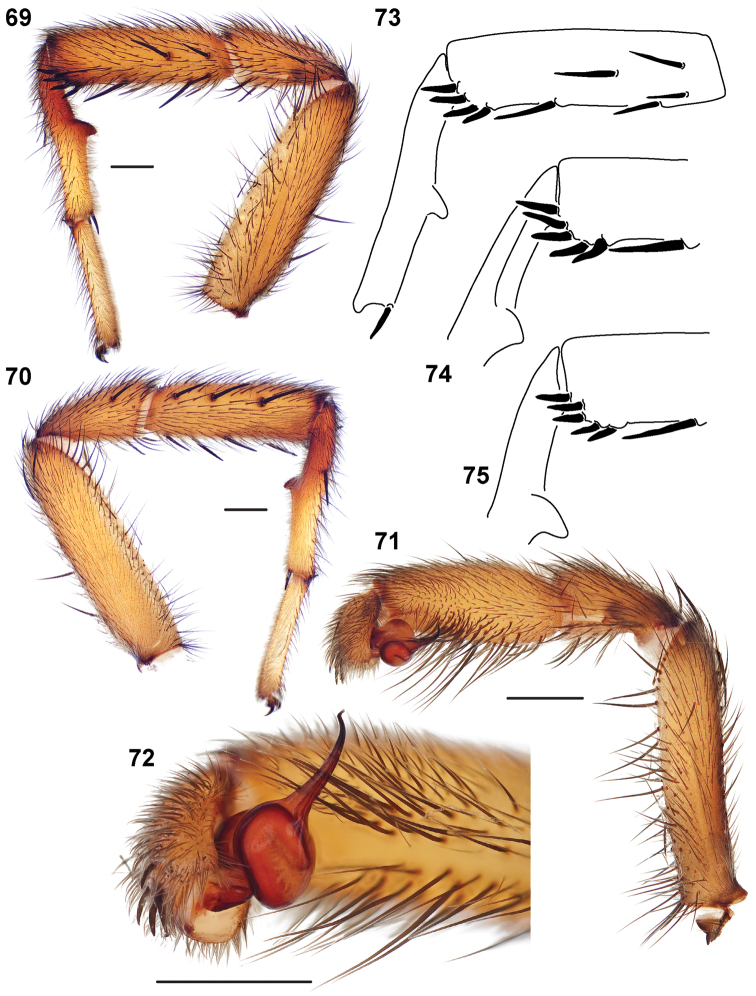
*Aptostichus atomarius* Simon, 1891. **69–72** Specimen AP357 from San Bernardino Co., San Bernardino; scale bar = 1.0mm **69** retrolateral aspect leg I [805733] **70** prolateral aspect leg I [805737] **71** retrolateral aspect pedipalp [805743] **72** ventral aspect palpal bulb [805745] **73–75** line drawings of spination patterns on metatarsus and tibia, leg I, retrolateral aspect **73** Los Angeles Co., Baldwin Hills (AP181) **74** San Luis Obispo Co., San Luis Obispo (AP172) **75** Los Angeles Co., Eaton Canyon Park (AP157).

**Maps 2, 3.**
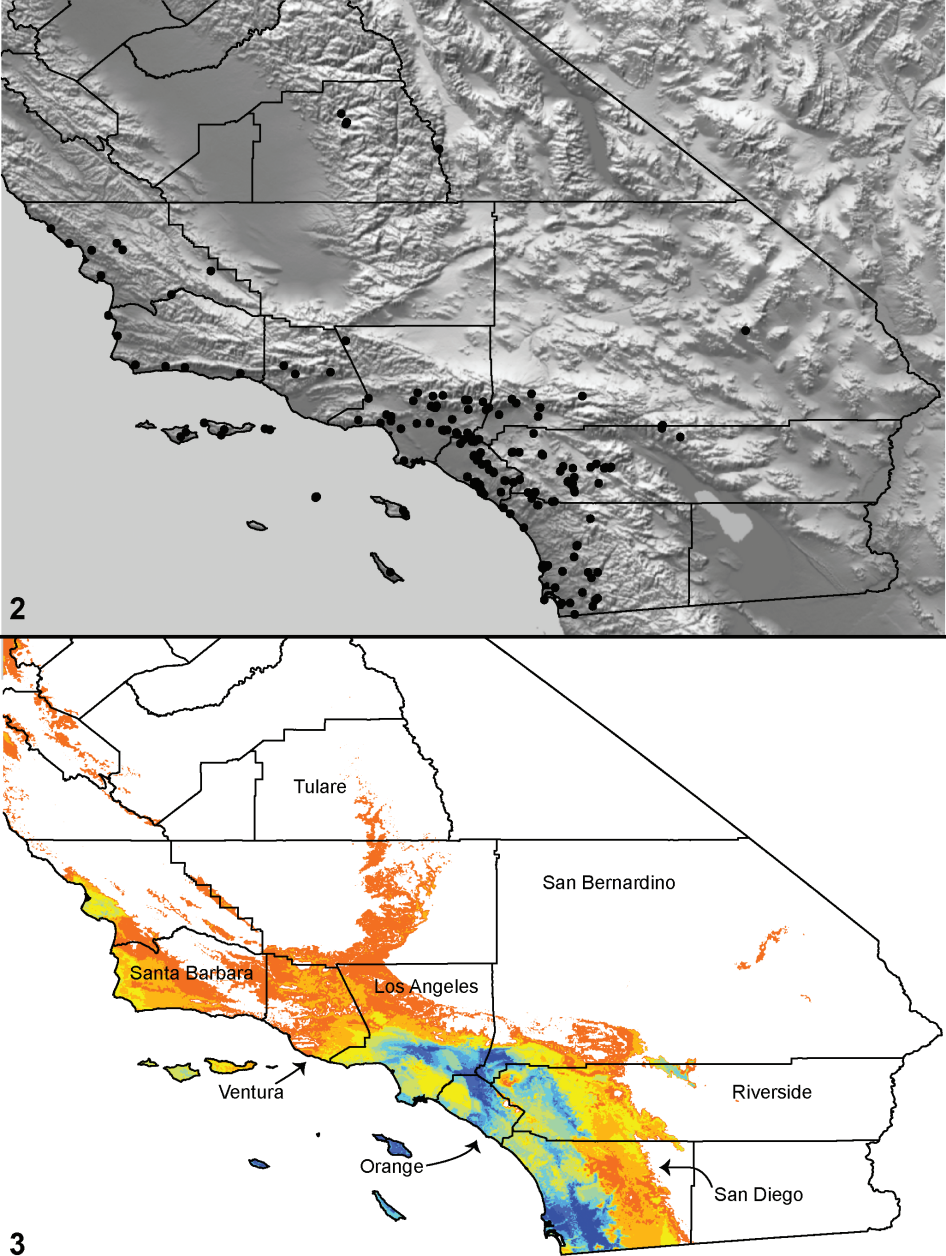
*Aptostichus atomarius* Simon, 1891. **2** distribution of known specimens **3** predicted distribution; cooler colors–blue shades–represent areas of high probability of occurrence, warmer colors–yellow and orange shades–represent areas of low probability of occurrence.

Figures 69 [Fig F16] –81 Map 2, 3

Aptostichus atomarius Simon, 1891: 317; female lectotype (AR4263) from California, San Bernardino; specimen in MNHP, examined–P. Smith 1908: 220–221.–Bond & Opell 2002: 518–519.
Actinoxia versicolor Simon, 1891: 318, synonymized by Bond & Opell 2002: 518.

#### Diagnosis.

**Figures 76–78. F16:**
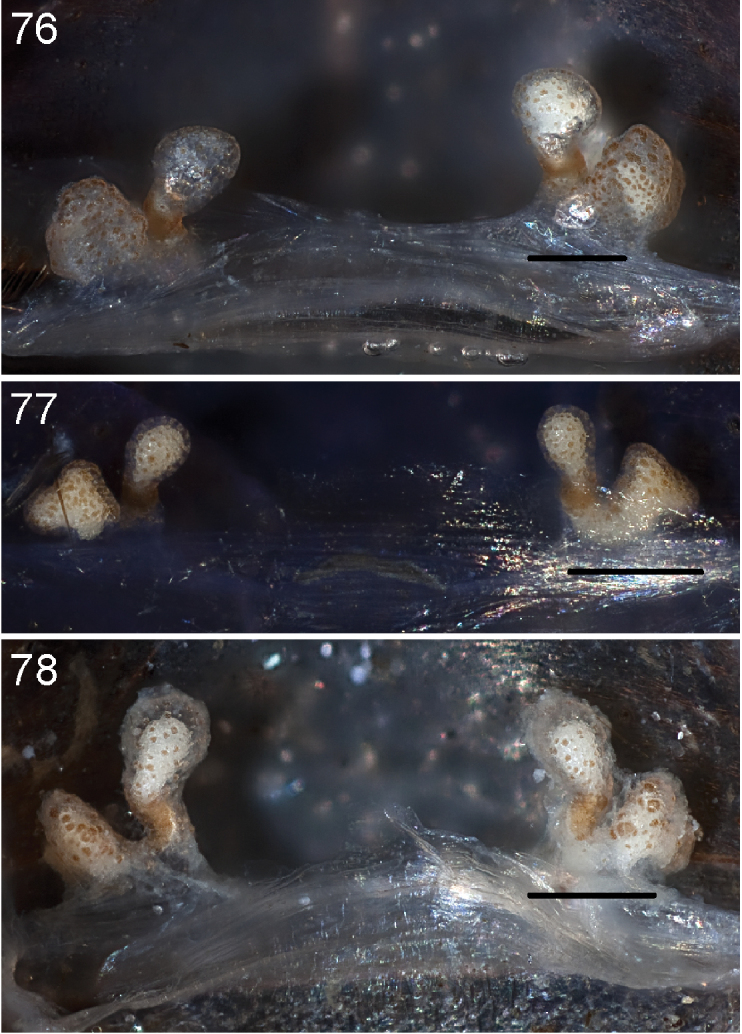
*Aptostichus atomarius* cleared spermathecae; scale bar = 0.25mm. **76** Los Angeles Co., San Gabriel Canyon (MY2618) [806561] **77** Riverside Co., Winchester (MY2603) [806564] **78** San Bernardino Co., San Bernardino National Forest (AP724) [806567].

**Figures 79–81.**
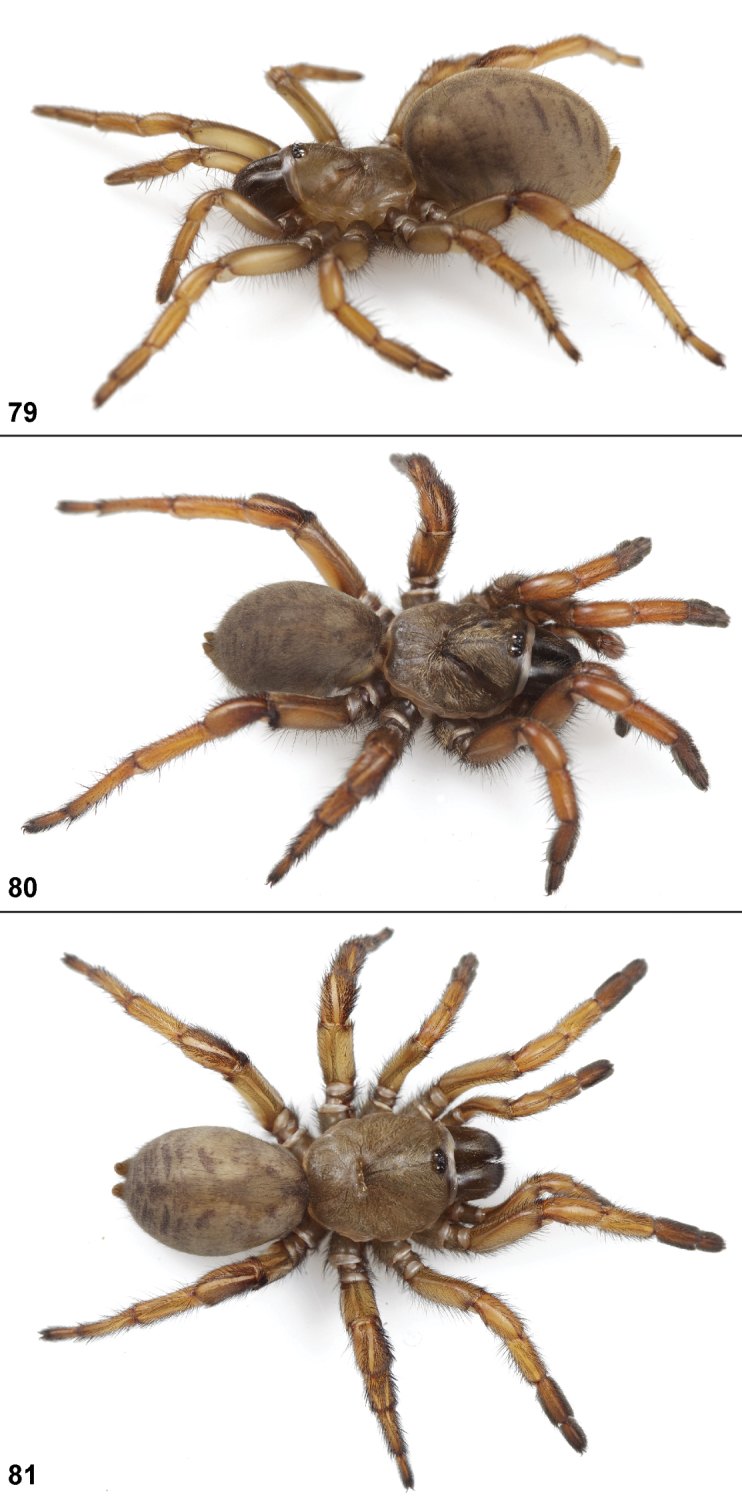
*Aptostichus atomarius* specimens, live photographs. **79** MY3794 **80** AUMS74 **81 **AUMS71.

Like all *Atomarius* Sibling Species Complex males, *Aptostichus atomarius* can be diagnosed by virtue of having a sharp triangular metatarsal mating apophysis and four or more TSrd spines arranged linearly without overlapping (Figs 69–73). Male pedipalp morphology relatively homogenous, comprising a slender palpal tibia that lacks a retrolateral spine patch (Fig. 74) and a simple unserrated bulb (Fig. 75). Females can be distinguished by having a secondary spermathecal bulb that extends below the horizontal plane of the lateral spermathecal base (Figs 76–78). Specimens in life have a mottled abdominal coloration pattern and tend to have carapace and legs with an orange tint (Figs 79–81) whereas other sympatric species (e.g., *Aptostichus icenoglei*) have darker leg and carapace coloration. *Aptostichus atomarius* females also tend to have a narrower sternum than *Aptostichus icenoglei*, however, this difference is very subtle and not quantifiable. Generally, individuals of this species are difficult to distinguish from other *Atomarius* Sibling Species Complex members on the basis of morphological features alone but can be diagnosed on the basis of a set of unique mtDNA site substitutions (see Bond and Stockman 2008). The distribution of *Aptostichus atomarius* is restricted to Southern California and does not overlap with other closely related sibling species (Map 2).

#### Descriptions.

Female originally described by Simon (1891); Male described and female redescribed by Bond and Opell (2002).

#### Material examined.

**United States: California: Inyo Co.:** Summit Creek, 8km S 4.8km W Olancha, 36.2109, -118.0676^5^, 1951m, D Giuliani 16.iv.1989 [AP169, 1♂, CAS]; **Los Angeles Co.:** ~3.2km SE of Tujunga, ~91m NW of intersection Lowell & Honolulu Ave, now site HWY 210, 34.230626, -118.270262^4^, 515m, W Icenogle 12.x.1972 [AP195, 1♀ 78juv, CAS; AP203, 1♀ 38 juv., AMNH]; Alamos CG (Hard Luck) Rd, lower Hungry Valley, 34.702, -118.8016^1^, 860m, M Hedin, J Satler, J Starrett, C Richart 16.ii.2009 [MY3767, 3769 1♀ 1juv, AUMNH]; Altadena, 34.1792, -118.1093^3^, 340m, M Thompson 1.xi.1969 [AP614, 1♂, AMNH]; Altadena, 34.1897, -118.1311^7^, 430m, 2.xii.1985 [AP1245, 1♂, CAS]; Angeles National Forest, San Gabriel Mtns, ~4.8km (road) W Mt Baldy Village, 70m S Glendora Ridge Rd S side Cow Canyon, 34.2237, -117.7239^3^, 1237m, W Icenogle 21.v.1974 [AP200, 1♀, CAS]; Baldwin Hills, 34.0114, -118.3686^4^, 125m, G Morris 1.xii.1944 [AP181, 1♀ 1♂, AMNH], 28.viii.1947 [AP490, 1♀, AMNH]; Baldwin Park, 34.0854, -117.9647^5^, 111m, R Crandall 18.x.1962 [AP001, 1♂, AMNH]; Chatsworth, intersection Lassen St & Valley Circle Blvd, ravine just E Oakwood Cemetery, 34.2512, -118.6227^4^, 315m, W Icenogle 28.viii.1966 [AP191, 1♀ 91juv, CAS], 13.x.1966 [AP477, 1♀, CAS], 23.x.1966 [AP183, 1♂, AMNH]; Claremont, Palmer Canyon Rd, 34.1593, -117.6996^6^, 817m, 9.ix.1923 [AP462, 1♀ 1juv, AMNH]; E Fork San Gabriel Canyon in Burro Canyon, 34.2366, -117.8392^3^, 432m, K Kinulan 6.x.1968, [AP664, 1♀, AMNH]; Eaton Canyon Park, 34.1783, -118.0951^4^, 298m, D Marqua 18.xi.1965 [AP157, 1♂, AMNH]; Henninger Flats, 34.1925, -118.0894^1^, 775m, J Bond, C Spruill, D Beamer 15.iv.2004, [MY2634, 1juv, AUMNH]; Henninger Flats, 34.19266, -118.0875^3^, 792m, D Marqua 18.xi.1967 [AP613, 1♂, AMNH]; Lady Bug Canyon, San Gabriel Mountains, 34.2685, -118.117^5^, 1225m, 1.iii.1971 [AP152, 1♂, AMNH]; Little Dalton Canyon, Glendora Mountain Rd, 34.1637, -117.8389^1^, 380m, J Bond, C Spruill, D Beamer 16.iii.2004 [MY2610, 2612, MY2616, 2juv, 1♀]; Los Angeles, 1220 Williams Rd, 34.0570, -118.1407^3^, 159m, D Lowrie 21.xi.1959 [AP168, 1♂, CAS]; Malibu Canyon, Malibu Canyon Rd 160m from intersection Piuma Rd, 34.0799, -118.7036^1^, 149m, J Bond, C Spruill, D Beamer 14.iv.2004 [MY2636, 1juv, AUMNH]; Mt Baldy Rd, ~0.2 km N jct N Mountain Ave, 34.1773, -117.6767^1^, 800m, M Hedin, J Satler, J Starrett, C Richart 15.ii.2009 [MY3764, 1juv, AUMNH]; Near Los Angeles, 34.0522, -118.2428^7^, 85m, G Jenko 11.viii.1939 [AP471, 1♀, AMNH]; Pacific Palisades, near Santa Monica, 34.0553, -118.5288^6^, 113m, G Morris 1.ii.1945 [AP308, 1♀, AMNH]; Pasadena, Eaton Canyon Park, 34.1807, -118.095^4^, 325m, B Crandall 31.xii.1963 [AP151, 1♂, AMNH], M Thompson 1.xi.1964 [AP153, 4♂, AMNH], 7.ii.1965 [AP161, 1♀ 2♂, CAS], 3.xi.1967 [AP165, 1♂, AMNH], 1.xii.1967 [AP174, 1♂, AMNH], 17.x.1969 [AP615, 1♂, AMNH], 10.xi.1969 [AP612, 1♂, AMNH]; Puente Hills, intersection Azusa & Tomich Rd, 33.9816, -117.9335^1^, 210m, J Bond, C Spruill, D Beamer 14.iii.2004 [MY2591, 1♀, AUMNH]; Puente Hills, near Diamond Bar, Pitfall array PUE-6, 33.9785, -117.8436^1^, 269m, R Fisher 1.xi.1998 [AP829, 2♂, CAS], 1.x.1999 [AP830, 1♂, CAS], 1.xi.2002 [AP831, 1♂, CAS]; Puente Hills, near Diamond Bar, Pitfall array PUE-7, 33.9802, -117.8443^1^, 271m, R. Fisher 1.xi.1998 [AP832, 1♂, CAS]; Puente Hills, north of Powder Canyon, 33.96812, -117.92347^1^, 273m, USGS-BRD San Diego Sta. 1.xi.1998 [AP933, 1♂, CAS]; Puente Hills, S Roland Heights, Pitfall array PUE-10, 33.969, -117.9268^1^, 294m, R Fisher 1.iv.1998 [AP833, 1♂, CAS], 1.x.1999 [AP834, 1♂, CAS]; Puente Hills, S Sycamore Canyon, 33.9985, -118.0339^1^, 268m, R Fisher 1.x.1999 [AP836, 3♂, CAS]; Puente Hills, S Sycamore Canyon, 33.998, -118.031^1^, 273m, R Fisher 1.x.1999 [AP837, 1♂, CAS]; Puente Hills, S Sycamore Canyon 2, 34.0001, -118.0424^1^, 196m, R Fisher 1.x.1999 [AP838, 2♂, CAS], 1.xi.2002 [AP839, 1♂, CAS]; Puente Hills, S Sycamore Canyon 4, 34.0001, -118.0291^1^, 673m, R Fisher 1.x.1999 [AP842, 1♂, CAS]; Puente Hills, Sycamore Canyon, 34.0023, -118.0372^1^, 161m, R Fisher 1.xi.1998 [AP840, 1♂, CAS], 1.x.1999 [AP841, 1♂, CAS]; Puente Hills, Sycamore Canyon, E Whittier, 34.0027, -118.0305^1^, 200m, USGS-BRD San Diego Sta. 1.x.1999 [AP934, 1♂, CAS]; Puente Hills, S Roland Heights 1, 33.9688, -117.9288^1^, 266m, R Fisher 1.x.1999 [AP835, 1♂, CAS]; Rincon Fire Station, San Gabriel Canyon, 34.2379, -117.8633^1^, 477m, J Bond, C Spruill, D Beamer 15.iii.2004 [MY2618, 1♀, AUMNH]; Rollings Hills, 33.7604, -118.3433^5^, 275m, J Buttram [AP1266, 1♀, AMNH]; San Clemente Island, E face Mount Thirst, 32.884, -118.451^5^, 584m, J Boyen 22.iii.1972 [AP176, 1♂, CAS]; San Gabriel Mountains, shortcut AC Hwy, 34.2737, -118.0385^6^, 1402m, M Thompson 26.iv.1964 [AP660, 1♀, AMNH]; San Gabriel Mtns, Angeles National Forest, Big Tujunga Canyon Rd, ~1.2 km W Vogel Flat Rd, 34.2949, -118.2365^1^, 600m, M Hedin, J Satler, J Starrett, C Richart 15.ii.2009 [MY3765, 1juv, AUMNH]; Santa Catalina Island, Avalon, 33.3444, -118.3334^5^, 66m, V Roth, [AP272, 1♂, AMNH]; Santa Monica Mnts., Beverly Glen Canyon, 34.1091, -118.4459^5^, 255m, F Hovore 11.xi.1965 [AP150, 1♂, AMNH]; Santa Monica Mnts, Sepulveda Pass, 34.1228, -118.4824^5^, 394m, F Hovore 27.xi.1969 [AP310, 1♀, AMNH]; Toyon Bay, Santa Catalina Island, 33.3707, -118.3496^4^, 1♂, M Ramirez 12.viii.1988 [AP470, 1♀, CAS]; UCLA, 34.0723, -118.448^3^, 117m, 1.xi.1952 [AP003, 1♂, AMNH]; **Orange Co.:** Bauer Peak, 33.8012, -117.7953^1^, 228m, USGS-BRD San Diego Sta. 1.xi.1999 [AP1052, 1053, 2♂, CAS]; Bauer Peak, 33.802, -117.7938^1^, 227m, USGS-BRD San Diego Field Sta. 1.xi.1998 [AP1051, 1♂, CAS]; Chino Hills, E Gilman Peak, 33.9282, -117.766^1^, 486m, USGS-BRD San Diego Field Sta. 1.i.2001 [AP1018, 1♂, CAS], 1.i.2002 [AP1019, 1♂, CAS]; Chino Hills, E Lookout Tower, 33.9393, -117.8169^1^, 396m, USGS-BRD San Diego Sta. 1.x.2001 [AP1047, 1♂, CAS], 1.iv.2002 [AP1048, 1♂, CAS]; Chino Hills, ridge south of Telegraph Canyon, 33.9116, -117.7888^1^, 355m, USGS-BRD San Diego Field Station 1.x.2000 [AP1038, 1♂, CAS]; Chino Hills, ridge S Telegraph Canyon, 33.9109, -117.7891^1^, 354m, USGS-BRD San Diego Field Sta. 1.x.2000 [AP1036, 1♂, CAS], 1.x.1998 [AP1034, 2♂, CAS], 1.x.1999 [AP1035, 1♂, CAS]; Chino Hills, ridge S Telegraph Canyon, 33.9116, -117.7888^1^, 355m, USGS-BRD San Diego Field Sta. 1.x.2001 [AP1037, 1♂, CAS]; Chino Hills, ridge S Telegraph Canyon, 33.9119, -117.7891^1^, 358m, USGS-BRD San Diego Field Sta. 1.x.2000 [AP1042, 1040 2♂, CAS], 1.x.1998 [AP1041, 1♂, CAS]; Chino Hills, Telegraph Canyon, 33.9133, -117.8109^1^, 179m, USGS-BRD San Diego Sta. 1.x.2001 [AP1045, 1046 1♂, CAS]; Chino Hills, ridge S Telegraph Canyon, 33.9112, -117.8018^1^, 255m, USGS-BRD San Diego Sta. 1.x.2000 [AP1044, 1♂, CAS], 1.x.1999 [AP1043, 1♂, CAS]; Crow Canyon, 33.6029, -117.5442^1^, 338m, USGS-BRD San Diego Sta. 1.i.2000 [AP1131, 1♂, CAS]; Dana Point, Salt Creek above golf course and bridge, 33.4971, -117.7145^1^, 58m, J. Bond 2.ii.2004 [MY2482, 1juv, CAS]; E El Modena, N Chapman Ave, 33.7936, -117.7942^1^, 176m, USGS-BRD San Diego Sta. 1.xi.1998 [AP957, 2♂, CAS]; El Modena, ridge S Bauer Peak, 33.7936, -117.7942^1^, 176m, USGS-BRD San Diego Sta. 1.xi.2001 [AP1049, 1♂, CAS]; El Modena, ridge S Bauer Peak, 33.7981, -117.7942^1^, 196m, USGS-BRD San Diego Sta. 1.xii.2000 [AP1050, 1♂, CAS]; Fullerton, E Coyote Hills, 33.8957, -117.9003^1^, 139m, USGS-BRD San Diego Sta. 1.xi.2001 [AP1107, 1♂, CAS], 1.xi.1999 [AP1109, 1♂, CAS], 1.x.2001 [AP1108, 1♂, CAS]; Intersection H74 and Trampas Canyon overpass, 33.5136, -117.5823^1^, 89m, J Bond, C Spruill, D Beamer 18.iii.2004 [MY2673, 2675 2♀, CAS]; Loma Ridge, W Limestone Canyon, 33.7363, -117.6901^1^, 417m, USGS-BRD San Diego Sta. 1.xi.1998 [AP1058, 1♂, CAS], 1.xii.1999 [AP1059, 1♂, CAS]; N Aliso Wood Canyon, 33.5575, -117.7464^1^, 64m, USGS-BRD San Diego Field Station 1.x.2002 [AP950, 1♂, CAS]; N Aliso Wood Canyon, 33.5675, -117.7482^1^, 80m, USGS-BRD San Diego Field Station 1.xi.2002 [AP951, 1♂, CAS]; NE Brea, S Oil Field, 33.9272, -117.8742^1^, 174m, R Fisher 1.xi.1998, [AP826, 1♂, CAS], 1.ii.1999 [AP827, 1♂, CAS], 1.x.1999 [AP828, 1♂, CAS]; NE Lemon Heights Lomas de Santiago E Peters Canyon, 33.7643, -117.7667^1^, 284m, USGS-BRD San Diego Sta. 1.xii.2000 [AP958, 1♂, CAS]; NE Lemon Heights, Peters Canyon, 33.7631, -117.77^1^, 272m, USGS-BRD San Diego Sta. 1.xii.2000 [AP956, 1♂, CAS]; NE Rattlesnake Reservoir, 33.7313, -117.731^1^, 143m, USGS-BRD San Diego Sta. 1.xi.2000 [AP888, 1♂, CAS], 1.vi.2000 [AP889, 1♂, CAS]; NW upper Oso Reservoir, N El Toro Rd, 33.6725, -117.6415^1^, 260m, USGS-BRD San Diego Sta. 1.xi.2001 [AP1116, 1♂, CAS]; NW Upper Oso Reservoir, N El Toro Rd, 33.6702, -117.6396^1^, 283m, USGS-BRD San Diego Field Station 1.i.2000 [AP1228, 1♀, CAS]; Ridge E Bell Canyon, 33.6041, -117.5525^1^, 518m, USGS-BRD San Diego Sta. 1.i.2000 [AP1138, 1♂, CAS]; San Joaquin Hill, ridge N of E Morro Canyon, 33.59, -117.7931^1^, 249m, USGS-BRD San Diego Sta. 1.xi.2002 [AP1112, 1♂, CAS]; San Joaquin hills, E branch Laguna Canyon, 33.5946, -117.7752^1^, 175m, USGS-BRD San Diego Sta. 1.x.2000 [AP1175, 1♂, CAS]; S University of Irvine Campus, N Bonita Canyon, E Spring Canyon, 33.6334, -117.8474^1^, 66m, USGS-BRD San Diego Sta. 1.xi.1998 [AP1180, 1♂, CAS]; Weir Canyon, 33.8148, -117.7462^1^, 252m, USGS-BRD San Diego Field Sta. 1.xii.2000 [AP1122, 1♂, CAS]; Weir Canyon, 33.8155, -117.7471^1^, 232m, USGS-BRD San Diego Field Sta. 1.x.1999 [AP1123, 1♂, CAS]; Weir Canyon, 33.8184, -117.7446^1^, 280m, USGS-BRD San Diego Field Sta. 1.xii.2000 [AP1125, 1♂, CAS], 1.x.2001 [AP1126, 1♂, CAS]; Weir Canyon, 33.8252, -117.7419^1^, 249m, USGS-BRD San Diego Field Sta. 1.i.2000 [AP1127, 1♂, CAS]; E Cowan Heights, Peters Canyon, 33.7741, -117.7616^1^, 260m, USGS-BRD San Diego Sta. 1.xi.2001 [AP995, 1♂, CAS], 1.x.1998 [AP961, 962 4♂, CAS]; E El Toro Marine Corp Air Sta. N Borrego Canyon, 33.6896, -117.6741^1^, 266m, USGS-BRD San Diego Sta. 1.i.2003 [AP969, 1♂, CAS]; Niguel Hill, E of S Aliso Wood Canyon, 33.5123, -117.7397^2^, 195m, USGS-BRD San Diego Sta. 1.ii.2000 [AP948, 1♂, CAS], 1.ii.2002 [AP949, 1♂, CAS]; N Aliso Wood Canyon, 33.574, -117.7493^1^, 104m, USGS-BRD San Diego Sta. 1.ii.2001 [AP952, 1♂, CAS]; NE Lemon Heights, Lomas de Santiago, E Peters Canyon, 33.7672, -117.7637^1^, 295m, USGS-BRD San Diego Sta. 1.viii.1999 [AP959, 1♂, CAS]; San Joaquin Hills, SW Laguna Beach Rd & El Toro Rd jct, 33.5738, -117.771^1^, 243m, USGS-BRD San Diego Sta. 1.x.2000 [AP999, 1♂, CAS], 1.xi.2002 [AP998, 1♂, CAS]; S Aliso Wood Canyon, 33.5185, -117.7386^1^, 7m, USGS-BRD San Diego Sta. 1.ii.2000 [AP974, 3♂, CAS]; S Aliso Wood Canyon, 33.5327, -117.7414^1^, 20m, USGS-BRD San Diego Sta. 1.x.2001 [AP978, 1♂, CAS]; S Aliso Wood Canyon, 33.5333, -117.0066^1^, 26m, USGS-BRD San Diego Sta. 1.x.2002 [AP947, 1♂, CAS]; S Alliso Wood Canyon, 33.5264, -117.7358, 30m, USGS-BRD San Diego Sta. 1.ii.2001 [AP976, 1♂, CAS], 1.ii.2002 [AP977, 1♂, CAS]; Temple Hill, W of N Aliso Wood Canyon, 33.005, -117.0066^1^, 295m, USGS-BRD San Diego Sta. 1.ii.2000 [AP953, 1♂, CAS]; 0.4km S hwy 74, 4km E San Juan Fire Station, Hot Spring Canyon turnoff, 33.5902, -117.4755^1^, 373m, W Icenogle 3.xi.2000 [MY3785, 1♂, AUMNH], 6.ii.2001 [MY3786, 1♂, AUMNH], 8.xi.2001 [MY3787, 1♂, AUMNH], 30.xii.2005 [MY3788, 1♂, AUMNH]; **Riverside Co.:** ~300m W of jct hwys 243 & 74 on hwy 74 W Mt. Center, 33.7047, -116.7256^1^, 1341m, M Hedin, C Johnson, R Keith 2.ii.2003 [MY0722, 1juv, AUMNH]; 3.0km (road) W Lake Mathews, canyon just S Cajalco Rd, 33.8256, -117.4957^3^, 265m, W Icenogle 14.xii.1998 [AP371, 1♂, CAS], 2.i.1999 [AP370, 1♂, CAS], 7.ii.1999 [AP661, 2♂, CAS], 4.iv.1999 [AP382, 1♂, CAS], 13.iv.1999 [MY3784, 1♂, CAS], 4.v.1999 [AP369, 1♂, CAS], 31.v.1999 [AP354, 1♀, AUMNH], 8.xii.2001 [MY3782, 4♂, CAS], 2.xii.2000 [MY3783, 1♂, CAS], 4.8km W Mountain Center on HWY 74, 33.7011, -116.769^3^, 1124m, W Icenogle 26.viii.1968 [AP184, 1♂, CAS; AP190, 451, 456, 2♀ 41juv, AMNH]; 13km NW Winchester on N facing hillside and base of vegetation, 33.5747, -117.0302^1^, 495m, J Bond 13.xii.1997 [AP677, 1♀, AUMNH]; Bautista Canyon, along Bautista Canyon Road, 33.7099, -116.8775^1^, 611m, J Bond 1.ii.2004 [MY2477, 1♀, AUMNH]; canyon adjacent to Cajalco Canyon along Cajalco Exp, 33.828, -117.4871^1^, 335m, J Bond, W Icenogle 29.i.2004 [MY2490, MY2517, 2 juv., AUMNH], 29.i.2004, [MY2491, 1♀, AUMNH]; E Rawson Canyon, S Crown Valley, 33.6299, -117.0052^1^, 661m, USGS-BRD San Diego Field Sta. 1.ii.2000 [AP1118, 1♂, CAS]; E Skinner Reservoir, 33.5869, -117.0212^1^, 501m, USGS-BRD San Diego Sta. 1.ii.2000 [AP1009, 1012, 2♂, CAS]; E Skinner Reservoir, 33.5819, -117.0189^1^, 488m, USGS-BRD San Diego Field Sta. 1.iv.1999 [AP1211, 2♂, CAS], 1.ii.2000, [AP1212, 1♂, CAS]; E Skinner Reservoir, 33.5827, -117.0211^1^, 479m, USGS-BRD San Diego Field Sta. 1.ii.2000 [AP1007, 1♂, CAS]; E Skinner Reservoir, 33.5977, -117.024^1^, 464m, USGS-BRD San Diego Field Sta. 1.ii.2000 [AP1027, 1♂, CAS]; E Skinner Reservoir, 33.5989, -117.0235^1^, 467m, USGS-BRD San Diego Field Sta. 1.ii.2000 [AP1026, 1♂, CAS]; hills N Diamond Valley, 33.7024, -117.0164^1^, 553m, USGS-BRD San Diego Sta. 1.x.1998 [AP1110, 2♂, CAS], 1.iii.1999, [AP1111, 1♀, CAS]; Joshua Tree National Park, Upper Covington Flat, 34.0145, -116.3159^1^, 949m, USGS-BRD San Diego Sta. 1.iii.2000 [AP900, 1♂, CAS]; Joshua Tree National Park, Covington Flat, 34.0311, -116.3177^1^, 1554m, J Bond 8.xii.1997 [AP678, AP1262, 2♀, CAS]; Joshua Tree National park, S Cap Rock, 33.9476, -116.1716^1^, 1412m, USGS-BRD San Diego Sta. 1.iv.2000 [AP931, 1♂, CAS]; just S Winchester on Leona Rd, ~1.6km S intersection with Patton Avenue, 33.6771, -117.1157^1^, 444m, J Bond 1.ii.2004, [MY2511, 2518, 2514, 2524, 4♀, AUMNH]; Lake Matthews, 33.8266, -117.438^1^, 446m, J Bond 22.xi.1998 [AP685, 686, 688, 2♀ 1♂, AUMNH]; Lake Skinner Rec Area, 0.8km N Lake, 33.599, -117.0539^3^, 488m, T Prentice 16.xii.1996 [AP242, 1♂, CAS]; Lake Skinner, 13km NW Winchester, 33.595, -117.058^3^, 485m, [AP504, 1♂, CAS]; Mountain Center, San Jacinto Mountains, 33.7042, -116.725^3^, 1375m, W Icenogle 26.viii.1968 [AP015, 1♀, AMNH], 9.ii.1969 [AP020, 1♀, AMNH]; Mountain Center, small ravine on H243, ~1.6km from junction H74, 33.712, -116.7164^1^, 1440m, J Bond 31.i.2004 [MY2478, 1juv, AUMNH]; N Perris, E Mayer Farms, 33.8045, -117.2518^1^, 571, USGS-BRD San Diego Sta. 1.i.2000 [AP1100, 1♂, CAS]; N Perris, E Mayer Farms, 33.8109, -117.2578^1^, 593m, USGS-BRD San Diego Field Sta. 1.ii.1999 [AP1103, 1♂, CAS]; N Perris, E Mayer Farms, 33.8145, -117.2601^1^, 575m, USGS-BRD San Diego Field Sta. 1.ii.1999 [AP1106, 1♂, CAS]; N Tenja Canyon, S Wildomar Rd., 33.5104, -117.3687^1^, 604m, USGS-BRD San Diego Sta. 1.i.2000 [AP874, 1♂, CAS]; Ortega HWY H74, ~2.7km N Orange Co./Riverside Co. line, 33.6127, -117.4346^1^, 523m, J Bond 2.ii.2004 [MY2487, 2493 2juv, AUMNH]; San Bernardino National Forest, 33.7363, -116.8316^1^, 603m, J Bond 28.iii.1996 [AP724, 1♀, AUMNH]; San Bernardino National Forest, 1.1km W Mt Center, 33.7083, -116.7604^1^, 964m, J Bond 31.i.2004 [MY2472, 1juv, AUMNH]; San Bernardino National Forest, Juan Diego Flats Rd, ~1km from intersection w/Reed Valley Rd, 33.5836, -116.8141^1^, 1110m, J Bond 7.iv.1996 [AP745, 1♀, AUMNH]; Santa Margarita Ecological Reserve, S Santa Margarita River, NW Royal Oak Ranch, 33.4388, -117.1798^1^, 256m, USGS-BRD San Diego Sta. 1.ii.2000 [AP897, 1♂, CAS]; S Santa Margarita River, N Royal Oak Ranch, 33.4399, -117.1605^1^, 421m, USGS-BRD San Diego Sta. 1.ii.2000 [AP893, 2♂, CAS]; University of California, Riverside Campus, 33.9742, -117.3251^3^, 327m, W Icenogle 17.xii.1969 [AP350, 1♂, AMNH]; W De Luz Creek, S Tenaja Rd, 33.5139, -117.0016^1^, 688m, USGS-BRD San Diego Field Sta. 1.i.2001 [AP982, 1♂, CAS]; Winchester, 1.6km NW of town center, Grand Ave, vicinity of Double Butte, 33.7149, -117.0922^1^, 478m, W Icenogle 17.xi.1968 [AP248, 1♂, AMNH]; Winchester, intersection Leon Rd & Ano Crest Dr, 33.6777, -117.1159^1^, 445m, J Bond 18.i.1997, [AP1221, 1♀, AUMNH]; Winchester, just E Icenogle residence, 33.7156, -117.0936^1^, 465m, J Bond 29.i.2004 [MY2471, 1♀, AMNH]; Winchester, Leona Rd ~1.6km S intersection w/Patton Ave, 33.6771, -117.1157^1^, 444m, J. Bond, W. Icenogle, et al. 13.iii.2004 [MY2595, 2601-03, 2695-08, 8♀ 1juv, AUMNH]; between Squaw Mountain & Redonda Mesa, N Tenaja Rd, 33.5025, -117.3386^1^, 1020m, USGS-BRD San Diego Sta. 1.ii.2000 [AP1196, 1♂, CAS]; SE Skinner Reservoir, 33.5766, -117.0316^1^, 482m, USGS-BRD San Diego Sta. 1.ii.2000 [AP1223, 2♂, CAS]; W De Luz Creek, S Tenaja Rd, 33.5097, -117.3139^1^, 650m, USGS-BRD San Diego Sta. 1.i.2000 [AP992-94, 3♂, CAS]; W De Luz Creek, S Tenaja Rd, 33.511, -117.3147^1^, 674m, USGS-BRD San Diego Sta. 1.i.2000 [AP984, 986, 3♂, CAS], 1.xii.1999, [AP983, 1♂, CAS]; W De Luz Creek, S Tenaja Rd., 33.515, -117.3126^1^, 664m, USGS-BRD San Diego Sta. 1.i.2000 [AP987, 1♂, CAS]; **San Bernardino Co.:** Alta Loma, 34.122, -117.597^3^, 412m, D Bixler 18.iii.1969 [AP005, 3♂, AMNH], 20.iii.1969 [AP004, 1♂, AMNH], 20.iv.1969 [AP495, 1♂, AMNH]; Fawnskin, 34.2681, -116.9417^3^, 2077m, L Underwood 1.ix.1996 [AP497, 1♂, CAS]; Granite Mtns Preserve, 34.7844, -115.6579^1^, 1317m, R Vetter, J Bond 15.xii.1997 [AP673, 1♂, AUMNH]; Hwy 18, base San Bernardino Mnts, National Forest Boundary, 34.1776, -117.2752^1^, 516m, J Bond, W Icenogle 28.i.2004 [MY2466, 2497, 2498, 1♀ 2 juv., AUMNH]; Joshua Tree National Park, Lower Covington Flat, 34.0401, -116.3102^3^, 1433m, E Schlinger [AP529, 1♂, AMNH], E Sleeper, S Jenkins 10.iii.1965 [AP537, 1♂, CAS]; Lytle Creek Rd, ~4.5km N of jct w/ I-15, 34.2138, -117.4613^1^, 750m, M Hedin, J Satler, J Starrett, C Richart 15.ii.2009 [MY3762, 1♀, AUMNH]; Lytle Creek Rd, near Scotland, 34.244, -117.4952^1^, 950m, M Hedin, J Satler, J Starrett, C Richart 15.ii.2009 [MY3766, 1j, AUMNH]; San Bernardino, 1km S jct HWY 18 & Waterman Canyon Rd, 34.1774, -117.2736^1^, 488m, W Icenogle 16.i.2000-16.ii.2000 [AP351, 356, 3♂, AMNH; AP355, 357, 361, 362, 365, 5♂, CAS]; Silverwood Lake, 34.2871, -117.3408^1^, 1018m, USGS-BRD San Diego Sta. 1.x.2001 [AP1169, 1♂, CAS]; Chino Hills, E Gilman Peak, 33.93, -117.7543^1^, 486m, USGS-BRD San Diego Sta. 1.i.2002 [AP1017, 1♂, CAS]; **San Diego Co.:** Camp Pendleton, 33.2341, -117.4013^5^, 2m T Prentice 1.xii.1995 [AP579, 580 2♂, UCR]; Camp Pendleton Marine Corps Base, SW Horno Hill, between Foley & Horno Canyons, 33.3448, -117.5087^1^, 87m, R Fisher 26.i.2004 [AP823, 1♂, CAS]; Camp Pendleton, mouth San Onofre Creek, 33.3898, -117.5626^1^, 35m, USGS-BRD San Diego Sta. 1.xii.1999 [AP1159, 1♂, CAS]; Carmel Mountain, 32.9283, -117.2227^1^, 118m, USGS-BRD San Diego Field Sta. 1.x.2002 [AP940, 1♂, CAS]; Carmel Mountain, 32.9383, -117.2136^2^, 111m, USGS-BRD San Diego Field Sta. 1.xi.2002 [AP942, 1♂, CAS]; Chula Vista, Terra Nova, N Rice Canyon, 32.6432, -117.0377^2^, 105m, USGS-BRD San Diego Field Sta. 1.ii.2000 [AP1227, 1♂, CAS]; DeLuz Road, 8km N Fallbrook at DeLuz Rd./S13 jct, 33.411, -117.2898^1^, 229m, M Hedin 31.viii.2003 [MY2273, 2275 2♀ 4juv, AUMNH]; E Lakeside between El Monte Park & entrance to El Capitan Res, El Monte Rd, 32.8836, -116.8223^1^, 180m, M Hedin 23.ii.2008 [MY3632-34, 3637 3♀ 1juv, AUMNH]; Escondido Wild Animal Park, 33.0932, -116.9849^1^, 191, USGS-BRD San Diego Field Sta. 1.xi.1998 [AP1151, 1♂, CAS]; Escondido Wild Animal Park, 33.0961, -116.98^1^, 242m, USGS-BRD San Diego Field Sta. 1.i.2000 [AP1153, 1♂, CAS]; Escondido, San Diego Wild Animal Park, 33.0898, -116.9866^1^, 201, USGS-BRD San Diego Sta. 1.ii.2001 [AP1016, 1♂, CAS]; Escondido, Wild Animal Park, 33.0932, -116.9849^1^, 191m, USGS-BRD San Diego Field Sta. 1.ii.2001 [AP1150, 1♂, CAS]; Jamul Hollenbeck Canyon, E HWY 94, 32.6799, -116.8211^1^, 245m, USGS-BRD San Diego Field Sta. 7.xi.2003 [MY1192, 1♂, CAS]; Jamul, S Sweetwater River & Steele Canyon jct, 32.7252, -116.9416^1^, 129m, USGS-BRD San Diego Sta. 1.i.2001 [AP905, 1♂, CAS]; Jamul, S Sweetwater River & Steele Canyon jct, 32.7255, -116.9408^1^, 118m, USGS-BRD San Diego Sta. 1.i.2000, [AP902, 903, 2♂, CAS]; Little Cedar Canyon, SE Otay Mountain, 32.6164, -116.8604^1^, 443m, USGS-BRD San Diego Sta. 1.ii.1999 [AP884, 1♂, CAS]; Mission Valley, end of Hewlett Dr, 32.7625, -117.1565^5^, 64m, S Johnson 25.iii.1971 [AP002, 2♂, AMNH]; NW upper Oso Reservoir, N El Toro Rd, 33.6653, -117.6384^1^, 272m, USGS-BRD San Diego Field Sta. 1.i.2000 [AP1114, 1♂, CAS]; Otay Mesa, S HWY 905, 32.553, -117.0025^1^, 134m, USGS-BRD San Diego Sta. 1.ii.2000 [AP1177, 1♂, CAS]; Palomar Mountain area Cleveland National Forest, State Rd S6, ~4.8km (road) SW intersection S6 & S7, 33.3048, -116.8786^4^, 1275m, W Icenogle 15.vii.1970 [AP453, 1♀, AMNH]; Point Loma, Cabrillo National Monument, 32.667, -117.24^3^, 89m, E Jones 8.iii.2002 [AP1243, 1♂, CAS]; S University of Irvine Campus, N Bonita Canyon, E Spring Canyon, 33.6357, -117.8459^1^, 84m, USGS-BRD San Diego Field Sta. 1.ii.2000 [AP1179, 2♂, CAS],1.xi.1998 [AP1178, 1♂, CAS]; Tenaja Rd, 2mi N Deluz, 33.4642, -117.3395^5^, 198m, W Icenogle 19.ix.1968 [AP260, 1♀, 35juv, CAS]; Torrey Pines State Reserve, E beach, W North Torrey Pines Rd 2, 32.921, -117.2574^1^, 54m, R Fisher 1.ii.2000 [AP863, 1♂, CAS]; Torrey Pines State Reserve, E beach, W North Torrey Pines Road 5, 32.9238, -117.256^1^, 70m, R Fisher 1.xi.1998 [AP866, 2♂, CAS]; Torrey Pines State Reserve, S Del Mar Heights School 10, 32.941, -117.2529^1^, 73m, R Fisher 1.ii.2001 [AP857, 1♂, AUMNH], 1.xi.1998 [AP855, 1♂, CAS], 1.xii.1999 [AP856, 1♂, CAS]; Torrey Pines State Reserve, W of N Torrey Pines Rd, 32.9258, -117.2505^1^, 11m, USGS-BRD San Diego Sta. 1.ii.2000, [AP1194, 1♂, CAS]; Torrey Pines State Reserve, W of N Torrey Pines Rd, 32.9252, -117.2525^1^, 23m, USGS-BRD San Diego Field Sta. 1.xii.1999 [AP928, 1♂, CAS]; University of California Elliot Reserve, S Carroll Canyon, 32.8917, -117.0971^1^, 194m, USGS-BRD San Diego Sta. 1.iv.2004 [AP879, 1♂, CAS]; vic. Chula Vista, 32.6405, -117.1041^3^, 5m, R Fisher 21.ii.2000 [AP1249, 1♂, CAS]; Wildcat Canyon, 32.8831, -116.8967^5^, 219m, 6.i.1962 [AP496, 1♂, AMNH]; Camp Pendleton Marine Corps Base, E DeLuz Creek Rd, SW Sandia Canyon, Pitfall array CAM-23, 33.4112, -117.295^1^, 337m, R Fisher [AP824, 1♂, CAS]; Carmel Mountain, 32.9366, -117.21508^1^, 111m, USGS-BRD San Diego Sta. 1.xi.2002-1.xii.2002 [AP944-46, 3♂, CAS]; Flinn Springs, Rios Canyon, 32.8409, -116.8699^1^, 295m, USGS-BRD San Diego Sta. 1.i.2001 [AP971, 1♂ 1juv, CAS]; Jamul, Hollenbeck Canyon, E HWY 94, 32.6799, -116.8211^1^, 245m, USGS-BRD San Diego Sta. 27.v.2004 [AP1000, 1♀, CAS]; Jamul, Jamul Ecological Reserve, W HWY 94, near Dluzura Creek, 32.6665, -116.84^1^, 288m, USGS-BRD San Diego Sta. 1.iii.2002 [AP972, 1♂, CAS]; Torrey Pines State Reserve, E of N Torrey Pines Rd, mouth Soledad Valley, 32.927, -117.2562^1^, 16m, R Fisher 1.xi.1998 [AP867, 2♂, CAS]; Torrey Pines State Reserve, S Del Mar Heights School 3, 32.9398, -117.2518^1^, 62m, R Fisher 1.ii.2001 [AP850, 1♂, CAS]; Torrey Pines State Reserve, W of N Torrey Pines Rd, 32.9148, -117.25^1^, 98m, R Fisher 1.ii.2000 [AP844, 1♂, CAS]; **San Luis Obispo Co.:** 8km N Santa Margarita, 35.4694, -120.6017^6^, 400m, D Marqua 26.xi.1970 [AP172, 1♂, AMNH]; Corrizo Plain, Soda Lake, 21km SE Simmler, 35.2509, -119.8632^5^, 625m, D Giuliani 11.iii.1987 [AP570, 1♂, CAS]; Hwy 1 S jct Villa Creek Rd, 35.4682, -120.9758^5^, 12m, W Icenogle 6.x.1970 [AP202, 3juv, AMNH]; Hwy 58, 4.1mi E Santa Margarita, 35.4169, -120.5572^1^, 366m, M Hedin, P Paquin, J Starrett 5.iv.2003 [MY741, 745, 746, AP1200 2♀ 2juv, AUMNH], 11.x.2003, [MY2462, 1♀, CAS]; Morro Creek, off hwy 41, 14.5km W HWY 101, 35.4133, -120.8009^2^, 85m, D Palmer, J Starrett 26.vi.2003 [MY2270, 2274, 1♀ 1juv, AUMNH]; See Canyon Rd, 2.9km N San Luis Bay Dr, 35.216, -120.7264^1^, 65m, A Stockman 9.vii.2009 [MY3707, 3708, 2♀, AUMNH]; ~320m S jct Old Moonstone Beach Dr & HWY 1 on inland rd cut, 35.5836, -121.1206^1^, 16m, J Bond 6.xii.2005 [MY3444-46, 2juv, 1♂, AUMNH]; San Simeon Creek Rd off Hwy 1, N Cambria, 35.6091, -121.0837^1^, 40m, M Hedin, J Starrett, D Leavitt, D Carlson, B Keith 26.iii.2011 [MY3789, 1♀, AUMNH]; Toro Creek Rd off of Hwy 41, W of Atascadero, 35.4605, -120.7660^1^, 350m, M Hedin, J Starrett, D Leavitt, D Carlson, B Keith 26.iii.2011 [MY3794, 4005, 4014, 3♀, AUMNH]; Upper Lopez Canyon Rd, E of Lopez Lake, 35.1896, -120.4451^1^, 270m, M Hedin, J Starrett, D Leavitt, D Carlson, B Keith 25.iii.2011 [MY4015, 1♀, AUMNH]; La Panza CG, E La Panza, E Pozo Rd, 35.3531, -120.2633^1^, 420m, M Hedin, L Hedin, J Satler, D Carlson 25.vii.2010 [MY3810, 1juv, AUMNH]; **Santa Barbara Co.:** Cave Canyon, Santa Barbara Island, 33.4777, -119.0305^4^, 63m, M Ramirez 8.vii.1987 [AP439, 467, 480, 3♀, CAS]; Hot Springs, 34.503, -120.2186^5^, 206m, F.J.M. 29.v.1969 [AP262, 1♀, AMNH]; Montecito, E Mountain Dr 1.4km from San Ysidro Rd jct, 34.4463, -119.6303^1^, 118m, D Palmer, J Starrett 23.vi.2003 [MY2268, 1♀, AUMNH]; Point Sal, 34.9031, -120.6694^6^, 52m, W Icenogle 12.viii.1978 [AP249, 3juv, AMNH]; Refugio Rd, 2.7km N HWY 101, 34.4927, -120.0658^1^, 86m, A Stockman, A Bailey 9.vii.2009 [MY3711, 1♀, AUMNH]; Santa Barbara Island, Middle Canyon, 33.4756, -119.0353^7^, 106m, M Ramirez 8.vii.1987 [AP192, 1♀, CAS]; Santa Barbara Island, near jct of all points, Arch, Signal Peak, & Elephant Seal Cove trails, 33.4758, -119.0372^5^, 137m, M Ramirez 28.iii.1982 [AP298, 1♀, AMNH]; Santa Cruz Island, 33.9617, -119.7812^6^, 3m, 12.ix.1982 [AP265, 1♀, CAS]; Santa Cruz Island, Central Valley, 34.0061, -119.7567^6^, 230m, 10.ix.1982 [AP294, 1juv, AMNH]; Santa Cruz Island, Forney’s Cove, 34.0583, -119.915^3^, 15m, E Sleeper 15.iii.1969 [AP446, 1juv, AMNH]; Santa Rosa Island, 33.95, -120.1^6^, 445m, M Thompson 13.ix.1974 [AP312, 2♀ 1juv, AMNH]; Santa Rosa Island Upper Torrey Pine area, 33.9839, -120.0599^5^, 190m, M Ramirez, H David 10.viii.1994 [AP507, 1♀, CAS]; Sierra Madre Mountains, along HWY 166, 35.0663, -120.1738^1^, 340m, J Bond, M van der Merwe 18.xii.1999 [MY0714, 1♀, AUMNH]; Signal Peak, W slope Santa Barbara Island, 33.4717, -119.0398^4^, 174m, C Drost 24.vii.1981 [AP282, 1juv. CAS]; Vandenberg Air Force Base, Plot #6, 34.7422, -120.5974^4^, 83m, 19.xi.2004 [MY2978, 2980, 2♂, CAS]; Jalama Rd, 7.5km E coast line, 34.5145, -120.4551^1^, 83m, J Bond, A Stockman, D Beamer 17.iii.2005 [MY3417, 1♀, AUMNH; MY3419, 1♀, AMNH]; **Tulare Co.:** Kaweah Power Station #3, 36.488, -118.837^6^, 460m, D Burdick 4.vi.1983 [AP006, 1♂, CAS]; N base Case Mountain, 36.4218, -118.7937^1^, 1646m, USGS-BRD San Diego Sta. 1.v.2002 [AP1166, 1♂, CAS]; SE side Case Mountain, 36.407, -118.8016^1^, 1998m, USGS-BRD San Diego Sta. 1.ix.2003 [AP1176, 2♂, CAS]; **Ventura Co.:** Anacapa Island, 34.0044, -119.3986^5^, 72m, M Thompson 2.iv.1968 [AP500, 1♀ 10juv, AMNH]; Hwy 150, W Ojai, N Dennison Park, 34.4407, -119.1976^1^, 341m, A Stockman, A Bailey 11.vii.2009 [MY3726, 1♀, AUMNH]; Los Padres National Forest, 8.8km S Squaw Flats, 34.4557, -118.922^6^, 477m, Marqua 25.x.1970 [AP269, 1♀, AMNH]; Los Padres NF, Hwy 33, mile marker 17.92, N Ojai, 34.5071, -119.289^1^, 75m, A Stockman, A Bailey 9.vii.2009 [MY3714, 1♀, AUMNH]; W Anacapa Island, 34.012, -119.4373^4^, 114m, M Ramirez 7.vii.1987, [AP440, 1♀, CAS]; Lockwood Valley Rd, 8km E int w/Hwy 33, N Wheeler Springs, 34.6895, -119.2800^1^, 1217m, J Satler, J Grummer, J Nash 2.iv.2010 [MY3818, 1juv, AUMNH].

**Variation, males (5).** Cl 5.31-5.94, 5.70±0.11; Cw 4.25-5.13, 4.78±0.15; STRl 3.09-3.48, 3.24±0.07; STRw 1.60-2.94, 2.50±0.25; LBw 0.68-0.98, 0.81±0.05; LBl 0.30-0.53, 0.40±0.04; leg I: 5.19-6.00, 5.49±0.15; 3.44-4.25, 3.8±0.14; 3.38-4.25, 3.72±0.16; 2.10-2.73, 2.45±0.13; 1.89-2.97, 2.28±0.21; leg IV: 5.00-5.88, 5.41±0.16; 2.50-3.05, 2.79±0.11; PTl 2.34-2.76, 2.55±0.07; PTw 0.71-1.04, 0.89±0.06; Bl 1.13-1.26, 1.20±0.02; TSp 3-5, 4.4±0.4; TSr 3-5, 3.8±0.49; TSrd 4-5, 4.6±0.24.

**Variation, females (5).** Cl 9.13-10.0, 9.38±0.17; Cw 7.44-8.50, 8.02±0.21; STRl 5.13-5.81, 5.50±0.14; STRw 4.38-5.00, 4.64±0.13; LBw 1.44-1.71, 1.56±0.05; LBl 0.80-0.98, 0.87±0.03; Leg I: 19.82-22.63, 21.29±0.56; ANTd 7-8, 7.6±0.24; PTLs 10-15, 13.00±0.89; TBs 3-7, 5.00±0.84.

#### GenBank accessions.

16S-tRNAval-12S: EU569971-EU569918, EU569964-EU569990, EU570023-EU570027, JX103256-JX103292; 18S (partial)-ITS1-5.8S-ITS2: EU569876-EU569879.

#### Distribution and natural history.

*Aptostichus atomarius* is widely distributed throughout southern California and has been collected in San Diego, Orange, Riverside, San Bernardino, Inyo, Los Angeles, Ventura, and San Luis Obispo counties (Map 2). Populations to the south are found throughout the Peninsular Ranges including the San Ysidro and Jamul Mountains, bounded to the east by the Laguna Mountain range. Moving northward in the Peninsular Ranges populations are abundant in the San Jacinto and Santa Ana Mountains. Like species described for the closely related genus *Apomastus* Bond and Opell 2002, *Aptostichus atomarius* was probably once more extensively distributed across the Los Angeles Basin but is now restricted to the steep ravines of the surrounding San Gabriel and Santa Monica Mountains and the Palos Verdes Hills along the coast. Populations to the north are distributed throughout the northeast extent of the Transverse Ranges bounded by the San Bernardino Mountains but extending northward along the coast in the Santa Ynez Mountains into the southernmost extent of the Coastal Ranges. A couple of morphologically similar specimens, collected from Tulare County in the southern extent of the Sierra Nevada Mountain range, are considered herein to be *Aptostichus atomarius* but will likely be removed to a separate species at a later date. From a geographical perspective it would seem reasonable to include these specimens in the *Aptostichus dantrippi* hypothesis, however, *Aptostichus dantrippi* individuals are distinctive morphologically and are considered a separate cohesion species at this time. Future molecular studies will likely resolve the placement of these outlier populations but until such data are available they are retained here as *Aptostichus atomarius* on the basis of their morphological affinity with all other specimens. Finally, a number of additional allopatric populations are found out on the Channel Islands and have likely been separated for a considerable amount of time but are likewise retained herein as *Aptostichus atomarius* until additional character systems can be employed to resolve their placement (or allocation to a new species).

The DM produced for this species (Map 3) shows the areas with the highest probability of predicted occurrence to be in the Peninsular and southernmost extent of the Transverse Ranges (around the Los Angeles Basin). Many of the populations in the desert to the east and the Coastal and Sierra Nevada Ranges to the north fall in areas predicted to have a very low probability of occurrence in the model, likely reflecting the fact that this species delineation comprises > 1 species.

In the Peninsular, Transverse, and Coastal Ranges, *Aptostichus atomarius* is restricted primarily to the California Coastal Range Open Woodland-Shrub-Coniferous and California Coastal Chaparral Forest and Shrub ecoregions. Vegetation types comprise mainly mixed chaparral, chamise-redshank chaparral, and coastal scrub. Male dispersal times are widely varied across specimens examined as part of this study. However, the majority of males were collected during the late fall to winter months of October through to March. Occasionally specimens have been collected during the summer months of June and August but these represent very rare occurrences. Females construct burrows that are typical for members of the genus.

#### Conservation status.

*Aptostichus atomarius* is widespread, abundant, and appears to thrive in moderately developed suburban areas throughout Southern California and thus is likely secure.

#### Species concept applied.

Morphological. *Aptostichus atomarius* presents a number of problems with respect to species delineation and diagnosis and thus is delineated herein as a single morphologically distinguishable species with the expectation that it will be further divided at some point in the future on the basis of other criteria.

#### Remarks.

**T**he type locality of *Aptostichus atomarius* as “San Bernardino” coupled with the relatively similar morphology of *Aptostichus atomarius* and *Aptostichus icenoglei* females contributes some uncertainty to the identity of the species attributed to this name. That is, without a more specific locality or the ability to recover DNA for molecular studies from the type specimen, the *Aptostichus atomarius* type could potentially be either. However, as discussed in the diagnosis the sternum of *Aptostichus icenoglei* is subtlety narrower than that of *Aptostichus atomarius*. Based on my morphological and molecular surveys of many specimens of both species I feel confident that *Aptostichus atomarius*, as described by Simon, is correctly attributed to the specimens I have identified as such. Second, as mentioned above, the molecular data indicate that the *Aptostichus atomarius* specimens listed in the material examined section above likely comprise a number of additional species. However, the data and sampling currently on hand are, in my opinion, insufficient to further split this complex into species lineages. Future studies of the entire *Atomarius* species complex will address these issues.

### Aptostichus stephencolberti

Bond, 2008

‘The Stephen Colbert Trapdoor Spider’

urn:lsid:zoobank.org:act:D4432939-144C-4E2F-BBF4-4632013E4927

http://species-id.net/wiki/Aptostichus_stephencolberti

Figures 82 –90 Maps 4, 5

Aptostichus stephencolberti Bond, 2008: 646. Male holotype (MY3515, EU570029) and female paratype (MY3513, EU570028), California, San Mateo Co., Pescadero State Beach, 37.26598, -122.41219^1^, elev. 3m, coll. A. Stockman & P. Marek, 29.i.2008, in CAS, examined.

#### Diagnosis.

**Figures 82–87.**
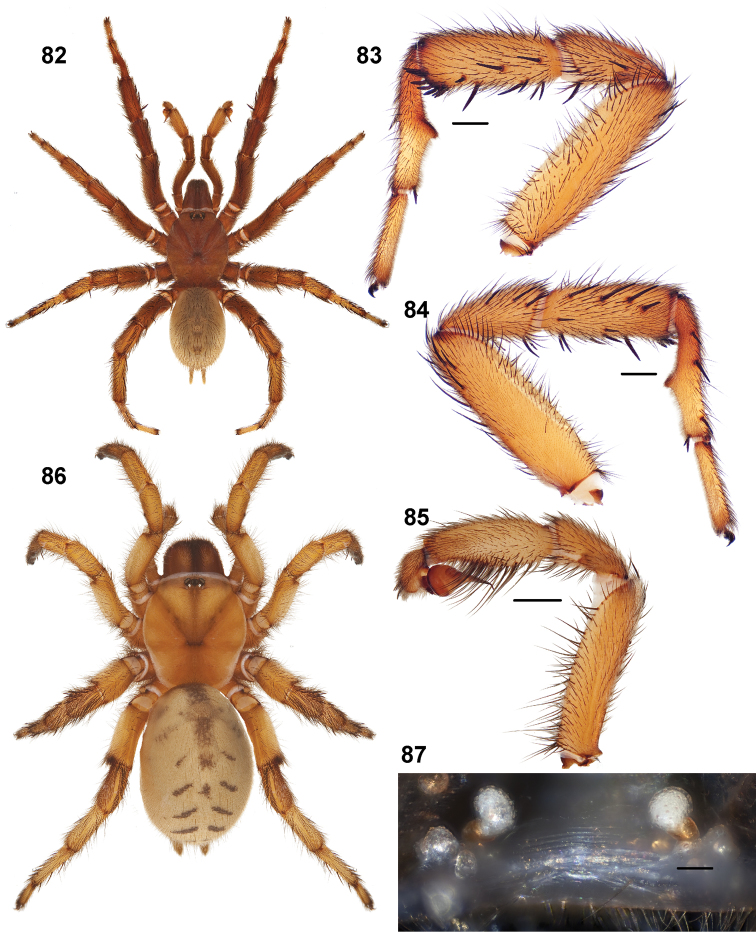
*Aptostichus stephencolberti* Bond, 2008. **82** male habitus from San Francisco Co. (AP156) [805762] **83–85** male holotype from San Mateo Co., Pescadero State Beach (MY3515); scale bar = 1.0mm **83 **retrolateral aspect leg I [805751] **84** prolateral aspect leg I [805747] **85** retrolateral aspect pedipalp [805753] **86** female habitus from Santa Cruz Co., Waddell Creek Beach (MY3508) [805758] **87** cleared spermathecae, from San Luis Obispo Co., Wilder Ranch State Beach (MY3500) [805754]; scale bar = 0.25mm.

**Figures 88–90.**
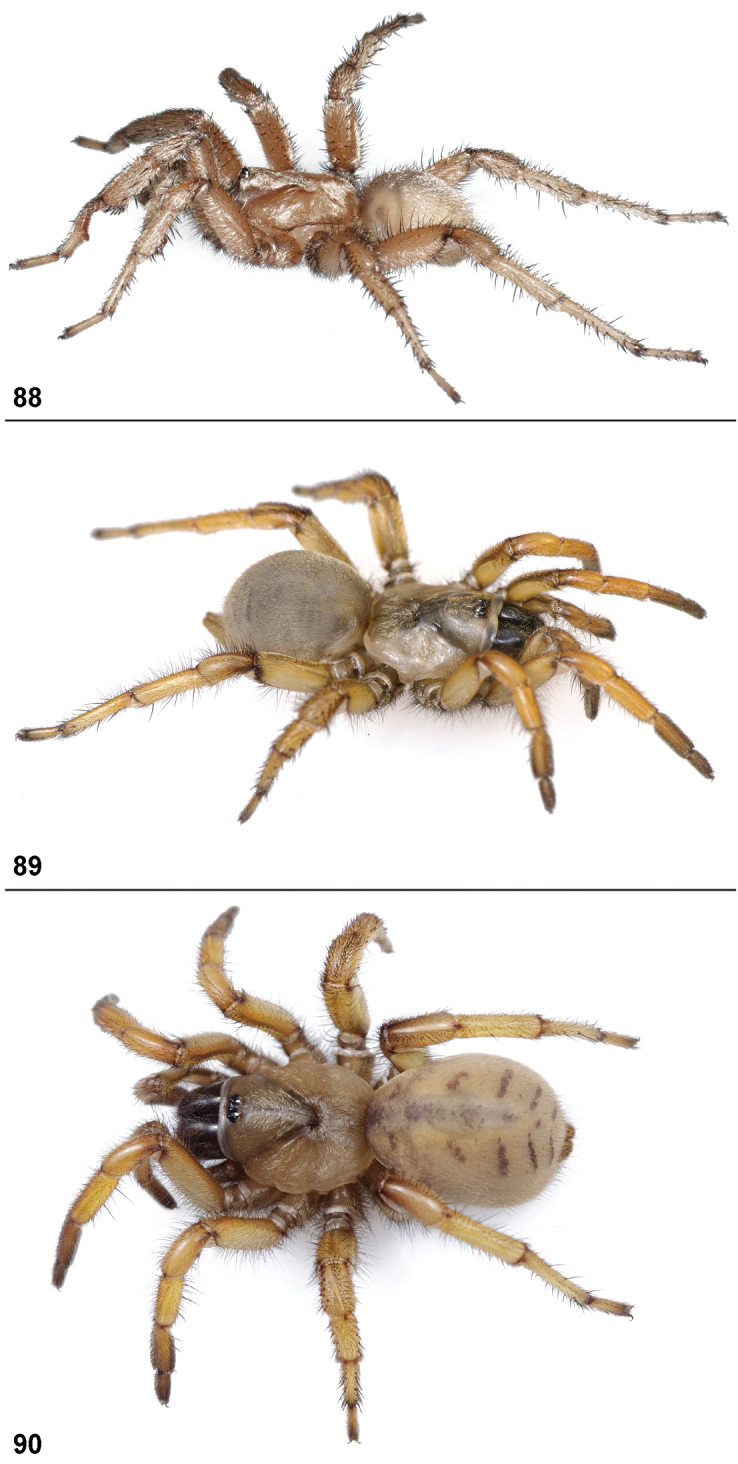
*Aptostichus stephencolberti* specimens, live photographs. **88** male holotype from San Mateo Co., Pescadero State Beach (MY3515) **89, 90** specimens from Monterey Co., Marina Dunes State Park (AUMS20).

**Maps 4, 5.**
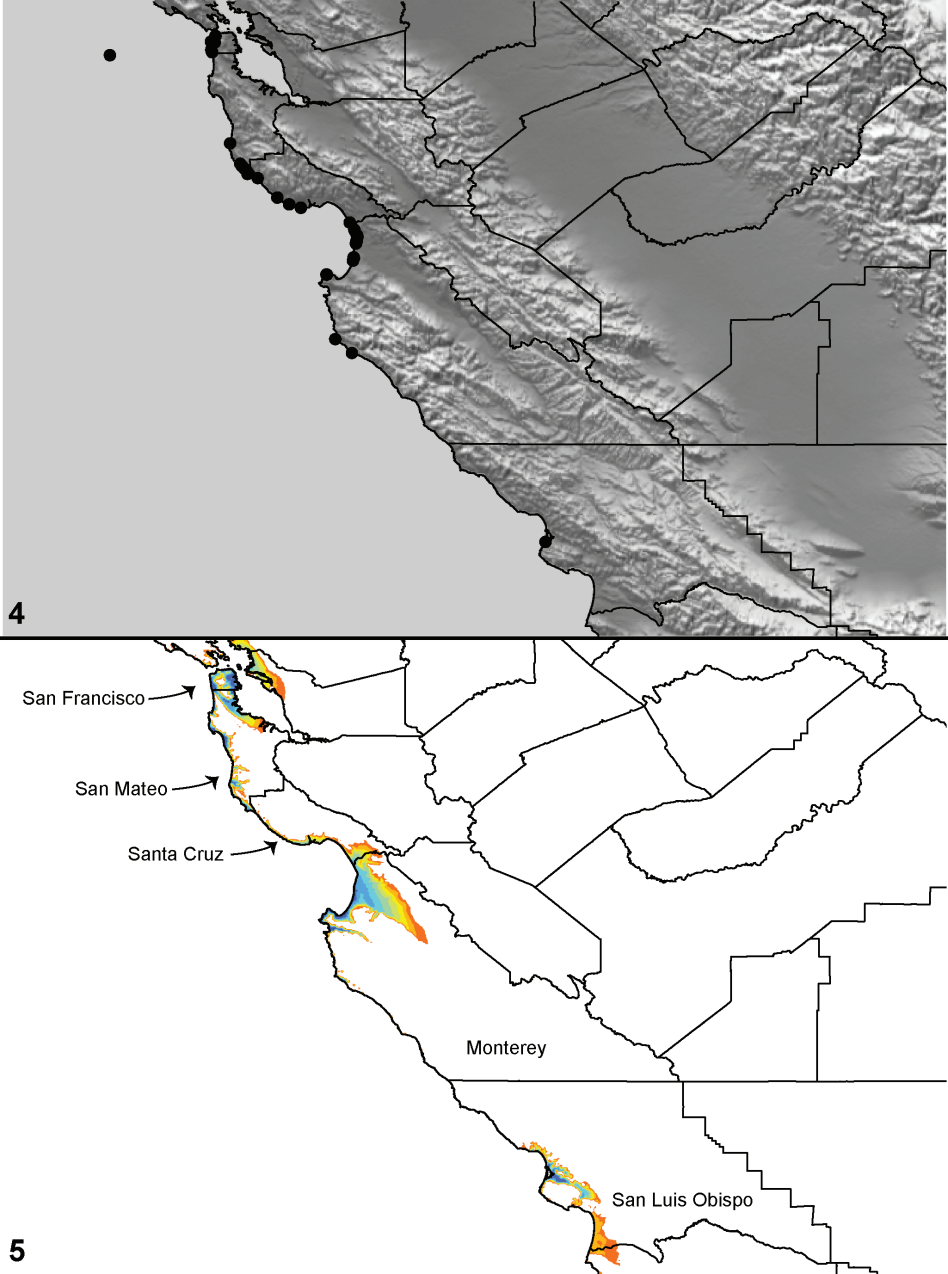
*Aptostichus stephencolberti*, Bond 2008. **4** distribution of known specimens **5** predicted distribution; cooler colors–blue shades–represent areas of high probability of occurrence, warmer colors–yellow and orange shades–represent areas of low probability of occurrence.

Individuals of this species are difficult to distinguish from other *Atomarius* Sibling Species Complex members on the basis of morphological features (Figs 82–90) alone but can be diagnosed on the basis of a set of unique mtDNA site substitutions (see Bond and Stockman 2008). The species is restricted in distribution to the coastal dune habitats of Monterey, Santa Cruz, San Mateo, and San Francisco Counties (California; Maps 4, 5) and is much lighter in coloration (Figs 88–90) than species found in inland habitats (e.g., *Aptostichus atomarius*, *Aptostichus angelinajolieae*, and *Aptostichus stanfordianus*).

#### Descriptions.

Described by Bond (2008).

#### Material examined.

**United States: California: Monterey Co.:** Asilomar St Beach, 36.6221, -121.9391^3^, 8m, W Icenogle 14.x.1971 [AP463-64, 3♀, 7juv, CAS]; Asilomar St Beach, 36.6236, -121.9405^1^, 3m, J Bond 15.v.1997, [AP800-802, 812-13, 2♀4juv, AUMNH]; 2.5km S Marina Ranger Station, 36.6969, -121.8088^1^, 14m, J Bond 16.v.1997 [AP803, 814-16, 4juv, AUMNH]; end Dunes Rd, N Marina St Beach, 36.7068, -121.807^1^, 8m, J Bond, W Bond 27.vii.2008 [MY3750-51, 2♀, AUMNH]; dunes Marina Beach, 36.705, -121.8077^1^, 3m, J Bond 4.v.1997 [AP772-74, 13♀, AUMNH]; Marina Beach (dunes across from RV park), 36.7033, -121.8063^1^, 7m, J Bond 2-3.iv.1996 [AP711 722, 739-40, 748,, 4♀, 1juv, AUMNH]; Marina Beach dunes, 36.703, -121.808^1^, 13m, J Bond 2.iv.1996 [AP736, 1♀, AUMNH]; Marina State Beach, 36.6973, -121.8092^4^, 12m, M Ramirez 16.v.1983 [AP278, 1juv, AMNH], 28.x.1984 [AP468, 1♀, AMNH], E Schlinger 23.iv.1970 [AP485, 2♀, SCW]; Marina Dunes Natural Preserve, 36.6905, -121.8105^2^, 3m, J Bond, D Beamer, A Stockman 17.iii.2005 [MY3069-70, 1♀ 1juv, AUMNH]; Moss Landing St Beach, 36.8086, -121.7883^1^, 3m, J Bond 14.iv.1997 [AP1265, 1♀, AUMNH]; Moss Landing St Beach, 36.8155, -121.79^1^, 0m, M Hedin, M Lowder 28.vi.2000 [AP1267, 1♀, AUMNH], J Bond 21.xii.1997 [AP706, 1♀, AUMNH]; Point Sur Lighthouse, 36.3062, -121.8971^1^, 0m, M Hedin, M Lowder 29.vi.2000 [AP1252, MY699–MY701, 3♀, 2juv, AUMNH]; Salinas River St Beach, 36.7823, -121.7939^1^, 9m, W Icenogle 14.x.1999 [MY0713, 1♀, CAS]; Salinas River St Beach, 36.7831, -121.7944^1^, 3m, J Bond 17.v.1997 [AP758-60, AP804, 4juv, AUMNH], 6.v.1997 [AP778, 1juv, AUMNH]; Salinas River St Beach, Molera Rd Parking Lot, 36.7752, -121.796^4^, 6m, M Ramirez 16.iv.1983 [AP258, AP306, 2♀, AMNH]; Salinas River St Beach, 36.7855, -121.7945^3^, 3m, A Stockman, P Marek 28.i.2006 [MY3489-91, 3juv, AUMNH]; Salinas River St Beach, 36.7907, -121.7919^1^, 3m, J Bond 8.xii.2005 [MY3455-57, 2♀, 1juv, AUMNH]; Zmudowski St Beach, 36.8436, -121.8041^2^, 3m, J Bond 15.v.1997 [AP797-99, 809-11, 5♀, 1juv, AUMNH]; Zmudowski St Beach, 36.8358, -121.802^1^, 3m, J Bond 8.xii.2005 [MY3461-63, 2♀, 1juv, AUMNH]; Pfeiffer Beach, 36.2384, -121.8154^2^, 6m, J Bond 7.xii.2005 [MY3451, 1juv, AUMNH]; **San Francisco Co.:** Ft Funston National Recreation Area, 37.7231, -122.5043^3^, 31m, M Ramirez 23.vi.1988 [AP196, 1♀, CAS]; Ft Funston National Recreation Area, 37.7121, -122.5014^1^, 48m, A Stockman, P Marek 30.i.2006 [MY3517-20, 1♀, 3juv, AUMNH]; San Francisco, near jct Lincoln Way & 46 Ave, 37.7642, -122.5062^3^, 16m, L Trautz 1.x.1969 [AP1273, 1♀, SCW)]; Petrel Bluff, 37.6978, -123.0017^7^, 3m, M Ramirez 30.vii.1986 [AP156, 1♂, CAS]; San Francisco, Baker Beach, 37.792, -122.484^4^, 7m, D Ubick 8.iii.1980 [AP301, 3♀, CAS]; San Francisco, Sunset District, 37.7601, -122.4877^5^, 71m, J McNally 3.x.1969 [AP255, 1♀, CAS]; **San Mateo Co.:** Gazos Creek Coastal Access off HWY 1, 37.1659, -122.3625^3^, 10m, M Thompson 17.ix.1976 [AP1278, 2♀, 1♂, CAS]; Ano Nuevo St Reserve, 37.1165, -122.3277^1^, 1m, M Hedin, M Lowder 28.vi.2000 [MY698, AP1253, 1♀, 3juv, AUMNH]; Ano Nuevo St Reserve, Cascade Creek Trail, 37.139, -122.3403^2^, 3m, A Stockman, P Marek 29.i.2006 [MY3509, 1♀, AUMNH]; Ano Nuevo St Reserve, Franklin Point Trail, 37.1551, -122.3555^1^, 7m, A Stockman, P Marek 29.i.2006 [MY3510-12, 2♀, 4juv, AUMNH; 1juv, AUMNH]; Pescadero St Beach, 37.2659, -122.4121^1^, 4m, A Stockman, P Marek 29.i.2006 [MY3513-16, 2♀, 2♂, AUMNH]; **San Luis Obispo Co.:** S Morro Bay, 0.64km N parking lot**,** 35.3136, -120.7679^4^, 24m, W Icenogle 16.x.1999 [MY712, 1♀, CAS]; **Santa Cruz Co.:** Younger Lagoon, 36.9508, -122.0658^4^, 11m, M Ramirez 27.vii.1987 [AP437, 1juv, CAS]; Bonny Doon Beach W Bonny Doon Rd, 37.0003, -122.181^1^, 8m, A Stockman, P Marek 29.i.2006 [MY3501-04, 2♀ 2juv, AUMNH]; Sunset St Beach, 36.878, -121.8261^1^, 1m, A Stockman, P Marek 28.i.2006 [MY3492-97 1♀, 5juv, AUMNH]; Waddell Creek Beach, 37.0953, -122.277^1^, 11m, A Stockman, P Marek 29.i.2006 [MY3505, 08, 3♀ 1juv, AUMNH]; Wilder Ranch St Park, parking area N main entrance, 36.9668, -122.1228^1^, 4m, A Stockman, P Marek 29.i.2006 [MY3498-500, 3♀, AUMNH; MY3499, 1♀, AUMNH].

**Variation, males (3).** Cl 5.65-6.38, 6.09±0.22; Cw 4.85-5.63, 5.37±0.26; STRl 3.10-3.60, 3.28±0.16; STRw 2.76-3.25, 3.00±0.14; LBw 0.85-0.94, 0.91±0.03; LBl 0.51-0.53, 0.52±0.01; leg I: 5.10-5.50, 5.33±0.12; 3.25-3.75, 3.53±0.15; 3.25-3.75, 3.53±0.15; 2.00-2.35, 2.23±0.12; 1.75-2.25, 2.08±0.17; leg IV: 5.00-5.25, 5.15±0.08; 2.35-3.00, 2.70±0.19; PTl 2.25-2.50, 2.42±0.08; PTw 0.85-1.00, 0.93±0.04; Bl 1.05-1.45, 1.29±0.12; TSp 3-6, 4.33±0.88; TSr 0-6, 3.00±1.73; TSrd 4-8, 5.67±1.20.

**Variation, females (5).** Cl 7.20-9.00, 7.98±0.32; Cw 6.56-7.90, 7.39±0.23; STRl 3.90-5.15, 4.63±0.22; STRw 3.70-4.75, 4.31±0.17; LBw 1.28-1.70, 1.50±0.07; LBl 0.85-1.02, 0.94±0.04; Leg I: 14.05-20.51, 17.74±1.08; ANTd 6-10, 7.60±0.81; PTLs 16-29, 20.40±2.38; TBs 5-11, 7.20±1.11.

#### GenBank accessions.

16S-tRNAval-12S: EU569930-EU569939, EU570008-EU570021, EU570028-EU570031, JX103357- JX103366; 18S (partial)-ITS1-5.8S-ITS2: EU569888-EU569892.

#### Distribution and natural history.

*Aptostichus stephencolberti* is distributed throughout the coastal dune habitats of San Francisco, San Mateo, Santa Cruz, and Monterey Counties with a single population recorded from San Luis Obispo County (Map 4). Individuals are found in relatively deep burrows on the steep faces of sand dunes and at the base of coastal vegetation. Burrows comprise a thick silk lining and are covered by a very cryptic trapdoor constructed of silk and sand. Dune habitats disturbed by high concentrations of the invasive *Carpobrotus edulis* (ice plant) tend to lack *Aptostichus stephencolberti* individuals entirely. The DM for this species (Map 5) follows closely with the known distribution to include the southernmost-recorded locality for the species. Wandering males (2) have been collected in August and September; two males have been collected from burrows in January.

#### Conservation status.

*Aptostichus stephencolberti* is generally abundant at localities along the California coast (Map 4). However, the species is particularly vulnerable to invasive plant species (e.g., ice plant, *Carpobrotus edulis*) and is highly structured genetically across its distribution thus many populations contain unique alleles (Bond and Stockman 2008). Moreover, divergent populations at the southernmost extent of the distribution comprise very few individuals (specimens are rare, despite extensive searching). Consequently, I would consider this species to be apparently secure or vulnerable, contingent upon future delimitation of divergent lineages as nominal taxa.

#### Species concept applied.

Cohesion. *Aptostichus stephencolberti* is considered to be ecological non-interchangeable from other closely related lineages–it is restricted to coastal dune habitat and has psammophilic features (e.g., lighter coloration).

### Aptostichus angelinajolieae

Bond, 2008

‘The Angelina Jolie Trapdoor Spider’

urn:lsid:zoobank.org:act:04A7F666-D585-43A7-A5A0-45FEF5B8DE75

http://species-id.net/wiki/Aptostichus_angelinajolieae

Figures 91 –97 Maps 6, 7

Aptostichus angelinajolieae Bond, 2008: 646. Female holotype (MY3310; EU569958), California, Monterey Co., Carmel Valley Rd/G16, 5.9km N of Arroyo Seco Rd, 36.29045, -121.46594^1^, elev. 337m, coll. A. Stockman 9.vi.05, in CAS; male paratype (AP167) from Carmel, 36.5495, -121.9103^6^, elev. 74m, coll. 8.ii.54, in AMNH examined.

#### Diagnosis.

**Figures 91–94.**
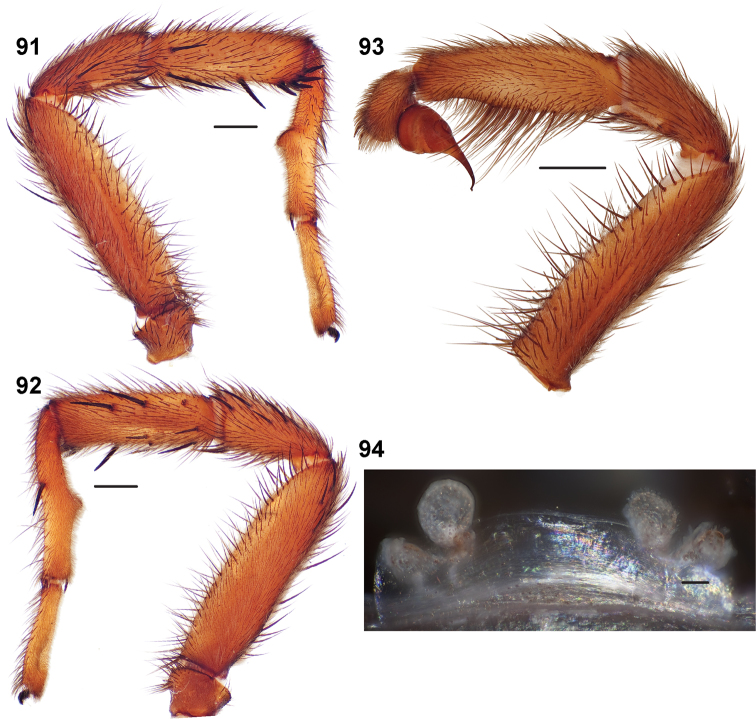
*Aptostichus angelinajolieae* Bond, 2008. **91–93** male paratype from Monterey Co., Carmel (AP167); scale bars = 1.0mm **91** retrolateral aspect right leg I [805766] **92** prolateral aspect right leg I [805770] **93** retrolateral aspect pedipalp [805772] **94** cleared spermathecae, female holotype from Monterey Co., Carmel Valley (MY3310) [805773]; scale bar = 0.25mm.

**Figures 95–97.**
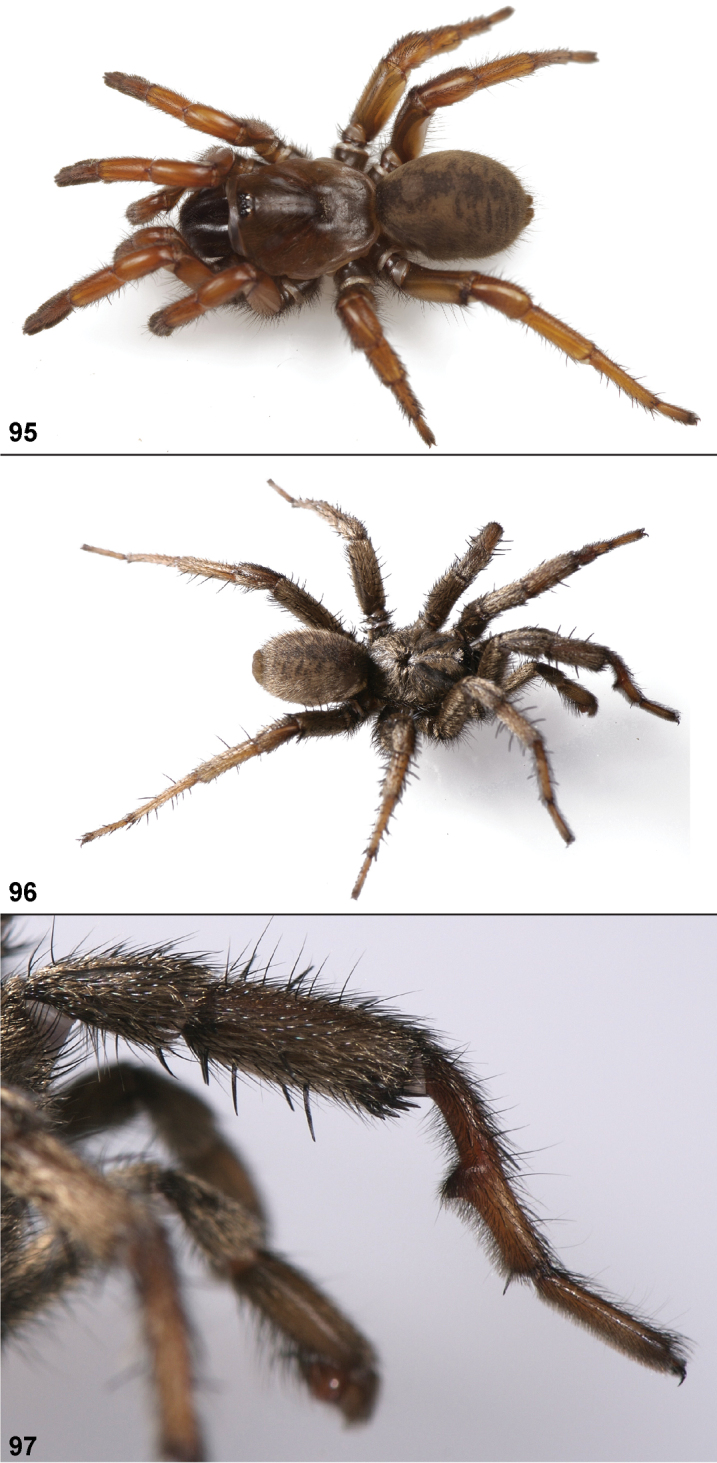
*Aptostichus angelinajolieae* specimens from Monterey Co., Monterey, live photographs. **95** female specimen (AUMS62) **96, 97** malespecimen (MY3630) **97** close up shot of leg I, retrolateral aspect, in life.

**Maps 6, 7.**
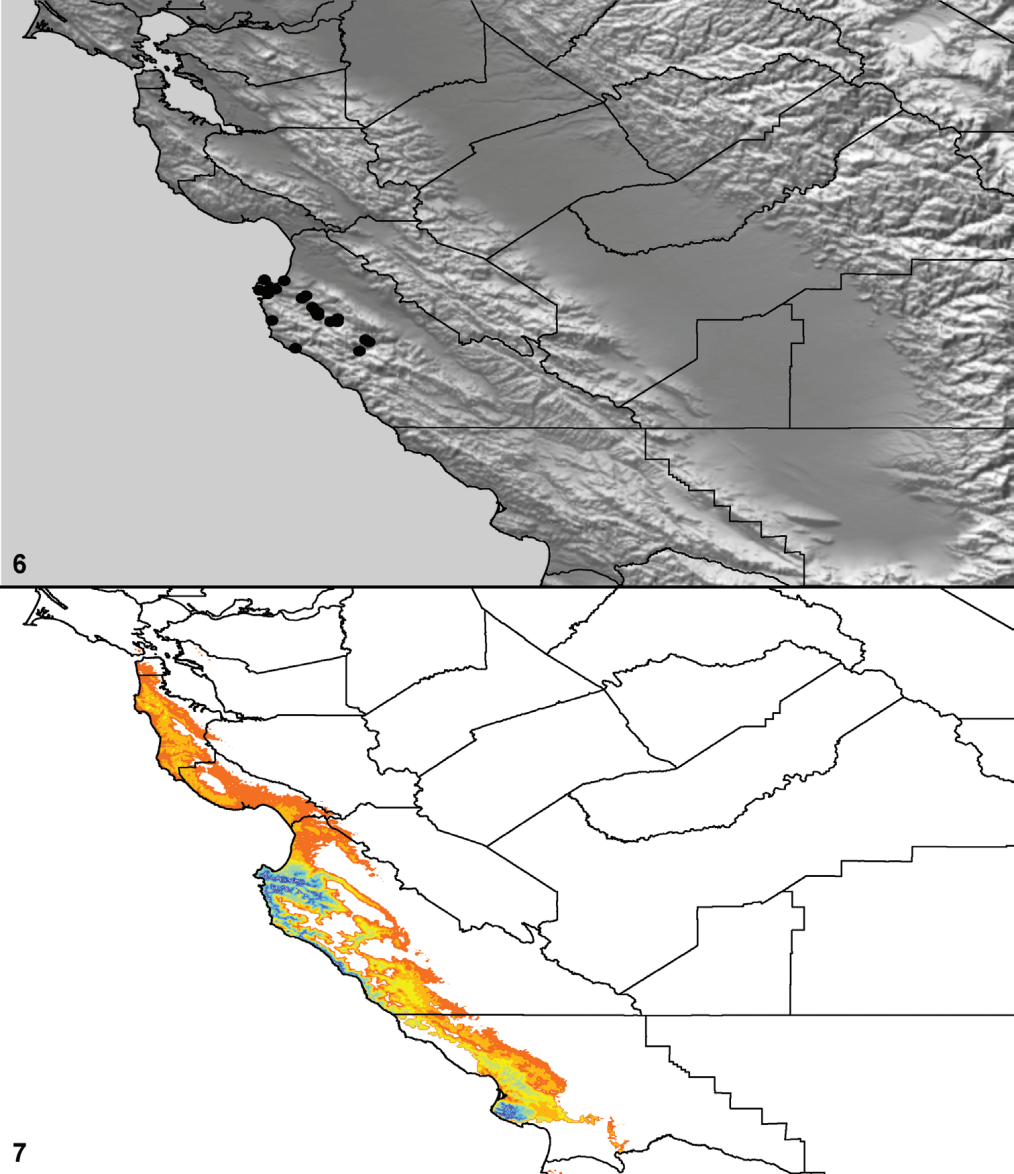
*Aptostichus angelinajolieae* Bond, 2008. **6** distribution of known specimens **7** predicted distribution; cooler colors–blue shades–represent areas of high probability of occurrence, warmer colors–yellow and orange shades–represent areas of low probability of occurrence.

Individuals of this species are difficult to diagnose from *Aptostichus atomarius* and *Aptostichus stanfordianus* on the basis of morphological features alone (Figs 91–94) but can be diagnosed on the basis of a set of unique mtDNA site substitutions (see Bond & Stockman 2008). The species is restricted in distribution to northern Monterey Co. (California) localities west of the Salinas Valley (Maps 6, 7) and can be distinguished from geographically proximate populations of *Aptostichus stephencolberti* on the basis of its darker coloration (Figs 95–97) and absence from coastal dune habitat.

#### Descriptions.

Described by Bond (2008).

#### Material examined.

**United States: California: Monterey Co.:** 1.6km W Seaside, 36.6227, -121.8363^5^, 30m, M Irwin 23.viii.68 [AP289, 3juv, AMNH]; 2.2km from intersection G16/Carmel Valley Rd & Cachagua Rds on Cachagua Rd, 36.4411, -121.6877^1^, 387m, J Bond 5.v.97 [AP754-76, 2♀, AUMNH; 1♀, AMNH]; 24km W Greenfield on Arroyo-Seco Rd, 36.2263, -121.4947^5^, 300m, W. Icenogle 31.v.74 [AP193, 2♀, CAS]; Aguajito Rd & Monhollan Rd jct, 36.5753, -121.8737^1^, 56m, A Stockman 10.vi.05 [MY3317, 1♀, AMNH; MY3318, 3380 2♀, AUMNH]; Cachagua Rd, 160m W jct w/ Carmel Valley Rd, 36.4455, -121.6827^1^, 191m, A Stockman 10.vi.05 [MY3311-13, 2juv, 2♀, AUMNH]; Cachagua Rd, 13.7km W jct w/ Carmel Valley Rd, 36.392, -121.6252^1^, 333m, A Stockman 10.vi.05 [MY3315, 3316 2♀, AUMNH]; Cachagua Rd, just S intersection w/G16, 36.4447, -121.6855^1^ 1, 245m, M Hedin, J Starrett, D Leavitt 21.xii.07 [MY3620, 3626, 3627, 3630, 3631, 2♀ 1♂ 2juv, AUMNH]; Cachagua Rd, 160m from jct Carmel Valley Rd, 36.4454, -121.6833^1^, 225m, J Bond, M van der Merwe 13.xii.99 [MY708, 716 2♀, AUMNH]; J Bond, M van der Merwe 13.xii.99 [MY716, 1♀, AUMNH]; Calera Canyon Rd, 1.6km S Corral De Tierra Rd, 36.5383, -121.7376^1^, 137m, A Stockman 10.vi.05 [MY3319, 3321 2♀, AUMNH; MY3320, 1♀, AMNH]; Carmel, 36.5495, -121.9103^6^, 74m, 25.vii.67 [AP155, 1♂, AMNH], 8.ii.54 [AP167, 1♂, AMNH], 5.x.58 [AP177, 1♂, AMNH], V Roth 21.xii.53 [AP267, 2juv, AMNH]; Carmel Valley Rd, 36.4511, -121.6919^1^, 165m, A Stockman 15.v.05 [MY3129-3131 3♀, AUMNH]; Carmel Valley Rd (G16), 1.3km N Tassajara Rd, 36.4096, -121.5934^4^, 426m, J Starrett 29.xi.03 [AP1198, 1♀, AUMNH]; Carmel Valley Rd/G16, 5.9km N Arroyo Seco Rd, 36.2904, -121.4659^1^, 337m, A Stockman 9.vi.05 [MY3309, 3310 2♀, AUMNH]; County Rd G16, 17.7km S Martin Rd intersection, 36.2776, -121.4503^5^, 335m, F Coyle 30.vii.72 [AP268, 1♀, AMNH]; Cachagua Rd 6.5 miles SE Carmel Valley Village, 4.2km S intersection w/Carmel Valley Rd, 36.4278, -121.6825^4^, 460m, W Icenogle 2.vi.74 [AP279, 1♀, CAS]; G20 (Laureles Grade Rd), ~1.3km S Laureles Summit, 36.524, -121.7558^1^, 315m, M Hedin, J Starrett, D Leavitt 21.xii.07 [MY3623, 3628 2♀, AUMNH]; Intersection of Valenzuela & Viejo Rds, 36.5761, -121.8998^1^, 120m, J Bond 1.iv.96 [AP723, 727 1♀ 1juv, AUMNH], 2.iv.96 [AP742, 1♀, AUMNH], 5.v.97 [AP751, 752-3, 6, 2juv, 2♀, AUMNH]; Klondike Rd, 6.4km E G16/Carmel Valley Rd, 36.4731, -121.707^1^, 169m, J Bond, W Bond 27.vii.08 [MY3736, 1♀, AUMNH]; Monterey, ~100m N intersection Vieja & Valenzuela Rds, 36.5759, -121.8996^1^, 91m, M Hedin, P Paquin, J Starrett 27.vii.02 [MY0632, 1♀, AUMNH]; Near jct Cachagua & Tassajara Rds, 36.3908, -121.5955^4^, 410m, W Icenogle 2.vi.74 [AP277, 1♀, CAS]; Pacific Grove, 36.628, -121.9253^6^, 30m, 7.xi.53 [AP163, 1♂, AMNH], W Ivie 1.ix.37 [AP274, 1juv, AMNH], 16.viii.31 [AP472, 1♀, 6juv, AMNH]; Palo Colorado Rd, 1.4km E Hwy 1, near Rocky Point, 36.3997, -121.8914^1^, 105m, J Bond, A Stockman, D Beamer 17.iii.05 [MY3057, 3060, 2♀, AUMNH]; Pebble Beach, 36.5688, -121.9478^6^, 25m, W Icenogle 1.vi.74 [AP291, 1♀, AMNH]; Sycamore Canyon Rd ~0.72km W jct HWY 1, 36.2418, -121.7841^1^, 104m, J Bond 7.xii.05 [MY3452-54, 2♀ 1juv, AUMNH]; Viejo Rd, 36.5778, -121.90076^5^, 138m, W Icenogle 1.vi.74 [AP197, 1♀, 3juv, CAS].

**Variation, males (5).** Cl 5.81-7.50, 6.30±0.31; Cw 5.00-6.19, 5.34±0.22; STRl 3.20-4.19, 3.49±0.18; STRw 2.70-3.27, 2.90±0.1; LBw 0.85-1.02, 0.94±0.04; LBl 0.43-0.63, 0.56±0.04; leg I: 5.25-6.44, 5.58±0.22; 3.50-4.25, 3.73±0.14; 3.65-4.44, 3.91±0.15; 2.25-2.61, 2.34±0.07; 1.75-2.55, 2.25±0.14; leg IV: 5.06-6.25, 5.43±0.28; 2.50-3.13, 2.81±0.13; PTl 2.51-3.00, 2.72±0.08; PTw 0.76-1.02, 0.89±0.04; Bl 1.19-1.49, 1.32±0.05; TSp 3-6, 4.20±0.58; TSr 3-5, 4.20±0.37; TSrd 4-5, 4.40±0.24.

**Variation, females (5).** Cl 6.69-8.96, 7.55±0.45; Cw 5.31-8.16, 6.50±0.53; STRl 3.75-5.70, 4.41±0.39; STRw 3.12-4.75, 3.70±0.33; LBw 1.07-1.55, 1.31±0.1; LBl 0.71-1.19, 0.93±0.08; Leg I: 14.56-20.94, 17.27±1.32; ANTd 6-8, 7.20±0.37; PTLs 14-17, 15.00±0.55; TBs 2-5, 3.00±0.55.

#### GenBank accessions.

16S-tRNAval-12S: EU569940-EU569958, EU569991, JX103243-JX103249; 18S (partial)-ITS1-5.8S-ITS2: EU569880-EU569883.

#### Distribution and natural history.

*Aptostichus angelinajolieae* is restricted in distribution to the Santa Lucia Range of Monterey County (Map 6), bounded to the east by the Salinas River Valley (SRV). The ecoregion is characterized as California Coastal Chaparral Forest and Shrub. As discussed in Bond and Stockman (2008) the DM (Map 7) predicts the areas with the highest probability of occurrence in the regions east of the SRV, with the valley likely serving as a barrier to dispersal. The few male specimens known were collected during the late fall through winter months (October–December, February), with one specimen collected in July that molted to the final adult stage a month later in August. Female specimens are frequently found on shaded, damp steep banks and road cuts throughout the region. Burrows are generally shallow comprising a white silken lined retreat, covered by a thin silk-soil trapdoor.

#### Conservation status.

The conservation status of *Aptostichus angelinajolieae* is likely classified as secure because it is widespread, abundant, and appears to thrive in moderately developed residential areas.

#### Species concept applied.

Cohesion. *Aptostichus angelinajolieae* is considered a cohesion species on the basis of it exclusivity as a lineage in DNA studies and lack of genetic exchangeability (gene flow) with other *Atomarius* Sibling Species Complex lineages as a consequence of the SRV barrier to gene flow.

### Aptostichus miwok

Bond, 2008

‘The Miwok Trapdoor Spider’

urn:lsid:zoobank.org:act:3C1A0066-B8A1-40A5-8896-377CD61ECAE8

http://species-id.net/wiki/Aptostichus_miwok

Figures 98 –105 Map 8, 9

Aptostichus miwok Bond, 2008: 646. Female holotype (MY301; EU69907) from California, Humboldt Co., Clam Beach Co. Park, just S Crannell Rd exit on Clam Beach Dr, near Little River, 41.01333, -124.10923^1^, elev. 3m, coll., J. Bond & M. Hedin 13.i.02 in CAS, examined; male paratype (AP149) from Farallon Island, San Francisco Co., California, in CAS, examined).

#### Diagnosis.

**Figures 98–103.**
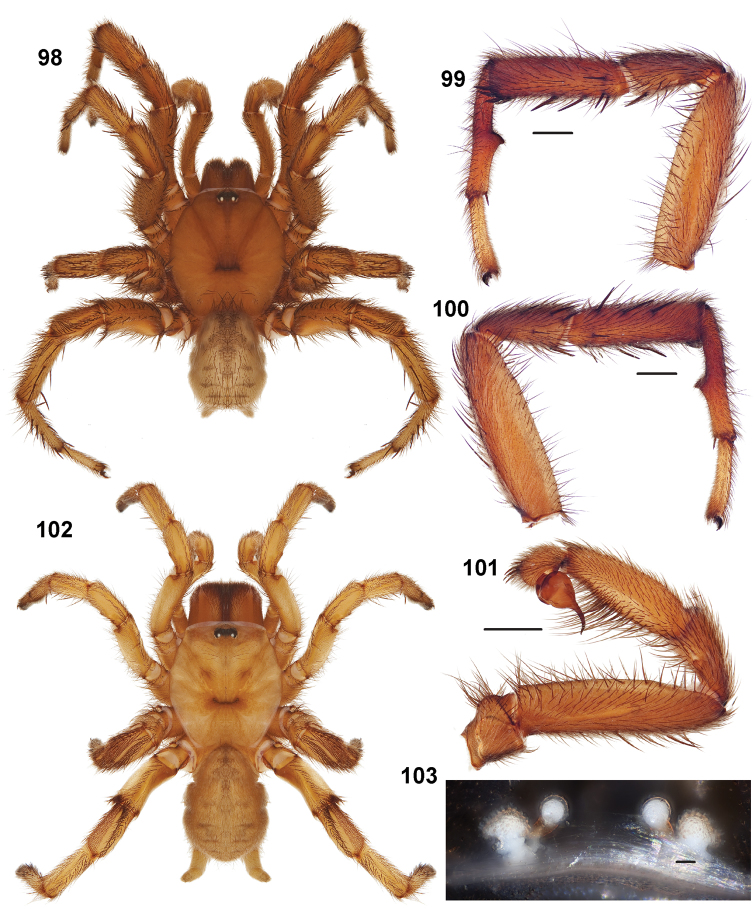
*Aptostichus miwok* Bond, 2008. **98** male habitus from Mendocino Co., Inglenook Dunes (AP1270) [805812] **99–101** male paratype (AP149) from San Francisco Co., Farallon Islands; scale bars = 1.0mm **99** retrolateral aspect, leg I [805801] **100** prolateral aspect, leg I [805797] **101** retrolateral aspect, pedipalp [805803] **102** female habitus from Mendocino Co., Inglenook Dunes (AP520) [805808] **103** spermathecae, female holotype (MY301) [805804]; scale bar = 0.10mm.

**Figures 104, 105.**
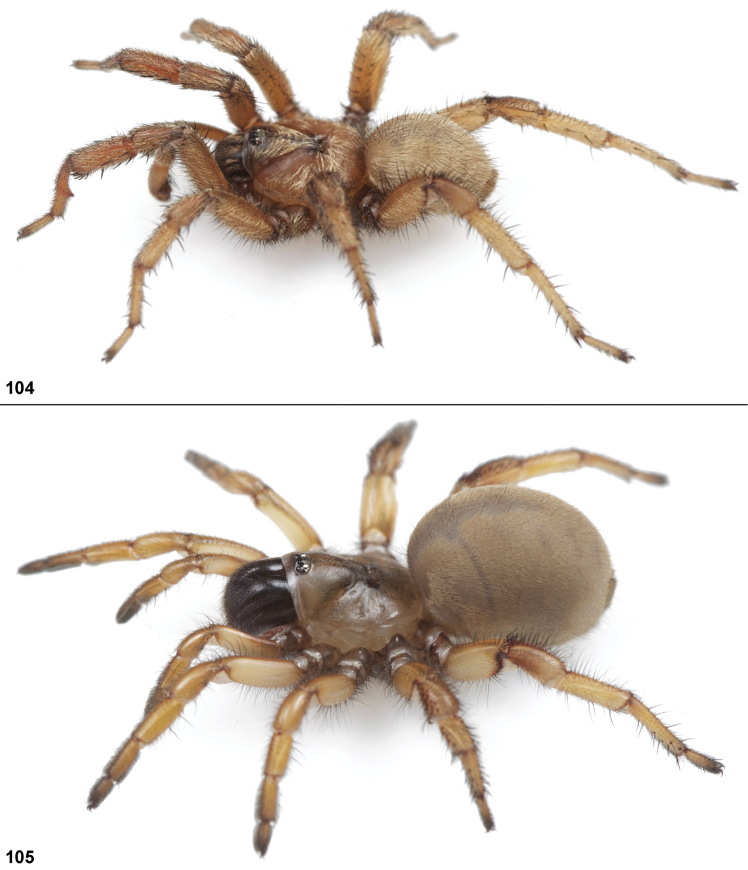
*Aptostichus miwok* from Mendocino Co., Inglenook Dunes area, live photographs. **104** male specimen (AUMS18) **105** female specimen (AUMS16).

Based on morphological features alone (Figs 98–103) this species can be difficult to diagnose from geographically proximate sibling species, *Aptostichus stanfordianus*, *Aptostichus angelinajolieae*, and *Aptostichus stephencolberti* but can be distinguished on the basis of a set of unique mtDNA nucleotide substitutions (see Bond and Stockman 2008). The species is restricted in distribution to the coastal dune habitats of Marin, Sonoma, Mendocino, Humboldt, and Del Norte Counties (California). Individuals of *Aptostichus stanfordianus*, found only in inland habitats,are much darker in coloration whereas *Aptostichus miwok* specimens are much lighter (Figs 104, 105).

#### Description.

Described by Bond (2008).

#### Material examined.

**United States: California: Del Norte Co.:** Smith River St Park, Beach 1km W Kellogg Rd from Lower Lake Rd, 41.8695, -124.2073^1^, 0m, J Bond, M Hedin 14.i.02 [MY290-93, 95, 3♀ 2juv, AUMNH]; **Humboldt Co.:** ~3.2km W Arcata, Lanphere-Christensen Dune Preserve, 40.8823, -124.1478^3^, 3m, E Schlinger 26.vii.75 [AP522, 1♀, CAS]; Clam Beach Co Park, just S Crannell Rd exit on Clam Beach Dr, near Little River, 41.0133, -124.1092^1^, 3m, J Bond, M Hedin 13.i.02 [MY300-04, 5♀, AUMNH]; S Spit Humboldt Bay, 40.6979, -124.2725^1^, 0m, M Hedin, M Lowder 22.vi.00 [MY702-04, 3♀, AUMNH]; S Spit Humboldt Bay, S Eureka, 40.6992, -124.2738^1^, 3m, M Hedin, R Keith, S Thomas, J Starrett 14.iii.06 [MY3542-46, 4♀, 1juv, AUMNH]; Trinidad, 41.0571, -124.1419^5^, 8m, B Malkin 29.v.69 [AP266, 1♀, AMNH]; Hwy 96, N of Willow Creek, 41.0096, -123.6455^1^, 210m, J Bond, A Stockman, D Beamer 15.iii.05 [MY3052, 1juv, AUMNH]; **Marin Co.:** Dillon St Beach, 38.2494, -122.9675^1^, 3m, J Bond 14.v.97 [AP763, 764, 793, 805, 4juv, AUMNH]; N Beach, P Reyes Natl Seashore, 38.0011, -122.9973^5^, 30m, E Rogers, 13.v.72 [AP465, 1♀, SCW]; E Schlinger 14.vi.75 [AP469, 1♀, CAS]; C Griswold 31.iii.75 [AP476, 1♀, CAS]; [AP479, 1♀, AMNH]; C Griswold 16.viii.81 [AP499, 1♀, CAS]; H Ewing 14.v.71 [AP1276, 1juv, CAS]; Pt Reyes Natl Seashore, 38.0262, -122.8831^3^, 3m, J Cate 12.v.73 [AP1274, 1juv, CAS]; Dillon Beach, 38.2499, -122.9676^2^, 1♂, A Stockman, P Marek 30.i.06 [MY3528, 1juv, AUMNH]; Pt Reyes Natl Seashore Kehoe Beach, 38.1553, -122.9483^1^, 9m, A Stockman, P Marek 30.i.06 [MY3524-27, 1♀ 1♂ 3juv, AUMNH]; Pt Reyes Natl Seashore, Limantour Beach, 38.0262, -122.8831^1^, 3m, A Stockman, P Marek 30.i.06 [MY3521-23, 2♀, 1 juv, AUMNH]; **Mendocino Co.:** 6.4km N Ft Bragg, Inglenook Fen area, 39.5304, -123.7712^3^, 12m, L Keram 11.iv.73 [AP520, 1♀, CAS]; E Schlinger 17.vi.73 [AP521, 1♀, CAS]; Hwy 1, just S Ten Mile River crossing, N Ingelnook, dunefield W hwy, 39.5476, -123.7631^1^, 5m, M Hedin, J Starrett 13.iii.06 [MY3538-41, 3♀ 1juv, AUMNH]; Inglenook Dunes, 39.5304, -123.7712^3^, 10m, R Jackson 1.xi.74 [AP1269, 1♂, CAS], 19.iv.75 [AP1270, 1♂, CAS]; E Schlinger 30.iv.73 [AP1271, 1juv, CAS], 22.vii.72 [AP1272, 3juv, CAS]; N Inglenook Sand Dunes, 39.5422, -123.7618^1^, 37m, J Bond, A Stockman, D Beamer 14.iii.05 [MY3049, 1juv, AUMNH]; **San Francisco Co.:** Petrel Bluff, 37.6978, -123.0017^7^, 3m, M Ramirez 31.viii.86 [AP498, 5♀, 2juv, CAS]; SE Farallon Island, 37.6978, -123.0024^4^, 7m, M Ramirez 1.ix.86 [AP438, 5♂, CAS], 2.ix.86 [AP475, 482, 149, 1♂ 2♀, CAS; AP491, 1juv, AMNH], 2.ix.88 [AP492, 2♀, AMNH], 1.ix.88 [AP007, 1♀, CAS], 30.viii.88 [AP189, 8♀, AMNH]; P Anderson 20.xii.56 [AP481, 1♀, 2juv, AMNH]; W Azevedo 12.iv.70 [AP494, 3♀, AMNH]; D Spadoni 10.iii.69 [AP505, 1♀ 1♂ 1juv, CAS]; D Hanna 6.v.49 [AP483, 2juv, AMNH]; H Leech 18.xi.49 [AP484, 6juv, AMNH]; W Hazeltine 23.x.51 [AP486, 2juv, AMNH]; H Keifer 15.x.26 [AP487, 1juv, AMNH]; E Bixford 17.iv.29 [AP502, 1♀, AMNH]; **Sonoma Co.:** Bodega Bay Dunes, 38.3137, -123.0391^3^, 1m, R Robertson 16.ix.92 [AP493, 1♂, CAS]; Sonoma Coast St Beach, Bodega Dunes, 38.3389, -123.06149^1^, 47m, A Stockman, P Marek 30.i.06 [MY3529-32, 4juv, AUMNH].

**Variation, males (4).** Cl 4.50-5.94, 5.35±0.30; Cw 4.25-5.00, 4.65±0.18; STRl 2.48-3.04, 2.78±0.13; STRw 2.33-2.73, 2.52±0.08; LBw 0.85-0.87, 0.86±0.01; LBl 0.43-0.60, 0.50±0.04; leg I: 4.10-5.30, 4.76±0.26; 2.48-3.50, 3.10±0.23; 2.36-3.30, 3.00±0.22; 1.52-2.00, 1.81±0.11; 1.55-2.05, 1.87±0.11; leg IV: 3.80-5.05, 4.44±0.26; 2.00-2.50, 2.25±0.1; PTl 1.85-2.38, 2.21±0.13; PTw 0.71-0.85, 0.79±0.03; Bl 0.85-1.17, 1.04±0.07; TSp 4-6, 4.75±0.48; TSr 1-3, 2.25±0.48; TSrd 4-5, 4.75±0.25.

**Variation, females (5).** Cl 6.06-7.00, 6.51±0.19; Cw 5.69-6.40, 6.01±0.14; STRl 3.32-4.00, 3.69±0.12; STRw 3.29-3.75, 3.50±0.10; LBw 1.11-1.36, 1.24±0.05; LBl 0.68-0.94, 0.81±0.05; Leg I: 13.00-16.15, 14.30±0.59; ANTd 5-8, 6.80±0.49; PTLs 16-25, 21.80±1.59; TBs 3-6, 5.20±0.58.

#### GenBank accessions.

16S-tRNAval-12S: EU569901-EU569910, EU569919-EU569921, EU569922, EU569927-EU569929, EU570022, EU570035, JX103340-JX103353; 18S (partial)-ITS1-5.8S-ITS2: EU569893, EU569894.

#### Distribution and natural history.

**Maps 8, 9.**
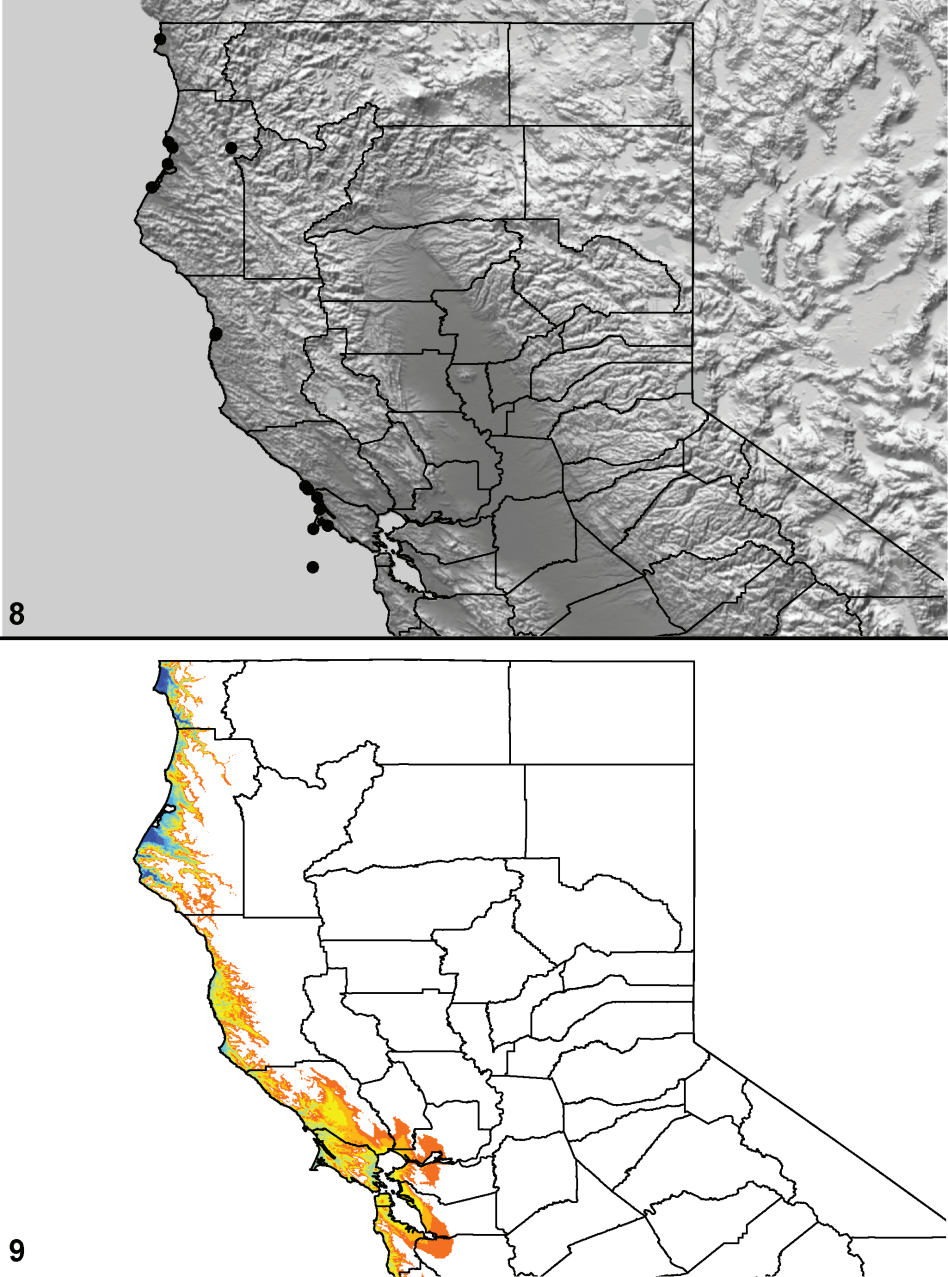
*Aptostichus miwok* Bond 2008. **8** distribution of known specimens **9** predicted distribution; cooler colors–blue shades–represent areas of high probability of occurrence, warmer colors–yellow and orange shades–represent areas of low probability of occurrence.

*Aptostichus miwok* is distributed throughout the geographically disjunct coastal dune habitats of Del Norte, Humboldt, Mendocino, Sonoma, Marin, and San Francisco counties of central and northern California (Map 8). A single inland specimen (Humboldt) is placed with this species on the basis of the molecular data. The DM for *Aptostichus miwok* (Map 9) corresponds closely to the known occurrences with the largest areas of high probability of occurrence in the more northern extent of the species’ distribution. The model indicates that inland localities may be suitable despite the paucity of known inland populations. The habitat requirements and general burrow structure is very similar to that described for *Aptostichus stephencolberti*. All populations are located in the coastal dune habitat. Males have been collected in the months of September, November, January, March, and April.

#### Conservation status.

*Aptostichus miwok* is abundant and widespread across its distribution and thus is likely classified as secure.

#### Species concept applied.

Cohesion. *Aptostichus miwok* is considered to be ecological non-interchangeable from other closely related lineages–it is restricted to coastal dune habitat and has psammophilic features (e.g., lighter coloration).

#### Remarks.

The recognition of *Aptostichus miwok* by Bond and Stockman (2008) as a species renders one of the *Aptostichus stanfordianus* lineages paraphyletic (Fig. 28). While this is somewhat problematic, I have chosen to uphold this decision because of the adaptive diversity contained within the nominal *Aptostichus miwok* lineage and apparent lack of both genetic and ecological interchangeability between this lineage and *Aptostichus stanfordianus*.

### Aptostichus stanfordianus

Smith, 1908

‘The Stanford Hills Trapdoor Spider’

urn:lsid:zoobank.org:act:98C5560A-3053-4810-AD17-ACD95541015A

http://species-id.net/wiki/Aptostichus_stanfordianus

Figures 106 –114 Map 10, 11

Aptostichus stanfordianus Smith, 1908: 221. Female holotype, specimen no. 102 deposited in the Entomological Museum of Stanford University, presumed lost.

#### Type material.

Specimens described by Smith (1908) and deposited in the “Entomological Museum of Stanford University” have long been considered to be lost (e.g., Coyle 1974). To clarify the taxonomic status of the species, I designate herein a neotype because the name-bearing type specimen is believed to no longer exist. Female neotype(MY3248; EU570032, EU569895) designated from California, San Mateo Co., Hwy 92, 1.5km E of HWY 1, 37.48452, -122.39926^1^, elev. 76m, coll. A. Stockman 2.vi.05, deposited in AUMNH. Based on the evidence, Smith’s (1908) drawings and documentation of locality data for the species, I am reasonably confident that designation of this specimen as the neotype is consistent with what is known of the former name-bearing type. The locality from which the neotype was collected is ~20km to the west of Smith’s type locality, described as “Stanford Estate” near Stanford University; considerable development has occurred in this region over the last century and specimens from the direct vicinity of Stanford University are not available. Smith noted collecting specimens, for the series on which *Aptostichus stanfordianus* was described, from points to the west below ~121m in elevation. The carapace length and width for the neotype are noted in the variation section below (enclosed in brackets [ ]) and a complete set of measurements are archived in the Dryad Data Repository at doi: 10.5061/dryad.3b95n; specimen coloration is similar to that illustrated in figure 110. As noted above, GenBank accession numbers EU570032 (12S-tRNA-val-16S) and EU569895 (18S (partial)-ITS1-5.8S-ITS2) are attributed to this specimen.

#### Diagnosis.

**Figures 106–112.**
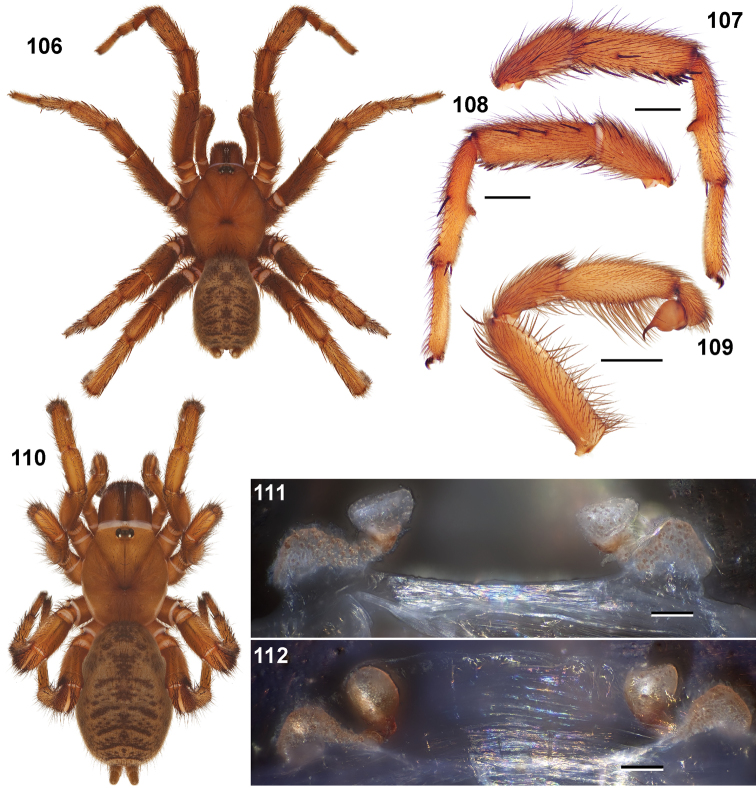
*Aptostichus stanfordianus* Smith, 1908.**106–109** male specimen from Contra Costa Co., Orinda Village (AP162); scale bars = 1.0mm **106** male habitus [805794] **107** retrolateral aspect, right leg I [805784] **108** prolateral aspect, right leg I [805780] **109** retrolateral aspect, right pedipalp [805786] **110, 111** female habitus and cleared spermathecae from Santa Cruz Co. (MY3100) [805790, 805795] **112** cleared spermathecae from Alameda Co., Alameda (AP296) [805776]; scale bars = 0.1mm.

**Figures 113, 114.**
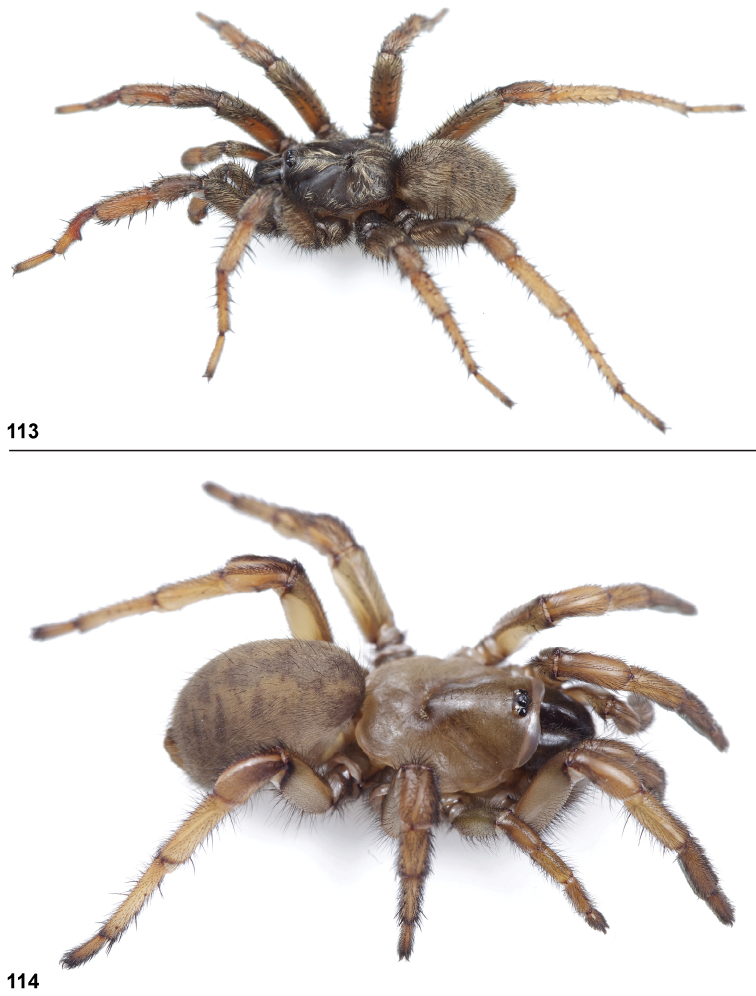
*Aptostichus stanfordianus* specimens from Sonoma Co., Santa Rosa, live photographs. **113** male specimen (AUMS32) **114** female specimen (AUMS35).

Individuals of this species are particularly difficult to distinguish morphologically (Figs 106–112) from *Aptostichus angelinajolieae* specimens but were diagnosed on the basis of a unique set of mtDNA nucleotide substitutions documented in Bond and Stockman (2008; but see discussion below). The species is separated geographically from *Aptostichus angelinajolieae* by the Salinas River Valley and is found only in inland habitats whereas *Aptostichus miwok* is found in coastal dunes. *Aptostichus miwok* is much lighter in color than *Aptostichus stanfordianus* (Figs 106, 110, 113, 114).

#### Description.

Female described by Smith (1908).

#### Material examined.

**United States: California: Alameda Co.:** Alameda, 37.7652, -122.2416^6^, 4m, E Kools 1.i.94 [AP1277, 1♀, CAS]; Berkeley, 37.8776, -122.266^6^, 87m, 21.x.53 [AP251, 1♀, CAS]; J Doyen 10.x.72 [AP296, 1♀, AMNH]; Del Valle Regional Park, 37.5889, -121.6959^5^, 249m, M Thompson, 1.xi.81 [AP609, 1♀, CAS]; Lake del Valle St Recreational Area, Campsite #53, 37.5722, -121.69^1^, 218m, A Stockman 12.v.05 [MY3091, 1juv, AUMNH]; **Colusa Co.:** Bear Valley Rd, 3.2km NW intersection hwy 20, 39.0204, -122.3897^1^, 396m, J Bond, M Hedin 12.i.02 [MY282-84, 3juv, AUMNH]; **Contra Costa Co.:** 0.8km E of S Gate of Mount Diablo State Park, 37.853, -121.9291^5^, 532m, P Hird 7.ii.47 [AP171, 1♂, AMNH]; Orinda Village, San Pablo Ridge below Eureka Peak, 37.8801, -122.2102^5^, 335m, E Schlinger 13.xi.70 [AP162, 1♂, CAS]; Tilden Park, 37.9024, -122.2471^3^, 325m, J Fraser 24.x.80 [AP501, 1♂, AMNH]; Briones Rd, near Bear Creek Rd, 37.9693, -122.1571^1^, 134m, A Stockman 15.vi.05 [MY3371, 1♀, AUMNH]; Morgan Territory Rd, 3.5km S Marsh Creek Rd, 37.8794, -121.8653^1^, 235m, A Stockman 12.v.05 [MY3085, 3086, 2♀, AUMNH]; Briones Regional Park, Alhambra Creek Staging Creek, 37.9537, -122.1250^1^, 120m, M Hedin, J Starrett, D Carlson, R Keith, H Wood, J Ledford 31.iii.2010 [MY3815, 1♀, AUMNH]; **Fresno Co.:** Hwy 198, 35.2km E HWY 25, 36.0962, -120.5249^1^, 464m, A Stockman 9.vi.05 [MY3305, 1♀, AUMNH]; **Marin Co.:** Kentfield, 37.9533, -122.5578^6^, 69m, B Hopey 19.vi.36 [AP271, 1♀, AMNH]; Ridge between San Anselmo & San Rafael, 37.994, -122.558^5^, 180m, L Freihofer 13.xi.76 [AP160, 1♂, CAS]; Tamalpais St Park, 37.9231, -122.5958^5^, 750m, M Bentzien 18.xi.68 [AP1275, 1♀, SCW]; N San Pedro Rd at Pt San Pedro Rd, 37.9974, -122.4571^1^, 20m, A Stockman 15.vi.05 [MY3357, 1juv, AUMNH; MY3358, 1♀, CAS]; **Merced Co.:** Dinosaur Pt Rd, 0.8km SE HWY 152, 37.0649, -121.2102^1^, 412m, A Stockman 7.vi.05 [MY3284, 1juv, AUMNH]; Dinosaur Pt Rd, 2.7km SE of Hwy 152, 37.067, -121.1941^1^ 305m, A Stockman 7.vi.05 [MY3279, 1juv, AUMNH]; **Monterey Co.:** Hwy 198, 10.9km E Hwy 25, 36.1988, -120.7381^1^, 696m, A Stockman 9.vi.05 [MY3308, 1♀, 12juv, AUMNH]; Maher Rd, ~2.4km S San Miguel Canyon Rd, 36.8333, -121.6761^1^, 52m, A Stockman 11.vi.05 [MY3325-27, 3♀, AUMNH]; San Juan Canyon S Salinas, 36.8307, -121.5377^1^, 102m, J Bond 8.xii.05 [MY3464, 3465, 2♀, AUMNH]; **Napa Co.:** Chiles Pope Valley Rd, just S Pope Valley, 38.5963, -122.3997^1^, 250m, M Hedin, J Ledford 31.i.08 [MY3639, 1juv, AUMNH]; Hwy 121, 1.9km S jct w/Hwy 128 at Moskowite Corners, along Capel Creek, 38.432, -122.2073^1^, 250m, M Hedin, J Ledford 31.i.08 [MY3640, 1♂, AUMNH]; N side Howell Mountain, 38.5998, -122.4392^5^, 235m, H Leech 3.xi.83 [AP414, 1♂, CAS]; Hwy 128, 3.2km W Monticello Dam, 38.4952, -122.1236^1^, 143m, A Stockman 13.vi.05 [MY3342, 1♀, AUMNH]; **San Benito Co.:** ~2.25km from Pinnacles Natl Monument entrance, 36.4872, -121.2233^1^, 502m, J Bond 7.v.97 [AP779-81, 2♀, 1juv, AUMNH]; ~2km before Pinnacles Natl Monument entrance, 36.4544, -121.2219^1^, 470m, J Bond 3.iv.96 [AP717, 738, 1♀, 1juv, AUMNH; AP734, 1♀, CAS]; ~6km from Pinnacles Natl Monument entrance Hwy 146, 36.4323, -121.2275^1^, 4m, J Bond, W Bond 27.vii.08 [MY3740, 1♀, AUMNH]; Pinnacles Natl Monument, 36.4872, -121.2066^1^, 475m, J Bond 3.iv.96 [AP731, 1juv, AUMNH]; Pinnacles Natl Monument, ~2.25km from entrance, 36.4873, -121.2233^1^, 475m, J Bond 3.iv.96 [AP747, 1♀, AUMNH]; Pinnacles Natl Monument, W Chaparral ranger station, 36.4893, -121.2119^1^, 439.9m, J Starrett 1.vii.03 [MY2272, 1♀, AUMNH]; San Juan Canyon Rd/G1, 17km S Hwy 156, near Fremont Peak SP entrance, 36.7602, -121.502^4^, 850m, A Stockman 7.vi.05 [MY3291, 1♀, AUMNH]; San Juan Canyon Rd vic. Fremont Peak St Park, 36.7858, -121.4607^1^, 549m, M Hedin, P Paquin, J Starrett 5.iv.03 [MY731, 1♀, AUMNH]; Hwy 25, N Hwy 146, ~2.4km N Gloria Rd, 36.5796, -121.1906^1^, 398m, A Stockman 8.v.05 [MY3301, 1♀, AUMNH]; San Juan Canyon Rd/G1, 3.2km S Hwy 156, 36.8166, -121.5264^1^, 130m, A Stockman 8.vi.05 [MY3295-97, 2♀ 1 juv., AUMNH]; San Juan Canyon Rd/G1, 12.2km S Hwy 156, 36.7852, -121.4632^1^, 566m, A Stockman 8.vi.05 [MY3292, 3294 2juv, AUMNH; MY3293, 1♀, AMNH]; vic Hollister Hills, S of Hollister, 36.7716, -121.4112^1^, 220m, M Hedin, J Starrett, D Leavitt, D Carlson, B Keith 27.iii.2011 [MY3795, 4012, 2♀, AUMNH]; New Idria Rd, Griswold Hills, Griswold Canyon, 36.5417, -120.8342^1^, 420m, M Hedin, J Starrett, D Leavitt, D Carlson, B Keith 28.iii.2011 [MY4010, 1♀, AUMNH]; New Idria Rd, 0.8km N New Idria mine site, 36.4235, -120.6674^1^, 725m, M Hedin, J Starrett, D Leavitt, D Carlson, B Keith 29.iii.2011 [MY4003, 1♀, AUMNH]; Panoche Rd, E Panoche Pass, W Llanada, 36.6190, -120.9771^1^, 40m, M Hedin, J Starrett, D Leavitt, D Carlson, B Keith 28.iii.2011 [MY3791, 4008, 4011, 2♀, 1juv, AUMNH]; **San Francisco Co.:** San Francisco, 37.75, -122.45^7^, 230m, R Schick 1.ix.61 [AP166, 1♂, AMNH]; 23.i.34 [AP473, 1♀, AMNH]; San Joaquin: 6mi W Tracy, 37.7426, -121.5566^5^, 80m, J McSwain 30.iii.49 [AP154, 1♂, AMNH]; **San Mateo Co.:** ~1.6km W Woodside city limit, 37.4416, -122.2416^1^, 88m, J Bond 3.v.97 [AP765-68, 1♀, 3juv, AUMNH]; Alpine Rd, 4km E Pescadero Rd, 37.291, -122.2294^1^, 314m, A Stockman 13.v.05 [MY3107-09, 3♀, AUMNH]; Half Moon Bay, 37.4611, -122.440^5^, 17m, W Lange 21.ii.40 [AP444, 1♀, AMNH]; Highway 84 on way to La Honda, 37.3988, -122.2594^1^, 257m, J Bond 4.v.97 [AP770-71, 2juv, AUMNH]; Hillsborough, swimming pool along Summit Dr, 37.5728, -122.3854^5^, 146m, B Thompson 25.ix.72 [AP159, 1♂, CAS]; Hwy 92, 1.5km E HWY 1, 37.4845, -122.3992^1^, 76m, A Stockman 2.vi.05 [MY3247-49, 3♀, AUMNH]; San Mateo, 37.5568, -122.3177^7^, 60m, 31.xii.38 [AP474, 1♀, AMNH]; **Santa Clara Co.:** 14.5km W Morgan Hill, 1.6km Loma Prieta mountain peak, along Casa Loma Rd, Llagas Creek ravine, 37.1427, -121.7812^4^, 250m, W Icenogle 10.x.70 [AP273, 1♀, 164juv, CAS]; Alum Rock St Park, 37.397, -121.797^3^, 200m, W Icenogle 11.x.70 [AP187, 1♀, 59juv. AMNH]; 8.x.78 [AP261, 1♀, CAS]; 11.xi.70 [AP264, 1♀, 14juv, AMNH]; 8.x.70 [AP478, 1♀, 32juv, CAS]; 30.vii.71 [AP488, 1♂, AMNH]; Bear Creek Rd, ~0.4km W jct w/ hwy 17, 37.1887, -121.9954^1^, 213m, M Hedin, P Paquin, J Starrett 5.iv.03 [MY725, 1♀, AUMNH]; Guadalupe Creek, 37.1777, -121.87479^3^, 228m, D Ubick 17.ii.76 [AP448, 1juv, CAS]; Marsh Rd, ~0.8km S Calaveras Reservoir, ~6.4km E Milpitas, 37.4501, -121.8138^5^, 240m, W Icenogle 7.x.70 [AP199, 1♀, 1juv, CAS; AP254, 1♀, 52juv, AUMNH]; Marsh Rd, 0.8km S Calaveras Reservoir, 1.6km NE intersection Marsh & Felter Rds, 37.4468, -121.8099^4^, 274m, W Icenogle 7.x.70 [AP466, 1♀, 87juv, AUMNH]; Congress Springs Rd/Hwy 9, 37.2609, -122.0985^1^, 492m, A Stockman 13.v.05 [MY3095-99, 3♀, 2juv, AUMNH]; East Dunne Rd, 37.1519, -121.5865^1^, 226m, A Stockman, P Marek 26.i.06 [MY3486, 1juv, AUMNH; MY3487, 1♀, CAS]; Leavesley Rd just E Roop Rd, 37.0574, -121.5188^1^, 235m, A Stockman, P Marek 24.i.06 [MY3473, 3476-78 2♀, 1 juv., AUMNH]; Uvas Rd/G8, 7.2km S Croy Rd, 37.0599, -121.6778^1^, 126m, A Stockman 11.vi.05 [MY3328-30, 2♀, 1juv, AUMNH]; Alum Rock Park, trail near Quail Hollow bridge, 37.3934, -121.8157^1^, 137m, A Stockman, P Marek 25.i.06 [MY3481-83, 1♀, 2juv, AUMNH]; East Dunne Rd, SW Henry Coe State Park, vic Anderson Lake, 37.1496, -121.5909^1^, 230m, M Hedin, J Starrett, D Carlson 27.iii.2010 [MY3811, 1♀, AUMNH]; **Santa Cruz Co.:** Ben Lomond Area, 37.077, -122.084^3^, 100m, Father Koenig 1.xi.64 [AP170, 1♂, AMNH]; Hwy 9, S Brookdale, 37.1037, -122.1048^1^, 130m, M Hedin, R Keith, S Thomas, J Starrett 11.iii.06 [MY3536, 1juv, AUMNH]; Mt Madonna County Park, along Mt Madonna road, 0.72km from jct Pole Line Rd, 37.0116, -121.7208^1^, 455m, J Bond, M van der Merwe 15.xii.99 [MY705, 706 2♀, AUMNH]; University of California Kresge College, 36.99, -122.06^3^, 227m, M Ramirez 14.x.83 [AP158, 1♂, CAS]; Bear Creek Rd 10km E Hwy 9, 37.1767, -122.063^1^, 445m, A Stockman 4.vi.05 [MY3258, 1♀, AUMNH]; Henry Cowell Redwoods SP, Hwy 9, 1.8km S entrance, 37.0309, -122.0648^1^, 161m, A Stockman 15.v.05 [MY3121, 1♀, AUMNH]; Hwy 35, 3.2km S of Hwy 9, 37.27828, -122.14957^1^, 747m, A. Stockman 13.v.05 [MY3100, 1♀, AUMNH]; Swanton Rd, 6.3km from S entrance off hwy 1, S Swanton, 37.0644, -122.2277^1^, 17m, J Bond, A Stockman, D Beamer 17.iii.05 [MY3066, 3067 1♀, 1juv, AUMNH]; **Sonoma Co.:** Bennett Valley Rd, 4.3km W Sonoma Mountain Rd, 38.4163, -122.661^1^, 94m, A Stockman 14.vi.05 [MY3353, 1juv, AUMNH]; Mark W Springs Rd, 7.2km E Old Redwood Hwy, 38.5401, -122.7209^1^, 129m, A Stockman 14.vi.05 [MY3352, 1juv, AUMNH]; **Stanislaus Co.:** Del Puerto Canyon Rd, 22.8km W I-5, 37.4245, -121.3425^1^, 304m, A Stockman 6.vi.05 [MY3275, 1juv, AUMNH]; Del Puerto Canyon Rd, 5.9km W I-5, 37.47373, -121.236^1^, 101m, A Stockman 6.vi.05 [MY3267, 3268 1♀, 1juv, AUMNH].

**Variation, males (5).** Cl 5.50-6.88, 6.18±0.28; Cw 4.50-5.94, 5.16±0.25; STRl 3.03-3.60, 3.39±0.13; STRw 2.54-3.19, 2.83±0.12; LBw 0.83-1.11, 0.93±0.05; LBl 0.41-0.60, 0.53±0.04; leg I: 5.00-6.38, 5.55±0.27; 3.25-4.38, 3.72±0.21; 3.28-4.44, 3.81±0.21; 2.16-3.00, 2.50±0.15; 1.98-2.45, 2.28±0.10; leg IV: 5.00-6.31, 5.65±0.28; 2.63-3.44, 2.94±0.14; PTl 2.37-2.88, 2.61±0.11; PTw 0.80-1.00, 0.89±0.04; Bl 1.07-1.34, 1.22±0.05; TSp 3-5, 3.6±0.4; TSr 0-5, 3.00±0.84; TSrd 4-6, 4.80±0.37.

**Variation, females (5).** Cl 6.38-8.63, 7.27±0.37 [6.88]; Cw 5.25-6.40, 5.82±0.25 [5.75]; STRl 3.50-5.31, 4.19±0.31; STRw 3.01-4.50, 3.64±0.25; LBw 1.19-1.38, 1.25±0.03; LBl 0.62-0.93, 0.79±0.05; Leg I: 14.00-19.88, 16.44±0.98; ANTd 6-9, 8.20±0.58; PTLs 14-24, 19.40±1.60; TBs 2-6, 3.80±0.66.

#### GenBank accessions.

16S-tRNAval-12S: EU569898-EU569900, EU569905, EU569923-EU569926, EU569959-EU569963, EU569992-EU570007, EU570016-EU570018, EU570032-EU570037, JX103401-JX103421; 18S (partial)-ITS1-5.8S-ITS2: EU569884-EU569887; EU569895, EU569896.

#### Distribution and natural history.

**Maps 10, 11.**
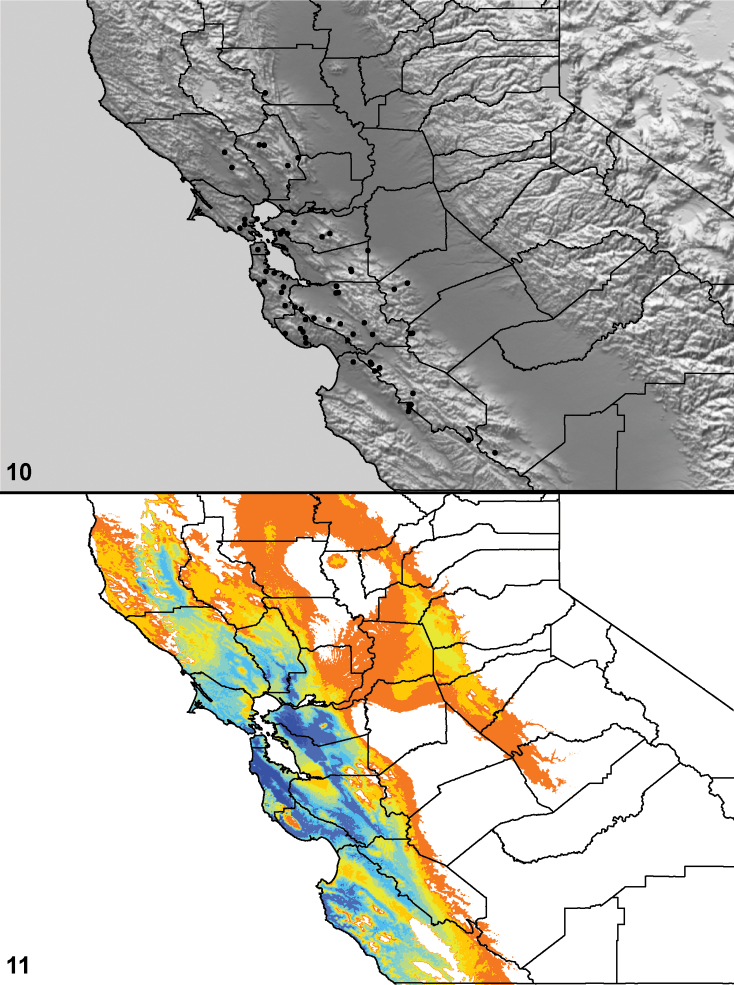
*Aptostichus stanfordianus* Smith, 1908. **10** distribution of known specimens **11** predicted distribution; cooler colors–blue shades–represent areas of high probability of occurrence, warmer colors–yellow and orange shades–represent areas of low probability of occurrence.

*Aptostichus stanfordianus* is distributed widely throughout the Coastal Ranges of central California, bounded to the east by the Central Valley (Map 10). The greatest concentration of populations appears to be centered in the Santa Cruz Mountains and the Diablo and Gabilan Ranges with additional populations in the Sonoma and Howell Mountains to the north. The DM for *Aptostichus stanfordianus* (Map 11) corresponds closely to the known distribution of the species with the exception of areas of high probability of occurrence that overlaps considerably with the predicted distribution of *Aptostichus angelinajolieae* but likewise is separated from this species by an area of low probability of occurrence across the Salinas Valley. Also, populations found on the eastern facing slopes of the Coastal Range are found in areas of a low probability of occurrence. Like *Aptostichus angelinajolieae*, this species is found along damp, north-facing steep banks and road cuts. Populations of this species are found in a number of ecoregion types including California coastal chaparral and shrub, coastal range open woodland and shrub, coniferous forest, coastal steppe, mixed forest and redwood forest. Males have been collected early fall through late winter (September–March).

#### Conservation status.

*Aptostichus stanfordianus* is widespread and abundant throughout its distribution and thus is likely classified as secure. Nevertheless, its distribution throughout the San Francisco Bay region has likely resulted in its extirpation from many localities; specimens from Smith’s (1908) type locality have not been collected in many years.

#### Species concept applied.

Cohesion (sensu Bond and Stockman 2008). Based on the evidence available at the time, Bond and Stockman (2008) considered the two *Aptostichus stanfordianus* clades to comprise a single ecologically interchangeable species; that is, the two sister lineages (no longer retained as such in our more recent analysis, see comments below) overlapped in their predicted distributions and thus were ecologically equivalent (not adaptively diverged).

#### Remarks.

Although cohesion species concept criteria were applied in the formulation of this species the subsequent addition of molecular data for more populations seems to have clouded the picture. The new data indicate that *Aptostichus stanfordianus*, as currently delineated, may comprise at least two distinct, unrelated lineages, within the *Atomarius* Sibling Species Complex (Fig. 28). As such the current diagnosis and delimitation will require reconsideration in the future if these two lineages remain genealogically exclusive under further scrutiny.

### Aptostichus dantrippi sp. n.

‘The Dan Tripp Trapdoor Spider’

urn:lsid:zoobank.org:act:BF39042D-D9CE-49CA-8B18-17404D5CD777

http://species-id.net/wiki/Aptostichus_dantrippi

**Figures 115–119.**
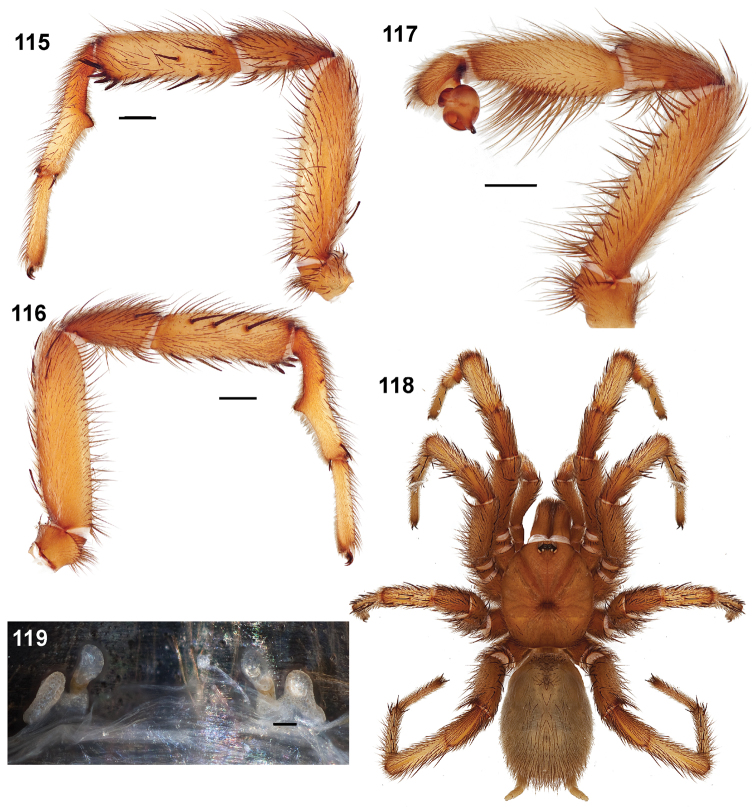
*Aptostichus dantrippi* sp. n. specimens from Kern Co., Bakersfield. **115–118** male holotype (AP179); scale bars = 1.0mm **115** retrolateral aspect, leg I [805816] **116** prolateral aspect, leg I [805820] **117** retrolateral aspect, pedipalp [805822] **118** male habitus [805824] **119** cleared spermathecae of paratype specimen (AP173) [806570]; scale bar = 0.1mm.

**Maps 12, 13.**
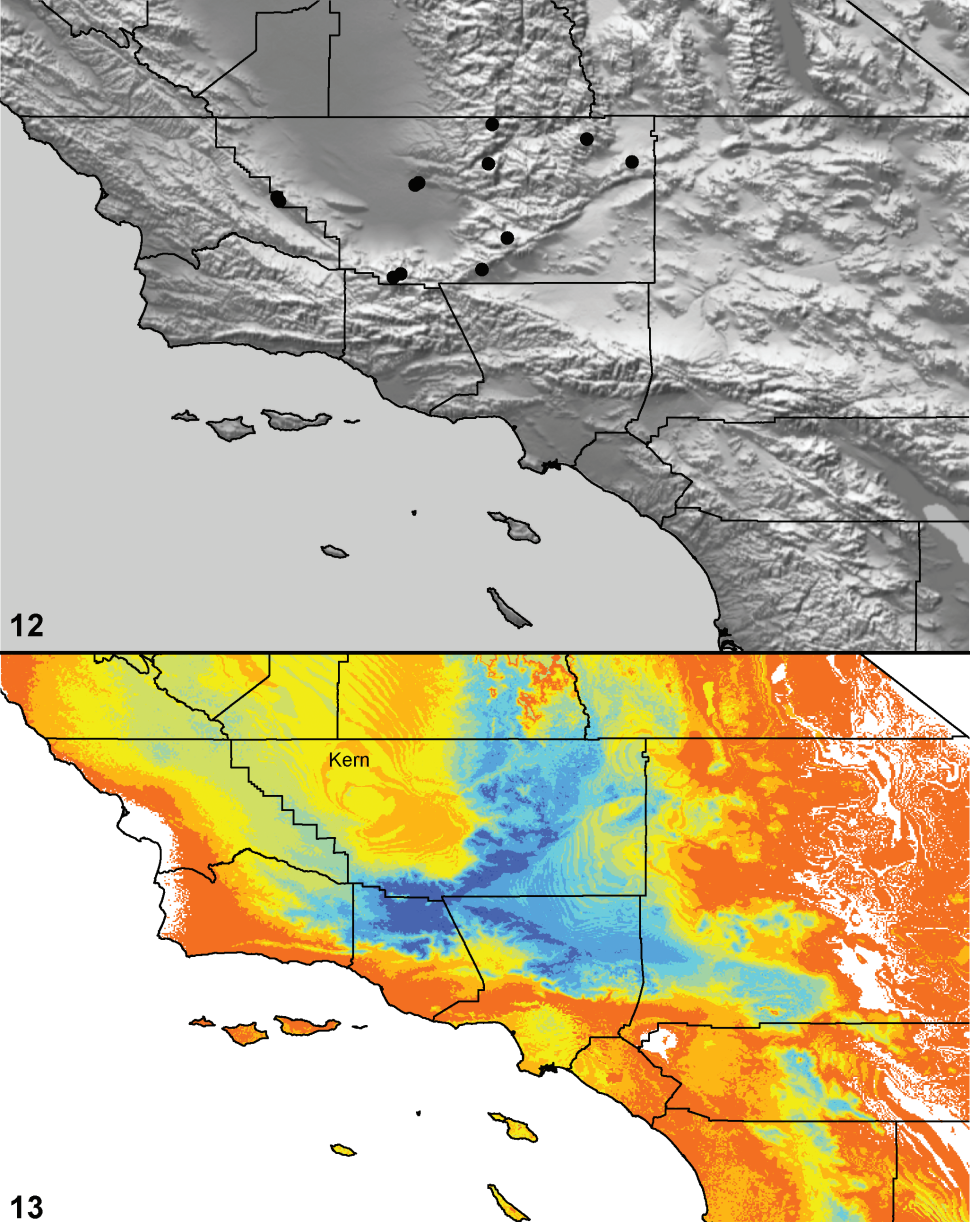
*Aptostichus dantrippi* sp. n. **12** distribution of known specimens **13** predicted distribution; cooler colors–blue shades–represent areas of high probability of occurrence, warmer colors–yellow and orange shades–represent areas of low probability of occurrence.

Figures 115–119 Maps 12, 13

#### Types.

Male holotype (AP179) and female paratype (AP173) from California, Kern Co., Bakersfield, South Bank of Kern River, 35.3947, -119.0313^5^, elev. 137m, coll. W. Icenogle 6.x.1971, deposited in AUMNH.

#### Etymology.

The specific epithet is patronym in honor of Daniel Tripp in recognition of the Tripp family support of biodiversity research and scholarship at East Carolina University, Greenville, North Carolina.

#### Diagnosis.

Morphological differences, particularly secondary sexual characteristics (Figs 115–119), between *Aptostichus dantrippi* and other geographically proximate species of the *Atomarius* Sibling Species Complex are subtle. First, *Aptostichus dantrippi* male and female individuals are found only in far inland habitats but tend to be much lighter in coloration (Fig. 118) than other inland species (*Aptostichus atomarius*, *Aptostichus angelinajolieae*, and *Aptostichus stanfordianus*) and thus superficially resemble coastal dune species *Aptostichus miwok* and *Aptostichus stephencolberti*. Spermathecae have a very elongate lateral lobe that is directed anteriorly whereas the secondary lobe in other taxa is directed more posterior-laterally (Fig. 119). The species is restricted in distribution to Kern County (California) and does not overlap with any closely related *Atomarius* Sibling Species Complex taxa (Map 12).

#### Description of male holotype.

*Specimen preparation and condition*. Specimen collected live from burrow, preserved in 70% ethanol. Coloration moderately faded. Pedipalp, leg I left side removed, stored in vial with specimen. *General coloration*. Carapace, chelicerae, legs dark yellowish brown 10YR 4/6. Abdomen uniform dark reddish gray 5YR 5/2 dorsally. Very light diminutive abdominal color pattern comprising 3 patches along dorsal midline (Fig. 118). *Cephalothorax*. Carapace 6.32 long, 5.44 wide, clothed in light white hairs, stout black bristles along fringe; surface smooth, pars cephalica elevated slightly. Fringe, posterior margin lacks black bristles. Foveal groove deep, straight. Eyes on low mound. AER slightly procurved, PER slightly recurved. PME, AME subequal diameter. Sternum moderately setose, STRl 3.45, STRw 3.00. Posterior sternal sigilla moderate sized, positioned centrally, not contiguous, anterior sigilla pairs small, oval, marginal. Chelicerae with distinct anterior tooth row comprising 6 teeth, posterior margin with single row of small denticles. Palpal endites with patch of small cuspules on proximal, inner margin, labium lacks cuspules, LBw 1.02, LBl 0.42. Rastellum consists of 5 very stout spines not on a mound. *Abdomen*. Setose, sparse heavy black setae intermingled with fine lighter colored setae. *Legs*. Leg I: 6.10, 4.19, 4.13, 2.68, 2.40; leg IV: 6.10, 3.30. Light scopulae on legs I, II tarsus, metatarsus. Tarsus I with single, slightly staggered row of 14 trichobothria. Leg I spination pattern illustrated in Figures 115, 116; TSp 3, TSr 5, TSrd 5. Metatarsus I anteverted retrolaterally, modified with mid-ventral mating apophysis terminating in triangular mound. *Pedipalp*. Articles slender (Fig. 117). PTw 1.40, PTl 2.50, Bl 1.36. Embolus slender, tapering sharply toward tip, slightly curved, lacking serrations (Fig. 117).

**Variation.**Known only from the type specimen.

#### Description of female paratype.

*Specimen preparation and condition*. Female collected live from burrow, prepared in same manner as male holotype. Genital plate removed stored in microvial with specimen. *General coloration*. Carapace, legs, chelicerae, yellowish brown 10YR 5/6. Abdomen uniform dark yellowish brown dorsally 10YR 4/4, ventrum, spinnerets pale yellow; narrow small dusky stripes on dorsal surface. *Cephalothorax*. Carapace 6.90 long, 6.00 wide, glabrous; generally smooth surface, pars cephalica moderately elevated. Fringe lacks setae. Foveal groove deep, slightly procurved. Eye group slightly elevated on low mound. AER slightly procurved, PER slightly recurved. PME-AME subequal diameter. Sternum widest at coxae II/III, moderately setose, STRl 4.20, STRw 3.60. Three pairs of sternal sigilla anterior pairs small, oval, marginal, posterior pair much larger, oval, mesially positioned. Chelicerae anterior tooth row comprising 7 teeth with posterior margin denticle patch. Palpal endites with 10 cuspules concentrated at inner (promargin) posterior heel; labium with 8 cuspules, LBw 1.40, LBl 0.80. Rastellum consists of 8 very stout spines not positioned on mound; fringe of short spines along distal promargin extending upward from rastellum. *Abdomen*. Moderately setose. PLS all 3 segments with spigots. Terminal segment 1/2 length of medial segment, 2 enlarged spigots visible at tip. PMS single segment, with spigots, short with rounded terminus. *Legs*. Anterior two pairs noticeably more slender than posterior pairs. Leg I: 5.60, 3.40, 3.40, 2.80, 1.60. Tarsus I with single staggered row of 19 trichobothria. Legs I, II with moderately heavy scopulae on tarsus, metatarsus. PTLs 14, TBs 3. Rudimentary preening comb on retrolateral distal surface (at tarsus - metatarsus joint) of metatarsus III, IV. *Spermathecae*. 2 simple spermathecal bulbs that lack an elongate neck, basal lateral extension of bulb enlarged, directed anteriorly (Fig. 119).

**Variation (5).** Cl 5.90-7.60, 6.79+0.28; Cw 5.05-6.56, 5.98+0.26; STRl 3.60-4.56, 4.16+0.16; STRw 3.00-3.68, 3.46+0.12; LBw 1.19-1.40, 1.32+0.04; LBl 0.77-0.95, 0.84+0.03; Leg I: 14.50-18.00, 16.69+0.58; ANTd 5-7, 6.20+0.37; PTLs 11-17, 14.60+1.29; TBs 3-5, 3.60+0.40.

#### Material examined.

**United States: California: Kern Co.:** Bakersfield, South Bank Kern River, 35.3947, -119.0313^5^, 137m, W Icenogle 6.x.71 [AP173, 1♀, 10juv, CAS; AP178, 179, 182, 2♀ 1♂ 20juv, AMNH], 16.x.70 [AP175, 1♀, AMNH], 22.vi.70 [AP180, 2♀, CAS], 23.vi.70 [AP545, 1♀, CAS]; Cedar Creek, Cedar Creek Campground, 35.7493, -118.5816^1^, 1495m, F Moore, R Berry 22.v.69 [AP443, 1♀, AMNH]; NE edge El Paso Mountains, hills ~ 1 mile W hwy 395, 35.5288, -117.7636^4^, 900m, W Icenogle 11.x.68 [AP257, 1♀, CAS]; S bank Kern River ~1/4mi from Manor St Bridge, 35.4067, -119.0118^1^, 127m, J Bond 31.iii.96 [AP713, 716, 741, 746 3♀ 1 juv, AUMNH]; Tehachapi Mountains, Antelope Canyon, 34.9005, -118.6406^5^, 1524m, S Frommer 12.xi.75 [AP595, 1♀, CAS]; Tehachapi Mountains, Water Canyon, 35.0848, -118.4934^5^, 1372m, W Icenogle 22.vi.70 [AP194, 2♀, CAS]; San Emigdio Mountain, San Emigdio Creek, NE of Pine Mountain Club, off Mill Potrero Hwy, 34.8624, -119.1275^1^, 1380m, M Hedin, J Satler, D Carlson 27.vii.2010 [MY3808, 3806 2juv, AUMNH]; Breckenridge Rd, 33km E int w Comanche Dr, NE Edison, 35.4843, -118.6477^1^, 1527m, J Satler, S Derkarabetian, C Richart, P van Niekerk 28.iii.2011 [MY3817, 1♀, AUMNH]; Hwy 178, ~100m W of Walker Pass, 35.66343, -118.02767^1^, 1067m, M Hedin, P Paquin, J Starrett 29.iii.2003 [MY0730, 1♀, AUMNH]; **San Luis Obispo.:** Temblor Range, Hwy 58, Pee Wee Park, 35.3494, -119.8174^1^, 900m, M Hedin, J Starrett, D Leavitt, D Carlson, B Keith 29.iii.2010 [MY3792, 1♀, AUMNH]; Hwy 58, Temblor Range, 35.3452, -119.8107^1^, 860m, M Hedin, J Starrett, D Leavitt, D Carlson, B Keith 25.iii.2010 [MY3807 3809, 1juv, 1♀, AUMNH].

#### GenBank accessions.

16S-tRNAval-12S: EU569911, EU569912, JX103294-JX103300.

#### Distribution and natural history.

*Aptostichus dantrippi* as currently defined is distributed primarily throughout Kern County with a few specimens taken from the Temblor Range along the western border with San Luis Obispo County (Map 12). The distribution essentially “rings” the ranges that bound the southernmost extent of the Central Valley and includes the San Emigdio, Tehachapi, and Greenhorn Mountains. The species is mostly restricted to the south valley alluvium and basins, Sierran steppe and mixed and coniferous forests ecoregions of Kern County (Map 12). The only known male specimen was collected during late fall (October). The DM prediction corresponds closely to the known distribution but predicts a high probability of occurrence along the Transverse Ranges. Conversely, the Bakersfield locality along the banks of the Kern River is located in an area of relatively low probability of occurrence; it is likely that this population is relictual given the nature and paucity of the habitat at this location.

#### Conservation status.

The status of *Aptostichus dantrippi* appears to be relatively secure; the species is widespread and abundant. However, the type locality, along the banks of the Kern River, in Bakersfield, California is disturbed and has been highly impacted by proximal development of the last quarter century and thus the species is likely to be locally vulnerable.

#### Species concept applied.

Morphological/phylogenetic. As noted below the molecular data support the hypothesis that these populations constitute a single evolutionary lineage that has subtle morphological differences that distinguish it from sibling species.

#### Remarks.

I have included additional Kern County specimens from the El Paso Mountain and Tehachapi Mountains as part of the *Aptostichus dantrippi* species hypothesis. Specimens from both of these outlying populations appear to be mostly consistent, morphologically, with those specimens collected from the type locality with the exception of the two specimens collected from Water Canyon; they appear to have more prominent abdominal markings and a slightly darker cephalothorax than the type specimens. Molecular data to date obtained from specimens collected just west of the Tehachapi’s corroborate the hypotheses that these populations be included as *Aptostichus dantrippi* despite their somewhat divergence coloration.

### Aptostichus pennjillettei sp. n.

‘The Atomic Penn Jillette Trapdoor Spider’

urn:lsid:zoobank.org:act:812B051A-27F9-4933-89D4-CFB68D22226F

http://species-id.net/wiki/Aptostichus_pennjillettei

Figures 120–126 Map 1

#### Types.

Male holotype (AP413) and paratype (AP412) from Nevada, Clark Co., Mercury, Nuclear Test Site, 37.08906, -116.06187^3^, elev. 1376m, coll. 12.ii.1962 & 3.i.1962, deposited in AMNH.

#### Etymology.

The specific epithet is a patronym in honor of Mr. Penn Jillette, freethinker and advocate of scientific skepticism.

#### Diagnosis.

Males (Fig. 120) can be diagnosed from all known species of *Aptostichus* by having a unique spination pattern on the distal most aspect of tibia I consisting of a few elongate spines (TSrd 3–5) that do not overlap (Figs 121–125). This spination pattern is similar to *Aptostichus atomarius* (Figs 69-75); however, male *Aptostichus pennjillettei* individuals are smaller and much lighter in color (Fig. 120).

#### Description of male holotype.

**Figures 120–126.**
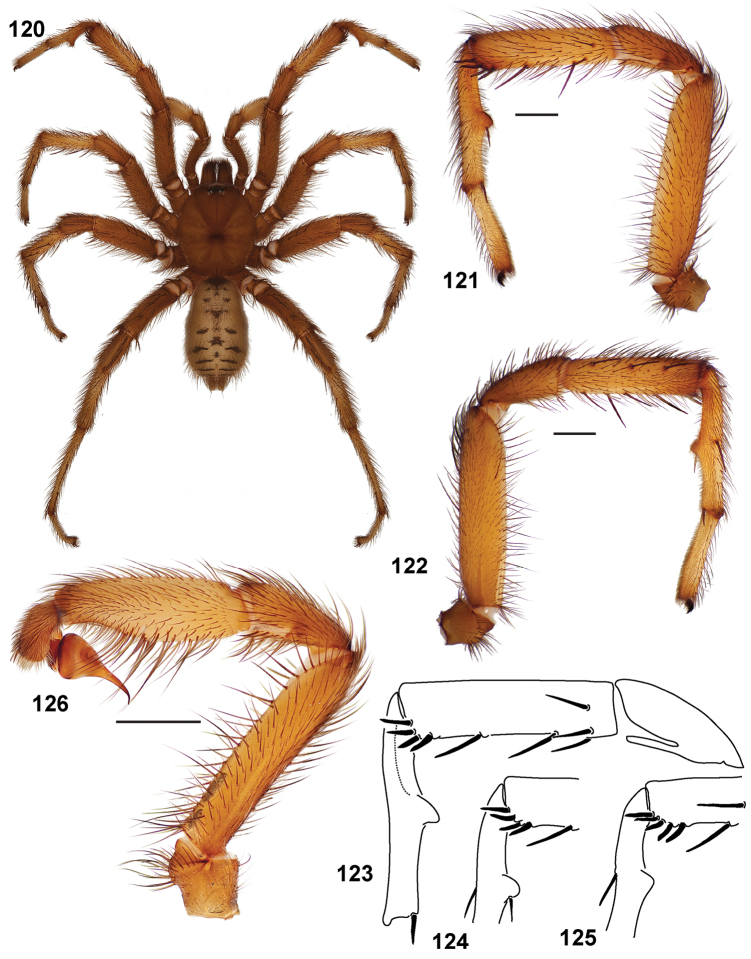
*Aptostichus pennjillettei* sp. n. from Nevada, Clark Co., Mercury, Nuclear Test Site; scale bars = 1.0mm. **120** habitus, male holotype (AP413) [805830] **121–122** male paratype (AP412), leg I **121** retrolateral aspect [805838] **122** prolateral aspect [805834] **123–125** line drawings of spination patterns on leg I, retrolateral aspect, tibia and metatarsus **126** retrolateral aspect, holotype pedipalp [805832].

*Specimen preparation and condition*. Specimen collected from pitfall trap, preserved in 70% ethanol. Coloration presumed to be moderately faded. Pedipalp, leg I left side removed stored in vial with specimen. *General coloration*. Carapace, chelicerae, and legs red 2.5YR 4/6. Abdomen uniform strong brown 7.5YR 4/6 dorsally, ventrally spinnerets same. *Cephalothorax*. Carapace 4.50 long, 4.06 wide, glabrous, stout black bristles along fringe; surface smooth, pars cephalica moderately elevated (Fig. 120). Fringe, posterior margin with black bristles. Foveal groove deep, slightly procurved. Eyes on mound. AER slightly procurved, PER slightly recurved. PME, AME subequal diameter. Sternum moderately setose, STRl 2.79, STRw 2.25. Posterior sternal sigilla intermediate size, positioned centrally, not contiguous, anterior sigilla pairs small, oval, marginal. Chelicerae with distinct anterior tooth row comprising 6 teeth, posterior margin with single row of small denticles. Palpal endites with patch of 10 small cuspules on proximal, inner margin, labium 2 cuspules, LBw 0.78, LBl 0.51. Rastellum consists of 6 very stout spines. *Abdomen*. Setose, black setae intermingled with fine black setae. *Legs*. Leg I: 5.00, 3.60, 3.55, 2.33, 2.02; leg IV: 4.90, 2.50. Light tarsal scopulae on legs I, II. Tarsus I with single, slightly staggered row of 14 trichobothria. Leg I spination pattern illustrated in Figures 121-125; TSp 3, TSr 3, TSrd 3. *Pedipalp*. Articles slender, lacking distinct spines (Fig. 126). PTw 0.77, PTl 2.23, Bl 1.10. Embolus slender, tapering sharply toward tip, lacking serrations (Fig. 126).

**Variation (10).** Cl 4.06-5.81, 4.79±0.19; Cw 3.31-5.00, 3.98±0.19; STRl 2.19-3.54, 2.73±0.14; STRw 1.80-2.58, 2.18±0.09; LBw 0.53-0.98, 0.74±0.04; LBl 0.30-0.53, 0.40±0.03; leg I: 4.06-5.94, 4.85±0.21; 2.94-4.06, 3.43±0.12; 2.50-4.13, 3.26±0.20; 1.92-2.70, 2.22±0.09; 1.35-2.13, 1.75±0.11; leg IV: 3.81-5.88, 4.58±0.23; 2.13-2.88, 2.40±0.10; PTl 1.62-2.61, 2.05±0.13; PTw 0.54-0.86, 0.69±0.04; Bl 0.83-1.26, 1.03±0.05; TSp 3-4, 3.20±0.13; TSr 3-5, 4.20±0.2; TSrd 3-5, 4.10±0.18.

#### Description of female.

Known only from male specimens.

#### Material examined.

**United States: Nevada: Nye Co.**: Mercury Nuclear Testing Site, 37.089, -116.0618^3^, 1376m, BYU-AEC 3.i.62 [AP412, 1♂, AMNH], 12.ii.62 [AP413, 1♂, AMNH], 19.i.61 [AP508, 1♂, AMNH], 2.ii.61 [AP509, 1♂, AMNH], 5.ii.62 [AP510, 1♂, AMNH], 20.ii.61 [AP511, 1♂, AMNH], 9.ii.61 [AP512, 1♂, AMNH], 30.i.61 [AP513, 1♂, AMNH], 20.ii.61 [AP514, 1♂, AMNH], 2.xi.60 [AP515, 1♂, AMNH], 16.i.61 [AP516, 1♂, AMNH]; **Clark Co.**: Lee Canyon, Spring Mountains, 36.3723, -115.6202^5^, 1829m, D Giuliani 10.iii.82 [AP517, 1♂, CAS].

#### Distribution and natural history.

The known distribution of *Aptostichus pennjillettei* is restricted to Mojave Desert localities in Nye and Clark Counties of Nevada. Males appear to disperse during the winter months of November-March.

#### Conservation status.

The conservation status of *Aptostichus pennjillettei* is likely to be imperiled given its very restricted distribution.

#### Species concept applied.

Morphological.

### Aptostichus asmodaeus sp. n.

‘The Demon Trapdoor Spider’

urn:lsid:zoobank.org:act:0BDD8713-6059-482E-A6C1-89FE91F4C234

http://species-id.net/wiki/Aptostichus_asmodaeus

Figures 127–133 Map 1

#### Types.

Male holotype (AP428) and female paratype (AP427), from California, Contra Costa County, Mount Diablo State Park, 37.85309, -121.9291^3^, 532m, coll. W. Icenogle 6-9.vi.74 deposited in AUMNH.

#### Etymology.

The specific epithet is the Latin spelling variation of Asmodeus (a King of Demons), from the Book of Tobias, in reference to the type locality, Mount Diablo State Park.

#### Diagnosis.

**Figures 127–133.**
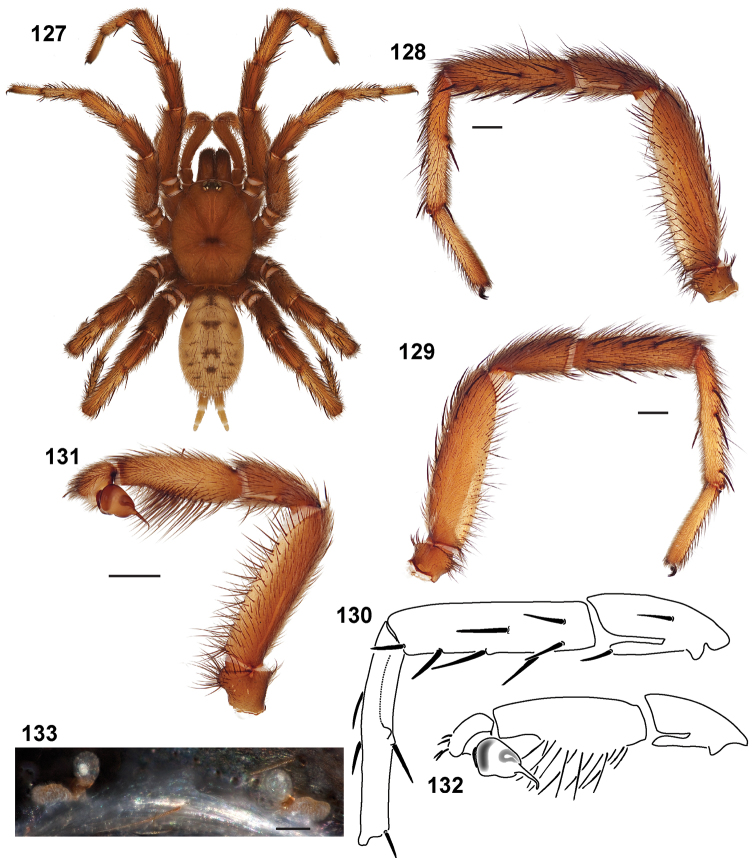
*Aptostichus asmodaeus* sp. n. from Contra Costa Co., Mt. Diablo. **127–132** male holotype (AP428); scale bars = 1.0mm **127** habitus [805850] **128–130** leg I **128** retrolateral aspect [805844] **129** prolateral aspect [805848] **130** line drawing of spination patterns on leg I, retrolateral aspect, tibia and metatarsus **131–132** pedipalp, retrolateral aspect of photograph [805852] and line drawing **133** cleared spermathecae (AP551) [806573]; scale bar = 0.1mm.

Males (Fig. 127) can be distinguished from all known species of *Aptostichus* by having a metatarsal I mating apophysis that forms a distinct knob (Figs 128-130). Females can potentially be recognized by having a large number of labial cuspules, > 8 that tend to form at least two distinctive rows. However, some *Atomarius* Sibling Species Complex individuals also have many labial cuspules but not forming two distinct rows.

#### Description of male holotype.

*Specimen preparation and condition*. Specimen collected from burrow raised to maturity in captivity (matured 24.ix.74), preserved in 70% ethanol. Coloration faded. Pedipalp, leg I left side removed stored in vial with specimen, excuviae. *General coloration*. Carapace, chelicerae, dark red 2.5YR 3/6; legs strong brown 7.5YR 4/6. Abdomen uniform brown 7.5YR 4/4 dorsally; ventrally, spinnerets pale yellow. *Cephalothorax*. Carapace 5.88 long, 4.56 wide, with fine white setae, stout black bristles along fringe; surface smooth, pars cephalica slightly elevated. Fringe, posterior margin with black bristles. Foveal groove deep, recurved slightly. Eyes on low mound. AER slightly procurved, PER slightly recurved. PME, AME subequal diameter. Sternum moderately setose, STRl 3.09, STRw 2.49. Posterior sternal sigilla small, positioned towards margin, not contiguous, anterior sigilla pairs small, oval, at margins. Chelicerae with distinct anterior tooth row comprising 7 teeth, posterior margin with single row of small denticles. Palpal endites with patch of small cuspules on proximal, inner margin, labium lacks distinct cuspules, LBw 0.84, LBl 0.40. Rastellum consists of 5 very stout spines arranged along anterior margin, not on distinct mound. *Abdomen*. Setose, heavy black setae intermingled with fine black setae; light markings (Fig. 120). *Legs*. Leg I: 5.25, 3.63, 4.00, 2.49, 1.98; leg IV: 5.25, 2.90. Very light tarsal scopulae on legs I, II. Tarsus I with single, slightly staggered row of 15 trichobothria. Leg I spination pattern illustrated in Figures 128-130, metatarsal I mating apophysis knob shaped (Figs 128, 130; TSp 7, TSr 6, TSrd 1. *Pedipalp*. Articles relatively slender, lacking distinct spines (Figs 131, 132). PTw 0.74, PTl 2.19, Bl 1.10. Embolus slender, tapering gradually toward tip, lacking serrations (Fig. 131).

**Variation.** Males known only from the holotype specimen.

#### Description of female paratype.

*Specimen preparation and condition*. Female collected live from burrow with eggsac, preserved in same manner as male holotype. Genital plate removed stored in microvial with specimen. *General coloration*. Carapace, legs, chelicerae, strong brown 7.5YR 4/6. Abdomen uniform brown dorsally 7.5YR 5/4, ventral aspect spinnerets pale yellow; dusky mottled stripes dorsal abdomen. *Cephalothorax*. Carapace 6.44 long, 4.94 wide, with fine dark setae; generally smooth surface, pars cephalica moderately elevated. Fringe lacks setae. Foveal groove deep, procurved. Eye group elevated on low mound. AER straight, PER slightly recurved. PME-AME subequal diameter. Sternum widest at coxae II/III, moderately setose, STRl 3.39, STRw 2.88. Three pairs of sternal sigilla anterior pairs small, oval, marginal, posterior pair slightly larger, oval, laterally positioned. Chelicerae anterior tooth row comprising 6 teeth with posterior margin denticle patch. Palpal endites with 22 cuspules concentrated at inner (promargin) posterior heel; labium with 15 cuspules, LBw 1.01, LBl 0.53. Rastellum consists of 7 very stout spines not positioned on a mound; fringe of short spines along distal promargin extending upward from rastellum. *Abdomen*. Moderately setose. PLS all 3 segments with spigots. Terminal segment 1/2 length of medial segment, 2 enlarged spigots visible at tip. PMS single segment, with spigots, short with rounded terminus. *Legs*. Anterior two pairs more slender than posterior pairs. Leg I 14.26 long. Tarsus I with single staggered row of 7 trichobothria. Legs I, II, moderate to heavy scopulae on tarsi and metatarsi. PTLs 12, TBs 3. Distinct preening comb on retrolateral distal surface at tarsus - metatarsus joint of metatarsi III, IV. *Spermathecae*. Intermediate sized stalk, slightly larger terminal bulb; median stalk heavily sclerotized along entire length. Basal extension with well-developed, distinct, basal bulb (Fig. 133).

**Variation (5).** Cl 5.69-6.63, 6.24±0.17; Cw 4.25-5.19, 4.81±0.16; STRl 3.12-3.75, 3.41±0.12; STRw 2.61-2.94, 2.81±0.07; LBw 0.87-1.01, 0.94±0.03; LBl 0.53-0.60, 0.56±0.02; Leg I: 12.95-15.43, 13.96±0.45; ANTd 6-7, 6.20±0.20; PTLs 9-12, 10.80±0.58; TBs 3-4, 3.60±0.24.

#### Material examined.

**United States: California: Contra Costa** **Co.**: 0.80 km E of S Gate Mt Diablo St Park, 37.853, -121.9291^5^, 532m, W Icenogle 6–10.vi.74, [AP427, AP428, AP547-552, 1♂, 5♀, 2juv, AUMNH].

#### Distribution and natural history.

Known only from the type locality (Map 1), Mt. Diablo in the Black Hills, in Contra Costa County. The ecoregion comprises California coastal chaparral forest and shrub habitat.

#### Conservation status.

The conservation status of this species is considered imperiled. Very few specimens have been collected and none over the past quarter century thus the species is rare; however, the type locality is a California State Park, which may afford this geographically restricted species some protection.

#### Species concept applied.

Morphological.

### Aptostichus nateevansi sp. n.

‘The Nate Evans Trapdoor Spider’

urn:lsid:zoobank.org:act:734BF384-2C61-4AAD-8082-0AF36E58DAA7

http://species-id.net/wiki/Aptostichus_nateevansi

Figures 134–141 Map 1

#### Types.

Male holotype (AP420) from California, Los Angeles County, Santa Catalina Island, Toyon Bay, 33.37079, -118.34963^4^, 1m, coll. S. Bennett 1.xi.87, deposited in CAS. Male (AP429) and female (AP421) paratypes from California, Los Angeles County, San Clemente Island, 32.878, -118.46417^4^, 500m, coll. R. Felger, P. Regal 2.v.65 and J. Scott 1.vi.38; deposited in CAS and AMNH respectively.

#### Etymology.

The specific epithet is patronym in honor of Nathaniel Evans in recognition of the Evans family support of biodiversity research at East Carolina University, Greenville, North Carolina.

#### Diagnosis.

**Figures 134–141.**
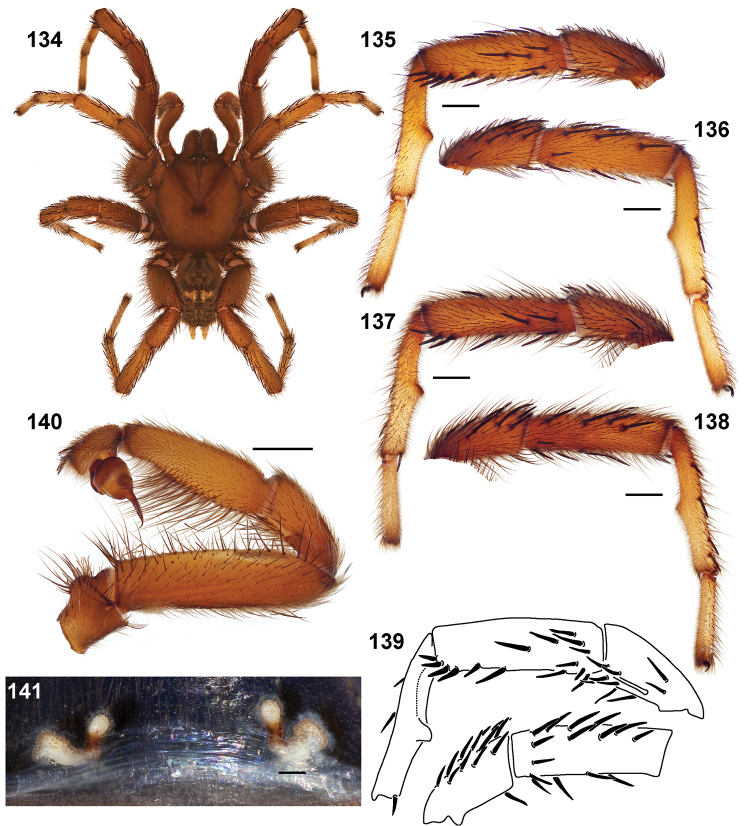
*Aptostichus nateevansi* sp. n. from Los Angeles Co., Santa Catalina Island (holotype) and San Clemente Island (paratype). **134–136, 139, 140** male holotype (AP420); scale bars = 1.0mm **134** habitus [805860] **135** retrolateral aspect, leg I [805854] **136** prolateral aspect, leg I [808858] **137, 138** male paratype (AP429) **137** retrolateral aspect, leg I **138** prolateral aspect, leg I **139** line drawings of leg I spination patterns, retrolateral aspect of patella, tibia and metatarsus and prolateral aspect of patella and tibia **140** retrolateral aspect, pedipalp [805864] **141** cleared spermathecae (AP543) [806576]; scale bar = 0.1mm.

Males (Fig. 134) can be distinguished having a distinctive row of spines on the prolateral surface of patella I (Figs 136, 138, 139). Females can be tentatively distinguished from all species, except some *Aptostichus atomarius* individuals, by having a large number of anterior margin denticles (ANTd= 8).

#### Description of male holotype.

*Specimen preparation and condition*. Specimen preserved in 75% ethanol, coloration faded. Pedipalp, leg I left side removed stored in vial with specimen. *General coloration*. Carapace, chelicerae, legs dark reddish brown 5YR 3/4. Abdomen uniform reddish brown 5YR 4/3 dorsally, ventrum, spinnerets pale yellow. *Cephalothorax*. Carapace 6.44 long, 5.44 wide, hirsute with thin white setae, stout black bristles along fringe; surface smooth, pars cephalica elevated slightly. Fringe, posterior margin with black bristles. Foveal groove deep, moderately straight to slightly recurved. Eyes on low mound. AER straight, PER slightly recurved. PME, AME subequal diameter. Sternum moderately setose, STRl 3.44, STRw 2.75. Posterior sternal sigilla small, heavily sclerotized, positioned towards mid sternum, not contiguous, anterior sigilla pairs small, oval, marginal. Chelicerae with distinct anterior tooth row comprising 8 teeth, posterior margin with single row of small denticles. Palpal endites with patch of small cuspules on proximal, inner margin, labium lacks cuspules, LBw 1.36, LBl 0.68. Rastellum consists of five stout spines arranged along anterior margin. *Abdomen*. Setose, heavy black setae intermingled with fine black setae. *Legs*. Leg I: 5.94, 4.06, 4.06, 2.44, 2.28; leg IV: 5.88, 3.06. Light tarsal scopulae on legs I, II. Tarsus I with single, slightly staggered row of 12 trichobothria. Leg I spination pattern illustrated in Figures 135–139; TSp 8, TSr 7, TSrd 5; numerous spines on patella prolateral surface. *Pedipalp*. Articles slender, lacking distinct spines (Fig. 140). PTw 0.84, PTl 2.70, Bl 1.28. Embolus slender, tapering gradually toward tip, lacks serrations (Fig. 140).

**Variation (2).** Cl 6.31-6.44, Cw 5.19-5.44, STRl 3.29-3.44, STRw 2.75-2.79, LBw 0.95-1.36, LBl 0.65-0.68, leg I: 5.45-5.94, 3.7-4.06, 3.75-4.06, 2.25-2.44, 2.25-2.28; leg IV: 5.35-5.88, 2.75-3.06; PTl 2.50-2.70, PTw 0.84-0.92, Bl 1.28-1.28, TSp 5-8, TSr 4-7, TSrd 5-5.

#### Description of female paratype.

*Specimen preparation and condition*. Female collected from burrow, prepared in same manner as male holotype. Genital plate removed stored in microvial with specimen. *Color*. Carapace, legs, chelicerae, dark reddish brown 2.5YR 2.5/4; abdomen uniform reddish brown dorsally 5YR 4/4, ventral aspect, spinnerets pale yellow. *Cephalothorax*. Carapace 7.39 long, 6.50 wide, lightly hirsute; generally smooth surface, pars cephalica moderately elevated. Fringe lacks setae. Foveal groove deep, procurved. Eye group slightly elevated on low mound. AER slightly procurved, PER slightly recurved. PME-AME subequal diameter. Sternum widest at coxae II/III, moderately setose, STRl 4.50, STRw 3.94. Three pairs of sternal sigilla, anterior pairs small, oval, marginal, posterior pair larger, oval, mesially positioned. Chelicerae anterior tooth row comprising 8 teeth with posterior margin denticle patch. Palpal endites with 48 cuspules concentrated at inner (promargin) posterior heel; labium with 6 cuspules, LBw 1.34, LBl 0.84. Rastellum consists of 5 very stout spines positioned along the anterior margin, not on mound; fringe of short spines along distal promargin extending upward from rastellum. *Abdomen*. Moderately setose. PLS all 3 segments with spigots. Terminal segment 1/2 length of medial segment, 2 enlarged spigots visible at tip. PMS single segment, with spigots, short with rounded terminus. *Legs*. Anterior two pairs noticeably more slender than posterior pairs. Leg I 17.76 long. Tarsus I with single staggered row of 11 trichobothria. Legs I, II with moderately heavy scopulae on tarsi, metatarsi. PTLs 9, TBs 3. Distinct preening combs lacking on legs III, IV. *Spermathecae*. Intermediate sized median stalk with larger terminal bulb; median stalk heavily sclerotized along its entire length. Basal extension well developed as distinct auxiliary bulb (Fig. 141).

**Variation (2).** Cl 7.00-7.39, Cw 5.70-6.50, STRl 4.25-4.50, STRw 3.70-3.94, LBw 1.26-1.34, LBl 0.84-0.85, Leg I: 17.45-17.76, ANTd 8-8, PTLs 9-12, TBs 3-4.

#### Material examined.

**United States: California: Alameda Co.:** Sunol Regional Wilderness Area, 37.5202, -121.8261^3^, 220m, M Thompson 31.xii.1980 [AP610**,** 1♂, AMNH]; **Los Angeles Co.:** Toyon Bay, Santa Catalina Island, 33.3708, -118.3496^4^, 1m, S Bennett 27.x.1979 [AP419, 1♂, CAS], 1.xi.1981 [AP420, 1♂, AMNH]; San Clemente Island, 32.878, -118.4641^6^, 500m, J Scott 1.vi.1938, [AP421, 1♀, AMNH), R Felger, P Regal 2.v.1965 [AP429, 1♂, AMNH], A Menke 14.v.1973, [AP543, 1♀, AMNH), J Doyen 21.v.1972 [AP544, 1juv, CAS].

#### Distribution and natural history.

*Aptostichus nateevansi* is known primarily from Los Angeles County, Channel Islands of Santa Catalina and San Clemente (see comments below regarding the Alameda County specimen). The primary habitat is California coastal chaparral forest and shrub.

#### Conservation status.

Few specimens of this species have ever been collected, and none within the last quarter century; it is known from only a few localities. The Catalina Island Conservancy manages most of Santa Catalina Island and the United States Navy owns San Clemente Island which may afford the species some protection. Nevertheless, I would consider the status of this species likely imperiled due to low abundance and rarity in collections.

#### Species concept applied.

Morphological.

#### Remarks.

Despite the geographical distance that separate the type locality and Alameda County, the anomalous specimen from the Sunol Regional Wilderness area is placed as part of the *Aptostichus nateevansi* species construct because the mating clasper and somatic morphology of the specimen is indistinguishable from those of other specimens. I have little doubt that this is a related, yet disjunct sister species (like *Aptostichus chiricahua*, below), but have chosen to conservatively wait until more specimens are available to set the Alameda County specimen aside as a separate species.

### Aptostichus chiricahua sp. n.

‘The Chiricahua Mountain Trapdoor Spider’

urn:lsid:zoobank.org:act:06C36BAF-E7A6-4D77-B37E-5DE2F815EB27

http://species-id.net/wiki/Aptostichus_chiricahua

**Figures 142–145.**
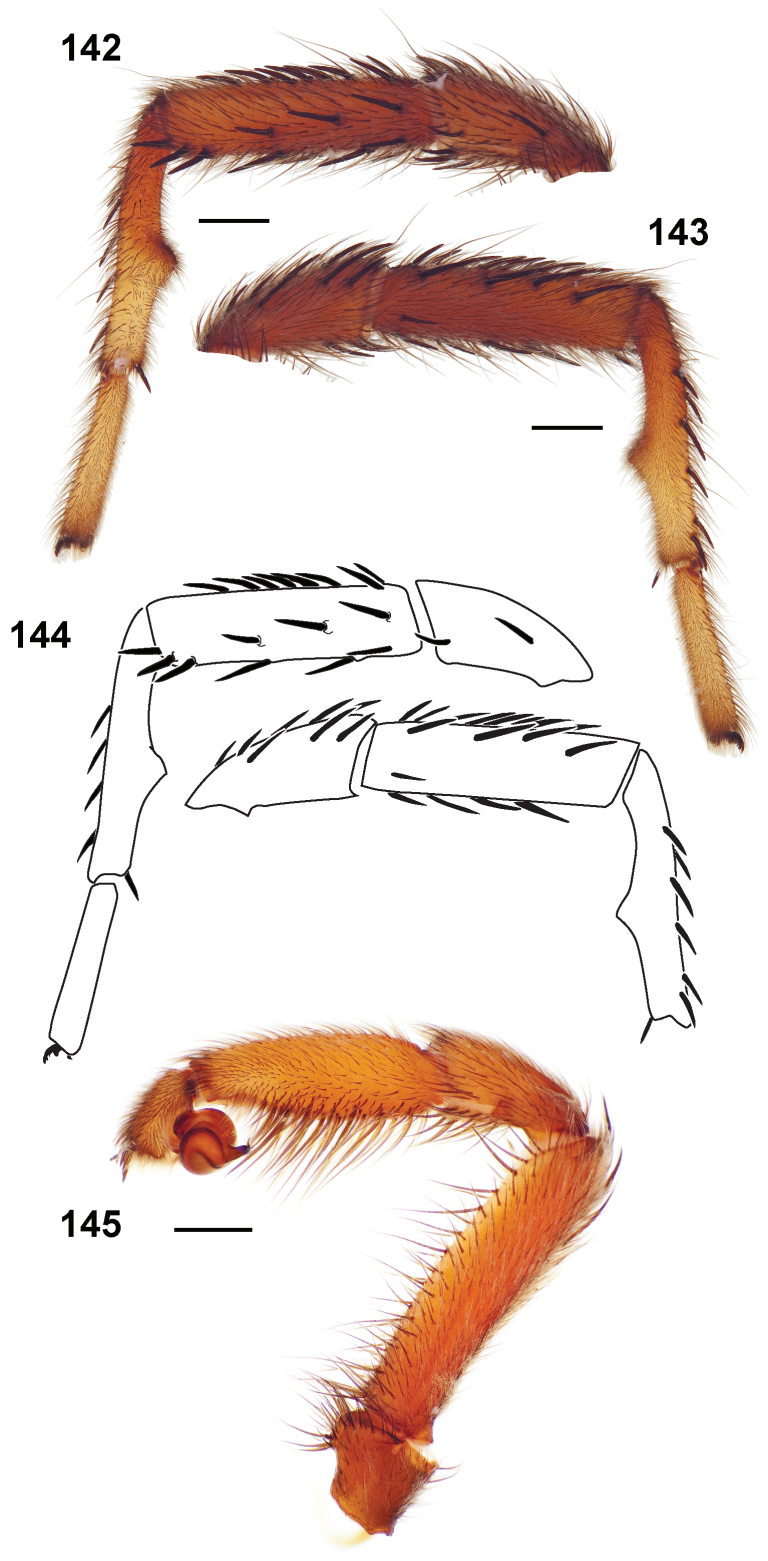
*Aptostichus chiricahua* sp. n. holotype specimen from Arizona, Cochise Co., Portal Arizona; scale bars = 1.0mm. **142–144** leg I **142** retrolateral aspect [806094] **143** prolateral aspect [806090] **144** line drawings of leg I retrolateral and prolateral aspects **145** retrolateral aspect, pedipalp [806096].

Figures 142–145 Map 1

#### Type.

Male holotype (AP645) from Arizona, Cochise County, Portal, 31.91369, -109.1408^1^, 1450m, coll. S. Bennett 12.ix.1980; deposited in AMNH.

#### Etymology.

The specific epithet is a noun taken in apposition from the type locality, the Chiricahua Mountains of southeastern Arizona.

#### Diagnosis.

Males can be diagnosed on the basis of a unique conformation of the tibia leg I, spination pattern which comprises numerous spines on the prolateral and distal surfaces (Figs 142–144). This spination pattern is most similar to the Channel Islands species *Aptostichus nateevansi*, however the *Aptostichus chiricahua* type specimen has considerably more spines, two rows, along the distal, prolateral aspects of the mating clasper tibia. A considerable geographic distance separates *Aptostichus chiricahua* and *Aptostichus nateevansi*. The geographical proximate species, *Aptostichus edwardabbeyi*, has dissimilar mating clasper morphology and has a distinct offset prolateral rastellar spine and thus is a *Hesperus* group species.

#### Description of male holotype.

*Specimen preparation and condition*. Specimen presumably collected live, wandering, preserved in 70% EtOH. Pedipalp, leg I left side removed, stored in vial with specimen; leg IV left side missing. *General coloration*. Carapace, chelicerae, dark reddish brown 2.5YR 2.5/4. Abdomen yellowish red 5YR 4/6, distinct mottled dorsal markings. *Cephalothorax*. Carapace 6.25 long, 5.10 wide, hirsute with intermingled thin white, black setae; stout black bristles along fringe; surface smooth, pars cephalica elevated. Fringe, posterior margin with black bristles. Foveal groove deep, straight. Eyes elevated on high mound. AER slightly procurved, PER strongly recurved. PME, AME subequal diameter. Sternum moderately setose, STRl 3.35, STRw 2.88. Posterior sternal sigilla moderate in size, positioned towards margin, not contiguous, anterior sigilla pairs small, oval, marginal. Chelicerae with distinct anterior tooth row comprising 6 teeth, posterior margin with patch of small denticles. Palpal endites with patch of small cuspules on proximal, inner margin, labium with 2 cuspules, LBw 0.94, LBl 0.65. Rastellum consists of 5 stout spines not on prominent mound. *Abdomen*. Setose, heavy black setae intermingled with fine black setae. *Legs*. Leg I: 5.60, 3.88, 3.80, 2.48, 2.33; leg IV: 5.55, 2.92. Tarsus I, slender, tarsus IV straight. Light tarsal scopulae on all legs, light scopulae on metatarsus I, II. Tarsus I with single, slightly staggered row of 12 trichobothria. Leg I spination pattern illustrated in Figures 142-144 comprising heavy spination on the patella, tibia, metatarsus; TSp 5, TSr 4, TSrd 3. *Pedipalp*. Articles slender, lacking distinct spines (Fig. 145). PTw 0.85, PTl 2.57, Bl 1.11. Embolus broad, tapering sharply toward tip, lacking serrations (Fig. 145).

**Variation.** Known only from the type specimen.

#### Description of female.

Known only from male specimens.

#### Material examined.

Known only from the type material.

#### Distribution and natural history.

*Aptostichus chiricahua* is known only from a single specimen taken from the type locality in Arizona, Cochise Co., Portal (Map 1). Despite extensive collecting efforts in the area female burrows have never been observed. Based on the paucity of specimens, the species may be quite rare.

#### Conservation status.

Undetermined but likely to be imperiled given its restricted distribution and rarity in collections.

#### Species concept applied.

Morphological.

#### Remarks.

As noted above in the description of *Aptostichus nateevansi*, *Aptostichus chiricahua* has a mating clasper that is very similar to the California Channel Island species and thus may be closely related despite the disjunct distribution.

### Aptostichus icenoglei sp. n.

‘The Icenogle Trapdoor Spider’

urn:lsid:zoobank.org:act:DFF124D3-A87E-4981-8FB8-9A340B1688E2

http://species-id.net/wiki/Aptostichus_icenoglei

Figures 146 –156 Maps 14, 15

#### Types.

Male holotype (AP391), female paratype (AP390), and male paratype (AP024) from California, Riverside County, Winchester, 1.6km NW of town center, vicinity of Double Butte, 33.71492, -117.09220^1^, 478m, coll. W. Icenogle; male specimens collected 20.xi.1967 and female 6.vii.1967. Male holotype and female paratype deposited in AUMNH; male paratype in CAS.

**Etymology.** The specific epithet is a patronym in honor of Wendell Icenogle who has collected many of the *Aptostichus* types and has studied this group’s natural history for many years. I am incredibly grateful to Wendell for his assistance, sage advice, patience, and friendship over many years; he has pointed out to me that he was collecting *Aptostichus* before I was born.

#### Diagnosis.

**Figures 146–150.**
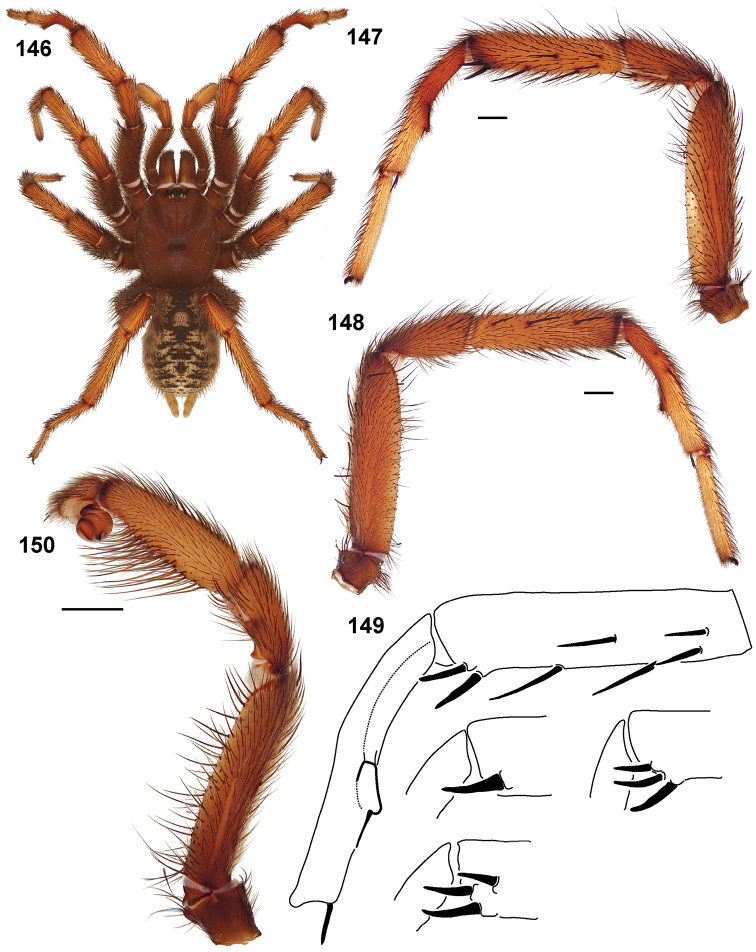
*Aptostichus icenoglei* sp. n.; scale bars = 1.0mm. **146–148, 150** male holotype from Riverside Co., Winchester (AP391) **146** habitus [805874] **147** retrolateral aspect, leg I [805866] **148** prolateral aspect, leg I [805870] **149** line drawings of leg I article spination patterns of holotype and additional specimens from the type locality **150** retrolateral aspect, pedipalp [805872].

**Figures 151–156.**
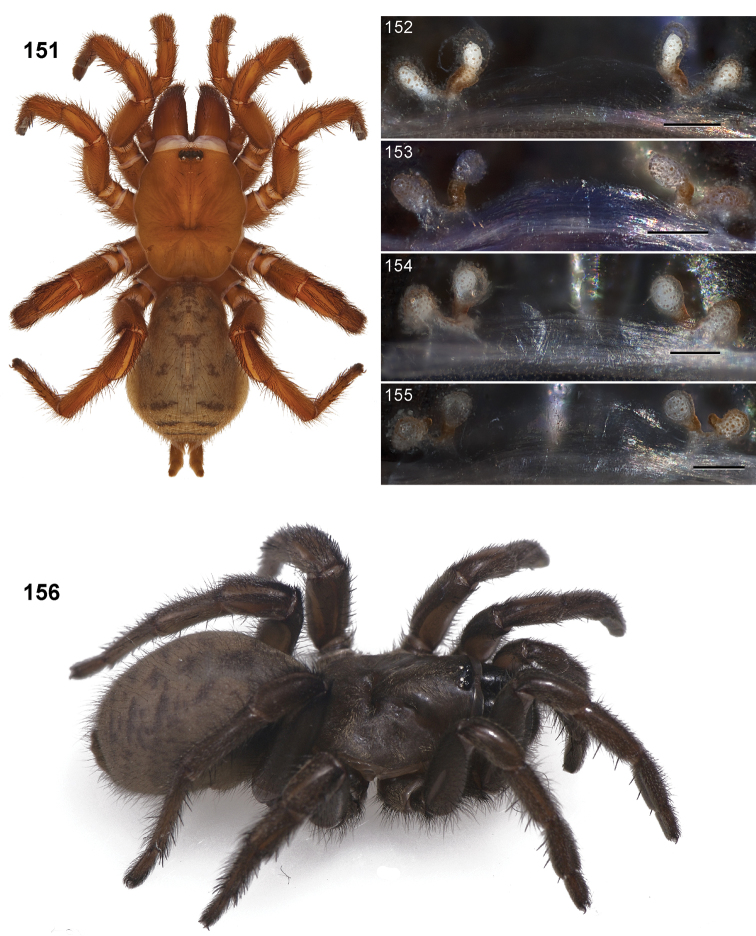
*Aptostichus icenoglei* sp. n. female. **151** habitus, female paratype from Riverside Co., Winchester (AP391) [805876] **152–155** cleared spermathecae of specimens (MY0718, 2465, 2467, 2505) from Riverside and San Diego Counties [806579, 806582, 806585, 806588]; scale bars = 0.1mm. **156** live photograph of specimen (MY3759) from Los Angeles Co.

Males (Fig. 146) can be diagnosed on the basis of a unique conformation of the tibia I mating apophysis and TSrd spination pattern (Figs 147, 149). The *Aptostichus icenoglei* tibial I apophysis (Figs 147, 149) is rectangular in shape and bears a distal spine. In all other *Aptostichus* species the tibial I apophysis is triangular, rounded, or absent, with the exception of *Aptostichus cabrillo* which has a similar rectangular apophysis (Fig. 160). *Aptostichus icenoglei* (Fig. 149) and *Aptostichus cabrillo* (Fig. 160) males can be differentiated on the basis of the TSrd spination pattern. The TSrd of *Aptostichus icenoglei* consists of no more than 3 non–overlapping spines (usually 2), whereas the TSrd of *Aptostichus cabrillo* is always greater than 4 overlapping spines. Female *Aptostichus icenoglei* specimens tend to be darker in coloration, larger (Cw > 5.50) and with fewer prolateral tibial spines on leg III (TBs < 4) than *Aptostichus cabrillo* (Figs 162, 164). Females are distinguished from the sympatric species *Aptostichus hesperus* by lacking contiguous sigilla (see that species’ diagnosis) and *Aptostichus cahuilla* by virtue of its much larger size. Distinguishing female *Aptostichus icenoglei* (Fig. 151) and *Aptostichus atomarius* is problematic. Although their sampled distributions overlap, the sternum width to length ratio tends to be smaller in *Aptostichus icenoglei* (i.e., the sternum of *Aptostichus icenoglei* tends to be more narrow). Additionally, the lateral spermathecal base of *Aptostichus atomarius* is developed into a more distinctive auxiliary bulb that extends below (posteriorly) beyond, the lateral base. The secondary bulb of *Aptostichus icenoglei* is smaller and does not extend below the lateral base (Figs 152–155). As noted in the diagnosis of *Aptostichus atomarius*, *Aptostichus icenoglei* specimens in life tend to be much darker in coloration (Fig. 156).

#### Description of male holotype.

*Specimen preparation and condition*. Specimen collected in pitfall trap, preserved in 70%. Coloration only moderately faded. Pedipalp, leg I left side removed, stored in vial with specimen. *General coloration*. Carapace, chelicerae, legs dark reddish brown 5YR 3/4. Abdomen brown 7.5YR 4/3 with dark distinct chevron striping dorsally (Fig. 146), ventrum and spinnerets pale yellow. *Cephalothorax*. Carapace 5.25 long, 4.70 wide, hirsute with light white setae, stout black bristles along fringe; surface smooth, pars cephalica elevated. Fringe, posterior margin with black bristles. Foveal groove deep, straight. Eyes on low mound. AER slightly procurved, PER slightly recurved. PME, AME subequal diameter. Sternum moderately setose, STRl 3.08, STRw 2.70. Posterior sternal sigilla small, widely separated, anterior sigilla pairs small, oval, marginal. Chelicerae with distinct anterior tooth row comprising 7 teeth, posterior margin with patch of small denticles. Palpal endites with patch of small cuspules on proximal, inner margin, labium lacks cuspules, LBw 0.51, LBl 0.87. Rastellum consists of 5 stout spines not on mound. *Abdomen*. Setose, heavy black setae intermingled with fine black setae. *Legs*. Leg I: 5.63, 4.25, 4.25, 3.00, 2.30; leg IV: 5.80, 3.24. Light scopulae on tarsi, metatarsi legs I, II. Tarsus I with single, slightly staggered row of 12 trichobothria. Leg I spination pattern illustrated in Figures 147-149; TSp 3, TSr 3, TSrd 2. Tibia mating apophysis rectangular. *Pedipalp*. Articles stout, lacking distinct spines (Fig. 150). PTw 0.77, PTl 2.45, Bl 1.11. Embolus slender, tapering sharply toward tip, lacking serrations (Fig. 150).

**Variation (9).** Cl 5.13-5.81, 5.41±0.09; Cw 4.19-4.70, 4.45±0.07; STRl 2.79-3.30, 3.00±0.05; STRw 2.19-2.70, 2.41±0.05; LBw 0.63-0.87, 0.76±0.03; LBl 0.38-0.51, 0.43±0.02; leg I: 4.85-5.81, 5.33±0.1; 3.38-4.56, 3.90±0.12; 3.60-4.25, 3.91±0.07; 2.50-3.06, 2.76±0.06; 2.00-2.61, 2.38±0.07; leg IV: 5.00-5.80, 5.41±0.09; 2.60-3.24, 2.89±0.07; PTl 2.07-2.45, 2.24±0.04; PTw 0.66-0.81, 0.73±0.02; Bl 0.98-1.13, 1.04±0.02; TSp 2-5, 3.11±0.31; TSr 3-6, 4.44±0.34; TSrd 2-3, 2.33±0.17.

#### Description of female paratype.

*Specimen preparation and condition*. Female collected live from burrow, preserved in same manner as male holotype. Genital plate removed, cleared in trypsin, stored in microvial with specimen. *General coloration*. Carapace, legs, chelicerae, red 2.5YR 4/6. Abdomen brown dorsally 7.5YR 4/2 with distinct chevron striping, ventrum and spinnerets pale yellow. *Cephalothorax*. Carapace 7.30 long, 6.50 wide, glabrous; generally smooth surface, lightly hirsute, pars cephalica moderately elevated. Fringe lacks setae. Foveal groove deep, slightly procurved. Eye group slightly elevated on low mound. AER straight, PER slightly recurved. PME-AME subequal diameter. Sternum widest at coxae II/III, moderately setose, STRl 4.50, STRw 3.45. Three pairs of sternal sigilla anterior pairs moderate size, oval, positioned marginally, posterior pair larger, oval, mid-sternal positioned. Chelicerae anterior tooth row comprising 7 teeth with posterior margin denticle patch. Palpal endites with 12 cuspules concentrated at the inner (promargin) posterior heel; labium with 1 cuspule, LBw 1.45, LBl 0.80. Rastellum consists of 6 very stout spines not positioned on mound. *Abdomen*. Moderately setose. PLS all 3 segments with spigots. Terminal segment 1/2 length of medial segment, 2 enlarged spigots visible at tip. PMS single segment, with spigots, short with rounded terminus. *Legs*. Anterior two pairs noticeably more slender than posterior pairs. Leg I 16.20 long. Tarsus I with single staggered row of 16 trichobothria. Legs I, II with moderately heavy scopulae on tarsi, metatarsi, distal aspect tibia. PTLs 8, TBs 2. Distinct preening comb on retrolateral distal surface (at tarsus - metatarsus joint) of metatarsus III, IV. *Spermathecae*. Heavily sclerotized intermediate sized median stalk with well-developed basal extension (Figs 152–155).

**Variation (8).** Cl 6.56-8.80, 7.49±0.23; Cw 5.80-7.13, 6.43±0.15; STRl 3.96-5.05, 4.52±0.13; STRw 3.07-4.10, 3.59±0.11; LBw 1.19-1.45, 1.34±0.03; LBl 0.80-1.19, 0.94±0.05; Leg I: 15.10-18.03, 16.39±0.31; ANTd 6-7, 6.25±0.16; PTLs 8-14, 11±0.6; TBs 2-3, 2.25±0.16.

#### Material examined.

**Mexico: Mexicali: Baja California Norte:** 3.2km W Rancho Grande, 24.1k S San Vicente, 32.4689, -115.3844^5^, 20m, V Roth 8.i.1965 [AP029, 1♂, AMNH]; **United States: California: Los Angeles** **Co.:** Chatsworth, intersection Lassen St & Valley Circle Blvd, ravine just E Oakwood Cemetery, 34.2512, -118.6227^4^, 315m, W Icenogle 16.x.1966 [AP253, 1♀, AMNH]; Chatsworth, Limekiln Canyon Park, 34.2876, -118.5606^4^, 407m W Icenogle 17.i.1971 [AP305, 1♀, 1juv, CAS]; Gabriel Mtns, Angeles Natl Forest, Evey Canyon, Off Brady Rd., 34.1638, -117.685^1^, 763m, J Bond 6.iv.1996 [AP719, 1♀, AUMNH]; Glendora Ridge Rd, San Gabriel Mountains, 34.2213, -117.7391^3^, 1133m, W Icenogle 21.v.1974 [AP017, 1♀, CAS]; Henninger Flats, 34.1926, -118.0875^3^, 792m, 27.x.1967 [AP018, AP297 2♀, 1juv, AMNH], F Coyle 19.vii.1972 [AP642, 1♀, 14juv, AMNH]; Mt Baldy Rd, 0.2km N jct w/ N Mountain Ave, 34.1773, -117.6767^1^, 800m, M Hedin, J Satler, J Starrett, C Richart 15.ii.2009 [MY3759, 1♀, AUMNH]; Mt Washington, Los Angeles, 34.0992, -118.2194^3^, 246m, G Bakker 1.ix.1967 [AP025, 1♂, AMNH]; Pasadena, Eaton Canyon Park, 34.1807, -118.095^4^, 325m, M Thompson 26.xii.1967 [AP049, 1♂, AMNH]; Placerita Canyon Park, 34.37, -118.443^4^, 655m, F Coyle 20.vii.1972 [AP452, 1♀, AMNH]; Puente Hills, intersection Azusa & Tomich Rd, 33.9816, -117.9335^1^, 210m, J Bond, C Spruill, D Beamer 14.iii.2004 [MY2600, 1♀, AUMNH]; San Gabriel Mtns, Angles Natl Forest, hwy 3, 1.3km jct w/Big Tujunga Canyon Rd, 34.2907, -118.1706^1^, 1030m, M Hedin, J Satler, J Starrett, C Richart 19.ii.2009 [MY3780, 1♀, AUMNH]; Santa Ysabel, Santa Ysabel Ecological Reserve, Kanaka Flat, 33.1083, -116.616^1^, 1277m, USGS-BRD San Diego Sta. 1.x.2002 [AP938, 1♂, CAS]; Santa Ysabel, Santa Ysabel Ecological Reserve, Santa Ysabel Creek, 33.133, -116.6484^1^, 1018m, USGS-BRD San Diego Sta. 1.iv.2002 [AP937, 1juv, CAS]; Sierra Madre, 34.1621, -118.0532^3^, 255m, M Thompson 8.ii.1972 [AP048, 1♂, CAS]; **Orange Co.:** Bell Canyon, 33.6178, -117.5627^1^, 252m, USGS-BRD San Diego Sta. 1.ix.1998 [AP1144, 1♂, CAS]; Crow Canyon, 33.6029, -117.5442^1^, 338m, USGS-BRD San Diego Sta. 1.xi.2000 [AP1129, 1♂, CAS], 1.xi.1998 [AP1130, 1♂, CAS]; N View Rancho Santa Margarita, 33.6138, -117.5504^1^, 484m, USGS-BRD San Diego Sta. 1.xi.1998 [AP1140, 1♀, CAS]; Ridge E Bell Canyon, 33.6041, -117.5525^1^, 518m, USGS-BRD San Diego Sta. 1.xi.1998 [AP1139, 1♂, CAS]; Ridge E Bell Canyon, S Fox Canyon, 33.6192, -117.5532^1^, 384m, USGS-BRD San Diego Sta. 1.viii.1999, [AP1141, 1juv, CAS], 1.ix.1998 [AP1142, 1♂, CAS], 1.ix.1999 [AP1143, 1♂, CAS]; Ridge E Fox Canyon, 33.6174, -117.5438^1^, 457m, USGS-BRD San Diego Sta. 1.ix.1998 [AP1135, 1♂, CAS]; Ridge N Crow Canyon, 33.6081, -117.5469^1^, 427m, USGS-BRD San Diego Sta. 1.ix.1998 [AP1132, 1133 2♂, CAS]; Salt Creek 2.4km N Dana Pt, 33.4819, -117.7206^3^, 21m, W Icenogle 6.xii.1968 [AP605, 1♀, 1juv, AMNH]; Santa Ana Mountains, 4.0km E an Juan Fire Station, Hot Spring Canyon Rd turnout, 33.5907, -117.4754^1^, 366m, W Icenogle 7.ix.2000 [AP366, 1♂, CAS]; SE View Rancho Santa Margarita, 33.6113, -117.5465^1^, 480m, USGS-BRD San Diego Sta. 1.ix.1998 [AP1134, 1♂, CAS]; S View Rancho Santa Margarita, 33.6118, -117.5482^1^, 530m, USGS-BRD San Diego Sta. 1.x.1999 [AP1136, 1♂, CAS]; SW View Rancho Santa Margarita, 33.6092, -117.5524^1^, 500m, USGS-BRD San Diego Sta. 1.vii.1998 [AP1137, 1♂, CAS]; Weir Canyon, 33.8299, -117.7391^1^, 252m, USGS-BRD San Diego Field Station 1.iv.2000 [AP1128, 1♂, CAS]; Bell Canyon, 33.6302, -117.5539^1^, 297m, USGS-BRD San Diego Sta. 1.x.1999 [AP1113, 1♂, CAS]; S Aliso Wood Canyon, 33.5185, -117.7386^1^, 7m, USGS-BRD San Diego Sta. 1.ix.2000 [AP975, 1♂, CAS]; **Riverside Co.:** 1.6km NW Winchester town center, 33.7148, -117.0921^1^, 470m, W Icenogle 27.vii.1968 [AP013, 1♀, 100+juv, CAS], 17.viii.1968 [AP186, AP460, 2♀, 180juv, CAS]; 3.2km NW Tenaja Ranger Station, 33.5298, -117.3886^6^, 515m, W Icenogle 19.ix.1968 [AP008, 1♀, 50+juv, AMNH]; 6.4km W Mountain Center, 33.7011, -116.769^3^, 1124m, W Icenogle 28.viii.1968 [AP459, 1♀, 100+juv, CAS]; Bautista Canyon, 33.6978, -116.8519^5^, 669m, K Cooper 19.ii.1978 [AP589, 1juv, UCR]; Bautista Canyon, along Bautista Canyon Rd, 33.7099, -116.8775^1^, 611m, J Bond 1.ii.2004 [MY2480, 1♀, AUMNH]; between Squaw Mountains & Redonda Mesa, N Tenja Rd, 33.5035, -117.3368^1^, 634m, USGS-BRD San Diego Sta. 1.ix.2000 [AP899, 1♂, CAS]; between Squaw Mtn. & Redona Mesa, N Tenaja Rd., 33.5026, -117.3464^1^, 646m, USGS-BRD San Diego Sta. 1.x.1999 [AP872, 873 2♂, CAS]; Cleveland Natl Forest, along H74, 33.6297, -117.4252^2^, 706m, J Bond, C Spruill, D Beamer 18.iii.2004 [MY2668, 2669, 2juv, AUMNH]; De Luz Murrieta Rd, 33.4956, -117.2433^1^, 366m, M Hedin 11.i.2003 [MY0718, 1♀, AUMNH]; E Gavilan Mtn, N Santa Margarita River, 33.4589, -117.172^1^, 820m, USGS-BRD San Diego Sta. 1.ix.1998 [AP891, 1♂, CAS]; E Skinner Reservoir, 33.5898, -117.0233^1^, 452m, USGS-BRD San Diego Sta. 1.viii.1998 [AP1032, 1♂, CAS]; James Reserve Lake Fulmor on hwy 243, 33.8042, -116.7803^3^, 1599m, 26.vii.1997 [AP030, 1♂, CAS]; Joshua Tree Natl Monument, 33.9617, -116.2294^6^, 1400m, 1.iii.1978 [AP583, 1♀, UCR]; Kabian Park, between Canyon Lake & Perris, off Goetz Rd 6.1km S Case & Goetz Rds jct in Perris, 33.7272, -117.2436^1^, 512m, S Kirshtner 8.xi.2003 [AP1231, 1♂, CAS]; Lake Matthews, 33.8266, -117.438^1^, 446m, J Bond 20.xi.1998 [AP691, 1juv, AUMNH]; Lake Skinner, 13.1km NW Winchester, 33.595, -117.058^3^, 485m, 7.xii.1997 [AP044, 1♂, CAS], J Bond 13.xii.1997 [AP676, 1♀, AUMNH]; Menifee Valley, 33.6848, -117.175^1^, 434m, S Frommer 7.vi.1992 [AP011, 1♀, UCR], 12.i.1991 [AP582, 1♀, UCR]; N base Avenaloca Mesa, S Tenaja Rd, 33.500, -117.3292^1^, 692m, USGS-BRD San Diego Field Station 1.ix.2000 [AP965, 1♂, CAS]; N Perris, E Mayer Farms, 33.8045, -117.2518^1^, 571m, USGS-BRD San Diego Sta. 1.ii.1999 [AP1099, 1♂, CAS]; N Perris, E Mayer Farms, 33.8073, -117.2572^1^, 571m, USGS-BRD San Diego Sta. 1.i.2000 [AP1102, 1♂, CAS]; N Tenja Canyon, S Wildomar Rd., 33.5104, -117.3687^1^, 604m, USGS-BRD San Diego Sta. 1.i.2000 [AP875, 1♂, CAS], 1.ix.2000 [AP876, 1♂, CAS]; Ortega hwy H74, 2.7km N Orange Co/Riverside Co line, 33.6127, -117.4346^1^, 523m, J Bond 2.ii.2004 [MY2492, 1juv, AUMNH]; Pigeon Pass, 33.993, -117.276^3^, 584m, W Rose 27.xii.1967 [AP032, 1♂, AMNH]; San Bernardino Natl Forest, Juan Diego Flats Rd, ~1km from intersection w/Reed Valley Rd, 33.5836, -116.8141^1^, 1110m, J Bond 7.iv.1996 [AP718, 1♀, AUMNH]; Santa Margarita Ecological Reserve, Santa Margarita River, E Gavilan Mtn, 33.455, -117.1729^1^, 786m, USGS-BRD San Diego Sta. 1.xi.1998 [AP892, 1♂, CAS]; Santa Margarita Ecological Reserve, S Santa Margarita River, N Royal Oak Ranch, 33.4411, -117.1637^1^, 396m, USGS-BRD San Diego Sta. 1.ix.1999 [AP895, 1♂, CAS]; S Santa Margarita river, N Royal Oak Ranch, 33.4399, -117.1605^1^, 421m, USGS-BRD San Diego Sta. 1.xi.1999 [AP894, 1♂, CAS]; University of California, Riverside Campus, 33.9742, -117.3251^3^, 327m, W Icenogle 5.x.1967 [AP010, 1♀, 100+juv, AUMNH], 13.ix.1967 [AP014, 1♀, 100+juv, CAS], 27.x.1967 [AP188, 1♀, CAS], W De Luz Creek, S Tenaja Rd, 33.505, -117.3138^1^, 674m, USGS-BRD San Diego Field Station 1.ix.1999 [AP988, 1♂, CAS]; W De Luz Creek, S Tenaja Rd, 33.5097, -117.3139^1^, 650m, USGS-BRD San Diego Field Station 1.xii.1999 [AP991, 1♂, CAS]; W De Luz Creek, S Tenaja Rd, 33.5114, -117.3135^1^, 661m, USGS-BRD San Diego Field Sta. 1.ix.2009 [AP989, 990, 2♂, CAS]; Winchester, 33.7138, -117.0915^1^, 470m, W Icenogle 28.ix.1984 [AP035, 1♂, AUMNH]; W Icenogle 22.ix.1985 [AP040, 1♂, FMNH], 1.vi.1986 [AP041, 1♂, AUMNH], 31.xii.1972 [AP046, 1♂, FMNH], 15.i.1991 [AP047, 1♂, CAS], 25.v.1970 [AP656, 1♀, CAS]; Winchester, 1.6km NW of town center, vicinity of Double Butte, 33.7149, -117.0922^1^, 478m, W Icenogle 18.iii.1974 [AP012, 1♀, CAS], 19.vi.1979 [AP022, 1♀, 100+juv, CAS], 28.xii.1972 [AP023, 1♂, CAS], 25.xii.1971 [AP024, 1♂, AMNH], 25.i.1975 [AP027, 1juv, CAS], 12.xi.1971 [AP033, 034 2♂, CAS], 3.xii.1975 [AP036, 1♂, AMNH], 16.xi.1997 [AP039, 1♂, FMNH], 30.ix.1967 [AP042, 1♂, CAS], 5.xi.1985 [AP043, 1♂, AMNH], 25.xi.1967 [AP045, 1♂, AMNH], 6.viii.1967 [AP390, 1♀, CAS], 20.xi.1967 [AP391, 1♂, CAS], 28.v.1967 [AP454, 1♀, AMNH], 6.viii.1967 [AP455, 1♀, 116juv., AMNH]; Winchester, Grand Ave ~1km E intersection Grand & Matthews, 33.7147, -117.11^1^, 460m, J Bond 17.v.2009 [MY3776, 1juv, FMNH; MY3777, 1♀, AUMNH]; Winchester, 1.6km NW of town center, vicinity of Double Butte, 33.7156, -117.0936^1^, 465m, J Bond 29.i.2004 [MY2505, 2512, 2523 3♀, AUMNH]; Winchester, Leona Rd ~1.6km S intersection w/Patton Ave, 33.6771, -117.1157^1^, 444m, J Bond, W Icenogle, et al. 13.iii.2004 [MY2597, 1juv, AUMNH]; between Squaw Mountain & Redonda Mesa, N Tenaja Rd, 33.5025, -117.3386^1^, 1020m, USGS-BRD San Diego Sta. 1.x.1999 [AP967, 1♂, CAS]; N base Avenaloca Mesa, S Tenaja Rd, 33.5, -117.3279^1^, 685m, USGS-BRD San Diego Sta. 1.x.1999 [AP963, 964, 2♂, CAS]; SE Skinner Reservoir, 33.5766, -117.0316^1^, 482m, USGS-BRD San Diego Sta. 1.ii.2000 [AP1022, 1♂, CAS]; S Skinner Reservoir, 33.577, -117.0424^1^, 508m, USGS-BRD San Diego Sta. 1.iv.1999 [AP1021, 1♂, CAS]; W base Mesa de Colorado, W Temecula, 33.5125, -117.3012^1^, 605m, USGS-BRD San Diego Sta. 1.ix.1999 [AP979, 1♂, CAS]; **San Bernardino Co.:** Alta Loma, 34.122, -117.597^3^, 412m, D Bixler 4.iv.1969 [AP021, 1♂, AMNH]; E Silverwood Lake, E fork of W fork Mojave River, 34.2718, -117.297^1^, 1099m, USGS-BRD San Diego Sta. 1.viii.2002 [AP1171, 1♂, CAS]; E Silverwood Lake, E fork of W fork Mojave River, 34.2727, -117.293^1^, 1090m, USGS-BRD San Diego Sta. 1.x.2000 [AP1172, 1♂, CAS], 1.vii.2002 [AP1173, 1♂, CAS]; Silverwood lake, E fork of W fork Mojave River, 34.2777, -117.3169^1^, 1035m, USGS-BRD San Diego Sta. 1.vii.2002 [AP1170, 1♂, CAS]; N Silverwood Lake, 34.303, -117.3346^1^, 1069m, USGS-BRD San Diego Sta. 1.viii.2002 [AP1174, 1♂, CAS]; Silverwood Lake, 34.2935, -117.3005^6^, 1099m, C Brown 5.x.2000 [AP1215, 1216, 1217, 3♂, CAS]; W Silverwood Lake, S of W fork Mojave River, 34.283, -117.3584^1^, 1074m, USGS-BRD San Diego Sta. 1.viii.2002 [AP1167, 1♂, CAS], 1.v.2000 [AP1168, 2♂, CAS]; Fawnskin, 34.2681, -116.9417^3^, 2077m, L Underwood [AP028, 4♂, UCR]; Fontana, 34.0922, -117.4342^6^, 377m, E Schlinger 16.vii.1942 [AP450, 1♂, AMNH]; Just N Alta Loma, end Archibald Avenue, N/S running ravine, E facing slope, 34.1722, -117.5947^1^, 850m, J Bond 6.iv.1996 [AP715, 1♀, AUMNH]; Lytle Creek Rd, near Scotland, 34.244, -117.4952^1^, 950m, M Hedin, J Satler, J Starrett, C Richart 15.ii.2009 [MY3763, 1juv, AUMNH]; San Bernardino, N edge city limits, beside hwy 18, 0.8km before turnoff to Waterman Canyon, 34.1918, -117.2773^3^, 696m, W Icenogle 25.iv.1999 [AP1192, 1281, 1juv, 1♀, CAS]; Yucaipa, 0.8km W junction Grape Avenue & Bryant Street, 34.0641, -117.0425^1^, 853m, W Icenogle 20.ii.2000 [AP353, 1♂, AUMNH], 23.i.2000 [AP359, 1♂, FMNH], 3.ii.2000 [AP364, 1♂, CAS], 14.xi.2000 [AP1242, 1♂, FMNH]; Yucaipa, residential area 183m S intersection Yucaipa Blvd, 34.065, -117.0422^4^, 853m, W Icenogle 3.xi.1968 [AP457, 1♀, 140juv, AMNH]; Yucaipa, Grape Rd, Housing Development, 34.065, -117.0422^1^, 852m, J Bond 17.xii.1997 [AP704, 1♀, AUMNH]; **San Diego Co.:** 0.8km N San Ysidro, 32.5629, -117.0364^4^, 104m, W Icenogle 5.iii.1974 [AP638, 1♀, 1juv, AMNH]; Border Field St Park, S of Monument Blvd., just N international border, 32.5351, -117.1191^1^, 16m, USGS-BRD San Diego Sta. 1.ii.2000 [AP1191, 1♂, CAS]; Carmel Mountain, 32.9283, -117.2227^1^, 118m, USGS-BRD San Diego Field Sta. 1.xii.2002 [AP939, AP941, 2♂, CAS], 1.xi.2002 [AP943, 1♂, CAS]; Chula Vista, E Long Canyon, 32.65, -116.9908^1^, 91m, USGS-BRD San Diego Field Sta. 1.ii.2000 [AP1069, 1♂, CAS]; Chula Vista, E Long Canyon, 32.6501, -116.9921^1^, 90m, USGS-BRD San Diego Field Sta. 1.xi.1998 [AP1073, 1♂, CAS]; Chula Vista, Proctor Valley, SE Horseshoe Bend, S Proctor Valley Rd, 32.663, -116.9806^1^, 152m, USGS-BRD San Diego Field Sta. 1.xi.1998 [AP1096, 2♂, CAS]; Chula Vista, Proctor Valley, SE Horseshoe Bend, S Proctor Valley Rd, 32.6637, -116.9798^1^, 151m, USGS-BRD San Diego Sta. 1.vii.2000 [AP1066, 1♂, CAS], 1.xi.1998 [AP1068, 1♂, CAS], 1.ii.2000 [AP1224, 1♂, CAS]; Chula Vista, Terra Nova, N Rice Canyon, 32.6401, -117.0381^1^, 94m, USGS-BRD San Diego Sta. 1.ii.2000 [AP1092, 1♂, CAS], 1.xi.1998 [AP1093, 1♂, CAS], 1.x.2000 [AP1094, 1♂, CAS]; Chula Vista, Terra Nova, N Rice Canyon, 32.6409, -117.0363^1^, 88m, USGS-BRD San Diego Field Sta. 1.v.2000 [AP1088, 1♂, CAS], 1.xi.1998 [AP1084, 1♂, CAS]; Chula Vista, Terra Nova, N of Rice Canyon, 32.6432, -117.0377^1^, 105m, USGS-BRD San Diego Field Sta. 1.ii.2000 [AP1095, 1♂, CAS]; Del Mar, 32.96, -117.265^3^, 37m, J Comstock 20.x.1956 [AP031, 2♂, AMNH]; E Lakeside between El Monte Park & entrance to El Capitan Res, El Monte Rd, 32.8836, -116.8223^1^, 180m, M Hedin 23.ii.2008 [MY3635, 1juv, AUMNH]; E of Border Field St Park, W Goat Canyon, just N international border, 32.5378, -117.1073^1^, 63m, USGS-BRD San Diego Sta. 1.xi.2002 [AP919, 1♂, CAS], 16.i.2003 [AP920, 1♂, CAS]; El Cajon, 32.7946, -116.9616^5^, 132m, P Smock 25.i.1969 [AP037, 1♂, AMNH], 30.xi.1968, [AP654, 1♀, 1juv, AMNH]; El Monte Park Rd., 32.8839, -116.8214^1^, 166m, M Hedin 19.i.2002 [MY0305, 0306, 2♀, AUMNH]; Fallbrook, 8km SE Fallbrook town center, Wilt Rd, 6.4km NW of hwy 76 & 395 jct, 33.3518, -117.1761^3^, 206m, W Icenogle 1.v.1968 [AP270, AP461, 1♀, 3juv, CAS]; hwy 395 6.4km E Fallbrook, 0.4km from jct Mission Rd, 33.3844, -117.1765^4^, 238m, W Icenogle 20.ix.1971, [AP263, 1♀, CAS]; hwy 76, 11.4km E junction with South 15, 33.2587, -116.8^1^, 768m, M Hedin, J Starrett, J Skejic 10.ii.2002 [AP1246, 1♀, AUMNH]; Jamul Hollenbeck Canyon, E hwy 94, 32.6799, -116.8211^1^, 245m, USGS-BRD San Diego Field Sta. 7.xi.2003 [AP886, 2♂, CAS]; Jamul, S Sweetwater River & Steele Canyon jct, 32.7241, -116.9443^1^, 108m, USGS-BRD San Diego Sta. 1.xi.1998, [AP906, 1♂, CAS]; Jamul, S Sweetwater River & Steele Canyon junction, 32.7192, -116.9521^1^, 104m, USGS-BRD San Diego Sta. 1.ii.2000 [AP912, 2♂, CAS]; Jamul, S Sweetwater River & Steele Canyon Junction, 32.7227, -116.9493^1^, 108m, USGS-BRD San Diego Sta. 1.xi.1998 [AP909, 910, 2♂, CAS]; Jamul, S Sweetwater River & Steele Canyon Junction, 32.7251, -116.9478^1^, 105m, USGS-BRD San Diego Sta. 1.ii.2000 [AP907, 2♂, CAS]; Jamul, SE Jamul Butte, E HWY 94, 32.6916, -116.8344^1^, 280m, USGS-BRD San Diego Sta. 7.xi.2003 [AP885, 1♂, CAS]; Jamul, SW Honey Springs Ranch, S Honey Springs Rd, 32.6632, -116.7989^1^, 389m, USGS-BRD San Diego Sta. 7.xi.2003 [AP887, 3♂, CAS]; Little Cedar Canyon, NE Otay Mtn, 32.6181, -116.8605^1^, 420m, USGS-BRD San Diego Sta. 1.xi.2000 [AP914, 1♂, CAS]; Nate Harrison Grade Rd, 3.4km E jct w/hwy 76, 33.3269, -116.9652^1^, 549m, M Hedin 11.i.2003 [MY0719, 1juv, AUMNH]; N Fallbrook on DeLuz Rd, 33.4109, -117.2898^1^, 232m, J Bond, M Hedin 30.i.2004 [MY2465, MY2467, 2♀, AUMNH]; Otay Mesa, E Spring Canyon, N Wruck Canyon 1, 32.5545, -116.9988^1^, 138m, R Fisher 16.i.2003 [AP821, 2♂, CAS]; Palomar Mountain area Cleveland Natl Forest, St Rd S6, ~4.8 rd km SW intersection of S6 & S7, 33.3048, -116.8786^4^, 1275m, W Icenogle 15.vii.1970 [AP009, 2♀, CAS]; Palomar Mtns, HWY S6, 1.4km N jct w/St Park Rd, 33.343, -116.9004^1^, 1445m, M Hedin, J Starrett, J Skejic 10.ii.2002 [AP1255, 1♀, AUMNH]; Rt 395, 0.3km S turnoff to Fallbrook onto Co Rd 513, 33.3811, -117.1739^4^, 217m, F Coyle 28.vii.1972 [AP639, 1♀, AMNH]; San Diego, 32.732, -117.102^3^, 85m, B Kaston 1.xi.1970 [AP019, 1♂, AMNH]; San Diego Area, 32.7327, -117.1104^7^, 65m, L Hutton 5.xi.1976 [AP038, 1♂, CAS]; San Diego, near San Diego St University, N end Hewlett Dr, 32.7776, -117.079^4^, 85m, B Kaston 1.xii.1970 [AP449, 3♂, CAS]; Tijuana Estuary, W Imperial Beach Naval Air station, 32.5603, -117.1263^1^, 4♂, USGS-BRD San Diego Field Sta. 1.iv.1999 [AP922, 923, 2♂, CAS]; Tijuana Estuary, W Imperial Beach Naval Air station, 32.5616, -117.1257^1^, 20m, USGS-BRD San Diego Sta. 1.ii.1999 [AP924, 1♂, CAS]; Torrey Pines St Reserve, 32.9205, -117.2574^3^, 60m, R Fisher 1.xi.1998 [AP862, 2♂, CAS]; Torrey Pines St Reserve 8, 32.927, -117.2562^3^, 12m, R Fisher 1.xii.1999 [AP868-870 2♂, 1juv, CAS]; Torrey Pines St Reserve, E beach, W of N Torrey Pines Rd 1, 32.9205, -117.2574^1^, 62m, R Fisher 1.ii.2000 [AP861, 1♂, CAS]; Torrey Pines St Reserve, E of beach, W of N Torrey Pines Road 3, 32.9217, -117.2568^1^, 59m, R Fisher 1.xi.1998 [AP864, 1♂, CAS], 1.xii.1999 [AP865, 1♂, CAS]; Torrey Pines St Reserve, S Del Mar Heights School 11, 32.9442, -117.2532^1^, 90m, R Fisher 1.v.1998 [AP858, 3♂, CAS]; Torrey Pines St Reserve, S Del Mar Heights School 2, 32.9398, -117.2518^1^, 62m, R Fisher 1.xii.1999 [AP849, 1♂, CAS]; Torrey Pines St Reserve, S Del Mar Heights School 5, 32.9414, -117.2504^1^, 85m, R Fisher 1.xi.1998 [AP852, 1♂, CAS], 1.xii.1999 [AP853, 1♂, CAS]; Torrey Pines St Reserve, S Del Mar Heights School 7, 32.9402, -117.2528^1^, 62m, R Fisher 1.xi.1998 [AP854, 1♂, CAS]; Torrey Pines St Reserve, W of N Torrey Pines Rd, 32.9258, -117.2505^1^, 11m, USGS-BRD San Diego Sta. 1.ii.2000 [AP929, 1♂, CAS], 1.xii.1999 [AP930, 1♂, CAS]; Torrey Pines St Reserve, W of N Torrey Pines Rd, 32.9208, -117.2568^1^, 66m, USGS-BRD San Diego Sta. 1.xi.1998 [AP927, 1♂, CAS]; Torrey Pines St Reserve, W of N Torrey Pines Rd 2, 32.9144, -117.2506^1^, 101m, R Fisher 1.iv.2000 [AP845, 1♂, CAS]; University of California Elliot Reserve, Carroll Canyon, 32.8943, -117.1058^1^, 166m, USGS-BRD San Diego Sta. 1.xi.1998 [AP877, 1♂, CAS]; University of California Elliot Reserve, S of Carroll Canyon, 32.8945, -117.0867^1^, 218m, USGS-BRD San Diego Sta. 1.iv.2000 [AP883, 1♂, CAS]; University of California Elliot Reserve, S of Carroll Canyon, 32.8917, -117.0971^1^, 194m, USGS-BRD San Diego Sta. 1.xi.1999 [AP880, 1♂, CAS]; University of California Elliot Reserve, S of Carroll Canyon, 32.8926, -117.1058^1^, 186m, USGS-BRD San Diego Sta. 1.ii.2000 [AP878, 1♂, CAS]; vic. Chula Vista, 32.6405, -117.1041^3^, 5m, R Fisher 21.ii.2000 [AP1250, 1♂, CAS]; San Diego, 32.7153, -117.1564^7^, 20m, B Kaston 1.xii.1971 [AP026, 2♂, AMNH]; Camp Pendleton Marine Corps Base, E of De Luz Creek Rd, SW of Sandia Canyon, Pitfall array CAM-23, 33.4112, -117.295^1^, 337m, R Fisher [AP825, 1♂, CAS]; Del Mar Mesa, 32.9404, -117.1742^1^, 105m, USGS-BRD San Diego Sta. 1.ix.2002 [AP1033, 1♂, CAS]; Marron Valley, 32.5673, -116.7737^1^, 171m, USGS-BRD San Diego Sta. 1.xi.2000 [AP970, 1♂, CAS]; Pt Loma, Ft Rosecrans, US Navy Reservation, NE of cemetery, 32.6949, -117.2435^1^, 90m, R Fisher 1.x.1998 [AP817, 1♂, CAS].

#### GenBank accessions.

16S-tRNAval-12S: JX103319-JX103339.

#### Distribution and natural history.

**Maps 14, 15.**
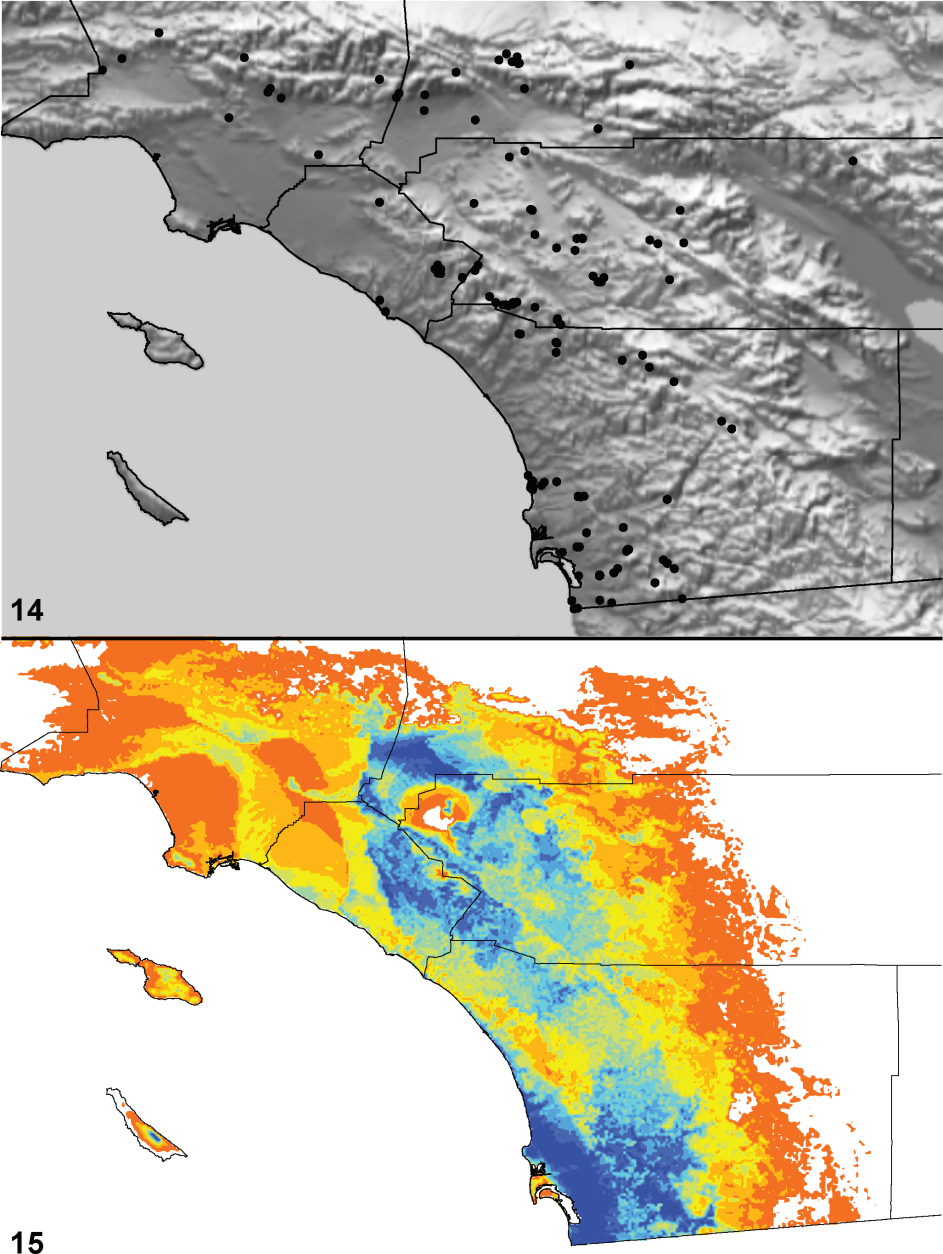
*Aptostichus icenoglei* sp. n. **14** distribution of known specimens **15** predicted distribution; cooler colors–blue shades–represent areas of high probability of occurrence, warmer colors–yellow and orange shades–represent areas of low probability of occurrence.

Most *Aptostichus icenoglei* specimens have been collected from the southern California counties Los Angeles, Orange, Riverside, San Bernardino, and San Diego (Map 14). Although only a single specimen is known from the northern reaches of the Baja Peninsula, it is likely that the species is distributed more extensively in this region of Mexico. The species is distributed widely throughout the Transverse Ranges to the north of the Los Angeles Basin and southward into the Santa Ana and San Jacinto Mountains and the ranges and hills surrounding the San Diego area (San Ysidro and Jamul Mountains and points north of the city). The DM (Map 15) indicates that considerable suitable habitat likely can be found in the areas surrounding San Diego and throughout the Santa Ana and San Jacinto Mountains. Interestingly the model shows the Transverse Ranges to the north and west of the Los Angeles Basin to be an area of lower probability. Given genetic structuring in populations and diversity of habitat it may very well be likely that *Aptostichus icenoglei* as defined here comprises more than one species. Primary habitat types for this species include coastal chaparral forest and shrub and coastal range open woodland shrub and coniferous forest. Based on pitfall trap records, male dispersal is wide ranging (August–May), however, most wandering males at lower altitudes appear to be taken during the early winter months of November–January likely associated with the winter rains.

#### Conservation status.

The status of this species is likely secure given how abundant it is in collections and how widespread it is geographically.

#### Species concept applied.

Morphological/Phylogenetic.

#### Remarks.

Despite some subtle differences, female *Aptostichus icenoglei* specimens can be difficult to distinguish from *Aptostichus atomarius*; juveniles are very problematic; molecular data were used to place many specimens (adults and juveniles) collected over the course of this study. As discussed above, *Aptostichus icenoglei* as currently defined is found in a diversity of habitats across a considerable geographical range. The ~20 mtDNA sequences thus far collected for the species show considerable structuring that is consistent with cryptic species complexes hypothesized for other *Aptostichus* species. As such this taxon will likely be further divided at some point in the future on the basis of more detailed molecular and ecological studies.

### Aptostichus cabrillo sp. n.

‘The Cabrillo Monument Trapdoor Spider’

urn:lsid:zoobank.org:act:DE290777-DB08-47D3-B701-B78F42008CFD

http://species-id.net/wiki/Aptostichus_cabrillo

Figures 157 –164 Maps 16, 17

#### Types.

Male holotype and paratype (AP1161) from California, San Diego County, Point Loma, Cabrillo National Monument, 32.66818, -117.24230^1^, 42m, coll. R. Fisher (USGS-BRD San Diego Field Station) vii.2002. Female paratype (MY3800) from type locality, 32.7101, -117.2523^1^, coll. J. Satler 13.vii.2009. Deposited in AUMNH.

#### Etymology.

The specific epithet is a noun taken in opposition taken from the type locality, Cabrillo National Monument, named in honor of Juan Rodríguez Cabrillo one of the first European explorers of the North American west coast.

#### Diagnosis.

*Aptostichus cabrillo* most closely resembles *Aptostichus icenoglei* specimens and are differentiated from that species in its diagnosis (above).

#### Description of male holotype.

**Figures 157–161.**
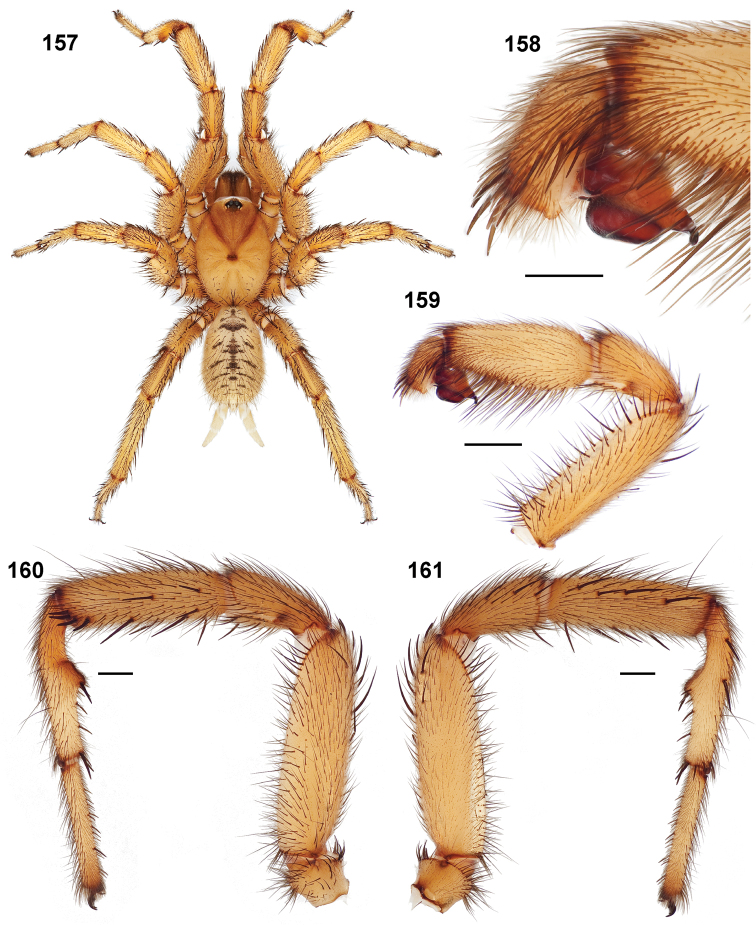
*Aptostichus cabrillo* sp. n. male holotype from San Diego Co. (AP1161). **157** habitus [806592]; scale bars = 1.0mm **158** retrolateral aspect, palpal bulb [806596] **159** retrolateral aspect, pedipalp [806713] **160, 161** leg I, retrolateral and prolateral aspect views [806600, 806598].

*Specimen preparation and condition*. Specimen collected live from pitfall trap, preserved in 80% ETOH. Coloration lightly faded. Pedipalp, leg I left side removed, stored in vial with specimen. *General coloration*. Carapace, chelicerae, legs strong brown 7.5YR 5/8. Abdomen strong brown 7.5YR 4/6, with light mottled striping, ventrum, spinnerets pale yellow (Fig. 157). *Cephalothorax*. Carapace 6.00 long, 4.75 wide, lightly hirsute with thin white spines, stout black bristles along fringe; pars cephalica elevated. Fringe, posterior margin with black bristles. Foveal groove deep, straight. Eyes on moderately high mound. AER slightly procurved, PER slightly recurved. PME, AME subequal diameter. Sternum moderately setose, STRl 3.32, STRw 2.60. Posterior sternal sigilla small, positioned more towards outer margin, not contiguous, anterior sigilla pairs small, oval, marginal. Chelicerae with distinct anterior tooth row comprising 7 teeth, posterior margin with small patch of small denticles. Palpal endites with patch of small cuspules on proximal, inner margin, labium with 4 poorly formed cuspules, LBw 0.94, LBl 0.60. Rastellum consists of 6 stout spines not on mound. *Abdomen*. Setose, heavy black setae intermingled with fine black setae. *Legs*. Leg I: 5.35, 3.90, 3.95, 3.00, 1.75; leg IV: 5.30, 3.25. Very light tarsal scopulae on legs I, II. Tarsus I with single, slightly staggered row of 12 trichobothria. Leg I spination pattern illustrated in Figures 160, 161; TSp 4, TSr 3, TSrd 4. *Pedipalp*. Articles stout, lacking distinct spines (Fig. 159). PTw 1.0, PTl 2.25, Bl 1.05. Embolus slender, sinuous, tapering sharply toward tip, lacking serrations (Fig. 158).

**Variation (7).** Cl 5.05-6.00, 5.40±0.14; Cw 3.75-4.75, 4.20±0.12; STRl 2.73-3.32, 3.07±0.08; STRw 2.10-2.60, 2.37±0.06; LBw 0.74-0.94, 0.81±0.03; LBl 0.43-0.60, 0.5±0.02; leg I: 4.65-5.35, 5.00±0.09; 3.50-3.90, 3.69±0.06; 3.21-3.95, 3.52±0.10; 2.40-3.00, 2.57±0.08; 1.60-1.95, 1.77±0.05; leg IV: 4.75-5.30, 5.03±0.08; 2.69-3.25, 2.84±0.07; PTl 1.93-2.25, 2.08±0.04; PTw 0.77-1.00, 0.91±0.03; Bl 0.96-1.10, 1.02±0.02; TSp 2-4, 3.29±0.36; TSr 2-6, 3.14±0.51; TSrd 4-10, 5.57±0.92.

#### Description of female paratype.

**Figures 162–164.**
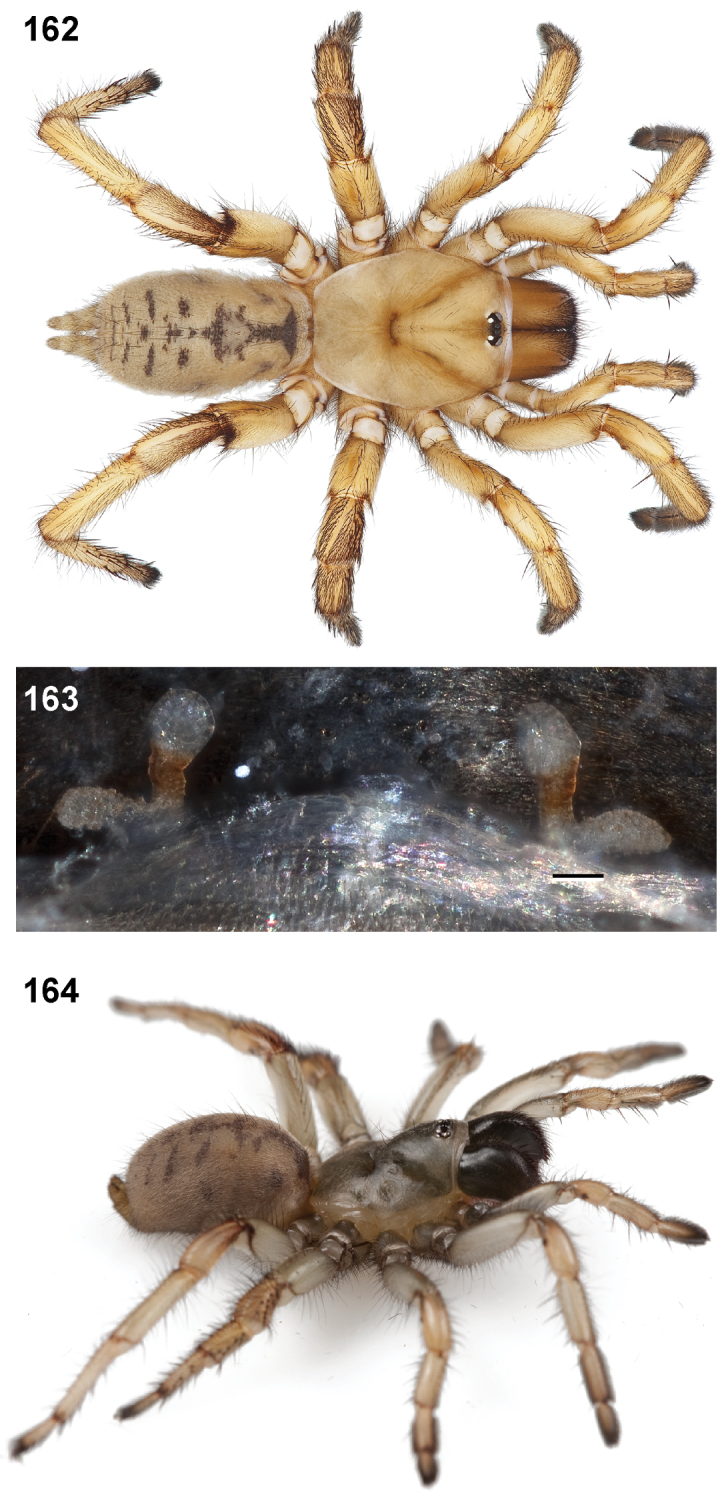
*Aptostichus cabrillo* female from San Diego Co., type locality. **162, 163** habitus and cleared spermathecae of paratype (MY3800) [806605]; scale bar = 0.1mm **164** live photograph (MY3801).

*Specimen preparation and condition*. Female collected live from burrow, preserved in same manner as male holotype. Genital plate removed, cleared in trypsin, stored in microvial with specimen. *Color*. Carapace, legs, chelicerae, yellowish brown 10YR 5/4. Abdomen brown dorsally 10YR 4/3 with distinct chevron striping, ventrum, spinnerets pale yellow. *Cephalothorax*. Carapace 6.19 long, 5.25 wide, glabrous; generally smooth surface, lightly hirsute, pars cephalica moderately elevated (Figs 162, 164). Fringe lacks setae. Foveal groove deep, procurved. Eye group slightly elevated on low mound. AER slightly procurved, PER slightly recurved. PME, AME subequal diameter. Sternum widest at coxae II/III, moderately setose, STRl 4.16, STRw 3.07. Three pairs of sternal sigilla anterior pairs small , oval, positioned marginally, posterior pair slightly larger, oval, mesially positioned but not contiguous. Chelicerae anterior tooth row comprising 8 teeth with posterior margin denticle patch comprising two rows. Palpal endites with 37 cuspules concentrated at inner (promargin) posterior heel; labium with 4 cuspules, LBw 1.19, LBl 0.66. Rastellum consists of 6 very stout spines not positioned on mound. *Abdomen*. Moderately setose. PLS all 3 segments with spigots. Terminal segment 1/2 length of medial segment, 2 enlarged spigots visible at tip. PMS single segment, with spigots, short with rounded terminus. *Legs*. Anterior two pairs noticeably more slender than posterior pairs. Leg I 14.71 long. Tarsus I with single staggered row of 17 trichobothria. Legs I, II with moderately heavy scopulae on tarsi, metatarsi, distal aspect tibia. PTLs 10, TBs 6. Distinct preening comb on retrolateral distal surface at tarsus-metatarsus joint of metatarsus III, IV. *Spermathecae*. Heavily sclerotized intermediate sized median stalk with small slender basal extension (Fig. 163).

**Variation (3).** Cl 6.00-6.38, 6.19±0.11; Cw 4.95-5.45, 5.22±0.15; STRl 3.72-4.16, 3.89±0.14; STRw 2.85-3.30, 3.07±0.13; LBw 1.07-1.19, 1.13±0.03; LBl 0.66-0.77, 0.73±0.04; Leg I: 14.20-15.16, 14.69±0.28; ANTd 6-8, 7.00±0.58; PTLs 10-14, 12.00±1.15; TBs 4-6, 5.33±0.67.

#### Material examined.

**Mexico: Baja California:** 9km NW Rancho Santa Ines, 29.766, -114.766^1^, 550m, [AP634, 1♂, AMNH], P Blom, W Clark [AP635, 422, 423, 3♂, AMNH]; Sierra San Pedro Martir Natl Park, Vallecitos, 32.3833, -116.8833^6^, 500m, 21.vii.1977 [AP633, 1♂, CAS]; **United States: California: San Diego:** 0.8km N of Mexican border & 2.4km E of ocean, 32.5434, -117.1143^5^, 3♂, W Icenogle 19.i.1969 [AP637, 1♀, AMNH]; Otay Mesa, S hwy 905, Spring Canyon, 32.5601, -117.0005^1^, 107m, USGS-BRD San Diego Sta. 1.vii.2001 [AP973, ♂, CAS]; Chula Vista, Terra Nova, N Rice Canyon, 32.6401, -117.0381^1^, 94m, USGS-BRD San Diego Sta. 1.vii.1998 [AP1091, 1♂, CAS]; Chula Vista, Terra Nova, N Rice Canyon, 32.6409, -117.0363^1^, 88m, USGS-BRD San Diego Field Sta. 1.ix.1999 [AP1087, 1♂, CAS]; Chula Vista, Terra Nova, N Rice Canyon, 32.6415, -117.0358^1^, 102m, USGS-BRD San Diego Field Sta. 1.vii.1998 [AP1086, 1♂, CAS]; Chula Vista, E Long Canyon, 32.6501, -116.9921^1^, 90m, USGS-BRD San Diego Field Sta. 1.vii.2000 [AP1078, 1♂, CAS], 1.vii.1998 [AP1076, 1♂, CAS], 1.vii.1999 [AP1074, AP1079, 3♂, CAS]; Pt Loma, Cabrillo Natl Monument, 32.667, -117.24^1^, 89m, J Javier 16.ii.2002 [AP1197, 1♀, CAS]; Pt Loma, US Coast Guard Reservation, S of Cabrillo Natl Monument, 32.667, -117.2421^1^, 22m, R Fisher 1.vii.2002 [AP871, 1♂, CAS]; Pt Loma, Cabrillo Natl Monument, N of U.S. Coast Guard reservation, 32.6681, -117.2423^1^, 33m, USGS-BRD San Diego Sta. 1.vii.2002 [AP1161, 3♂, CAS]; Pt Loma, Cabrillo Natl Monument, (Zuniga Point) E facing slope, 32.6689, -117.238^1^, 54m, USGS-BRD San Diego Sta. 1.vii.2002 [AP1162, 1♂, CAS], 1.ix.2002 [AP1163, 2♂, CAS]; Pt Loma, Cabrillo Natl Monument, W [beach] facing slopes, 32.6719, -117.2444^1^, 22m, USGS-BRD San Diego Sta. 1.vii.2002 [AP1160, 1♂, CAS]; Pt Loma, Cabrillo Natl Monument, W [beach] facing slopes, 32.672, -117.244^1^, 22m, USGS-BRD San Diego Sta. 1.x.2002, [AP1164, 1♂, CAS]; Pt Loma, Ft Rosecrans, US Navy Reservation, NE of cemetery, 32.6959, -117.2429^1^, 83m, USGS-BRD San Diego Sta. 1.viii.2002 [AP1001, 1♂, CAS]; Cabrillo Natl Monument, Pt Loma, 32.7101, -117.2523^1^, 79m, J Satler 13.vii.2009 [MY3800, 3801, 2♀, AUMNH]; El Cajon, 32.7946, -116.9616^5^, 132m, M Hoff 2.xi.1965, [AP572, 1♂, AMNH]; San Diego, Kearny Mesa area, 32.8284, -117.1447^5^, 124m, S Johnson 1.vii.1981, [AP573, 1♂, AMNH]; Flinn Spring, Rios Canyon, 32.8423, -116.8725^1^, 256m, USGS-BRD San Diego Sta. 1.i.2001 [AP1081, 1♂, CAS]; Del Mar Mesa, 32.9403, -117.1717^1^, 126m, USGS-BRD San Diego Sta. 1.vii.2001 [AP996, 1♂, CAS]; Del Mar Mesa, 32.9404, -117.1742^1^, 105m, USGS-BRD San Diego Sta. 1.vi2002 [AP1064, 1♂, CAS]; Del Mar Mesa, 32.942, -117.1749^1^, 104m, USGS-BRD San Diego Field Sta. 1.vii.2001 [AP1060, 2♂, CAS], 12.ix.2002 [AP1061, 1♂, CAS]; Del Mar Mesa, 32.9423, -117.168^1^, 102m, USGS-BRD San Diego Field Sta. 1.vi.2002 [AP1062, 1♂, CAS].

#### Distribution and natural history.

The distribution of *Aptostichus cabrillo* is restricted geographically to a small area in around San Diego, San Diego Co. with one specimen known from just to the south in Mexico (Baja California); the primary habitat throughout this region is coastal chaparral forest and shrub. The DM for this species indicates that suitable habitat in San Diego is restricted to the San Diego area along the coast. Failure to predict the occurrence of some of the outlying localities to the east may suggest that some of those specimens may have been misidentified–however, many of these are males that are easy to distinguish from *Aptostichus icenoglei*. Most male specimens have been collected during the summer months (June–September) with a few taken from pitfalls in October, November and January.

**Maps 16, 17.**
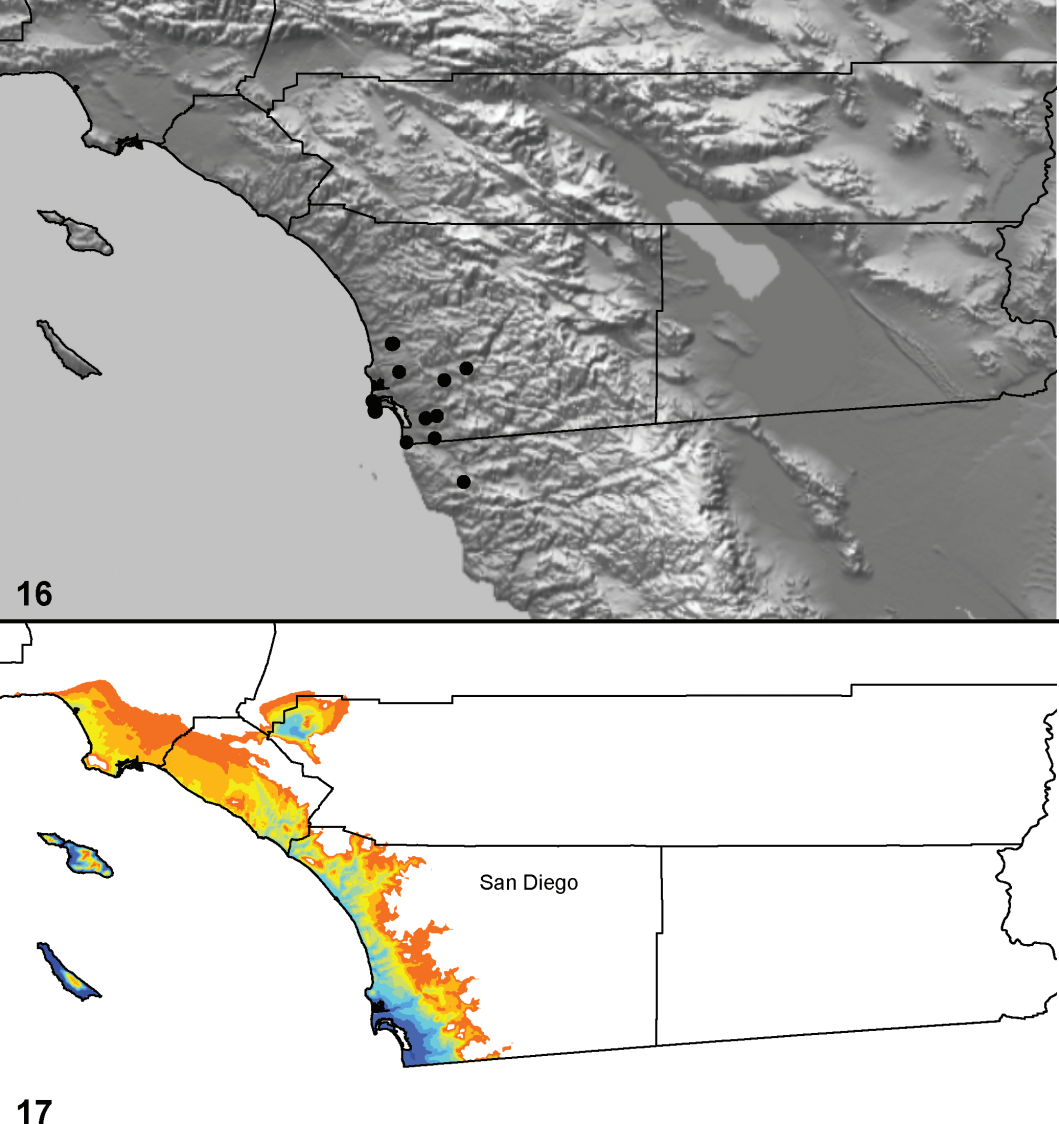
*Aptostichus cabrillo* sp. n. **16** distribution of known specimens **17** predicted distribution; cooler colors–blue shades–represent areas of high probability of occurrence, warmer colors–yellow and orange shades–represent areas of low probability of occurrence.

#### Conservation status.

Because this species is generally rare in collections and restricted in distribution to an area that is highly impacted by development, I consider its status to be vulnerable.

**Species concept applied.** Morphological.

### Aptostichus isabella sp. n.

‘The Lake Isabella Trapdoor Spider’

urn:lsid:zoobank.org:act:9FDFBE3F-F0AE-4861-B088-852AFDEC8FF1

http://species-id.net/wiki/Aptostichus_isabella

**Figures 165–168.**
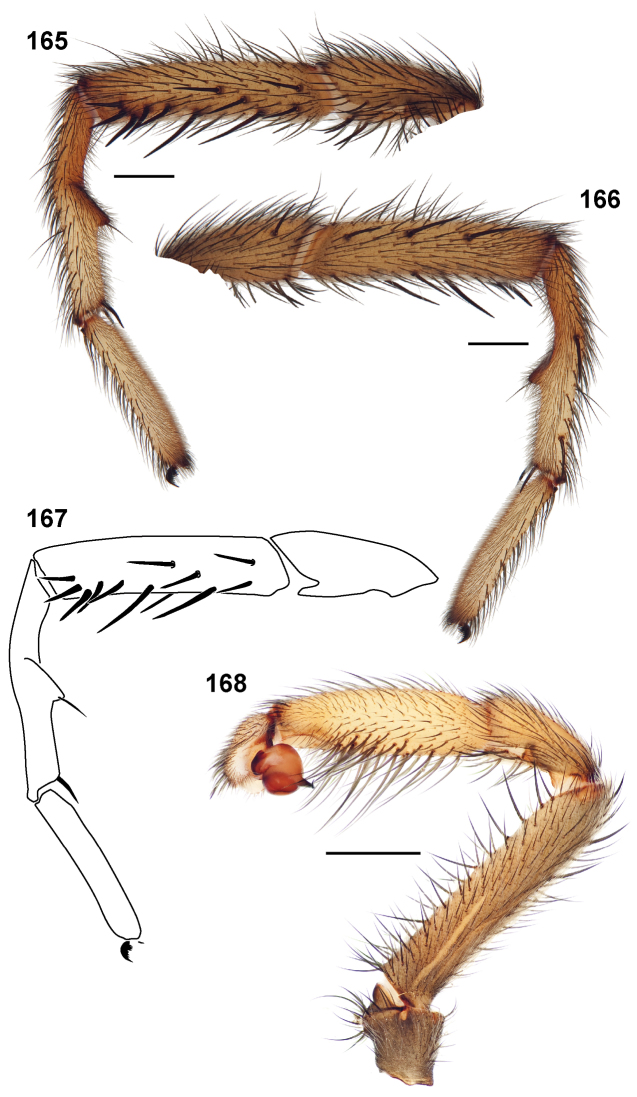
*Aptostichus isabella* sp. n. male holotype (MY3824) from Kern Co. **165–167** leg I **165** retrolateral aspect [805901] **166** prolateral aspect [806607] **167** line drawing, retrolateral aspect **168** retrolateral aspect, pedipalp [805903]. Scale bars = 1.0mm.

Figures 165–168 Map 1

#### Types.

Male holotype (MY3824), from California, Kern County, Erskine Creek Rd., 5.6km E or intersection with Lake Isabella Blvd., E of Bodfish, 35.5689, -118.4383^1^, 925m, coll. J. Satler 8–29x.2010; deposited in AUMNH.

#### Etymology.

The specific epithet is a noun taken in apposition from the type locality, Lake Isabella.

#### Diagnosis.

Males can be diagnosed on the basis of a unique conformation of the tibia I mating apophysis and TSrd spination pattern (Figs 165–167. Like *Aptostichus icenoglei*, the *Aptostichus isabella* tibial I apophysis (Figs 165, 167) is rectangular in shape and bears a distal spine. In all other *Aptostichus* species the tibial I apophysis is triangular, rounded, or absent, with the exception of *Aptostichus cabrillo* which has a similar rectangular apophysis. *Aptostichus isabella* males can be differentiated from *Aptostichus icenoglei* and *Aptostichus cabrillo* males on the basis of the TSr (Figs 165, 167) spination pattern. The TSr of *Aptostichus isabella* consists of a number of spines offset proximally with no TSrd spines whereas *Aptostichus icenoglei* and *Aptostichus cabrillo* have 2-4 non–overlapping TSrd spines.

#### Description of male holotype.

*Specimen preparation and condition*. Specimen collected in pitfall trap, preserved in 80%. Coloration in relatively pristine condition. Pedipalp, leg I left side removed, stored in vial with specimen. *General coloration*. Carapace, chelicerae, legs very dark brown 10YR 2/2. Abdomen yellowish brown 10YR 5/4 with dark distinct chevron striping dorsally. *Cephalothorax*. Carapace 5.60 long, 4.50 wide, very hirsute with white setae, stout black bristles along fringe; surface smooth, pars cephalica elevated. Fringe, posterior margin with black bristles. Foveal groove deep, straight. Eyes on relatively high mound. AER slightly procurved, PER slightly recurved. PME, AME subequal diameter. Sternum moderately setose, STRl 3.13, STRw 2.38. Posterior sternal sigilla small, widely separated, anterior sigilla pairs small, oval, marginal. Chelicerae with distinct anterior tooth row comprising 6 teeth, posterior margin with patch of small denticles. Palpal endites with very small patch of small cuspules on proximal, inner margin, labium lacks cuspules, LBw 0.92, LBl 0.44. Rastellum consists of 6 stout spines not on mound. *Abdomen*. Setose, heavy black setae intermingled with fine black setae. *Legs*. Leg I: 5.69, 4.20, 3.88, 2.79, 2.33; leg IV: 6.00, 3.13. Light scopulae on tarsi, metatarsi legs I, II. Tarsus I with single, slightly staggered row of 10 trichobothria. Leg I spination pattern illustrated in Figures 165-167; TSp 10, TSr 3, TSrd 0. *Pedipalp*. Articles slender, lacking distinct spines (Fig. 168). PTw 0.80, PTl 2.58, Bl 1.26. Embolus long, very slender, tapering gradually toward tip, lacking serrations (Fig. 168).

**Variation.** Known only from the type specimen.

#### Description of female.

Known only from male specimens.

#### Material examined.

Known only from the type specimen.

#### Distribution and natural history.

*Aptostichus isabella* is known only from a single specimen collected from the type locality (Map 1) in Kern Co., Piute Mountains; the habitat in the region is primarily classified as Sierran steppe, mixed coniferous forest.

#### Conservation status.

Undetermined.

#### Species concept applied.

Morphological.

#### Remarks.

Although based on a single specimen, the morphology of this hypothesized species is significantly divergent, and represents an interesting form such that recognizing it as a nominal taxon is warranted.

### Aptostichus muiri sp. n.

‘The Yosemite Valley Trapdoor Spider’

urn:lsid:zoobank.org:act:542F4E47-FDAF-4D5E-86AA-888BCD7AC539

http://species-id.net/wiki/Aptostichus_muiri

**Figures 169–173.**
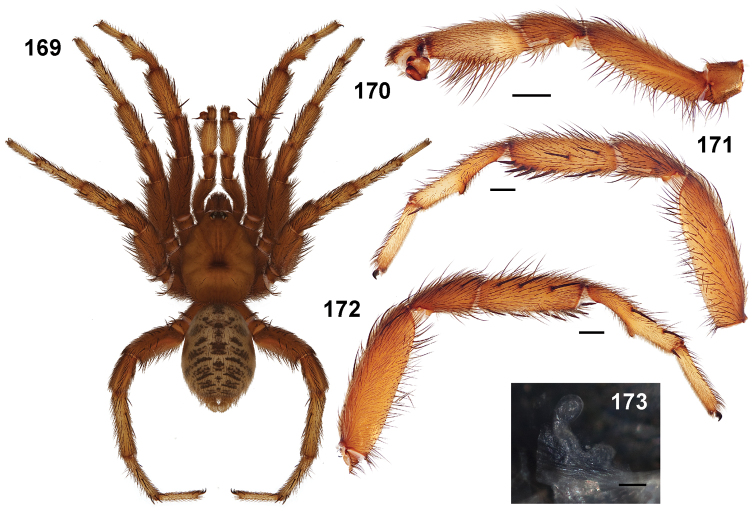
*Aptostichus muiri* sp. n. from Mariposa Co. **169–172** male holotype (AP409); scale bars = 1.0mm **169** habitus [805918] **170** retrolateral aspect, pedipalp [805916] **171** retrolateral aspect, leg I [805910] **172** prolateral aspect, leg I [805914] **173** female paratype (AP1263), cleared spermathecae [805906]; scale bar = 0.1mm.

Figures 169–173 Map 1

#### Types.

Male holotype (AP409) from California, Mariposa County, 3.2km SE of Mariposa, 37.4668, -119.9384^5^, 640m, coll. M. Bentzien 20.ix.1972, deposited in CAS. Female paratype (AP1263) from California, Mariposa County, Yosemite National Park, west facing slope of valley off of “4 Mile” trailhead, 37.7226, -119.5943^1^, 1128m, coll. J. Bond 10.v.1997, deposited in AUMNH.

#### Etymology.

The specific epithet is a patronym in honor of John Muir, one of the first European explorers to visit Yosemite Valley, and to subsequently fight for its preservation.

#### Diagnosis.

Males of this species are similar to those of *Aptostichus atomarius* complex individuals, however *Aptostichus muiri* has fewer TSrd spines and a more slender palpal tibia (Figs 169-172). They can be distinguished from all other species of *Aptostichus* by their unique tibia I spination pattern. The female paratype of this species, collected not far from the type locality, is tentatively placed as a conspecific with the male holotype. This female specimen can be distinguished from *Aptostichus atomarius* by having fewer labial cuspules and a secondary spermathecal bulb (Fig. 173) that is much smaller than that observed for most putative *Aptostichus atomarius* specimens.

#### Description of male holotype.

*Specimen preparation and condition*. Specimen collected live, preserved in 80%. Coloration faded. Pedipalp, leg I left side removed, stored in vial with specimen. *General coloration*. Carapace, chelicerae, legs strong brown 7.5YR 4/6. Abdomen brown 10YR 4/3 dorsally with distinct mottled striping, ventrum, spinnerets pale yellow. *Cephalothorax*. Carapace 5.70 long, 4.70 wide, glabrous, stout black bristles along fringe; surface hirsute with light white spines, pars cephalica elevated. Fringe, posterior margin with black bristles. Foveal groove deep, straight. Eyes on low mound. AER slightly procurved, PER slightly recurved. PME, AME subequal diameter. Sternum moderately setose, STRl 2.91, STRw 2.53. Posterior sternal sigilla moderate size, positioned centrally, not contiguous, anterior sigilla pairs small, oval, marginal. Chelicerae with distinct anterior tooth row comprising 6 teeth, posterior margin with single row of small denticles. Palpal endites with patch of small cuspules on proximal, inner margin, labium lacks cuspules, LBw 0.85, LBl 0.51. Rastellum consists of 5 very stout spines not on mound. *Abdomen*. Setose, heavy black setae intermingled with fine black setae. *Legs*. Leg I: 5.10, 3.50, 3.50, 2.33, 2.05; leg IV: 5.40, 2.75. Light tarsal scopulae on legs I, II. Tarsus I with single, slightly staggered row of 11 trichobothria. Leg I spination pattern illustrated in Figures 171, 172; TSp 3, TSr 3, TSrd 3. *Pedipalp*. Articles slender, lacking distinct spines (Fig. 170). PTw 0.94, PTl 2.18, Bl 1.21. Embolus slender, sinuous, lacking serrations (Fig. 170).

**Variation.** Known only from the type specimen.

#### Description of female paratype.

*Specimen preparation and condition*. Female collected live from burrow, prepared in same manner as male holotype. Genital plate removed, cleared in trypsin, stored in microvial with specimen. *General coloration*. Carapace, legs, chelicerae, brown 7.5YR 4/4. Abdomen black dorsally 7.5YR 2.5/1 with light mottled striping, ventrum, spinnerets pale yellow. *Cephalothorax*. Carapace 5.00 long, 4.08 wide, lightly hirsute with thin black spines; generally smooth surface, pars cephalica moderately elevated. Fringe lacks setae. Foveal groove deep, procurved. Eye group slightly elevated on low mound. AER slightly procurved, PER slightly recurved. PME’s larger in diameter than AME’s. Sternum widest at coxae II/III, moderately setose, STRl 2.83, STRw 2.40. Three pairs of sternal sigilla anterior pairs small, oval, marginal, posterior pair larger, oval, mesially positioned. Chelicerae anterior tooth row comprising 6 teeth with posterior margin denticle patch. Palpal endites with 17 cuspules concentrated at the inner (promargin) posterior heel; labium with 3 cuspules, LBw 0.94, LBl 0.58. Rastellum consists of 6 very stout spines not positioned on mound; fringe of short spines along distal promargin extending upward from rastellum. *Abdomen*. Moderately setose. PLS all 3 segments with spigots. Terminal segment 1/2 length of medial segment, 2 enlarged spigots visible at tip. PMS single segment, with spigots, short with rounded terminus. *Legs*. Anterior two pairs noticeably more slender than posterior pairs. Leg I 11.44 long. Tarsus I with single staggered row of 15 trichobothria. Legs I, II, III with moderately light scopulae on tarsi only. PTLs 12, TBs 3. Rudimentary preening comb on retrolateral distal surface at tarsus-metatarsus joint) of metatarsus III, IV. *Spermathecae*. Median stalk lightly sclerotized, basal extension lacks a well-developed bulb that does not extend below lateral plane of base (Fig. 173)

**Variation.** Known only from the paratype specimen.

#### Material examined.

Known only from the type material.

#### Distribution and natural history.

*Aptostichus muiri* is known only from Mariposa County in the Sierra Nevada Mountains (Map 1); the habitat from which the single female was collected from a shallow burrow is classified as mixed coniferous forest.

#### Conservation status.

Undetermined.

#### Species concept applied.

Morphological.

### Aptostichus barackobamai sp. n.

‘The Barack Obama Trapdoor Spider’

urn:lsid:zoobank.org:act:D037F7E4-D4FD-432B-93A8-C9D7DB932808

http://species-id.net/wiki/Aptostichus_barackobamai

**Figures 174–180.**
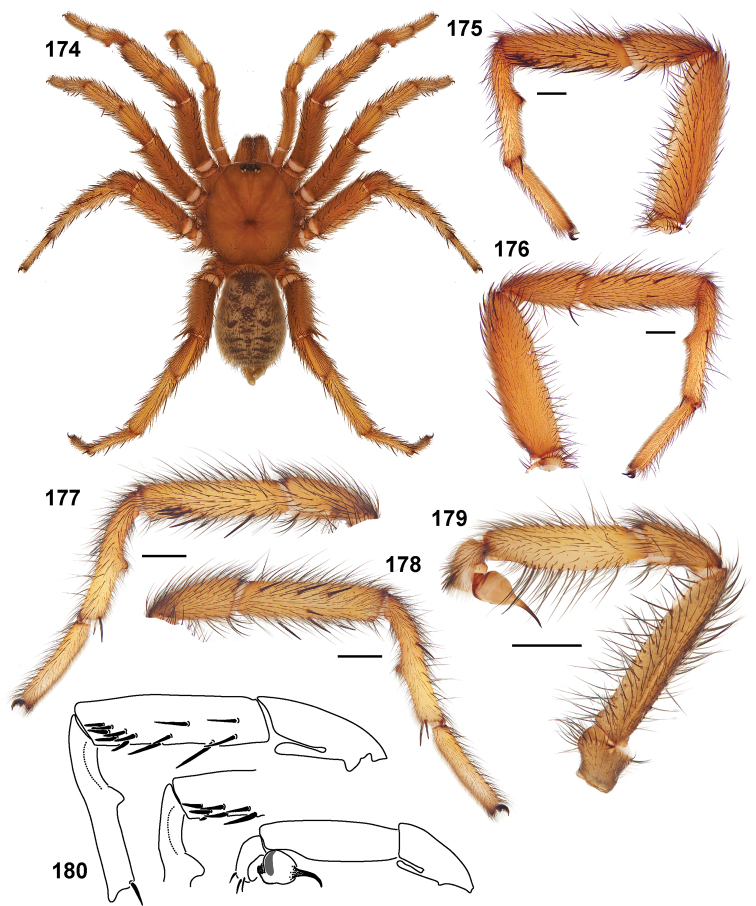
*Aptostichus barackobamai* sp. n. **174–176** male holotype from Mendocino Co. (AP411) **174** habitus [806630] **175** retrolateral aspect, leg I [806634] **176** prolateral aspect, leg I [806636] **177–179** male paratype from Tehama Co. (MY3805) **177** retrolateral aspect, leg I [806638] **178** prolateral aspect, leg I [806642] **179** retrolateral aspect, pedipalp [806644] **180** line drawings of leg I holotype and paratype specimens; retrolateral aspect of holotype pedipalp. Scale bars = 1.0mm.

**Maps 18, 19.**
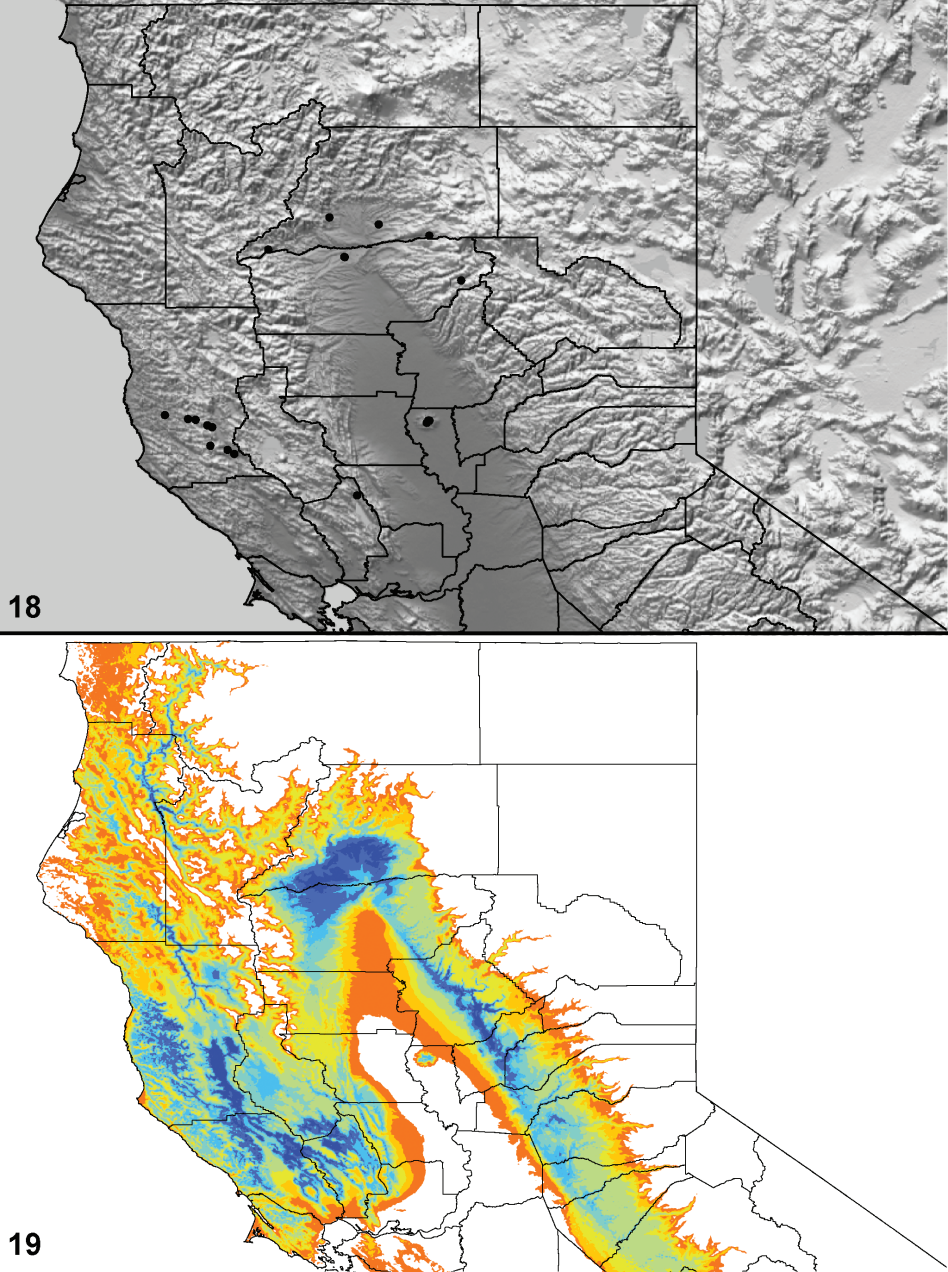
*Aptostichus barackobamai* sp. n. **18** distribution of known specimens **19** predicted distribution; cooler colors–blue shades–represent areas of high probability of occurrence, warmer colors–yellow and orange shades–represent areas of low probability of occurrence.

Figures 174 [Fig F46] –187 Maps 18, 19

#### Types.

Male holotype (AP411) and male paratype (AP411) from California, Mendocino County, Hopland Field Station, 39.0016, -123.0855^3^, 253m, coll. M. Bentzien 29.viii.1973, 21.xii.1972; deposited in AMNH. Male paratype (MY3805) from California, Tehama County, Cottonwood, 40.31677, -122.34998^1^, 183m, coll. C. Will 15.x.2009; female paratype (MY3158) from California, Mendocino County, County Rd 201, 6.8km N of JCT w/ Hwy 175, 39.02832, -123.13034^3^, coll. A. Stockman 18.v.2005; deposited in AUMNH.

#### Etymology.

The specific epithet is a patronym in honor of Barack Obama, first African American President of the United State and reputed fan of spiders.

#### Diagnosis.

**Figures 181, 182. F46:**
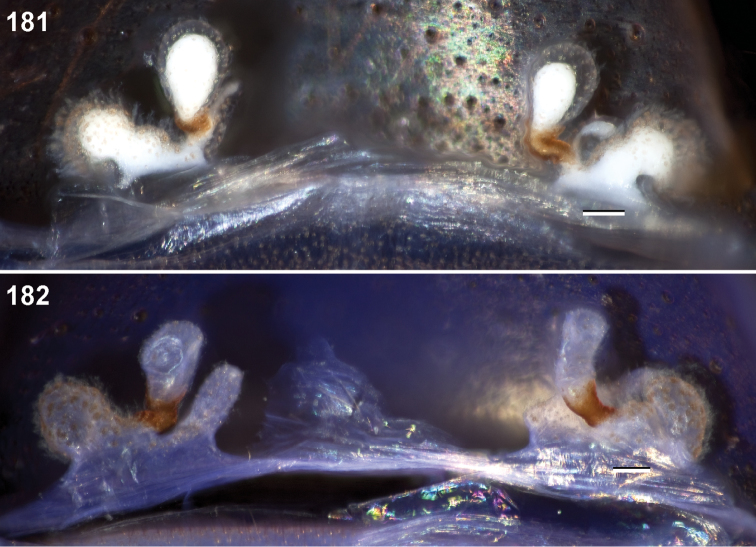
*Aptostichus barackobamai* sp. n. cleared spermathecae; scale bars = 0.1mm. **181** paratype from Mendocino Co. (MY3158) [806709] **182** from Sutter Co. (AP285) [806712].

*Aptostichus barackobamai* males (Fig. 174) can be distinguished from all other *Aptostichus* species on the basis of a unique TSrd spination pattern which comprise numerous spines offset proximally (similar to *Aptostichus isabella*) in combination with a triangular shaped metatarsal mating apophysis (Figs 175-178); male *Aptostichus isabella* specimens have a similar TSrd pattern but have a rectangular mating apophysis. Females can be distinguished from all other known *Aptostichus* species by having a medial auxiliary spermathecal bulb (Figs 181, 182).

#### Description of male holotype.

**Figures 183–187.**
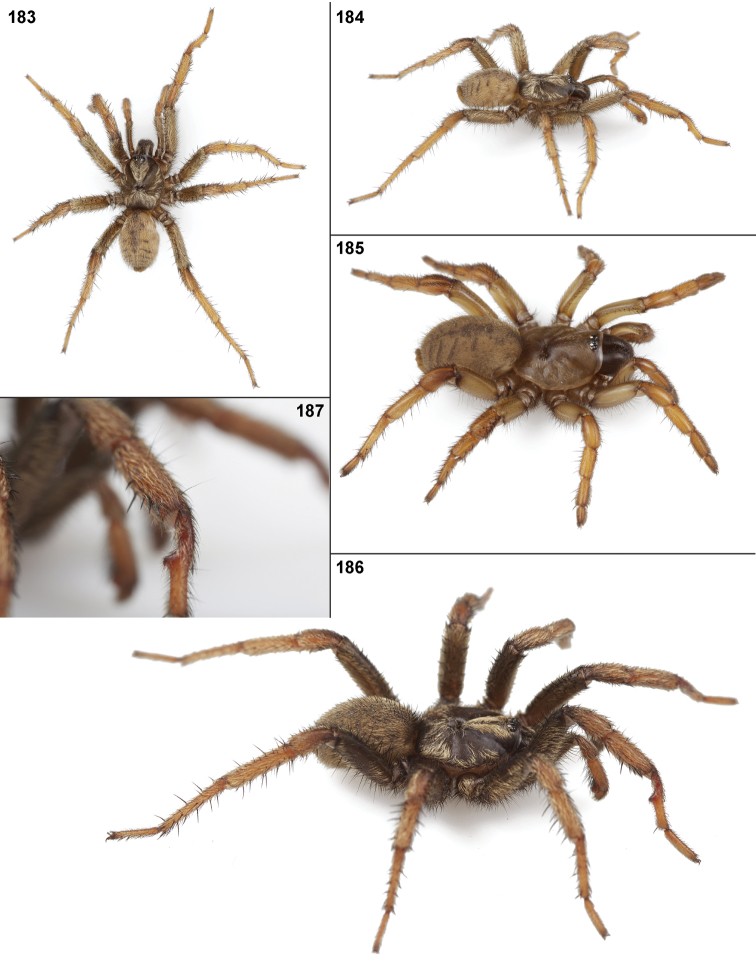
Photographs of live *Aptostichus barackobamai* sp. n. **183, 184** male paratype specimen from Tehama Co. (MY3805) **185** female specimen from Tehama Co. (MY3804) **186, 187** male specimen from Marin Co. (AUMS001).

*Specimen preparation and condition*. Specimen collected live, preserved in 70% EtOH. Coloration presumed faded. Pedipalp, leg I left side removed, stored in vial with specimen. *General coloration*. Carapace, chelicerae, legs dark red 2.5YR 3/6. Abdomen reddish brown 5YR 4/4, distinct mottled striping dorsally Figs 174, 183, 184, 186, 187). *Cephalothorax*. Carapace 5.94 long, 5.06 wide, covered in thin white setae, stout black bristles along fringe; surface smooth, pars cephalica elevated. Fringe, posterior margin with few black bristles. Narrow foveal groove deep, slightly procurved. Eyes on low mound. AER slightly procurved, PER slightly recurved. PME, AME subequal diameter. Sternum moderately setose, STRl 3.16, STRw 2.64. Posterior sternal sigilla moderate in size, positioned towards margin, not contiguous, anterior sigilla pairs small, oval, marginal. Chelicerae with distinct anterior tooth row comprising 6 teeth, posterior margin with single row of small denticles. Palpal endites with single cuspules on proximal, inner margin, labium lacks cuspules, LBw 0.94, LBl 0.53. Rastellum consists of 6 stout spines not on mound. *Abdomen*. Setose, heavy black setae intermingled with fine black setae. *Legs*. Leg I: 5.45, 4.05, 3.80, 2.79, 2.00; leg IV: 5.69, 3.20. Heavy to moderate scopulae on tarsus, metatarsus legs I, II; light scopulae on tarsus legs III, IV. Tarsus I with single, slightly staggered row of 12 trichobothria. Leg I spination pattern illustrated in Figures 175-178; TSp 4, TSr 6, TSrd 7. *Pedipalp*. Articles slender, lacking distinct spines (Fig. 179). PTw 0.75, PTl 2.55, Bl 1.29. Embolus broad, tapering sharply towards very thin tip, curved distally, lacking serrations (Fig. 179).

**Variation (4).** Cl 5.00-6.25, 5.81±0.28; Cw 4.08-5.06, 4.79±0.24; STRl 2.75-3.47, 3.14±0.15; STRw 2.23-2.79, 2.58±0.12; LBw 0.78-0.94, 0.9±0.04; LBl 0.43-0.60, 0.54±0.04; leg I: 4.55-6.13, 5.53±0.36; 3.26-4.45, 4.02±0.27; 3.04-4.00, 3.66±0.21; 2.13-2.79, 2.62±0.16; 1.70-2.17, 1.97±0.1; leg IV: 5.00-6.38, 5.83±0.31; 2.52-3.20, 3.01±0.16; PTl 2.18-2.95, 2.62±0.17; PTw 0.60-0.77, 0.72±0.04; Bl 1.07-1.33, 1.25±0.06; TSp 4-4, 4.00±0; TSr 1-6, 3.25±1.11; TSrd 5-9, 7.00±0.82.

#### Description of female paratype.

*Specimen preparation and condition*. Female collected live from burrow, prepared in same manner as male holotype. Genital plate removed, cleared in trypsin, stored in microvial with specimen. *General coloration*. Carapace, legs, chelicerae, dark reddish brown 2.5YR 2.5/4. Abdomen dark brown dorsally 10YR 3/3, distinct mottled chevron marking pattern. *Cephalothorax*. Carapace 8.00 long, 6.88 wide, lightly hirsute with fine black setae intermingled with white setae; generally smooth surface, pars cephalica moderately elevated. Fringe lacks setae. Foveal groove deep, slightly procurved. Eye group slightly elevated on low mound. AER slightly procurved, PER slightly recurved. PME-AME subequal diameter. Sternum widest at coxae II/III, moderately setose, STRl 4.56, STRw 3.76. Three pairs of sternal sigilla anterior pairs small in size, oval, marginal; posterior pair moderate in size, oval, positioned between margin, midpoint. Chelicerae anterior tooth row comprising 8 teeth with posterior margin denticle patch. Palpal endites with 12 cuspules concentrated at the inner promargin posterior heel; labium with 3 cuspules, LBw 1.39, LBl 1.02. Rastellum consists of 7 very stout spines not positioned on mound, forming a contiguous row; fringe of short spines along distal promargin extending upward from rastellum. *Abdomen*. Moderately setose. PLS all 3 segments with spigots. Terminal segment 1/2 length of medial segment, 2 enlarged spigots visible at tip. PMS single segment, with spigots, short with rounded terminus. *Legs*. Anterior two pairs noticeably more slender than posterior pairs. Leg I 17.25 long. Tarsus I with 12 trichobothria arranged in three staggered rows. Legs I, II with moderately heavy scopulae on tarsus, metatarsus; heavy scopulae distal most aspect tibia I, light scopulae on distal aspect tarsus legs III, IV. PTLs 17, TBs 3. Distinct preening comb on retrolateral distal surface, tarsus-metatarsus joint, of metatarsus III, IV. *Spermathecae*. 2 complex spermathecal bulbs with an elongate curved stalk, large basal extension, third medial bulb (Fig. 181, 182).

**Variation (5).** Cl 7.52-8.56, 8.05±0.19; Cw 6.31-7.52, 6.90±0.20; STRl 4.56-5.35, 4.88±0.16; STRw 3.72-4.40, 3.87±0.13; LBw 1.36-1.65, 1.43±0.06; LBl 0.82-1.11, 0.97±0.05; Leg I: 16.04-19.55, 17.66±0.63; ANTd 8-10, 8.60±0.40; PTLs 13-19, 17.00±1.41; TBs 2-5, 3.50±0.65.

#### Material examined.

**United States: California: Mendocino Co.:** Hopland Field Station, 39.0016, -123.0855^3^, 253m, M Bentzien 21.xii.1972 [AP410, 1♂, AMNH], 29.viii.1973 [AP411, 1♂, AMNH], 6.x.1972 [AP525, 1juv, AMNH], 7.iv.1972 [AP526, 1♀, AMNH], 27.ix.1972 [AP527, 1♂, AMNH], 10.x.1972 [AP528, 1♀, AMNH]; Hwy 253, SW Ukiah, 10.8km W jct W/Hwy 101, 39.0552, -123.245^1^, 600m, M Hedin, J. Starrett, D Leavitt 20.xii.2007 [MY3625, 1♀, AUMNH]; Co Rd 201, 6.8km N jct w/hwy 175, 39.0283, -123.1303^1^, 175m, A Stockman 18.v.2005 [MY3158, 1♀, AUMNH]; Orr Springs Rd, Ackerman Creek- 1st stream crossing W hwy 101, 39.1807, -123.233^1^, 211m, J Bond 14.iii.2005 [MY3025, 1♀, AUMNH]; Orr Springs Rd, 3.8km W bridge, 39.1924, -123.2659^1^, 543m, J Bond 14.iii.2005 [MY3026, 1♀, AMNH]; Orr Springs Rd, W of Hwy 101, 13.4km W 1st bridge, 39.2293, -123.3426^1^, 571m, J Bond 14.iii.2005 [MY3027, 1♀, FMNH], [MY3028, 1juv, AUMNH]; Orr Springs Rd, 19.5km W Ackerman Creek, 39.2352, -123.3941^1^, 225m, J Bond 14.iii.2005 [MY3029, 1♀, AUMNH]; Orr Springs Rd, 38.8km W Ackerman Creek, 39.2609, -123.5485^1^, 246m, J Bond 14.iii.2005 [MY3038, 1♀, AUMNH]; **Napa Co.:** Berryessa Knoxville Rd, 38.7235, -122.2656^1^, 195m, A Stockman 16.v.2005 [MY3138, 1♀, AUMNH]; **Shasta Co.:** Rock Creek Rd, crossing Rock Creek, E Manton, 40.4591, -121.7828^1^, 970m, M Hedin, J Starrett, D Leavitt 19.xii.2007 [MY3629, 1♀, AUMNH]; hwy 44, intersection hwy and Bear Creek, 32.2km E Redding, 6.4km E Millville, 40.5333, -122.1202^5^, 252m, W Icenogle 7.xi.1972 [AP518, 1♀, AMNH]; Platina Rd, 4.7km NE Hwy 36, 40.3656, -122.858^1^, 724m, A Stockman 22.v.2005 [MY3173, 1juv, AUMNH]; Lower Springs Rd, 0.8km S Hwy 299, 40.5807, -122.4501^1^, 225m, A Stockman 23.v.2005 [MY3175, 1♀, FMNH], [MY3176, 1juv, AUMNH]; **Sutter Co.:** Sutter Buttes, Moore Canyon, 39.2085, -121.7993^3^, 61m, W Icenogle 19.vii.1974 [AP285, 1♀, CAS]; Sutter Buttes, Dean Place, 39.223, -121.7812^1^, 259m, M. Hedin, P Paquin, J Starrett 4.iv.2003 [MY729, 1♀, AUMNH]; Sutter Buttes, Hough Canyon, Pete & Margit Sand Ranch, NW Mallot Rd, 39.2236, -121.7892^1^, 239m, A Stockman 13.vi.2005 [MY3336, 3382, 1♀, 1juv, AUMNH]; **Tehama Co.:** Hwy32, S Deer Creek, 5.3km SW Potato Patch CG, 40.1599, -121.5704^1^, 1100m, M Hedin, J Starrett, D Leavitt 19.xii.2007 [MY3622, 1♀, AUMNH]; Cottonwood, 40.3159, -122.3476^1^, 183m, C Will 27.ix.2009 [MY3802, MY3804, 1♂, 1♀, AUMNH], 15.x.2009 [MY3803, MY3805, 1♀, 1♂, AUMNH].

#### GenBank accessions.

16S-tRNAval-12S: JX103422-JX103440.

#### Distribution and natural history.

Distributed widely throughout north-central California with populations known from Mendocino, Napa, Shasta, Sutter, and Tehama Counties (Map 18). *Aptostichus barackobamai* has been collected in the Mayacamas Mountains in the west, Sutter Butte in the Central Valley, and the ridges to the north ringing the northernmost extension of the Central Valley. The DM (Map 19) indicates that the species is likely to be more widely distributed throughout the region and should occur further to the south along the Central Valley eastern ranges. The primary habitat type is mixed redwood and coniferous forest. Males have been collected in August, September, and December.

#### Conservation status.

The conservation status of this species is likely to be secure due to its abundance and widespread distribution; however, may be locally vulnerable (e.g., Sutter Butte locality).

#### Species concept applied.

Morphological/Phylogenetic.

#### Remarks.

Originally thought to be rare, only a few specimens were originally known from the Hopland Field Station locality, collecting efforts in recent years have recovered considerably more specimens and have significantly extended the known distribution of the species.

##### The *Hesperus* species group

**Included species.**

*Aptostichus hesperus* Chamberlin, 1919

*Aptostichus hedinorum* Bond sp. n.

*Aptostichus cahuilla* Bond sp. n.

*Aptostichus killerdana* Bond sp. n.

*Aptostichus serrano* Bond sp. n.

*Aptostichus aguacaliente* Bond sp. n.

*Aptostichus chemehuevi* Bond sp. n.

*Aptostichus sarlacc* Bond sp. n.

*Aptostichus derhamgiulianii* Bond sp. n.

*Aptostichus mikeradtkei* Bond sp. n.

*Aptostichus edwardabbeyi* Bond sp. n.

*Aptostichus anzaborrego* Bond sp. n.

*Aptostichus sinnombre* Bond sp. n.

### Aptostichus hesperus

Chamberlin, 1919

‘The Riverside Trapdoor Spider’

urn:lsid:zoobank.org:act:FDCD8714-9BD8-4F48-824F-D33D657AF392

http://species-id.net/wiki/Aptostichus_hesperus

Figures 188 –196 Maps 20, 21

Nemesoides hespera Chamberlin, 1919: 2. Male holotype (379), California, Los Angeles County, Claremont, 34.0968, -117.7195^5^, coll. W. Hilton, in MCZ, examined.
Aptostichus hesperus –Bond and Opell 2002: 518.

#### Diagnosis.

**Figures 188–191.**
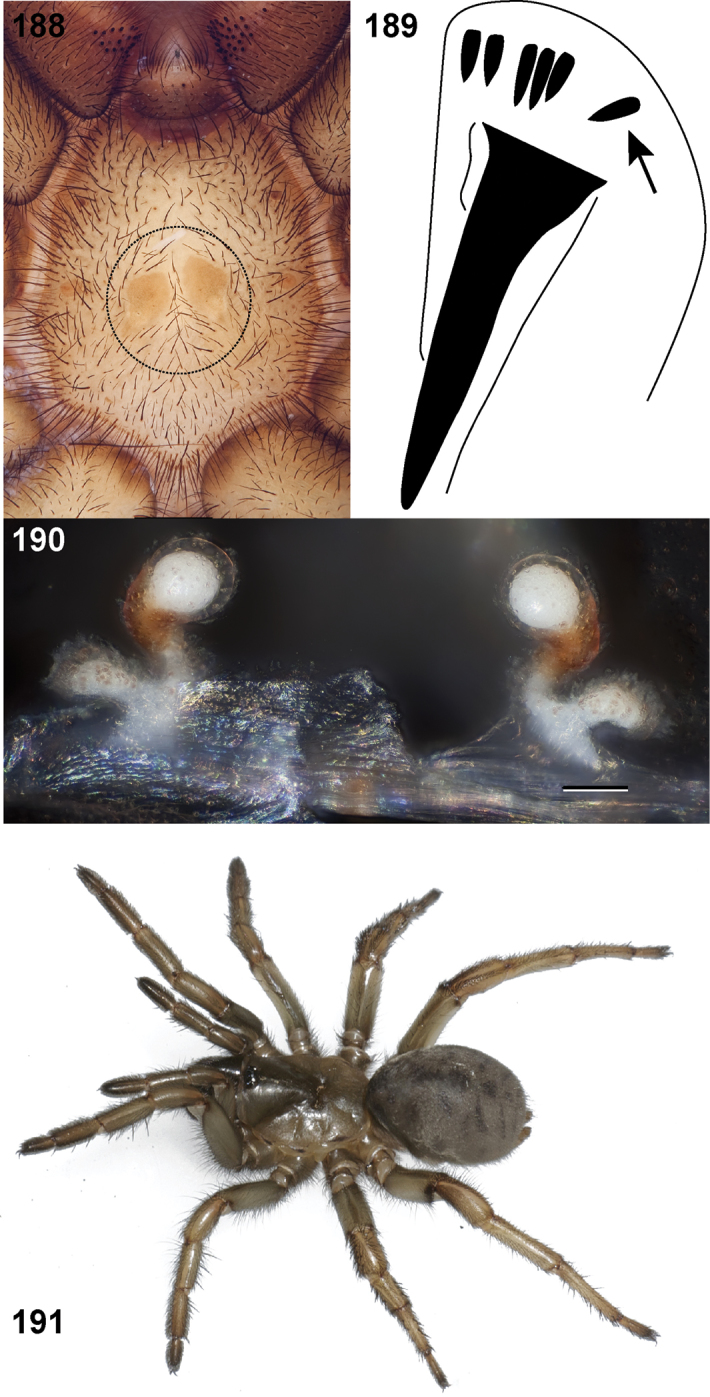
*Aptostichus hesperus* (Chamberlin, 1919). **188** sternal sigilla pattern (indicated by circled area, labium at top of photograph; MY2526)) [805941] **189** line drawing of rastellar spine pattern for *Aptostichus hesperus* and other *Hesperus* group species **190** cleared spermathecae, specimen from San Bernardino Co., Yucaipa (MY2526) [805942]; scale bar = 0.1mm **191** photograph of live specimen from Riverside Co., Winchester(MY2496).

**Figures 192–196.**
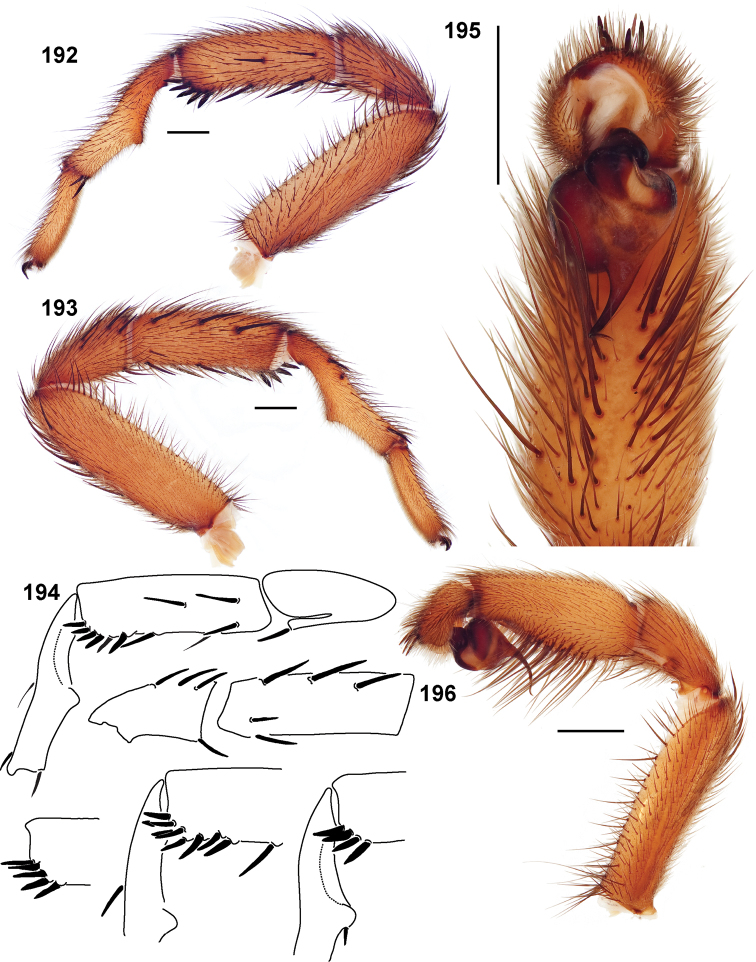
*Aptostichus hesperus* male, photographs of specimen from Riverside Co., Winchester (AP098). **192–194** leg I; scale bars = 1.0mm. **192** retrolateral aspect [805929] **193** prolateral aspect [805925] **194** line drawings of spination pattern variation of leg I **195–196** pedipalp; scale bars = 1.0mm **195** ventral view [805935] **196** retrolateral aspect 805933].

Female (Figs 188–191) and male (Figs 192–196) *Aptostichus hesperus* can be distinguished from all other *Aptostichus* species by having posterior sternal sigilla that are positioned mid - ventrally and are either very closely positioned, or contiguous (Fig. 188). The sigilla of other *Aptostichus* species are distinctly separated and tend to be positioned more posteriorly. A longer palpal bulb length and greater PTw/PTl ratio (Figs 195, 196) also help to distinguish this species from *Aptostichus atomarius*, *Aptostichus cahuilla* and *Aptostichus icenoglei* that potentially occur sympatrically with *Aptostichus hesperus*. All *Hesperus* group taxa have an offset rastellar spine (Fig. 189).

#### Descriptions.

Described by Chamberlin (1919: 1-2).

#### Material examined.

**United States: California: Orange Co.:** S Limestone Canyon, N The Sinks, 33.7231, -117.6663^1^, 378m, USGS-BRD San Diego Sta. 1.v.2001 [AP1054, 1♀, CAS]; Loma Ridge, W Limestone Canyon, 33.7358, -117.6877^1^, 327m, USGS-BRD San Diego Field Sta. 1.xi.2000 [AP1056, 2♂, CAS], 1.xi.1998 [AP1055, 1♂, CAS]; NE Lemon Heights, Lomas de Santiago, E Peters Canyon, 33.7672, -117.7637^1^, 295m, USGS-BRD San Diego Sta. 1.iv.1999, [AP960, 1♂, CAS]; Weir Canyon, 33.8155, -117.7471^1^, 232m, USGS-BRD San Diego Field Sta. 1.iv.2000 [AP1124, 1♂, CAS]; Anaheim, 33.8197, -117.8963^7^, 50m, F Handsfield 1.vii.1962 [AP618, 1♀, AMNH]; Ridge NE Weir Canyon, 33.8376, -117.7219^1^, 354m, USGS-BRD San Diego Sta. 1.i.2000 [AP1097, 1♂, CAS], 1.iv.1999 [AP1098, 1♂, CAS]; Chino Hills, ridge S Telegraph Canyon, 33.9116, -117.7887^1^, 355m, USGS-BRD San Diego Field Sta. 1.x.1998 [AP1039, 1♂, CAS]; **Riverside Co.:** S Santa Margarita River, NW Royal Oak ranch, 33.4463, -117.1719^1^, 353m, USGS-BRD San Diego Sta. 1.v.2000 [AP896, 1♂, CAS]; between Squaw Mountain & Redonda Mesa, N Tenaja Rd., 33.5025, -117.3386^1^, 1020m, USGS-BRD San Diego Sta. 1.ii.2000 [AP968, 1♂, CAS]; between Squaw Mountain & Redonda Mesa, N Tenaja Rd, 33.5028, -117.3406^1^, 690m, USGS-BRD San Diego Sta. 1.vi.1999 [AP966, 1♂, CAS]; SE Skinner Reservoir, 33.5766, -117.0316^1^, 482m, USGS-BRD San Diego Sta. 1.viii.1999 [AP1023, 1♂, CAS]; S Skinner Reservoir, 33.5774, -117.0545^1^, 469m, USGS-BRD San Diego Field Sta. 1.iv.1999 [AP1020, 1♂, CAS]; E Skinner Reservoir, 33.5819, -117.0189^1^, 488m, USGS-BRD San Diego Field Sta. 1.ii.2000 [AP1006, 1♂, CAS], 1.iv.1999 [AP1003, 1♂, CAS]; E Skinner Reservoir, 33.585, -117.0236^1^, 514m, USGS-BRD San Diego Field Sta. 1.ii.2000 [AP1013, 1♂, CAS]; E Skinner Reservoir, 33.5869, -117.0212^1^, 501m, USGS-BRD San Diego Sta. 1.iv.1999 [AP1005, 1010, 1011, 3♂, CAS], 1.v.1999 [AP1004, 1008, 2♂, CAS]; E Skinner Reservoir, 33.5898, -117.0233^1^, 452m, USGS-BRD San Diego Sta. 1.iv.1999 [AP1002, 1♂, CAS]; E Skinner Reservoir, 33.5947, -117.0253^1^, 468m, USGS-BRD San Diego Sta. 1.ii.2000 [AP1030, 1♂, CAS], 1.iv.1999 [AP1031, 1♂, CAS]; E Skinner Reservoir, 33.5977, -117.024^1^, 464m, USGS-BRD San Diego Field Sta. 1.ii.2000 [AP1029, 1♂, CAS], 1.iv.1999, [AP1028, 1♂, CAS]; E Skinner Reservoir, 33.5989, -117.0235^1^, 467m, USGS-BRD San Diego Field Sta. 1.ii.2000 [AP1024, 1025, 2♂, CAS]; E Rawson Canyon, S Crown Valley, 33.631, -117.0086^1^, 655m, USGS-BRD San Diego Sta. 1.v.2000 [AP1119, 1♂, CAS]; E Rawson Canyon, S Crown Valley, 33.6369, -117.0022^1^, 677m, USGS-BRD San Diego Field Sta. 1.v.2000 [AP1117, 1♂, CAS]; just S Winchester on Leona Rd, ~1.6km S intersection w/Patton Avenue, 33.6771, -117.1157^1^, 444m, J Bond 1.ii.2004 [MY2510, 2516 2♀, AUMNH]; Winchester, 33.7138, -117.0915^1^, 470m, W Icenogle 7.ii.1972 [AP143, 1♂, AUMNH], 28.i.1973 [AP146, 1♂, AMNH], W Icenogle 14.vi.1980 [AP145, 1♂, AUMNH], 26.i.1983 [AP144, 1♂, AUMNH]; 1.6km NW Winchester Town Center, 33.7148, -117.0921^1^, 470m, W Icenogle 11.i.1998 [AP358, 1♂, CAS]; Winchester, 1.6km NW of town center, vicinity of Double Butte, 33.7149, -117.0922^1^, 478m, W Icenogle 21.iii.1977 [AP107, 1♂, AMNH], 26.xii.1977 [AP100, 1♂, AMNH], 28.xii.1977 [AP139, 1♂, AMNH], 18.v.1988 [AP129, 1♂, AMNH], 29.xii.1968 [AP140, 2♂, AMNH], 12.ii.1967 [AP112, 1♀, CAS], 19.iii.1967 [AP617, 1♀, AMNH], 28.v.1967 [AP118, 1♀, 10juv, CAS], 11.vi.1967 [AP124, 1juv, AMNH], 6.viii.1967 [AP123, 1♀, AMNH], 23.viii.67 [AP125, 1♀, 45juv, AMNH], 23.viii.1967 [AP126, 1♂, CAS], 15.x.1967 [AP111, 1♀, 35juv, AMNH], 15.x.1967 [AP113, 1♀, AMNH], 12.xi.1967 [AP114, 1♀, 49juv, AMNH], 12.xi.1967 [AP117, 1♀, 46juv, AMNH], 22.xi.1967 [AP120, 1♂, AMNH], 12.xii.1967 [AP099, 1♂, AMNH], 17.viii.1968 [AP108, 1♀, 35juv, AMNH], 29.xii.1968 [AP135, 1♂, AMNH], 16.i.1969 [AP133, 3juv, AMNH], 6.ii.1972 [AP098, 1♂, AMNH]; Winchester, 1.6km NW of town center, vicinity of Double Butte, 33.7156, -117.0936^1^, 465m, J Bond 29.i.2004 [MY2496, 1♀, AUMNH]; ravine NW Icenogle residence, 33.72, -117.0922^1^, 523m, J Bond 26.iii.1996 [AP725, 2♀, AUMNH]; Winchester, deep ravine NW Icenogle residence Grand Ave, 33.7222, -117.0913^1^, 600m, J Bond 7.xi.1998 [AP690, AP733, 1♀, AUMNH]; N of Perris, E of Mayer Farms, 33.8049, -117.254^1^, 559m, USGS-BRD San Diego Field Sta. 1.ii.1999 [AP1101, 1♂, CAS]; N of Perris, E of Mayer Farms, 33.8115, -117.257^1^, 588m, USGS-BRD San Diego Field Sta. 1.i.2000 [AP1104, 1♂, CAS]; E of Perris, E of Mayer Farms, 33.8136, -117.2587^1^, 577m, USGS-BRD San Diego Field Sta. 1.i.2000 [AP1105, 1♂, CAS]; Lake Matthews, 33.82667, -117.438^1^, 446m, J Bond 22.xi.1998 [AP692, 1♀, AUMNH]; NE Perris Reservoir, 33.8666, -117.1938^1^, 533m, USGS-BRD San Diego Sta. 1.ii.1999 [AP925, 1♂, CAS]; Hwy 79, ~6km S Beaumont, N facing slope small ravine, 33.87, -116.9963^1^, 655m, J Bond 19.i.1997 [MY2286, 1juv, AUMNH]; 9.65 road km S of Banning, 33.8797, -116.8458^6^, 1221m, W Icenogle 7.x.1968 [AP103, AP104, 2♂, AMNH], [AP137, 2♀, 2juv, CAS]; E Perris Reservoir, between Bernasconi Hills & Mount Russell, 33.8864, -117.1349^1^, 507m, USGS-BRD San Diego Sta. 1.i.2000 [AP926, 1♂, CAS]; University of California Riverside, 33.9533, -117.3953^6^, 252m, D Bixler 10.v.1968 [AP119, 1juv, AMNH], E Schlinger 6.vii.1957 [AP134, AP142, 5♀, 7juv, AMNH]; University of California, Riverside Campus, 33.9742, -117.3251^3^, 327m, W Icenogle 17.xii.1969 [AP619, 2♀, CAS], 13.ix.1967 [AP110, AP136, 1♀, 26juv, AMNH], 17.ix.1967 [AP101, 1♂, AMNH], 19.ix.1967 [AP109, 1♀, 25juv, AMNH], 25.ix.1967 [AP116, AP616 2♀, 105juv, AMNH], 5.x.1967 [AP127, 1juv, AMNH], 10.x.1967 [AP106, 115, 128, 1♂, 2♀, 51juv, AMNH], 20.xi.1967 [AP105, 1♂, AMNH], 27.x.1967 [AP121, 130, 138, 141, 147, 5♂, AMNH], 31.x.1968 [AP131, 1♀, AMNH], H Nakakihara 11.iv.1974 [AP584, 1♂, UCR]; **San Bernardino Co.:** Yucaipa, bluffs behind city municipal office, 34.035, -117.0597^2^, 737m, J Bond 19.i.1997 [MY2287, MY2289, MY2291, 3juv, AUMNH]; Yucaipa, Jct Yucaipa Blvd & 7th Street, 34.0362, -117.0638^3^, 722m, W Icenogle 3.xii.1995 [AP102, 1♂, CAS]; Yucaipa, Bluff behind city municipal office on barren eroded bank, 34.0362, -117.0598^1^, 737m, J Bond 19.i.1997 [AP1206, 1juv, AUMNH]; Yucaipa, Oak Glen Creek, just N jct 6th St & Yucaipa Blvd, 34.0382, -117.0599^1^, 792m, W Icenogle 29.x.1995 [AP352, 1♂, CAS]; Yucaipa, 0.8km W junction Grape Ave & Bryant St, 34.0641, -117.0425^1^, 853m, W Icenogle 3.ii.2000 [AP363, 1♂, CAS]; Yucaipa, Grape Rd, housing development, 34.065, -117.0422^1^, 852m, J Bond 17.xii.1997 [AP703, 005, 708, 1♂, 2♀, AUMNH]; Yucaipa, Grape St, 0.6km from intersection Grape & Bryant, 34.0653, -117.0425^1^, 863m, J Bond, W Icenogle 28.i.2004 [MY2526, 1♀, AUMNH].

**Variation, males (10).** Cl 5.25-5.90, 5.54±0.05; Cw 4.38-4.88, 4.61±0.05; STRl 3.00-3.57, 3.23±0.05; STRw 2.46-2.79, 2.65±0.03; LBw 0.78-0.96, 0.88±0.02; LBl 0.42-0.68, 0.54±0.03; leg I: 4.81-5.56, 5.24±0.07; 3.45-3.88, 3.67±0.05; 3.33-3.81, 3.56±0.05; 2.10-2.37, 2.25±0.03; 1.86-2.16, 2.01±0.03; leg IV: 4.88-5.56, 5.25±0.07; 2.19-2.88, 2.60±0.06; PTl 2.25-2.52, 2.38±0.03; PTw 0.99-1.05, 1.03±0.01; Bl 1.20-1.38, 1.31±0.02; TSp 3-6, 3.30±0.30; TSr 1-2, 1.70±0.15; TSrd 4-6, 5.00±0.26.

**Variation, females (10).** Cl 5.38-7.50, 6.60±0.25; Cw 4.13-6.13, 5.33±0.22; STRl 3.27-5.06, 4.08±0.19; STRw 2.46-3.69, 3.29±0.13; LBw 0.92-1.37, 1.11±0.05; LBl 0.50-0.78, 0.65±0.03; Leg I: 12.01-17.13, 15.02±0.53; ANTd 6-8, 6.80±0.20; PTLs 7-14, 10.60±0.56; TBs 3-5, 3.90±0.28.

#### GenBank accessions.

16S-tRNAval-12S: JX103313-JX103318

#### Distribution and natural history.

**Map 20, 21.**
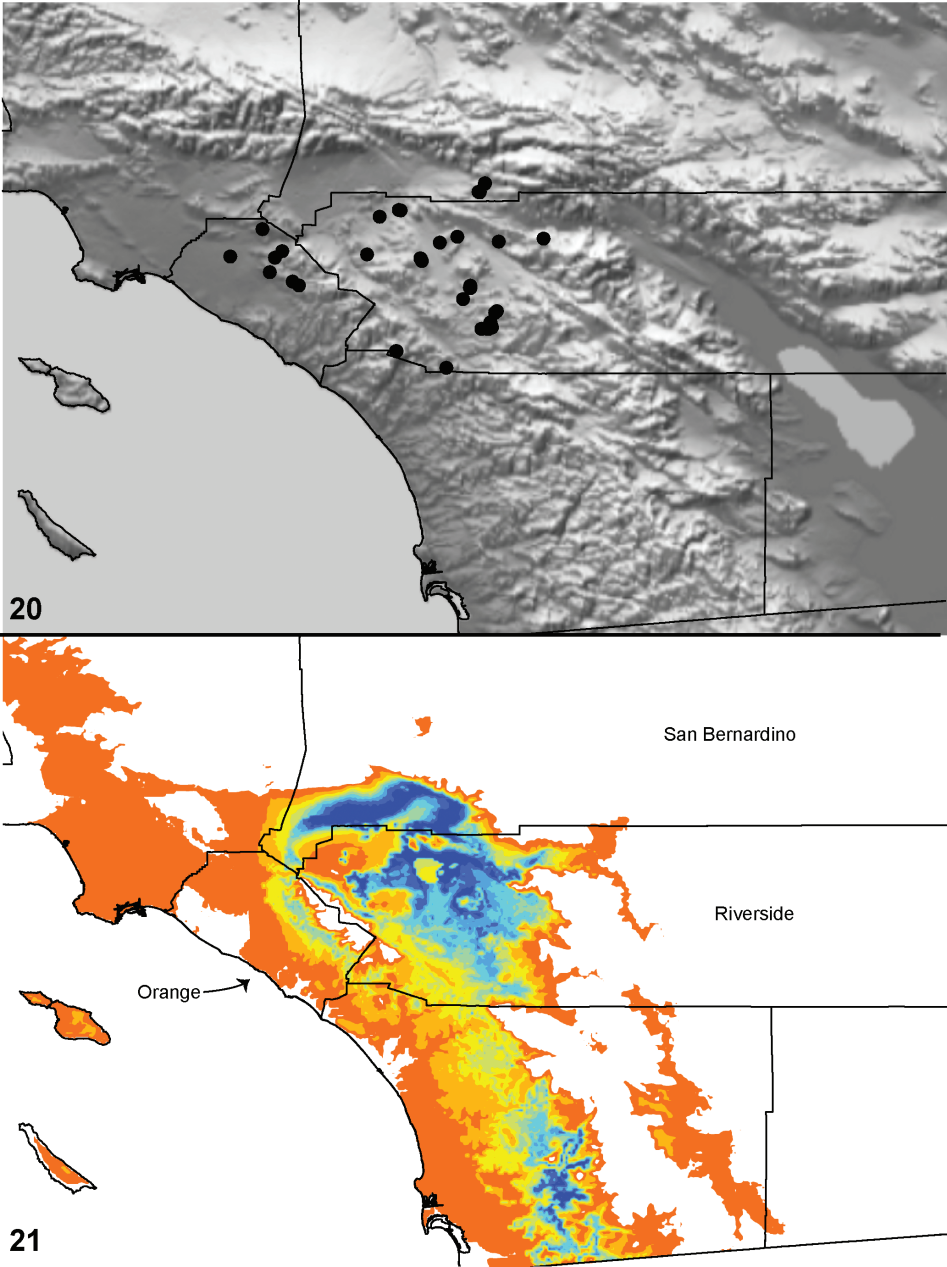
*Aptostichus hesperus* Chamberlin, 1919. **20** distribution of known specimens **21** predicted distribution; cooler colors–blue shades–represent areas of high probability of occurrence, warmer colors–yellow and orange shades–represent areas of low probability of occurrence.

*Aptostichus hesperus* is distributed in the Santa Ana, San Jacinto, San Bernardino Mountains, and intervening areas. County records include Orange, the western extent of Riverside, and a few localities in San Bernardino (Map 20). The primary habitat type throughout this area comprises chaparral forest and shrub, open woodland shrub, and coniferous forest. *Aptostichus hesperus* is particularly prevalent in chaparral habitat and is often collected from burrows at the base of large boulders and along steep inclines of north facing slopes. The DM (Map 21) corresponds well with the known distribution but indicates that *Aptostichus hesperus* should be more prevalent throughout open woodland shrub habitat in the southwestern extent of San Bernardino County; the species has not been collected in the areas of higher probability to the south in San Diego County. Although a few males have been recovered from pitfall traps in the spring and summer, the majority of wandering males have been collected during late fall and winter months (October-February).

#### Conservation status.

This species is relatively abundant across its range thus the conservation status of *Aptostichus hesperus* is likely secure.

#### Species concept applied.

Morphological/Phylogenetic.

#### Remarks.

*Aptostichus hesperus* is one of the easier species to identify because of its unique sternal sigilla morphology and its range is relatively restricted in Orange and Riverside Counties. However, all male specimens collected as part of the USGS survey in the southernmost extent of the species distribution in Riverside County lack a foveal groove. Although this form seems anomalous, the fact that multiple individuals from a number of populations share it suggests that it may be a diagnostic feature that would set these populations aside as a separate species. However, I have conservatively included these specimens as part of the *Aptostichus hesperus* hypothesis until molecular data are available to confirm that these morphological distinct populations are an exclusive lineage.

### Aptostichus hedinorum sp. n.

‘The Hedin Family Trapdoor Spider’

urn:lsid:zoobank.org:act:E0119E3C-6BE4-428A-B46A-80EF03F8BC64

http://species-id.net/wiki/Aptostichus_hedinorum

Figures 197–203 Map 22

#### Types.

Male holotype (MY3779) and three male paratypes (AP1279) from California, San Diego County, Anza-Borrego Desert State Park (ABSP), 0.4km N Hayden Spring, 32.71045, -116.11705^1^, 350m, coll. M. Hedin 14–15.x.2000; female paratype (AP675) from ABSP, along HWY 78 in wash by road, 33.13360 -11634083^1^, coll. J. Bond 12.xii.1998; deposited in AUMNH.

#### Etymology.

The specific epithet is a patronym in honor of the Hedin family, Marshal, Robin, Lars, and Molly.

#### Diagnosis.

**Figures 197–203.**
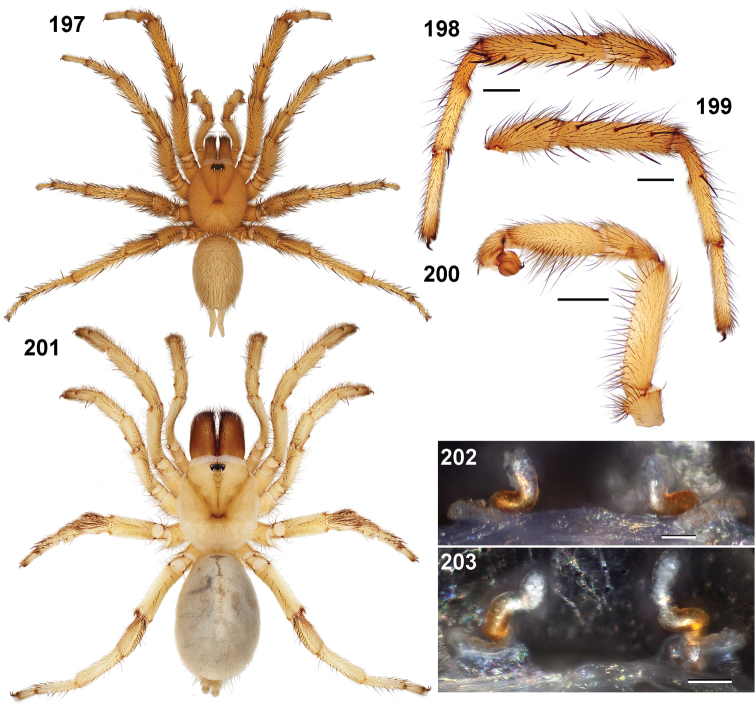
*Aptostichus hedinorum* sp. n. **197–200** male holotype (MY3779); scale bars = 1.0mm **197** habitus [806056] **198** retrolateral aspect, leg I [806048] **199** prolateral aspect, leg I [806052] **200 **pedipalp, leg I [806054] **201** female habitus (MY3781), from type locality [806058] **202, 203** cleared spermathecae, female paratype (AP675) [806608] and additional specimen from type locality (MY3781) [806702]; scale bars = 0.1mm.

Males (Fig. 197) can be diagnosed on the basis of a unique conformation of the distal most spination pattern of tibia I, which consists of 2-3 long spines that typically do not overlap (Fig. 198). This spination pattern is most similar to *Aptostichus icenoglei*, however *Aptostichus hedinorum* males lack a rectangular mating apophysis, are lighter in coloration, have very light dorsal abdominal markings or lack markings altogether, and have a prolaterally offset rastellar spine.

#### Description of male holotype.

*Specimen preparation and condition*. Specimen collected live, wandering, preserved in 80% EtOH. Coloration and specimen in relatively good condition. Pedipalp, leg I left side removed, stored in vial with specimen. *General coloration*. Carapace, chelicerae, brownish yellow 10YR 6/6. Abdomen light yellowish brown 10YR 6/4, lacking dorsal markings (Fig. 197). *Cephalothorax*. Carapace 4.20 long, 3.40 wide, very lightly hirsute with intermingled thin white, black setae; stout black bristles along fringe; surface smooth, pars cephalica elevated. Fringe, posterior margin with black bristles. Foveal groove deep, only slightly procurved. Eyes on low mound. AER slightly procurved, PER slightly recurved. PME, AME subequal diameter. Sternum moderately setose, STRl 2.40, STRw 1.90. Posterior sternal sigilla moderate in size, positioned towards margin, not contiguous, anterior sigilla pairs small, oval, marginal. Chelicerae with distinct anterior tooth row comprising 6 teeth, posterior margin with patch of small denticles. Palpal endites with patch of small cuspules on proximal, inner margin, labium with 3 cuspules, LBw 0.68, LBl 0.34. Rastellum consists of 5 stout spines not on prominent mound, two prominent thin spines offset prolaterally. *Abdomen*. Setose, heavy black setae intermingled with fine black setae. *Legs*. Leg I: 4.16, 3.10, 2.88, 2.15, 1.28; leg IV: 4.40, 2.45. Tarsus IV curved. Light tarsal scopulae on all legs, light scopulae on metatarsus I, II. Tarsus I with single, slightly staggered row of 10 trichobothria. Leg I spination pattern illustrated in Figures 198, 199; TSp 3, TSr 5, TSrd 2. *Pedipalp*. Articles slender, lacking distinct spines (Fig. 200). PTw 0.54, PTl 1.64, Bl 0.87. Embolus slender, tapering sharply toward tip, lacking serrations (Fig. 200).

**Variation (5).** Cl 4.20-4.56, 4.34±0.07; Cw 3.40-3.68, 3.56±0.05; STRl 2.40-2.60, 2.53±0.04; STRw 1.90-2.06, 1.99±0.03; LBw 0.68-0.77, 0.71±0.02; LBl 0.34-0.37, 0.36±0.01; leg I: 4.16-4.28, 4.24±0.02; 3.10-3.2, 3.16±0.02; 2.88-2.98, 2.95±0.02; 2.10-2.25, 2.16±0.03; 1.28-1.40, 1.36±0.02; leg IV: 4.40-4.68, 4.52±0.06; 2.44-2.50, 2.47±0.01; PTl 1.64-1.70, 1.66±0.01; PTw 0.54-0.60, 0.56±0.01; Bl 0.80-0.87, 0.84±0.01; TSp 3-4, 3.40±0.24; TSr 3-5, 4.00±0.32; TSrd 2-3, 2.40±0.24.

#### Description of female paratype.

*Specimen preparation and condition*. Female collected live from burrow, prepared in same manner as male holotype. Genital plate removed, cleared in trypsin, stored in microvial with specimen. *General coloration*. Carapace, legs, chelicerae, pale brown 10YR 6/4. Abdomen grayish brown dorsally 10YR 5/2, lacks distinct mottled chevron marking pattern (Fig. 201). *Cephalothorax*. Carapace 4.95 long, 4.40 wide, generally glabrous, very sparse fine black setae; generally smooth surface, pars cephalica moderately elevated. Fringe lacks setae. Foveal groove deep, slightly procurved. Eye group slightly elevated on very low mound. AER slightly procurved, PER slightly recurved. PME-AME subequal diameter. Sternum widest at coxae II/III, moderately setose, STRl 2.98, STRw 2.48. Three pairs of sternal sigilla anterior pairs small in size, oval, marginal; posterior pair moderate in size, oval, mesially positioned but not contiguous. Chelicerae anterior tooth row comprising 6 teeth with posterior margin denticle patch. Palpal endites with 30 cuspules concentrated at the inner (promargin) posterior heel; labium with 3 cuspules, LBw 1.02, LBl 0.51. Rastellum consists of 7 very stout spines not positioned on mound, two prominent spines offset prolaterally; fringe of short spines along distal promargin extending upward from rastellum. *Abdomen*. Moderately setose. PLS all 3 segments with spigots. Terminal segment 1/2 length of medial segment, 2 enlarged spigots visible at tip. PMS single segment, with spigots, short with rounded terminus. *Legs*. Anterior two pairs noticeably more slender than posterior pairs. Leg I 10.95 long. Tarsus I with 6 trichobothria arranged in straight row. Legs I, II with moderately heavy scopulae on tarsus, metatarsus; light scopulae on distal aspect tarsus legs III, IV. PTLs 7, TBs 4. Rudimentary preening comb on retrolateral distal surface, tarsus-metatarsus joint, of metatarsus III, IV. *Spermathecae*. 2 simple spermathecal bulbs with an elongate curved stalk; basal extension small (Figs 202, 203).

**Variation (4).** Cl 4.20-5.00, 4.68±0.19; Cw 3.76-4.40, 4.18±0.14; STRl 2.63-3.04, 2.92±0.10; STRw 2.18-2.48, 2.36±0.06; LBw 0.80-1.02, 0.94±0.05; LBl 0.48-0.63, 0.54±0.03; Leg I: 10.8-12.26, 11.46±0.35; ANTd 6-6, 6.00±0.00; PTLs 7-10, 8.25±0.75; TBs 3-5, 3.75±0.48.

#### Material examined.

**United States: California: San Diego Co.:** Anza-Borrego Desert St Park, 0.4km N Hayden Spring, 32.7104, -116.117^1^, 350m, M Hedin 14.x.2000 [AP1214, AP1218, AP1279, MY3779, MY3781, 2♀, 9♂, 1juv, AUMNH]; ABSP, ~0.8 NE Hayden Springs, 32.71118, -116.116^1^, 488m, M Hedin 19.x.2002 [AP1213, AP1282, MY264-647, 669, 5♂, 3♀, AUMNH], M Hedin, J Bond 10.i.2002 [MY265-269, 4♀, AUMNH]; Anza-Borrego Desert St Park, along hwy 78 wash by road, 33.1336, -116.3408^1^, 351m, J Bond 12.xii.1997 [AP675, 681, 684, 1♀, 2juv, AUMNH].

#### GenBank accessions.

16S-tRNAval-12S: JX103302-JX103312

#### Distribution and natural history.

*Aptostichus hedinorum* is known from Anza-Borrego Desert State Park in San Diego County within the Colorado Desert (Map 21). Male specimens have been collected during October. Female burrows can often be found in sandy banks and washes during the winter months when individuals excavate and presumably extend their burrows, creating a telltale mound at the burrow entrance.

**Map 22.**
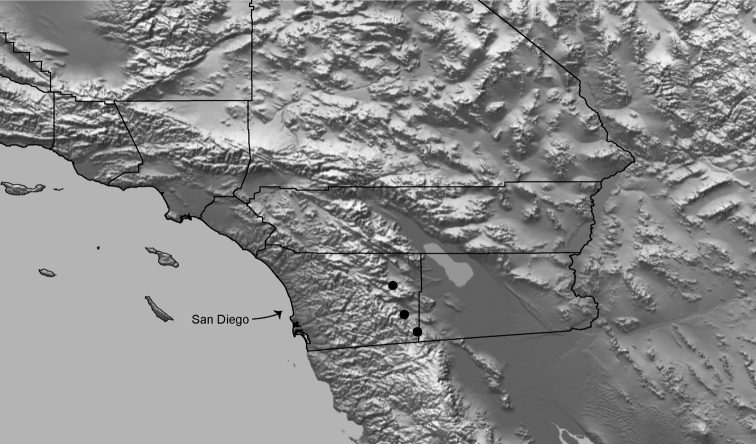
*Aptostichus hedinorum* Bond sp. n., distribution of known specimens.

#### Conservation status.

The conservation status of *Aptostichus hedinorum* is considered imperiled given its very restricted distribution.

#### Species concept applied.

Morphological/Phylogenetic.

### Aptostichus cahuilla sp. n.

‘The Winchester Trapdoor Spider’

urn:lsid:zoobank.org:act:138FA005-949E-45D0-8C95-2F418133AC8D

http://species-id.net/wiki/Aptostichus_cahuilla

**Figures 204–209.**
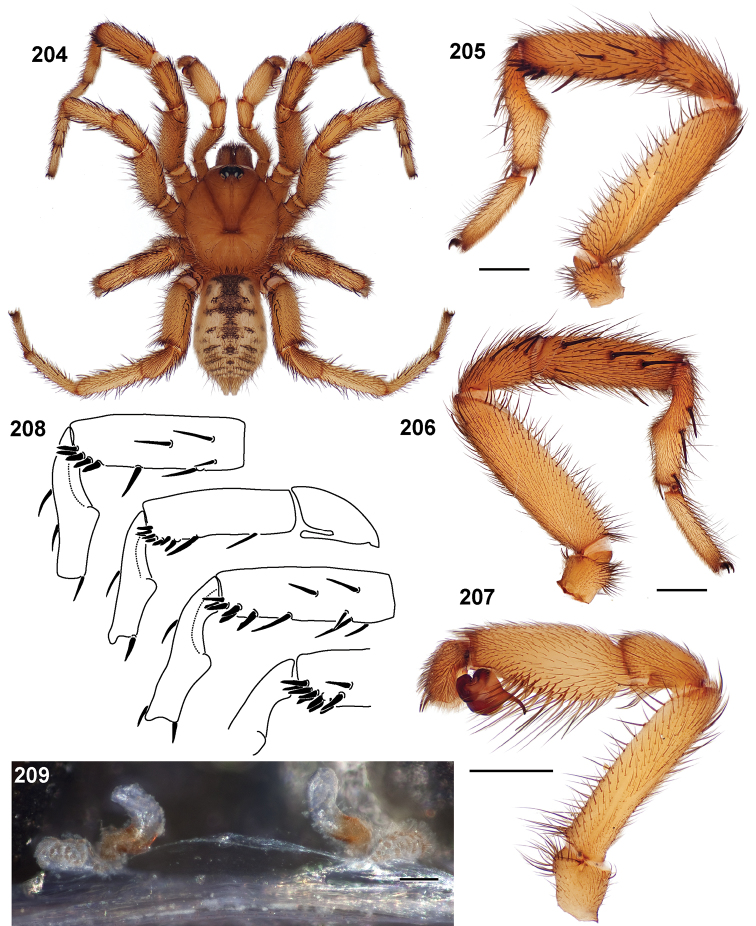
*Aptostichus cahuilla* sp. n. **204–207** male holotype from Riverside County, Winchester (AP392); scale bars = 1.0mm **204** habitus [805952] **205** retrolateral aspect, leg I [805944] **206 **prolateral aspect, leg I [805948] **207** retrolateral aspect, pedipalp [805950] **208** line drawings of male leg I tibia and metatarsus spination pattern variation **209** cleared spermathecae, female paratype (AP221) [806626]; scale bar = 0.1mm.

**Maps 23, 24.**
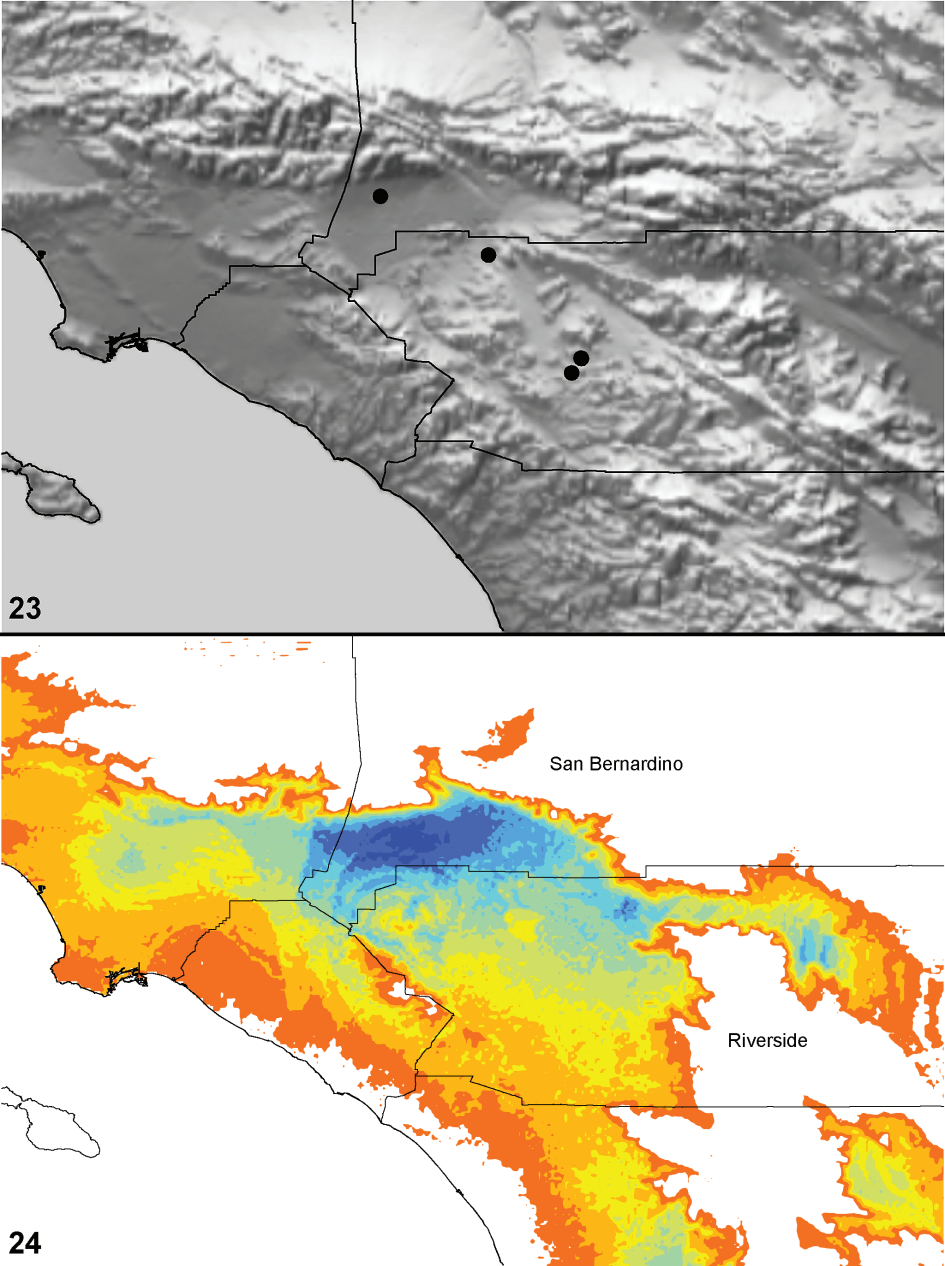
*Aptostichus cahuilla* Bond sp. n. **23** distribution of known specimens **24** predicted distribution; cooler colors–blue shades–represent areas of high probability of occurrence, warmer colors–yellow and orange shades–represent areas of low probability of occurrence.

Figures 204–209 Map 23, 24

#### Types.

Male holotype (AP392) and female paratype (AP221) from California, Riverside County, Winchester, 1.6km NW of town center, vicinity of Double Butte, 33.714924, -117.092205^1^, 478m, coll. W. Icenogle 3.ix.1967 & 15.x.1967, deposited in AUMNH.

#### Etymology.

The specific epithet is a noun in apposition taken from the Cahuilla Native American Tribal Group, which once resided in Southern California.

#### Diagnosis.

Males (Fig. 204) can be diagnosed on the basis of a unique conformation of the distal - most spination pattern of tibia I which consists of 5-9 short spines that are always overlapping (Figs 205, 208). This spination pattern is most similar to *Aptostichus derhamgiulianii*, however the retrolateral cymbium surface of *Aptostichus derhamgiulianii* bears a number of small, distinct spines, whereas that of *Aptostichus cahuilla* does not. Additionally, the MA4/MF4 ratio (Fig. 19) of *Aptostichus derhamgiulianii* is greater than that of *Aptostichus cahuilla* (i.e., the overall length of tarsus IV is greater for *Aptostichus derhamgiulianii*). Females can be distinguished from those of other known sympatric species of *Aptostichus* (*Aptostichus icenoglei*, *Aptostichus hesperus*, and *Aptostichus atomarius*) simply by their small size (Cl < 4.6). Additional features that distinguish females of this species from others that are closely related (*Aptostichus hesperus* and *Aptostichus aguacaliente*) is the presence of smaller sigilla that tend to be more widely spaced. Males can be further diagnosed on the basis of a greater PTw/PTl ratio (Fig. 23).

#### Description of male holotype.

*Specimen preparation and condition*. Specimen collected live from pitfall trap, preserved in 80% EtOH. Coloration moderately faded. Pedipalp, leg I left side removed, stored in vial with specimen. *General coloration*. Carapace, chelicerae, legs strong brown 7.5YR 4/6; dusky purple markings along anterior margins of carapace. Abdomen uniform yellowish brown 10YR 5/4 dorsally, ventrum, spinnerets pale yellow; mottled chevron pattern (Fig. 204). *Cephalothorax*. Carapace 3.88 long, 3.28 wide, lightly hirsute, stout black bristles along fringe; surface smooth, pars cephalica elevated. Fringe, posterior margin with black bristles. Foveal groove deep, straight. Eyes on mound. AER slightly procurved, PER slightly recurved. PME slightly larger in diameter than AME. Sternum moderately setose, STRl 2.00, STRw 1.84. Posterior sternal sigilla small, positioned laterally, anterior sigilla pairs small, oval, marginal. Chelicerae with distinct anterior tooth row comprising 5 teeth, posterior margin with single row of small denticles. Palpal endites with patch of small cuspules on proximal, inner margin, labium with few cuspules, LBw 0.68, LBl 0.43. Rastellum consists of 5 very stout spines, one spine separated laterally. *Abdomen*. Setose, heavy black setae intermingled with fine black setae. *Legs*. Leg I: 3.76, 2.60, 2.29, 1.55, 1.30; leg IV: 3.52, 1.70. Light tarsal scopulae on legs I, II. Tarsus I with single, slightly staggered row of 10 trichobothria. Leg I spination pattern illustrated in Figures 205, 206, 208; TSp 3, TSr 3, TSrd 5. *Pedipalp*. Articles stout, lacking distinct spines (Figs 207). PTw 0.80, PTl 1.68, Bl 0.85. Embolus slender, with slight curvature at terminus (Fig. 207).

**Variation (9).** Cl 3.42-4.38, 3.89±0.09; Cw 2.79-3.63, 3.11±0.08; STRl 1.92-2.46, 2.09±0.05; STRw 1.65-2.13, 1.84±0.05; LBw 0.57-0.69, 0.64±0.01; LBl 0.30-0.44, 0.37±0.02; leg I: 3.18-4.13, 3.60±0.10; 2.16-2.94, 2.52±0.08; 1.92-2.40, 2.20±0.05; 1.32-1.68, 1.55±0.04; 1.14-1.38, 1.27±0.02; leg IV: 3.00-3.75, 3.38±0.09; 1.5-1.81, 1.65±0.04; PTl 1.44-1.79, 1.62±0.04; PTw 0.72-0.81, 0.77±0.01; Bl 0.75-0.90, 0.81±0.02; TSp 3-5, 3.78±0.28; TSr 2-5, 4.11±0.35; TSrd 5-9, 6.22±0.46.

#### Description of female paratype.

*Specimen preparation and condition*. Female collected live from burrow, prepared in same manner as male holotype. Genital plate removed, cleared in trypsin, stored in microvial with specimen. *General coloration*. Carapace, legs, chelicerae, strong brown 7.5YR 4/6. Abdomen uniform dark grayish brown dorsally 10YR 4/2 with mottled striping, ventral, spinnerets pale yellow; more recently collected specimens much darker in color. *Cephalothorax*. Carapace 4.55 long, 3.64 wide, glabrous; generally smooth surface, pars cephalica moderately elevated. Fringe lacks setae. Foveal groove deep, slightly procurved, straight in most specimens. Eye group slightly elevated on low mound. AER slightly procurved, PER slightly recurved. PME-AME subequal diameter. Sternum widest at coxae II/III, moderately setose, STRl 2.58, STRw 2.15. Three pairs of sternal sigilla anterior pairs small, oval, marginal, posterior pair small, oval-suboval, mid-marginally positioned. Chelicerae anterior tooth row comprising 5 teeth with posterior margin denticle patch comprising 3 rows. Palpal endites with 27 cuspules concentrated at inner (promargin) posterior heel; labium with 6 cuspules, LBw 1.25, LBl 0.41. Rastellum consists of 5 very stout spines not on mound, one spine positioned laterally; fringe of short spines along distal promargin extending upward from rastellum. *Abdomen*. Moderately setose, appears elongate relative to other species. PLS all 3 segments with spigots. Terminal segment 1/2 length of medial segment, 2 enlarged spigots visible at tip. PMS single segment, with spigots, short with rounded terminus. *Legs*. Anterior two pairs noticeably more slender than posterior pairs. Leg I 10.08 long. Tarsus I with single staggered row of 12 trichobothria. Legs I, II with moderately heavy scopulae on tarsi, metatarsi; light sparse scopulae on legs III, IV tarsi, PTLs 8, TBs 3. Rudimentary preening comb on retrolateral distal surface at tarsus - metatarsus joint of metatarsus III, IV. *Spermathecae*. Simple spermathecal bulbs with elongate, heavily sclerotized median stalk neck, sinuous; basal extension comprises a small bulb (Fig. 209).

**Variation (4).** Cl 3.47-4.55, 3.98±0.22; Cw 2.98-3.8, 3.45±0.18; STRl 2.00-2.58, 2.30±0.12; STRw 1.72-2.15, 1.98±0.1; LBw 0.75-1.25, 0.91±0.12; LBl 0.34-0.51, 0.41±0.04; Leg I: 7.73-10.08, 8.96±0.51; ANTd 5-6, 5.25±0.25; PTLs 8-10, 9.00±1.00; TBs 2-3, 2.50±0.50.

#### Material examined.

**United States: California: Riverside Co.:** just S Winchester on Leona Rd, ~1.6km S intersection w/Patton Ave, 33.6771, -117.1157^1^, 444m, J Bond 01.ii.2004 [MY2521, 1juv, AUMNH]; Winchester, 33.7138, -117.0915^1^, 470m, W Icenogle 18.i.1970 [AP245, 1♂, AMNH]; Winchester, 1.6km NW of town center, vicinity of Double Butte, 33.7138, -117.0913^1^, 466m, J Bond, W Icenogle 25.iii.1996 [AP737, 1♀, AUMNH]; Winchester, 1.6km NW of town center, vicinity of Double Butte, 33.7148, -117.0922^1^, 476m, J Bond 29.i.1997 [AP1203, 1♀, AUMNH], W Icenogle 23.ii.1973 [AP246, 1♂, AMNH], 05.i.1977 [AP2471♂AMNH], 27.xii.1977 [AP241, 1♂, CAS], 03.ix.1967 [AP392, 1♀, 1♂, CAS], 15.x.1967 [AP221, 1♀, 29juv, AMNH], 20.xi.1967 [AP227, 1♂, AMNH], 04.ii.1968 [AP240, 1♂, AMNH], 17.xi.1972 [AP238, 1♂, AMNH]; University of California, Riverside Campus, 33.9742, -117.3251^3^, 327m, W Icenogle 05.x.1967 [AP232, 1♀, 12juv, AMNH], 10.x.1967 [AP222, 1♀, 31juv, CAS], 27.x.1967 [AP239, 1♂, AMNH]; **San Bernardino Co.:** Alta Loma, 34.122, -117.597^3^, 412m, D Bixler 04.iv.1969 [AP337, 1♂, AMNH], 15.iv.1969 [AP338, 1♂, AMNH], 20.iv.1969 [AP336, 1♂, AMNH].

#### GenBank accession.

16S-tRNAval-12S: JX103293

#### Distribution and natural history.

*Aptostichus cahuilla* is known from only from a few localities in Riverside County and one in southwestern San Bernardino County. Experience collecting this species indicates that it is found primarily in chaparral habitat but is considerably less abundant than the species with which it is sympatric (e.g., *Aptostichus atomarius*, *Aptostichus icenoglei*, and *Aptostichus hesperus*).

#### Conservation status.

The conservation status of *Aptostichus cahuilla* is likely to be considered imperiled; it is rare in collections, abundance is very low, and it is relatively restricted in distribution.

#### Species concept applied.

Morphological/Phylogenetic.

### Aptostichus killerdana sp. n.

‘The Killerdana Trapdoor Spider’

urn:lsid:zoobank.org:act:FF96A374-08CA-41BA-A2A5-F15CF547A177

http://species-id.net/wiki/Aptostichus_killerdana

**Figures 210–214.**
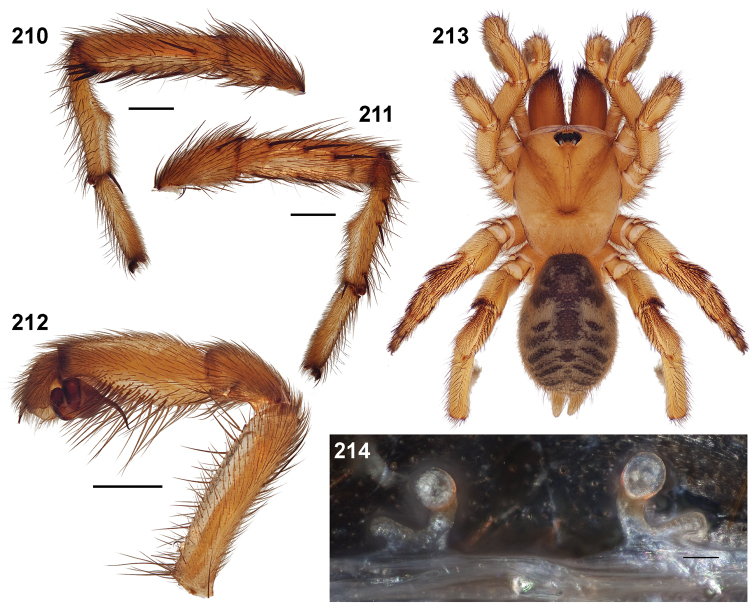
*Aptostichus killerdana* sp. n. (AP432) from Orange County. **210–212** male paratype; scale bars = 1.0mm **210** retrolateral aspect, leg I [806062] **211** prolateral aspect, leg I [806066] **212 **retrolateral aspect, pedipalp [806068] **213, 214** female holotype **213** habitus [806070] **214** cleared spermathecae [806703]; scale bar = 0.1mm.

**Maps 25, 26.**
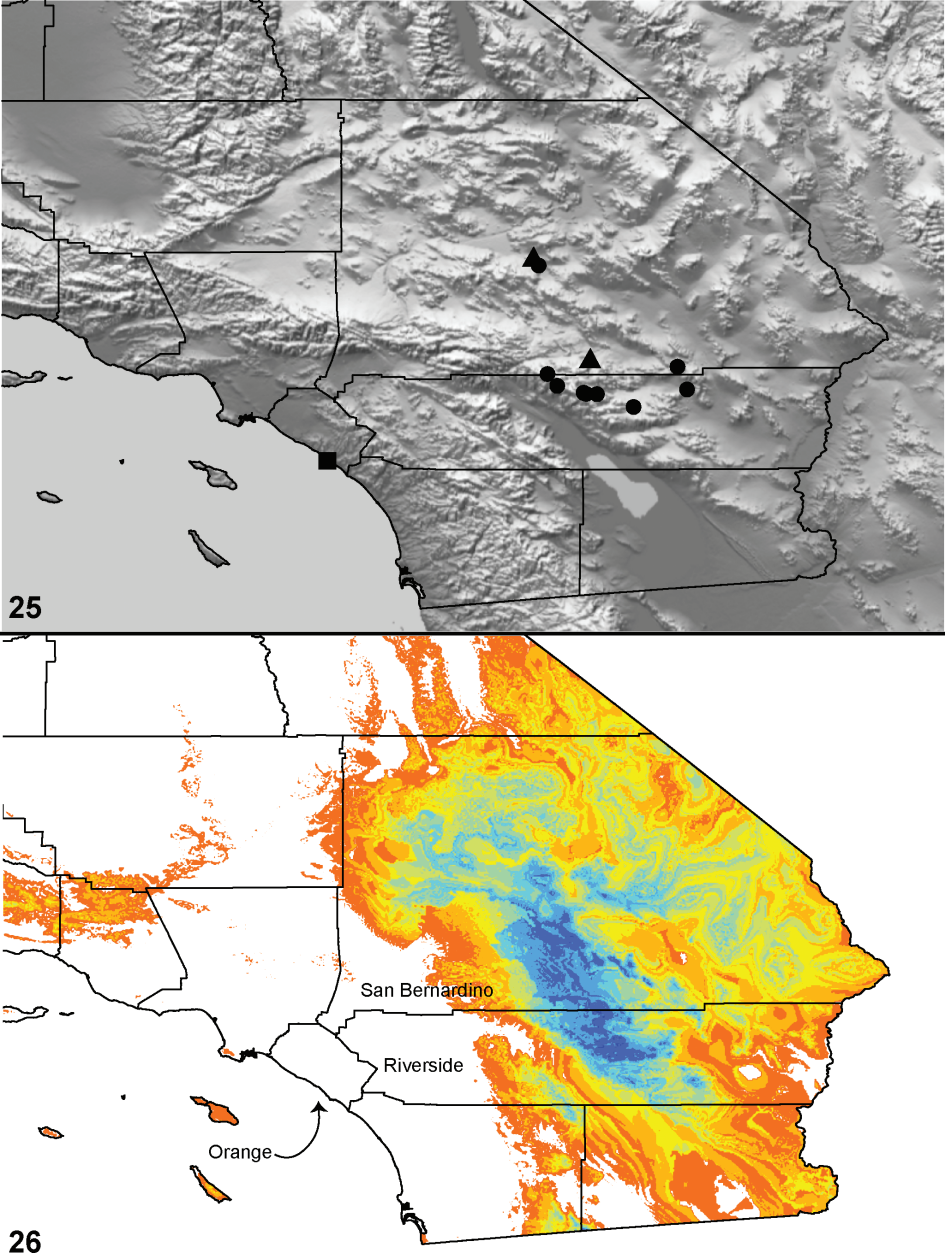
*Aptostichus killerdana* sp. n. (filled square), *Aptostichus serrano* sp. n. (filled circles), *Aptostichus chemehuevi* sp. n. (filled triangles). **25** known distributions **26** predicted distribution of *Aptostichus serrano*; cooler colors–blue shades–represent areas of high probability of occurrence, warmer colors–yellow and orange shades–represent areas of low probability of occurrence.

Figures 210–214 Map 25

#### Types.

Female holotype and male paratype (AP432) from California, Orange County, Salt Creek, 2.4km North of Dana Point, 33.4819, -117.7206^3^, 21m, coll. W. Icenogle 14.xi.1969, deposited in the AMNH.

#### Etymology.

The specific epithet is a noun in apposition taken from the name of the legendary surf break, “Killer Dana”, that once graced the shore of Dana Point. The break was destroyed in the mid 1960’s by the construction of the breakwater for Dana Point Harbor.

#### Diagnosis.

Males can be distinguished from other known related species of *Aptostichus* (e.g., *Aptostichus cahuilla*) by virtue of a unique tibia I TSrd spination pattern that comprises only a few spines (Fig. 210). In contrast, the spination pattern of *Aptostichus cahuilla* is formed of many overlapping distal spines. Females (Fig. 213) can be distinguished on the basis of having long median spermathecal stalks that do not curve as extensively and a median bulb that is larger (Fig. 214) than that of *Aptostichus cahuilla*; specimens also tend to have a larger number of labial cuspules (> 3).

#### Description of female holotype.

*Specimen preparation and condition*. Female collected live from burrow, preserved in 70% EtOH. Genital plate removed, cleared in trypsin, stored in microvial with specimen. *General coloration*. Carapace, legs, chelicerae, dark yellowish brown 10YR 4/6. Abdomen lighter in color with distinct mottled striping (Fig. 213), ventral aspect, spinnerets pale yellow. *Cephalothorax*. Carapace 4.75 long, 4.10 wide, generally glabrous with few light dark, thin setae; smooth surface, pars cephalica moderately elevated. Fringe lacks setae, with dark purple coloration along margins. Foveal groove deep, slightly procurved to straight. Eye group elevated on low mound. AER slightly procurved, PER slightly recurved. PME, AME subequal diameter. Sternum widest at coxae II/III, moderately setose, STRl 3.08, STRw 2.65. Three pairs of sternal sigilla anterior pairs small, oval, marginal, posterior small, somewhat irregular shaped/elongate, positioned at midpoint from margin to center. Chelicerae anterior tooth row comprising 6 teeth with posterior margin denticle patch comprising two rows. Palpal endites with 28 cuspules concentrated at the inner (promargin) posterior heel; labium with 7 cuspules, LBw 0.97, LBl 0.51. Rastellum consists of 7 stout spines not positioned on mound, one spine separated laterally; fringe of short spines along distal promargin extending upward from rastellum. *Abdomen*. Moderately setose. PLS all 3 segments with spigots. Terminal segment 1/2 length of medial segment, 2 enlarged spigots visible at tip. PMS single segment, with spigots, short with rounded terminus. *Legs*. Anterior two pairs noticeably more slender than posterior pairs. Leg I 10.63 long. Tarsus I with single staggered row of 11 trichobothria. Pedipalp, leg I moderately heavy scopulae, lighter on leg II; extends to distal aspect of leg I tibia, only on tarsus, metatarsus of leg II; sparse scopulae on tarsus of legs III, IV. PTLs 16, TBs 3. Rudimentary preening comb on retrolateral distal surface (at tarsus - metatarsus joint) of metatarsus III, VI. *Spermathecae*. Simple, small spermathecal bulbs with elongate sclerotized stalk, with small laterally basal extension (Fig. 214).

**Variation (3).** Cl 4.20-4.85, 4.60±0.20; Cw 3.45-4.10, 3.88±0.22; STRl 2.70-3.18, 2.99±0.15; STRw 2.00-2.65, 2.38±0.2; LBw 0.80-1.00, 0.92±0.06; LBl 0.50-0.60, 0.54±0.03; Leg I: 9.10-10.75, 10.16±0.53; ANTd 5-6, 5.67±0.33; PTLs 9-16, 11.33±2.33; TBs 2-3, 2.67±0.33.

#### Description of male paratype.

*Specimen preparation and condition*. Specimen collected live from burrow, preserved in 70% EtOH but subsequently dried; generally in poor condition. Pedipalp, leg I left side removed, stored in vial with specimen, molt removed from burrow bottom. *Cephalothorax*. Carapace 4.24 long, 3.56 wide, lightly hirsute with thin white spines, stout black bristles along fringe; surface smooth, pars cephalica elevated. Fringe, posterior margin with black bristles. Foveal groove deep, slightly recurved, almost forming a pit. Eyes on low mound. AER slightly procurved, PER slightly recurved. PME, AME subequal diameter. Sternum moderately setose, STRl 2.50, STRw 2.10. Posterior sternal sigilla positioned at margin, not contiguous, anterior sigilla pairs small, oval, marginal. Chelicerae with distinct anterior tooth row comprising 6 teeth, posterior margin with single row of small denticles. Palpal endites with patch of small cuspules on proximal, inner margin, labium with 5 cuspules, LBw 0.82, LBl 0.43. Rastellum consists of 5 stout spines not on prominent mound. *Abdomen*. Setose, heavy black setae intermingled with fine black setae. *Legs*. Leg I: 4.12, 3.10, 2.70, 1.75, 1.58; leg IV: 4.05, 2.10. Light tarsal scopulae on legs I, II. Tarsus I with single, slightly staggered row of 13 trichobothria. Leg I spination pattern illustrated in Figures 210, 211; TSp 3, TSr 1, TSrd 3. *Pedipalp*. Articles stout, lacking distinct spines (Fig. 212). PTw 0.9, PTl 1.9, Bl 1.1. Embolus slender, with slight curvature at tip (Fig. 212).

**Variation.** Known only from the type specimen.

#### Material examined.

**United States: California:** **Orange Co.:** Salt Creek 2.4km N Dana Pt, 33.4819, -117.7206^3^, 21m, W Icenogle 14.xi.1969 [AP432, 1♀, 1♂, AMNH], 12.xi.1969 [AP604, 1♀, 2juv, AMNH], 14.xi.1969 [AP606, 1♀, 10juv, CAS], S Johnson 15ii.1971 [AP607, 1♀, 1juv, AMNH].

#### Distribution and natural history.

*Aptostichus killerdana* is known only from the type locality in Orange County. The species was collected from a relatively low-lying, riparian habitat in the time interval from 1969-1971. Some point thereafter the area was developed and is now Monarch Beach Golf Links; the habitat was cleared and is now a golf course. Although classified as California coastal chaparral and scrub, photographs of the area prior to development seem to indicate that it was a relatively unique and pristine coastal riparian habitat. The location was also the southernmost known locality for *Apomastus kristenae* Bond, 2004, likewise a species that seems to have extirpated from the area. Extensive sampling in the low-lying areas around and above the golf resort has to date proven unsuccessful.

#### Species concept applied.

*Aptostichus killerdana* is presumed extinct.

### Aptostichus serrano sp. n.

‘The Joshua Tree Serrano Trapdoor Spider’

urn:lsid:zoobank.org:act:BE163831-1BAA-42C4-ABE4-7E60B2AE77A0

http://species-id.net/wiki/Aptostichus_serrano

**Figures 215–219.**
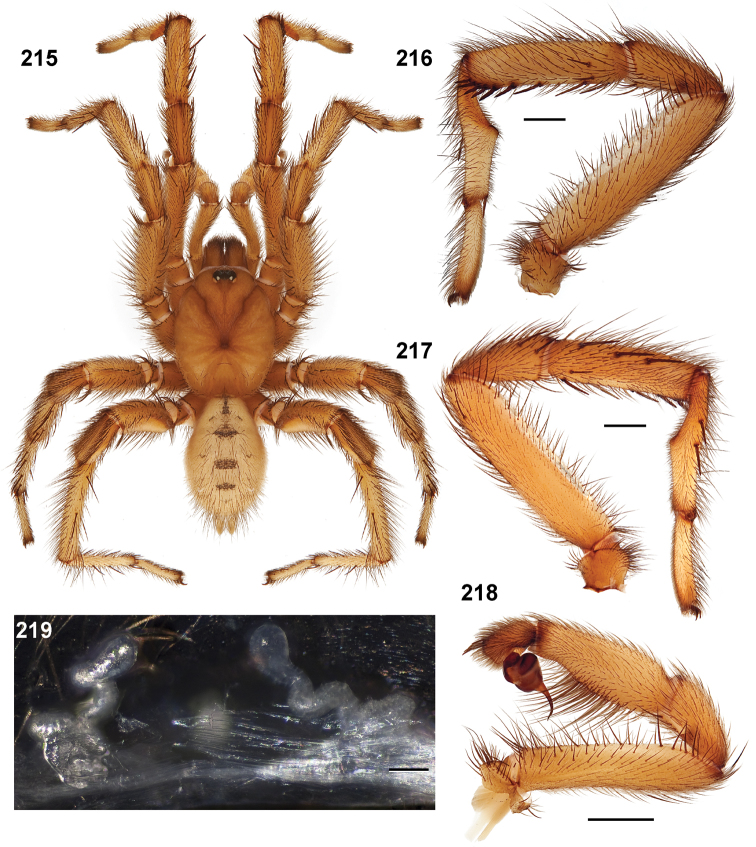
*Aptostichus serrano* sp. n. **215–218** male holotype (AP395); scale bars = 1.0mm. **215** habitus [805962] **216** retrolateral aspect, leg I [805954] **217** prolateral aspect, leg I [805960] **218 **retrolateral aspect, pedipalp [805958] **219** cleared spermathecae (AP712) [806611]; scale bar = 0.1mm.

Figures 215–219 Map 25, 26

#### Types.

Male holotype (AP395) and male paratype (AP396) from California, Riverside County, Joshua Tree National Park, Pleasant Valley, 33.9102, -115.9931^3^, 1066m, coll. E. Sleeper & S. Jenkins 20.iii.1966 & 8.i.1966, deposited in AMNH; Female paratypes (AP712, 714) from California, San Bernardino Co., Joshua Tree National Park, ~2.7km S of bend in road, HWY 62, 34.08667, -115.47778^1^, 684m, coll. J. Bond & W. Icenogle 17.i.1997, deposited in AUMNH.

#### Etymology.

The specific epithet is a noun in apposition taken from the Serrano Native American Tribal group, which once resided throughout what are now the California counties of Riverside and San Bernardino.

#### Diagnosis.

Males (Fig. 215) are easily distinguished from the other known sympatric species of *Aptostichus*, *Aptostichus bonoi*, by lacking spines on the ventral surface of tarsus I (Fig. 216). The TSrd spination of *Aptostichus serrano* (Fig. 216) is most similar to that of *Aptostichus atomarius*, however male *Aptostichus serrano* individuals can be distinguished by having a retrolaterally offset rastellar spine. Female *Aptostichus serrano* individuals can be distinguished from *Aptostichus atomarius* females by virtue of their smaller size and a rastellar configuration similar to that of the males. Male and females are both lighter in coloration and lack the distinct mottled striping of the non-desert species (Fig. 215).

#### Description of male holotype.

*Specimen preparation and condition*. Specimen presumed to have been collected live from a pitfall trap array, preserved in 70% EtOH. Coloration lightly faded. Pedipalp, leg I left side removed, stored in vial with specimen. *General coloration*. Carapace, chelicerae, legs strong brown 7.5YR 4/6. Abdomen dorsally light yellowish brown 10YR 6/4, light mid dorsal stripes (Fig. 215), ventral aspect similar coloration. *Cephalothorax*. Carapace 4.75 long, 4.06 wide, lightly hirsute, thin white spines, stout black bristles along fringe; surface smooth, pars cephalica elevated. Fringe, posterior margin with black bristles. Foveal groove deep, straight, almost forming a pit. Eyes on low mound. AER slightly procurved, PER slightly recurved. PME, AME subequal diameter. Sternum moderately setose, STRl 2.63, STRw 2.40. Posterior sternal sigilla moderate in size, positioned mid to central, not contiguous, anterior sigilla pairs small, oval, marginal. Chelicerae with distinct anterior tooth row comprising 5 teeth, posterior margin with row of small denticles. Palpal endites with patch of small cuspules on proximal, inner margin, labium with 3 cuspules, LBw 0.77, LBl 0.41. Rastellum consists of 5 stout spines not on prominent mound, one spine offset laterally. *Abdomen*. Setose, heavy black setae intermingled with fine black setae. *Legs*. Leg I: 4.85, 3.50, 3.10, 1.90, 1.75; leg IV: 4.69, 2.44. Light tarsal scopulae on legs I, II. Tarsus I with single, staggered row of 11 trichobothria. Leg I spination pattern illustrated in Figures 216, 217; TSp 5, TSr 4, TSrd 6. *Pedipalp*. Articles stout, lacking distinct spines (Fig. 218). PTw 0.80, PTl 2.03, Bl 0.96. Embolus slender, slight curvature at midpoint, lacking serrations (Fig. 218).

**Variation (7).** Cl 4.44-5.00, 4.73±0.1; Cw 3.54-4.13, 3.93±0.08; STRl 2.40-2.76, 2.60±0.05; STRw 1.98-2.40, 2.22±0.05; LBw 0.68-0.83, 0.75±0.02; LBl 0.39-0.45, 0.43±0.01; leg I: 4.50-5.13, 4.81±0.08; 3.25-3.56, 3.39±0.05; 2.73-3.15, 2.95±0.07; 1.80-2.04, 1.90±0.03; 1.50-3.25, 1.89±0.23; leg IV: 4.38-4.88, 4.59±0.07; 2.13-2.44, 2.28±0.04; PTl 1.86-2.10, 2.01±0.03; PTw 0.69-0.80, 0.75±0.01; Bl 0.90-1.02, 0.95±0.01; TSp 3-8, 4.29±0.68; TSr 2-4, 3.00±0.31; TSrd 4-6, 4.71±0.29.

**Description of female paratype (AP712).** *Specimen preparation and condition*. Female collected live from burrow, prepared in same manner as male holotype. Genital plate removed, cleared in trypsin, stored in microvial with specimen. *General coloration*. Carapace, legs, chelicerae, yellowish brown 10YR 5/4. Abdomen uniform pale brown 10YR 6/3, markings similar to males; recently collected specimens slightly darker in coloration. *Cephalothorax*. Carapace 4.05 long, 3.60 wide, generally glabrous with light thin setae; generally smooth surface, pars cephalica moderately elevated. Fringe lacks setae. Foveal groove deep, slightly procurved. Eye group slightly elevated on low mound. AER slightly procurved, PER slightly recurved. PME-AME subequal diameter. Sternum widest at coxae II/III, moderately setose, STRl 2.63, STRw 2.29. Three pairs of sternal sigilla anterior pairs small, oval, marginal, posterior pair larger, oval, mesially positioned but not contiguous. Chelicerae anterior tooth row comprising 6 teeth with posterior margin denticle patch. Palpal endites with 23 cuspules concentrated at inner (promargin) posterior heel; labium with 3 cuspules, LBw 0.95, LBl 0.49. Rastellum consists of 6 very stout spines positioned not on mound, one spine offset laterally; fringe of short spines along distal promargin extending upward from rastellum. *Abdomen*. Moderately setose. PLS all 3 segments with spigots. Terminal segment 1/2 length of medial segment, 2 enlarged spigots visible at tip. PMS single segment, with spigots, short with rounded terminus. *Legs*. Anterior two pairs noticeably more slender than posterior pairs. Leg I 10.44 long. Tarsus I with single staggered row of 10 trichobothria. Legs I, II, with moderately light scopulae on tarsi, metatarsi, legs III, IV with light tarsal scopulae. PTLs 9, TBs 3. Rudimentary preening comb on retrolateral distal surface (at tarsus - metatarsus joint) of metatarsus III, IV. *Spermathecae*. 2 simple spermathecal bulbs with elongate neck, small lateral basal extension (Fig. 219).

**Variation (5).** Cl 3.52-5.00, 4.29±0.31; Cw 2.73-4.20, 3.60±0.28; STRl 2.30-3.10, 2.70±0.16; STRw 1.70-2.50, 2.18±0.15; LBw 0.77-1.00, 0.92±0.04; LBl 0.44-0.51, 0.48±0.02; Leg I: 8.45-12.23, 10.67±0.73; ANTd 5-6, 5.80±0.22; PTLs 8-12, 10.2±0.89; TBs 2-4, 3.00±0.35.

#### Material examined.

**United States: California: Riverside Co.:** Joshua Tree Natl Park, Turkey Flats, jct main rd & Black Eagle Rd, 33.8283, -115.7586^1^, 738m, J Bond 11.xii.1997 [AP672, 674, 679, 683, 687, 5juv, AUMNH]; Joshua Tree Natl Park, Pleasant Valley, 33.9102, -115.9931^4^, 1066m, E Sleeper, S Jenkins 20.iii.1965 [AP395, 535, 2♂, AMNH], 8.i.1966 [AP396, 1♂, AMNH], 8.i.1965 [AP542, 1♂, AMNH], 26.iii.1966 [AP532, 1♀, AMNH], 7.i.1967 [AP536, 2♂, AMNH], 4.ii.1967 [AP541, 1♂, AMNH]; Quail Guzzler, Pleasant Valley, 33.9114, -116.0628^4^, 1016m, E Sleeper, S Jenkins 29.vii.1966 [AP568, 1♂, CAS]; Joshua Tree Natl Park, 1.3km S Squaw Tanks, 33.9185, -116.0785^4^, 1036m, E Sleeper, S Jenkins 20.iii.1965 [AP539, 1♂, AMNH]; 1.1km S Squaw Tanks, 33.9198, -116.0779^4^, 1036m, 18.xii.1965 [AP540, 1juv, AMNH]; Pinto Wells, 33.9408, -115.4161^4^, 305m, W Sakai 17.iii.1996 [AP599, 1♂, UCR]; Joshua Tree Natl Park, Pinyon Wells, 33.9642, -116.2472^5^, 1364m, E Sleeper 7.iv.1968 [AP530, 1♀, AMNH], E Sleeper, S Jenkins 4.iii.1967 [AP534, 1♂, AMNH]; **San Bernardino Co.:** Joshua Tree Natl Park, Lower Covington Flat, 34.0401, -116.3102^4^, 1433m, E Sleeper 16.ii.1962 [AP531, 1♂, AMNH], E Sleeper, S Jenkins 20.iii.1965 [AP533, 538, 2♂, AMNH]; Pisgah Crater, 34.7465, -116.3755^1^, 666m, Norris, Heath 11.ii.1961 [AP567, 620, 2♂, AMNH]; Joshua Tree Natl Park, ~2.7km S of bend in road, hwy 62, 34.0866, -115.4777^1^, 684m, J Bond, W Icenogle 17.i.1997 [AP712, 714, 2♀, 2juv, AUMNH]; NE Pinto Mountains, 34.0866, -115.4777^5^, 610m, W Icenogle, T Prentice 7.ii.1991 [AP394, 1♀, CAS].

#### Distribution and natural history.

*Aptostichus serrano* is a Mojave Desert endemic that has been collected in Riverside and San Bernardino Counties. Males apparently disperse during the later winter months, January–March. The DM indicates the species is probably more spread throughout the Mojave.

#### Conservation status.

The conservation status of *Aptostichus serrano* is considered to be secure because it is widespread and found in areas that are generally well protected (e.g., Joshua Tree National Park).

#### Species concept applied.

Morphological.

### Aptostichus aguacaliente sp. n.

‘The Windy Point Trapdoor Spider’

urn:lsid:zoobank.org:act:FD821ED4-8AF2-42C2-AE73-E65B2C014A24

http://species-id.net/wiki/Aptostichus_aguacaliente

Figures 220 –227 Map 27, 28

#### Types.

Male holotype and female paratype (AP393) from California, Riverside County, Windy Point, 8km NW of Palm Springs on HWY 111, 33.8964, -116.6251^1^, 488m, coll. W. Icenogle 15.i.1969 deposited in CAS.

#### Etymology.

The specific epithet is a noun in apposition taken from the Agua Caliente Band of the Cahuilla Native American Tribal group of Palm Springs, California.

#### Diagnosis.

**Figures 220–223.**
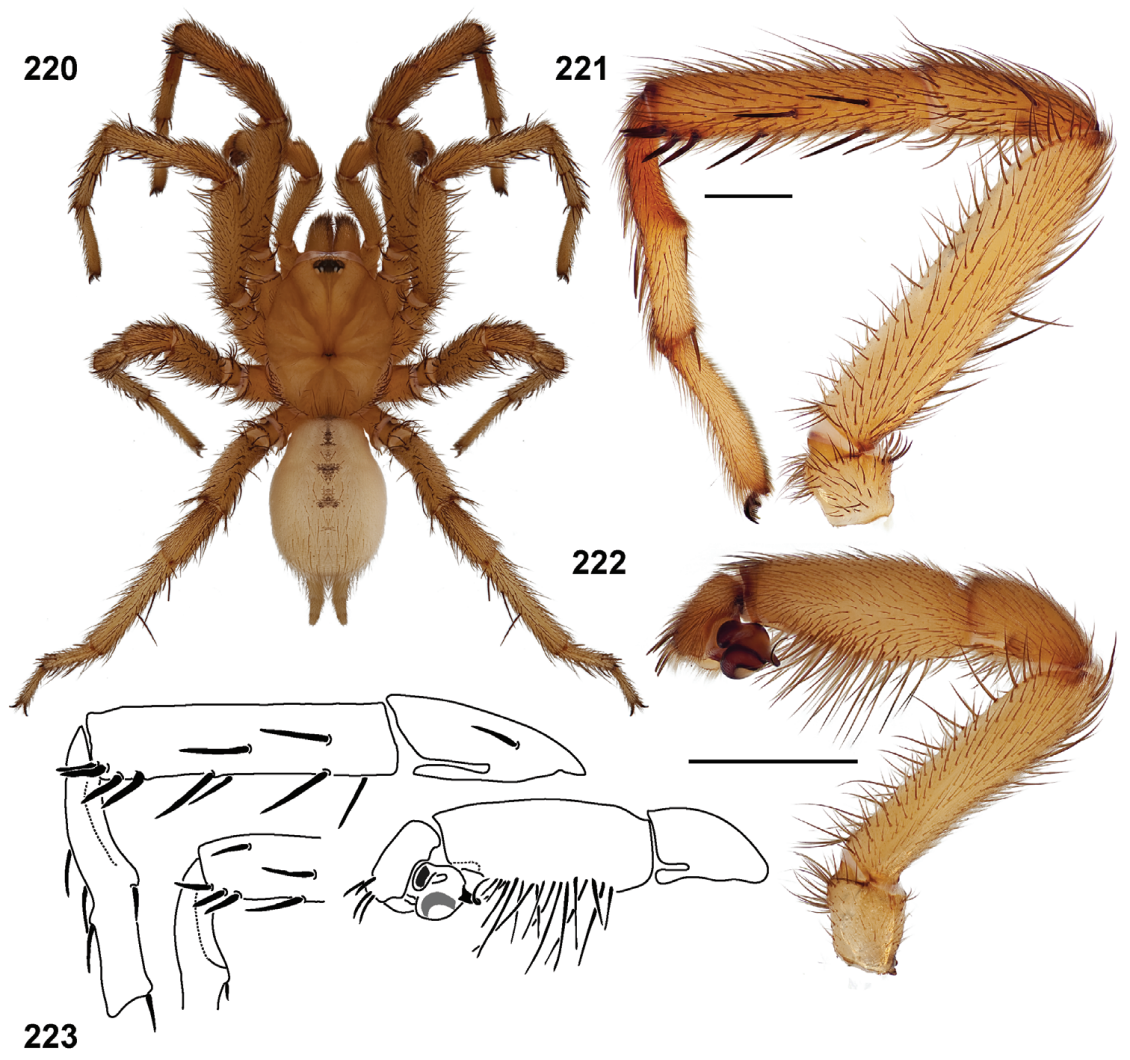
*Aptostichus aguacaliente* sp. n. male holotype (AP393). **220** habitus [805968] **221 **retrolateral aspect, leg I [805964]; scale bar = 1.0mm **222** retrolateral aspect, pedipalp; scale bar = 2.0mm [805970] **223** line drawings of retrolateral aspect leg pedipalp and I.

**Figures 224–227.**
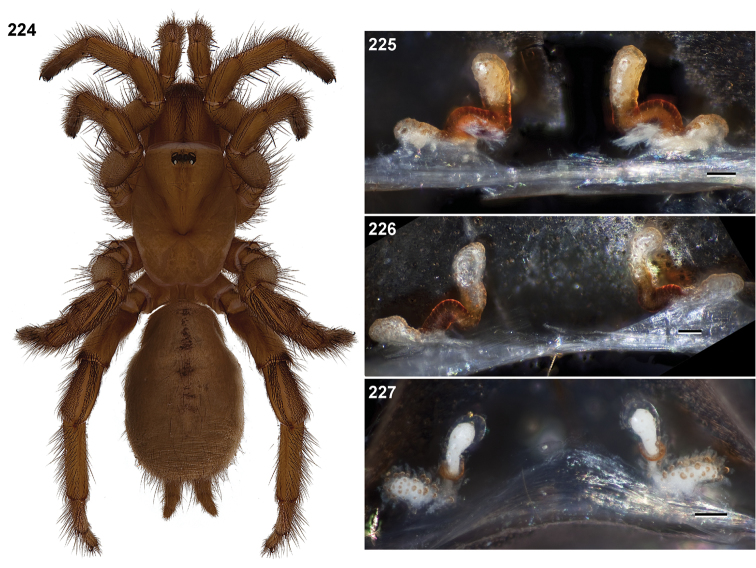
*Aptostichus aguacaliente* sp. n. female specimens. **224** paratype (AP393) habitus [805972] **225–227** cleared spermathecae of specimens from the type locality, Windy Point, Riverside Co. (AP393, 230, MY2508) [806704, 806614, 806617]; scale bars = 0.1mm.

Males (Fig. 220) of this species can be diagnosed on the basis of a unique conformation of the spination pattern of tibia I which consists of 3-5 long spines, sometimes overlapping, and by having a low tibia I apophysis that bears a spine (Figs 221, 223). Females can be distinguished by having a median spermathecal stalk that is sinuous and 8-9 times longer than wide (Figs 225–227). Males and females have features that are similar to *Aptostichus hesperus*: large sternal sigilla that are mid-ventrally positioned and a rastellum that consists of at least 6 enlarged spines with one offset prolaterally. However, *Aptostichus aguacaliente* sigilla are not contiguous and this species’ cephalothorax and abdominal coloration is very light (Figs 220, 224). *Aptostichus hesperus* coloration is much darker with a more distinctive abdominal banding pattern. *Aptostichus aguacaliente* males also tend to be smaller in size than *Aptostichus hesperus* males; however there is no discontinuous size difference between females of these two species.

#### Description of male holotype.

*Specimen preparation and condition*. Specimen collected live from under debris, preserved 70% EtOH. Coloration slightly faded. Pedipalp, leg I left side removed, stored in vial with specimen. *General coloration*. Carapace, chelicerae, legs yellowish brown 10YR 5/6. Abdomen uniform very pale brown 10YR 7/4, dark mid dorsal band markings (Fig. 220). *Cephalothorax*. Carapace 4.40 long, 3.63 wide, generally glabrous with sparse thin setae, stout long black bristles on posterior fringe; pars cephalica elevated. Foveal groove deep, straight. Eyes on low mound. AER straight, PER slightly recurved. PME, AME subequal diameter. Sternum moderately setose, STRl 2.32, STRw 2.10. Posterior sternal sigilla large, positioned centrally, not contiguous, anterior sigilla pairs small, oval, marginal. Chelicerae with distinct anterior tooth row comprising 7 teeth, posterior margin with single row of small denticles. Palpal endites with patch of small cuspules on proximal, inner margin, labium with 2 cuspules, LBw 0.77, LBl 0.48. Rastellum consists of 5 stout spines not on mound. *Abdomen*. Setose, heavy black setae intermingled with fine black setae. *Legs*. Leg I: 4.45, 3.20, 2.79, 1.90, 1.55; leg IV: 4.00, 2.00. Light tarsal scopulae on legs I, II. Tarsus I with single, slightly staggered row of 8 trichobothria. Leg I spination pattern illustrated in Figures 221, 223; TSp 4, TSr 2, TSrd 4; mid-ventral metatarsus mating apophysis bearing a single blunt spine. *Pedipalp*. Articles stout, lacking distinct spines (Fig. 222). PTw 0.87, PTl 1.88, Bl 1.02; palpal bulb long relative to carapace length; embolus slender, with slight distal curvature (Figs 222).

**Variation (10).** Cl 3.94-5.00, 4.57±0.12; Cw 3.19-4.31, 3.80±0.12; STRl 2.19-2.94, 2.52±0.09; STRw 1.80-2.56, 2.18±0.08; LBw 0.62-0.80, 0.73±0.02; LBl 0.38-0.60, 0.46±0.02; leg I: 4.19-5.38, 4.75±0.13; 2.81-3.75, 3.33±0.10; 2.56-3.38, 2.97±0.09; 1.71-2.16, 1.97±0.05; 1.14-1.74, 1.51±0.06; leg IV: 3.75-4.81, 4.28±0.12; 1.88-2.31, 2.13±0.05; PTl 1.77-2.34, 2.05±0.06; PTw 0.78-0.99, 0.91±0.02; Bl 0.96-1.13, 1.04±0.02; TSp 3-7, 4.50±0.37; TSr 2-7, 4.50±0.5; TSrd 3-5, 3.50±0.22.

#### Description of female paratype.

*Specimen preparation and condition*. Female collected live from burrow, prepared in same manner as male holotype. Genital plate removed, cleared in trypsin, stored in microvial with specimen. *General coloration*. Carapace, legs, chelicerae, dark yellowish brown 10YR 4/6. Abdomen uniform yellowish brown 10YR 5/4, markings similar to male (Fig. 224). *Cephalothorax*. Carapace 5.75 long, 4.88 wide, glabrous; generally smooth surface, pars cephalica moderately elevated. Fringe lacks setae. Foveal groove deep, procurved. Eye group slightly elevated on low mound. AER slightly procurved, PER slightly recurved. PME-AME subequal diameter. Sternum widest at coxae II/III, moderately setose, STRl 3.64, STRw 3.04. Three pairs of sternal sigilla, anterior pairs small, oval, marginal, posterior pair much larger, oval, mesially positioned but not contiguous. Chelicerae anterior tooth row comprising 8 teeth with posterior margin denticle patch comprising two short rows of teeth. Palpal endites with 55 cuspules concentrated at inner (promargin) posterior heel; labium with 3 cuspules, LBw 1.19, LBl 0.68. Rastellum consist of 6 very stout spines not positioned on mound, one spine offset prolaterally; fringe of short spines along distal promargin extending upward from rastellum. *Abdomen*. Moderately setose. PLS all 3 segments with spigots. Terminal segment 1/2 length of medial segment, 2 enlarged spigots visible at tip. PMS single segment, with spigots, short with rounded terminus. *Legs*. Anterior two pairs noticeably more slender than posterior pairs. Leg I 14.19 long. Tarsus I with single staggered row of 12 trichobothria. Legs I, II, with moderately heavy scopulae on tarsi, metatarsi; light scopulae on tarsi III, IV. PTLs 7, TBs 4. Distinct preening comb on retrolateral distal surface (at tarsus - metatarsus joint) of metatarsus III, IV. *Spermathecae*. 2 simple spermathecal bulbs with long, curved sclerotized median stalk, medial aspect runs parallel to genital lip before turning towards anterior (Figs 225-227). Basal extension lacks well-developed, distinct bulb.

**Variation (10).** Cl 5.00-6.69, 5.82±0.19; Cw 3.88-5.69, 4.87±0.19; STRl 3.00-4.25, 3.61±0.15; STRw 2.52-3.69, 3.10±0.12; LBw 0.98-1.35, 1.13±0.05; LBl 0.56-0.75, 0.69±0.02; Leg I: 12.19-17.13, 14.40±0.53; ANTd 6-9, 6.80±0.33; PTLs 7-11, 8.70±0.52; TBs 3-5, 4.10±0.28.

#### Material examined:

**United States: California: Imperial Co.:** 12.9km SE Niland, between Coachella Canal & Railroad, 33.192, -115.3933^4^, 26m, W Icenogle, T Prentice 22.ii.1996 [AP275, 1♀, 1juv, CAS]; **Riverside Co.:** Carrizo Creek, 6.4km S Palm Desert, 33.6264, -116.4215^3^, 1000m, D Bixler 27.ii.68 [AP216, 1juv, AMNH], W Icenogle 28.viii.1968 [AP213, 217, 218, 1♂, 2♀, 28juv, AMNH], 1.iv.1969 [AP219, 4juv, AMNH]; 16km W Chiriaco Summit on I-10, 33.6697, -115.9129^3^, 433m, R Vetter 1.iv.1997 [AP204, 205, 2♂, UCR]; 6.1km S Palm Desert, 33.6837, -116.4038^3^, 244m, S Telford 24.iii.1962 [AP208, 1♂, CAS]; 6.1km N Rancho Mirage, 33.8271, -116.4088^4^, 79m, W Icenogle 16.xi.1968 [AP066, 1♀, AMNH]; Off Gene Autry Rd, Whitewater Flood Plain Preserve, 33.87457, -116.5134^1^, 162m, J Bond 5.ii.2004 [MY2504, 1juv, AUMNH]; Palm Springs, Windy Point, 33.8908, -116.6302^1^, 325m, J Bond 24.i.1997 [AP720, 1♀, AUMNH]; Palm Springs, Windy Point, 33.8909, -116.6304^1^, 378m, J Bond 23.i.1997 [AP1205, 1207, 1220, 1233, 1234, 4juv, 1♀, AUMNH]; Windy Point, 33.8943, -116.6235^1^, 306m, J Bond, W Icenogle 28.i.2004 [MY2473, 1juv, AUMNH]; Santa Rosa Mountains, W side Windy Pt, ~1km SSW Tipton Rd & hwy 111 junction, 33.8956, -116.6481^1^, 344m, T Prentice 18.ii.2003 [AP1230, 1♂, UCR]; Windy Point, 8km NW Palm Springs on hwy 111, 33.8964, -116.6251^3^, 488m, W Icenogle, G Polis 7.ii.1976 [AP224-226, 3♀, CAS], [AP211, 223 1♂, 1♀, AMNH], G Ballmer 18.ii.1989 [AP600, 1♀, UCR], W Icenogle 15.i.1969 [AP210, 214, 228-231, 393, 10♀, 4♂, 27juv, CAS], [AP220, 1♀, AMNH], 13.iii.1968 [AP215, 1♀, AMNH], 27.i.1969, [AP206, 1♂, AMNH], 8.ii.1970 [AP212, 1♂, AMNH], 1.i.1972 [AP209, 4♂, AMNH], M Irwin 15.viii.1968 [AP574, 1juv, AMNH], S Johnson 18.ii.1976 [AP207, 1♂, AMNH], J Bond 16.i.1997 [AP1232, 1♂, AUMNH]; Windy Pt Area, Snow Creek Rd exit off hwy 111, 33.9109, -116.6757^1^, 342m, J Bond 5.ii.2004 [MY2502, 2508, 2509, 2515, 2519, 2♀, 3juv., AUMNH]; **San Bernardino Co.:** N Yucca Valley, NE of Water Canyon, S of Skyline Ranch Rd, 34.1451, -116.4515^1^, 1244m, USGS-BRD San Diego Sta. 1.iii.2002 [AP1244, 1♂, CAS]; Granite Mtns Preserve, 34.7844, -115.6579^1^, 1317m, R Vetter, J Bond 15.xii.97 [AP680, 1♂, AUMNH].

#### GenBank accession.

16S-tRNAval-12S: JX103235-JX103242

#### Distribution and natural history.

**Maps 27, 28.**
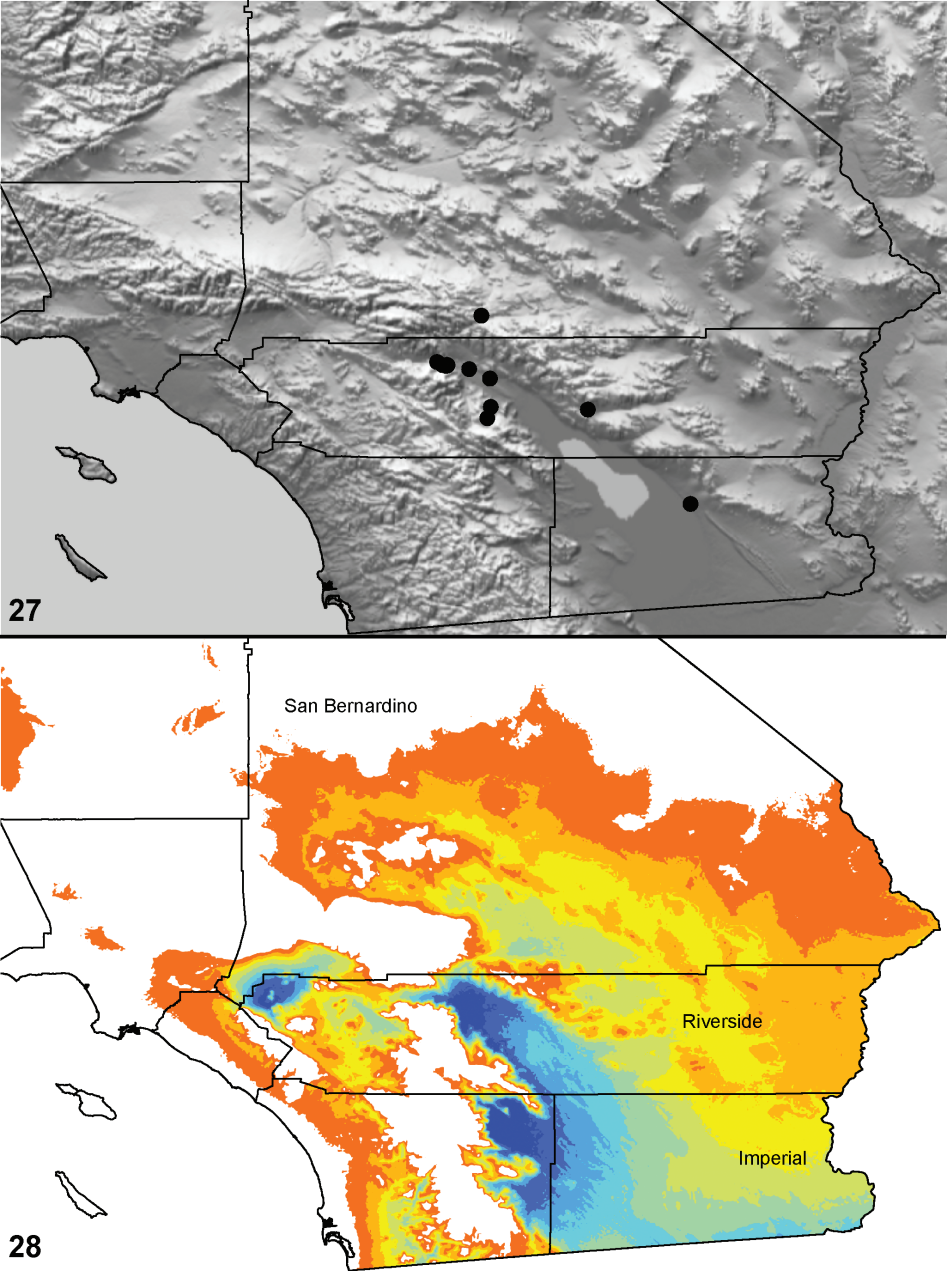
*Aptostichus aguacaliente* sp. n. **27** distribution of known specimens **28** predicted distribution; cooler colors–blue shades–represent areas of high probability of occurrence, warmer colors–yellow and orange shades–represent areas of low probability of occurrence.

*Aptostichus aguacaliente* is distributed primarily throughout Colorado Desert habitat in the low-lying ridges surrounding the Imperial Valley (Map 27). County records comprise San Bernardino, Riverside, and Imperial. The DM (Map 28) appears to considerably overpredict the occurrence of *Aptostichus aguacaliente* in areas to the west in Riverside County and into eastern San Diego County but otherwise corresponds to the known distribution. Based on the DM, it was likely that the species was more widely distributed throughout the Imperial Valley and areas to its north prior to the extensive agricultural development that has occurred in the region. Males appear to wander in the late winter, early spring, January–February. Female burrows can be detected during the winter months often after soaking rains when individuals extend their burrow leaving a small mound of soil at the burrow entrance.

#### Conservation status.

The conservation status of *Aptostichus aguacaliente* is likely classified as secure. However, the species is presumed to be extirpated from the type locality (Windy Point, Palm Springs) as a consequence of recent construction of a golf course and housing development.

#### Species concept applied.

Morphological/Phylogenetic.

### Aptostichus chemehuevi sp. n.

‘The Chemehuevi Desert Trapdoor Spider’

urn:lsid:zoobank.org:act:B78EEE09-995D-4E54-980F-44D52F4C626E

http://species-id.net/wiki/Aptostichus_chemehuevi

Figures 228–235 Map 25

#### Types.

Male holotype and paratype (AP398) from California, San Bernardino County, Pisgah Crater, 34.7465, -116.3755^1^, 666m, coll. Norris & Heath 17.ii.1967, deposited in AMNH.

#### Etymology.

The specific epithet is a noun in apposition taken from the Chemehuevi Band of the Southern Paiute Native American People.

#### Diagnosis.

**Figures 228–235.**
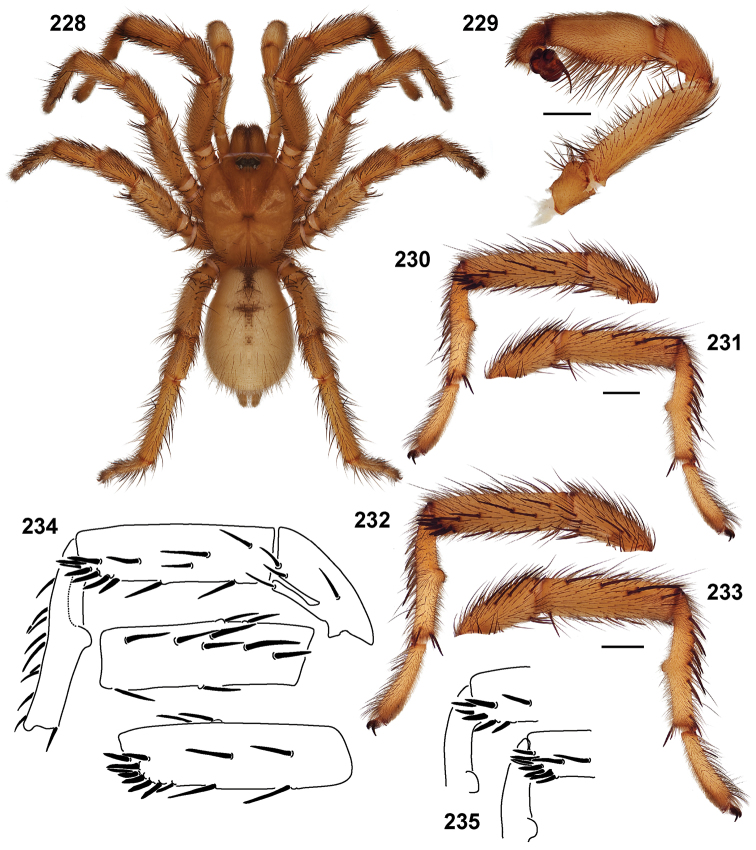
*Aptostichus chemehuevi* sp. n. male specimens; scale bars = 1.0mm. **228–231** holotype (AP398) **228** habitus **229** retrolateral aspect, pedipalp [805982] **230** retrolateral aspect, leg I [805978] **231 **prolateral aspect, leg I [805980] **232, 233** paratype (AP397) retrolateral and prolateral aspects, leg I [805984, 805988] **234, 235** line drawings of leg I tibia and metatarsus spination variation.

Males (Fig. 228) can be diagnosed from all known species of *Aptostichus* by having many spines on metatarsus I that form two distinct rows and by having a linear row of multiple spines on the prolateral surface of tibia I (Figs 230-234). Males of all other known species of *Aptostichus* have a single row of very few spines on metatarsus I and/or have very many spines dispersed across the prolateral surface of tibia I or have only three spines composing the row.

#### Description of male holotype.

*Specimen preparation and condition*. Specimen collected from pitfall, preserved in 70% EtOH. Coloration faded. Pedipalp, leg I left side removed, stored in vial with specimen. *General coloration*. Carapace, chelicerae, legs strong brown 7.5YR 4/6. Abdomen uniform pale brown 10YR 7/4, light markings dorsally (Fig. 228). *Cephalothorax*. Carapace 4.75 long, 4.15 wide, glabrous, stout black bristles along fringe; surface smooth, pars cephalica elevated. Fringe, posterior margin with black bristles. Foveal groove deep, moderately recurved. Eyes on low mound. AER slightly procurved, PER slightly recurved. PME, AME subequal diameter. Sternum moderately setose, STRl 2.91, STRw 2.33. Posterior sternal sigilla moderate in size, positioned toward center, not contiguous, anterior sigilla pairs small, oval, marginal. Chelicerae with distinct anterior tooth row comprising 6 teeth, posterior margin with single row of small denticles. Palpal endites with patch of small cuspules on proximal, inner margin, labium with 1 cuspule, LBw 0.80, LBl 0.44. Rastellum consists of 5 very stout spines arranged along anterior cheliceral margin, not on mound. *Abdomen*. Setose, heavy black setae intermingled with fine black setae. *Legs*. Leg I: 4.80, 3.40, 3.20, 1.98, 1.65; leg IV: 4.78, 2.17. Light tarsal scopulae on tarsus, metatarsus legs I, tarsus leg II. Tarsus I with single, slightly staggered row of 13 trichobothria. Leg I spination pattern illustrated in Figures 230, 231; TSp 6, TSr 4, TSrd 7. *Pedipalp*. Articles stout, lacking distinct spines (Fig. 229). PTw 1.02, PTl 2.17, Bl 1.04. Embolus slender, tapering sharply toward tip, without serrations (Fig. 229).

**Variation (10).** Cl 4.06-5.63, 4.77±0.13; Cw 3.38-4.75, 4.04±0.11; STRl 2.46-2.91, 2.69±0.05; STRw 1.77-2.49, 2.13±0.06; LBw 0.68-0.83, 0.75±0.02; LBl 0.44-0.56, 0.47±0.01; leg I: 4.19-5.44, 4.74±0.12; 2.81-3.81, 3.30±0.1; 2.79-3.50, 3.14±0.07; 1.65-2.10, 1.86±0.05; 1.53-1.86, 1.66±0.03; leg IV: 4.00-4.88, 4.53±0.09; 1.88-2.25, 2.12±0.04; PTl 1.89-2.37, 2.07±0.05; PTw 0.87-1.08, 0.96±0.02; Bl 0.98-1.20, 1.07±0.02; TSp 5-8, 6.20±0.29; TSr 4-8, 5.20±0.49; TSrd 6-9, 7.70±0.4.

#### Description of female.

Known only from male specimens.

#### Material examined.

**United States: California: San Bernardino Co.:** 29 Palms Joshua Tree National Park Headquarters, 34.1296, -116.0359^3^, 602m, J. Freilid 9.ii.1994 [AP601, 1♂, UCR]; Pisgah Crater, 34.7465, -116.3755^1^, 666m, Norris & Heath 11.xi.1961 [AP566, 1♂, AMNH], 17.ii.1962 [AP344, 346, 398, 349, 4♂, AMNH], 6.i.1963 [AP345, 397, 2♂, AMNH], 6.i.1967 [AP565, 1♂, AMNH], 1.ii.1961 [AP342, 347, 2♂, AMNH], 25.ii.1961 [AP343, 1♂, AMNH], 11.ii.1961 [AP348, 1♂, AMNH]; Kelso Dunes, 34.9108, -115.7303^3^, 770m, D. Weisman 20.xii.1986 [AP340, 1♂, AMNH], V. & B. Roth 4.i.1981 [AP341, 2juv., CAS].

#### Distribution and natural history.

*Aptostichus chemehuevi* is known from only three Mojave Desert localities in San Bernardino County (Map 25); despite extensive collecting efforts at the type locality females have never been collected thus little is known of this species natural history. Males appear to disperse during the winter months, November-February. This species is considered to be syntopic with *Aptostichus elisabethae*.

#### Conservation status.

Although little information is available, the conservation status of *Aptostichus chemehuevi* is likely imperiled (also see discussion of *Aptostichus elisabethae*) given its paucity of specimens in collections and distribution restricted to three locations.

#### Species concept applied.

Morphological.

### Aptostichus sarlacc sp. n.

‘The Sarlacc Trapdoor Spider’

urn:lsid:zoobank.org:act:2FCA4B5E-04D4-4D23-A829-617B0DCCFDA5

http://species-id.net/wiki/Aptostichus_sarlacc

Figures 236–242 Map 29

#### Types.

Male holotype (AP417) from California, San Bernardino County, 14.5km N, 16km E of Ridgecrest sand dunes, 35.7553, -117.5006^4^, 960m, coll. D. Giuliani 15.ii.1981-12.iv.1981; male paratype (AP416) from California, Kern County, 11.3km, N 9.6km W of Inyokern, 35.7362, -117.9849^4^, 2193m, coll. D. Giuliani 4.iv.1986; male paratype from California, Inyo County, Owens Valley, 9.6km S-SW of Independence, 36.7414, -118.2623^5^, 1961m, coll. D. Giuliani 1.xi.1986-12.vi.1987. Deposited in CAS.

#### Etymology.

The specific epithet is a noun in apposition taken from the fictional creature in George Lucas’ science fiction saga, *Star Wars: Return of the Jedi*.

#### Diagnosis.

**Figures 236–242.**
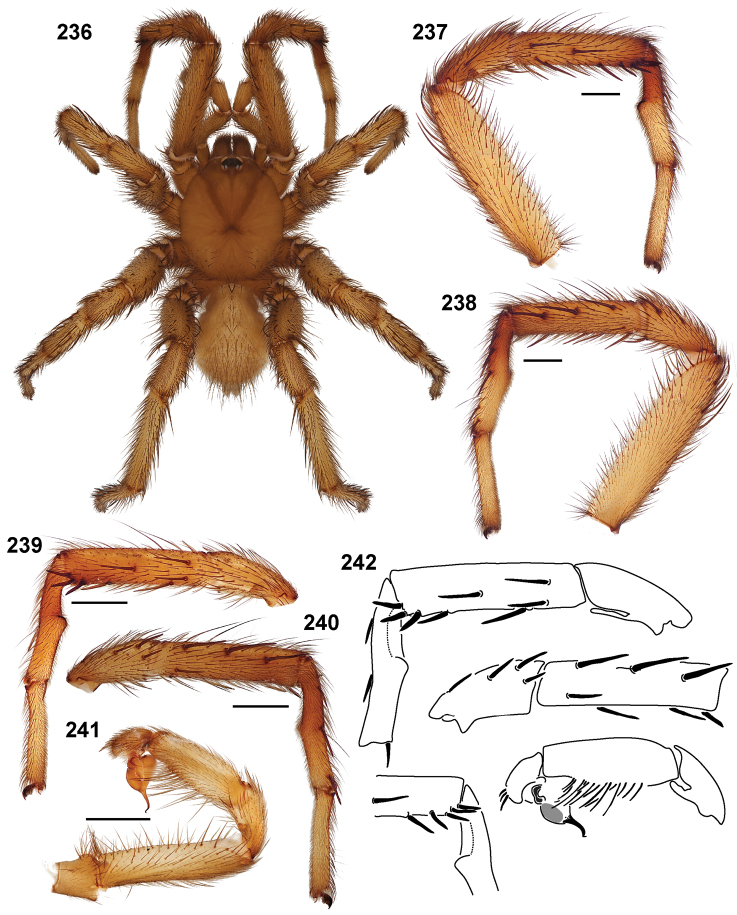
*Aptostichus sarlacc* sp. n.; scale bars = 1.0mm. **236–238** male paratype (AP416) from Kern Co. **236** habitus [806006] **237** retrolateral aspect, right leg I [806000] **238** prolateral aspect, right leg I [806004] **239–242** male holotype (AP417) **239** retrolateral aspect, leg I [805992] **240** prolateral aspect, leg I [805996] **241** retrolateral aspect, pedipalp [805998] **242** line drawings (in descending order) of holotype leg I metatarsus and tibia spination pattern (retro and prolateral views), pedipalp (retrolateral view), lower inset, line drawing of paratype TSrd spination pattern.

Males can be distinguished from other known closely related species of *Aptostichus* (e.g., *Aptostichus cahuilla*, *Aptostichus aguacaliente*) by having a long curved metatarsus IV relative to femur IV length and by having an abdomen devoid of any dorsal markings (Fig. 236). This species can be distinguished from geographical proximate members of the *Simus* species group, with similarly long fourth tarsi and by having a longer, more slender palpal tibia (Figs 241, 242). The distribution of *Aptostichus sarlacc* is distributed considerably further to the north of the aforementioned *Hesperus* species group taxa.

#### Description of male holotype.

*Specimen preparation and condition*. Specimen collected dead from pitfall trap, preserved 70% EtOH. Coloration likely faded. Pedipalp, leg I left side removed, stored in vial with specimen. *General coloration*. Carapace, chelicerae, legs strong brown 7.5YR 4/6. Abdomen uniform very pale brown, lacking distinct dorsal markings (e.g., paratype coloration pattern, Fig. 236). *Cephalothorax*. Carapace 3.41 long, 2.95 wide, glabrous with only sparse thin black setae, stout black bristles along fringe; surface smooth, pars cephalica elevated. Fringe, posterior margin with black bristles. Foveal groove deep, moderately procurved. Eyes on low mound. AER slightly procurved, PER slightly recurved. PME, AME subequal diameter. Sternum moderately setose, STRl 1.90, STRw 1.62. Posterior sternal sigilla small, positioned towards lateral margin, anterior sigilla pairs small, oval, marginal. Chelicerae with distinct anterior tooth row comprising 5 teeth, posterior margin with patch of very small denticles. Palpal endites with patch of small cuspules on proximal, inner margin, labium with 3 small cuspules, LBw 0.60, LBl 0.29. Rastellum consists of 6 stout spines, 2 offset prolaterally. *Abdomen*. Setose, heavy black setae intermingled with fine black setae. *Legs*. Leg I: 3.60, 2.40, 2.14, 1.67, 1.12; leg IV: 3.44, 2.16. Light tarsal scopulae on tarsi legs I, II. Tarsus I with single, slightly staggered row of 13 trichobothria. Leg I spination pattern illustrated in Figures 239, 240; TSp 4, TSr 4, TSrd 3. *Pedipalp*. Articles slender, lacking distinct spines (Figs 241, 242). PTw 0.48, PTl 1.40, Bl 0.70. Embolus slender, curved at midpoint, with slight curve distally, lacking serrations (Figs 241, 242).

**Variation (2).** Cl 3.41-4.85, Cw 2.95-4.28, STRl 1.9-2.45, STRw 1.62-2.19, LBw 0.60-0.85, LBl 0.29-0.46, leg I: 3.60-5.00, 2.40-3.19, 2.14-2.97, 1.67-2.25, 1.12-1.53; leg IV: 3.44-4.75, 2.16-2.88; PTl 1.40-1.98, PTw 0.48-0.63, Bl 0.70-1.01, TSp 4-4, TSr 3-4, TSrd 3-3.

#### Description of female.

Known only from male specimens.

#### Material examined.

Known only from the type material.

#### Distribution and natural history.

**Map 29.**
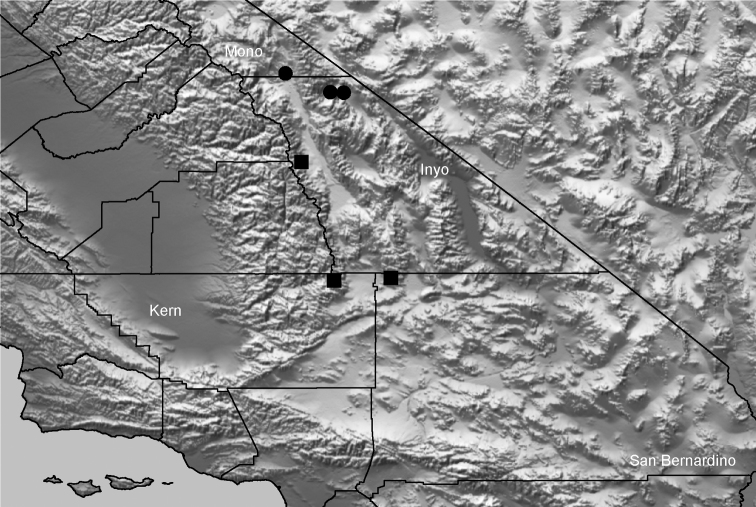
Distribution of *Aptostichus sarlacc* sp. n. (filled squares) and *Aptostichus derhamgiulianii* sp. n. (filled circles).

Little is known about this species; it is very rare in collections and is only known from two specimens collected in the Mojave Desert in Inyo, Kern and San Bernardino Counties (Map 29).

#### Conservation status.

This species is rare in collections and is known from only two localities; its status is considered imperiled.

#### Species concept applied.

Morphological.

### Aptostichus derhamgiulianii sp. n.

‘Giuliani’s Desert Trapdoor Spider’

urn:lsid:zoobank.org:act:826298C4-9579-4C4D-9B3F-8A755AFE01D9

http://species-id.net/wiki/Aptostichus_derhamgiulianii

**Figures 243–248.**
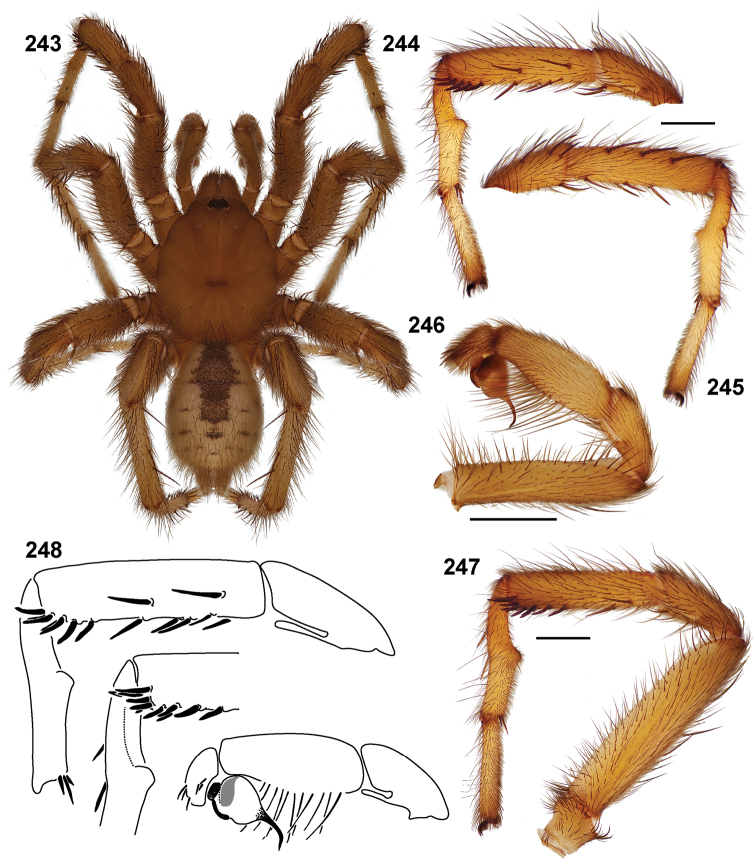
*Aptostichus derhamgiulianii* sp. n.; scale bars = 1.0mm. **243–246** male holotype (AP 415) Inyo County **243** habitus [806016] **244** retrolateral aspect, leg I [806010] **245** prolateral aspect, leg I [806014] **246** retrolateral aspect, pedipalp [806018] **247** male paratype (AP424) from Mono County, retrolateral aspect, leg I [806020] **248** line drawings, spination pattern, leg I tibia and metatarsus of holotype and paratype; retrolateral view, holotype pedipalp.

Figures 243–248 Map 29

#### Types.

Male holotype (AP415) from California, Inyo County, Deep Springs Valley sand dunes, 37.3333, -118.0167^4^, 1536m, coll. D. Giuliani 19.xii.1973; male paratype (AP424) from California, Mono County, 14.5km, N of Bishop, 37.4939, -118.3977^4^, 1300m, coll. D. Giuliani 29.vii.1980. Deposited in CAS.

#### Etymology.

The specific epithet is a patronym in honor of California naturalist Derham Giuliani who collected a number of very rare *Aptostichus* specimens.

#### Diagnosis.

Males of this species can be distinguished by having the following unique combination of features: a wide sternum, a rastellum with a single offset retrolateral spine, PME’s smaller in diameter than AME’s, multiple TSrd spines that overlap (Figs 244, 247, 248), and spines on the retrolateral surface of the cymbium (Figs 246, 248). The overlapping TSrd spination pattern is similar to that of *Aptostichus cahuilla*, however, the spines on tibia I appear to be distributed more along the distal ventral surface in *Aptostichus derhamgiulianii* than *Aptostichus cahuilla*.

#### Description of male holotype.

*Specimen preparation and condition*. Specimen presumed to have been collected from a pitfall trap, preserved in 70% EtOH. Coloration likely faded. Pedipalp, leg I left side removed, stored in vial with specimen. *General coloration*. Carapace, chelicerae, legs strong brown 7.5YR 4/6. Abdomen uniform light yellowish brown 10YR 6/4; dusky dorsal chevron marking (Fig. 243). *Cephalothorax*. Carapace 4.05 long, 3.41 wide, glabrous posteriorly, fine white setae anteriorly, stout black bristles along fringe posterior half; surface smooth, pars cephalica elevated. Fringe, posterior margin with black bristles. Foveal groove deep, slightly procurved. Eyes on low mound. AER slightly procurved, PER slightly recurved. PME’s much smaller in diameter than AME’s. Sternum moderately setose, STRl 2.31, STRw 2.04. Posterior sternal sigilla very small, positioned laterally; anterior sigilla pairs small, oval, marginal. Chelicerae with distinct anterior tooth row comprising 5 teeth, posterior margin with single row of small denticles. Palpal endites with patch of small cuspules on proximal, inner margin, labium with 3 cuspules, LBw 0.71, LBl 0.35. Rastellum consists of 7 stout spines, 2 spines offset prolaterally. *Legs*. Leg I: 4.16, 2.92, 2.60, 1.83, 1.38; leg IV: 4.00, 2.25. Light scopulae on tarsus, metatarsus I, tarsus II. Tarsus I with single, slightly staggered row of 11 trichobothria. Leg I spination pattern illustrated in Figures 244, 245, 247, 248; TSp 3, TSr 5, TSrd 7. *Pedipalp*. Articles stout, lacking distinct spines (Figs 246); spines on retrolateral surface of cymbium. PTw 0.59, PTl 1.62, Bl 0.84. Embolus slender, tapering sharply toward tip, with serrations (Figs 246, 248).

**Variation (3).** Cl 4.05-4.50, 4.31±0.13; Cw 3.41-3.75, 3.62±0.10; STRl 2.31-2.34, 2.32±0.01; STRw 2.04-2.13, 2.07±0.03; LBw 0.63-0.78, 0.71±0.04; LBl 0.35-0.35, 0.35±0; leg I: 4.16-4.56, 4.39±0.12; 2.92-3.25, 3.04±0.11; 2.58-2.85, 2.68±0.09; 1.83-1.95, 1.89±0.03; 1.38-1.56, 1.48±0.05; leg IV: 4.00-4.25, 4.17±0.08; 2.25-2.44, 2.33±0.06; PTl 1.62-1.65, 1.64±0.01; PTw 0.56-0.62, 0.59±0.02; Bl 0.84-0.93, 0.87±0.03; TSp 2-5, 3.33±0.88; TSr 2-6, 4.33±1.2; TSrd 6-11, 8.00±1.53.

#### Description of female.

Known only from male specimens.

#### Material examined.

**United States: California: Inyo Co.:** Eureka Valley, 32.2km NW sand dunes, 37.3297, -117.9029^3^, 1250m, D Giuliani 1.xii.1981-1.iv.1982 [AP571, 1♂, CAS]; Deep Springs Valley sand dunes, 37.3333, -118.0167^4^, 1536m, D Giuliani 19.xii.1973 [AP415, 1♂, CAS]; **Mono Co.:** 14.5km, N of Bishop, 37.4939, -118.3977^4^, 1300m, D Giuliani 29.vii.1980 [AP424, 1♂, CAS].

#### Distribution and natural history.

*Aptostichus derhamgiulianii* is known from only a few male specimens, two of which were collected from pitfall traps during December (the other July), from sand dune habitats of the eastern foothills and ranges of the Mojave Desert (Map 29) in Inyo and Mono Counties.

#### Conservation status.

This species is rare in collections and is known from only a few localities; its status is considered imperiled.

#### Species concept applied.

Morphological.

### Aptostichus mikeradtkei sp. n.

‘The Radtke Chula Vista Trapdoor Spider’

urn:lsid:zoobank.org:act:82756653-EA84-429B-A811-8A85AEE5775B

http://species-id.net/wiki/Aptostichus_mikeradtkei

Figures 249–256 Map 30

#### Types.

Male holotype (AP1085) from California, San Diego County, Chula Vista, Terra Nova, north of Rice Canyon, 32.64195, -117.03608^1^, 106m, coll. USGS-BRD San Diego Field Station 1.ii.2000; male paratype (AP1089) from same general vicinity of the type locality and by same collector, 32.6409, -117.03632^1^, 88m, coll. 1.ii.2000; female paratype (AP1148) from Escondido Wild Animal Park, 33.09157, -116.98298^1^, coll. 1.vi. 1998. Types deposited in CAS.

#### Etymology.

The specific epithet is a patronym in honor of Mike Radtke in recognition of his service to the National Institute of Health and his family’s support of the College of Sciences and Mathematics at Auburn University.

#### Diagnosis.

Males and females of this species can be distinguished from all other known species of *Aptostichus* by their unusually round, raised sternum, and striking sigilla (Fig. 256).

#### Description of male holotype.

**Figures 249–256.**
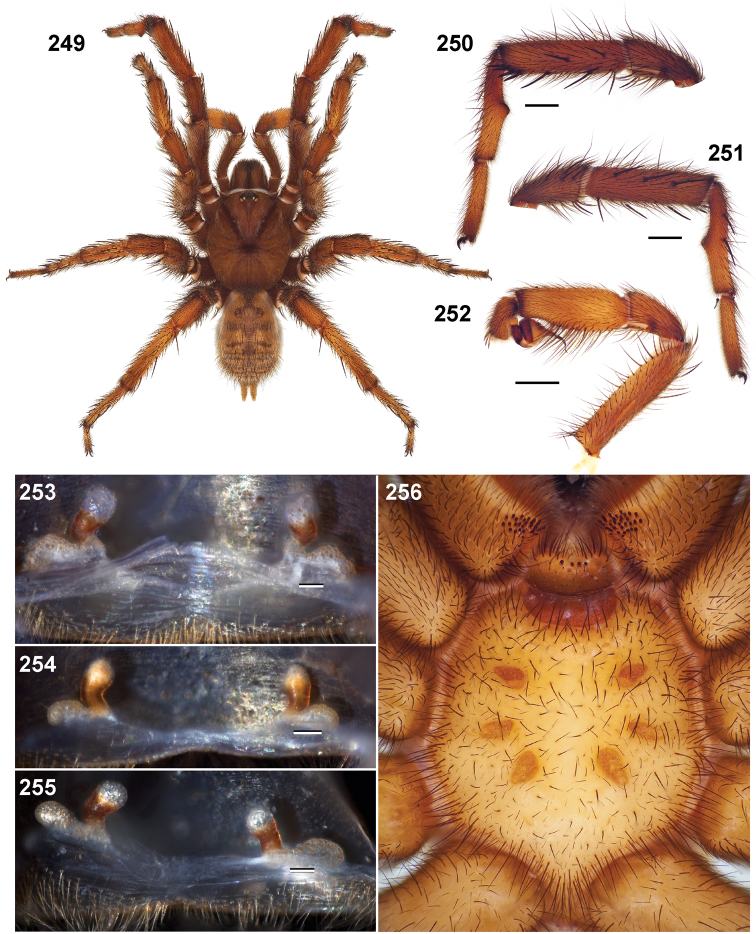
*Aptostichus mikeradtkei* sp. n. **249–252** male holotype (AP1085), from San Diego Co.; scale bars = 1.0mm. **249** habitus [806034] **250** retrolateral aspect, leg I [806032] **251** prolateral aspect, leg I [806028] **252** retrolateral aspect, pedipalp [806036] **253–255** cleared spermathecae; scale bars = 0.1mm **253** female paratype (AP1148) [806705] **254, 255** additional specimens from vicinity of type locality (AP1155, 1247) [806620, 806623] **256** sternum of paratype female (AP1148) [806038].

*Specimen preparation and condition*. Specimen collected from pitfall trap, preserved in 70% EtOH. Pedipalp, leg I left side removed, stored in vial with specimen. *General coloration*. Carapace, chelicerae, legs very dark brown 7.5YR 2.5/3. Abdomen brown 7.5YR 4/4, prominent mottled striping dorsally (Fig. 249). *Cephalothorax*. Carapace 5.60 long, 4.85 wide, hirsute covered in light white setae, stout black bristles along fringe; surface smooth, pars cephalica elevated. Fringe, posterior margin with black bristles. Foveal groove deep, straight. Eyes on low mound. AER slightly procurved, PER slightly recurved. PME, AME subequal diameter. Sternum moderately setose, rounded, raised towards center (Fig. 256), STRl 3.41, STRw 2.91. Posterior sternal sigilla large, heavily sclerotized, forming a depression, positioned centrally, but not contiguous; anterior sigilla medium in size, similar to posterior pair small, positioned towards margin. Chelicerae with distinct anterior tooth row comprising 11 teeth, posterior margin with single row of small denticles. Palpal endites with patch of small cuspules on proximal, inner margin, labium 5 cuspules, LBw 1.02, LBl 0.37. Rastellum consists of 6 stout spines not on prominent mound, one spine offset prolaterally. *Abdomen*. Setose, heavy black setae intermingled with fine black setae. *Legs*. Leg I: 5.35, 3.75, 3.44, 2.26, 1.86; leg IV: 5.25, 2.80. Moderate-light tarsal, metatarsal scopulae on leg I; tarsal scopulae leg II. Tarsus I with single, slightly staggered row of 11 trichobothria. Leg I spination pattern illustrated in Figures 250, 251; TSp 3, TSr 3, TSrd 4. *Pedipalp*. Articles slender, lacking distinct spines (Fig. 252). PTw 1.02, PTl 2.57, Bl 1.33. Embolus slender, curved slightly at midpoint, tip, lacks serrations (Fig. 252).

**Variation (5).** Cl 4.56-5.90, 5.42±0.23; Cw 3.84-5.10, 4.72±0.23; STRl 2.91-3.41, 3.20±0.09; STRw 2.38-3.12, 2.85±0.13; LBw 0.85-1.02, 0.96±0.03; LBl 0.37-0.60, 0.45±0.04; leg I: 4.35-5.35, 5.07±0.18; 3.20-3.80, 3.64±0.11; 2.88-3.56, 3.32±0.12; 2.03-2.33, 2.23±0.05; 1.63-2.0, 1.83±0.06; leg IV: 4.36-5.25, 5.03±0.17; 2.42-2.80, 2.68±0.07; PTl 2.13-2.57, 2.46±0.08; PTw 0.87-1.11, 1.01±0.04; Bl 1.07-1.33, 1.23±0.05; TSp 3-8, 4.2±0.97; TSr 1-6, 3±0.84; TSrd 3-5, 4.2±0.37.

#### Description of female paratype.

*Specimen preparation and condition*. Female collected from pitfall trap, prepared in same manner as male holotype. Genital plate removed, stored in microvial with specimen. *General coloration*. Carapace, legs, chelicerae, dark reddish brown 5YR 3/4. Abdomen brown dorsally 7.5YR 4/3, distinct mottled chevron marking pattern. *Cephalothorax*. Carapace 5.92 long, 4.95 wide, glabrous; generally smooth surface, pars cephalica moderately elevated. Fringe lacks setae. Foveal groove deep, straight. Eye group slightly elevated on low mound. AER slightly procurved, PER slightly recurved. PME, AME subequal diameter. Sternum widest at coxae II/III, moderately setose, STRl 3.80, STRw 3.48. Three pairs of sternal sigilla anterior pairs moderate in size, oval, shifted away from margin, posterior pair larger, oval, mesially positioned. Chelicerae anterior tooth row comprising 5 teeth with posterior margin denticle patch. Palpal endites with 37 cuspules concentrated at the inner (promargin) posterior heel; labium with 5 cuspules, LBw 1.19, LBl 0.51. Rastellum consists of 6 very stout spines not positioned on mound, one spine offset prolaterally; fringe of short spines along distal promargin extending upward from rastellum. *Abdomen*. Moderately setose. PLS all 3 segments with spigots. Terminal segment 1/2 length of medial segment, 2 enlarged spigots visible at tip. PMS single segment, with spigots, short with rounded terminus. *Legs*. Anterior two pairs noticeably more slender than posterior pairs. Leg I 13.76 long. Tarsus I with single staggered row of 11 trichobothria. Legs I with moderately heavy scopulae on tarsus, metatarsus, distal aspect tibia; leg II scopulae on tarsus, metatarsus; legs III, IV with light scopulae on distal aspect tarsus. PTLs 11, TBs 3. Distinct preening comb on retrolateral distal surface, tarsus-metatarsus joint, of metatarsus III, IV. *Spermathecae*. 2 simple spermathecal bulbs with an elongate curved stalk; basal extension small (Figs 253-255).

**Variation (3).** Cl 5.13-5.95, 5.58±0.21; Cw 4.38-5.15, 4.73±0.19; STRl 3.27-3.92, 3.64±0.14; STRw 2.91-3.48, 3.27±0.13; LBw 0.90-1.19, 1.1±0.07; LBl 0.41-0.60, 0.53±0.05; Leg I: 11.65-13.83, 12.73±0.62; ANTd 5-6, 5.50±0.29; PTLs 11-12, 11.25±0.25; TBs 3-3, 3.00±0.00.

#### Material examined.

**United States: California: San Diego Co.:** Little Cedar Canyon, SE of Otay Mountain, 32.6164, -116.8604^1^, 443m, USGS-BRD San Diego Sta. 1.ii.1999 [AP913, 1♂, CAS]; Little Cedar Canyon, SE Otay Mountain, 32.6252, -116.8634^1^, 373m, USGS-BRD San Diego Sta. [AP915, 2♂, CAS]; vic Chula Vista, 32.6406, -117.1041^3^, 5m, R Fisher 21.ii.2000 [AP1222, 1251, 1283, 3♂, CAS]; Chula Vista, Terra Nova, N Rice Canyon, 32.6409, -117.0363^1^, 88m, USGS-BRD San Diego Field Sta. 1.ii.2000 [AP1089, 1♂, CAS]; Chula Vista, Terra Nova, N Rice Canyon, 32.6419, -117.036^1^, 106m, USGS-BRD San Diego Field Sta. 1.i.2001 [AP1083, 1♂, CAS], 1.ii.2000 [AP1085, 1♂, CAS]; Chula Vista, Terra Nova, N Rice Canyon, 32.6432, -117.03778^1^, 105m, USGS-BRD San Diego Field Sta. 1.ii.2000 [AP1090, 1♂, CAS]; Chula Vista, E Long Canyon, 32.65, -116.9908^1^, 91m, USGS-BRD San Diego Field Sta. 1.i.2001 [AP1071, 1♂, CAS]; Chula Vista, E Long Canyon, 32.6501, -116.9921^1^, 90m, USGS-BRD San Diego Sta. 1.ii.2000 [AP1072, 1♂, CAS], 1.i.2001 [AP1075, 1♂, CAS], 1.xi.1998 [AP1077, 1♂, CAS]; Chula Vista, E Long Canyon, 32.65292, -116.9972^1^, 87m, USGS-BRD San Diego Field Station 1.x.2000 [AP1080, 1♂, CAS]; Chula Vista, Proctor Valley, SE Horseshoe Bend, S Proctor Valley Rd, 32.663, -116.9806^1^, 152m, USGS-BRD San Diego Sta. 1.ii.2000 [AP1065, 1♂, CAS]; Chula Vista, Proctor Valley, SE Horseshoe Bend, S Proctor Valley Rd, 32.6637, -116.9798^1^, 151m, USGS-BRD San Diego Sta. 1.ii.2000 [AP1067, 1♂, CAS], 1.xi.1998 [AP1225, 1♂, CAS]; Pt Loma, Cabrillo Natl Monument, 32.7101, -117.2523^1^, 79m, J Satler 13.vii.2009 [AP1247, 1♀, AUMNH]; San Diego, 32.733, -117.102^6^, 85m, B Kaston 1.ii.1971 [AP122, 1♂, CAS], L Passmore 31.xii.1938 [AP489, 1♂, AMNH]; Alpine, 32.835, -116.766^5^, 560m, P Lancaster 28.ii.1970 [AP430, 1♂, AMNH], P Sullivan, J Hark 22.iv.1973 [AP431, 1♀, CAS]; Flinn Springs, Rios Canyon, 32.8408, -116.8719^1^, 260m, USGS-BRD San Diego Sta. 1.i.2001 [AP1082, 1♂, CAS]; Carmel Mountain, 32.9283, -117.2227^1^, 118m, USGS-BRD San Diego Field Sta. 1.xii.2002 [AP1195, 1♂, CAS]; Del Mar Mesa, 32.9423, -117.168^1^, 102m, USGS-BRD San Diego Field Sta. 1.xii.2002 [AP1063, 1♂, CAS]; Escondido Wild Animal Park, 33.0893, -116.9888^1^, 177m, USGS-BRD San Diego Field Sta. 1.vi.2000 [AP1147, 1♂, CAS]; Escondido Wild Animal Park, 33.0898, -116.9866^1^, 201m, USGS-BRD San Diego Field Sta. 1.i.2000 [AP1145, 1♂, CAS]; Escondido, San Diego Wild Animal Park, 33.0912, -116.9829^1^, 173m, USGS-BRD San Diego Sta. 1.ii.2001 [AP1015, 2♂, CAS]; Escondido Wild Animal Park, 33.0915, -116.9861^1^, 194m, USGS-BRD San Diego Field Sta. 1.vi.1998 [AP1148, 1♀, CAS], 1.ii.2001 [AP1149, 1♂, CAS]; Escondido Wild Animal park, 33.0951, -116.9832^1^, 247m, USGS-BRD San Diego Sta. 1.ii.2001 [AP1152, 1♂, CAS]; Escondido Wild Animal park, 33.0967, -116.9812^1^, 236m, USGS-BRD San Diego Sta. 1.ii.2001 [AP1154, 1♂, CAS]; Escondido Wild Animal Park, 33.0976, -116.9782^1^, 254m, USGS-BRD San Diego Field Sta. 1.ii.2001 [AP1146, 2♂, CAS], 1.xi.98 [AP1155, 1♀, CAS]; Escondido Wild Animal Park, 33.1008, -116.9729^1^, 255m, USGS-BRD San Diego Sta. 1.ii.2001 [AP1158, 1♂, CAS].

#### Distribution and natural history.

**Maps 30, 31.**
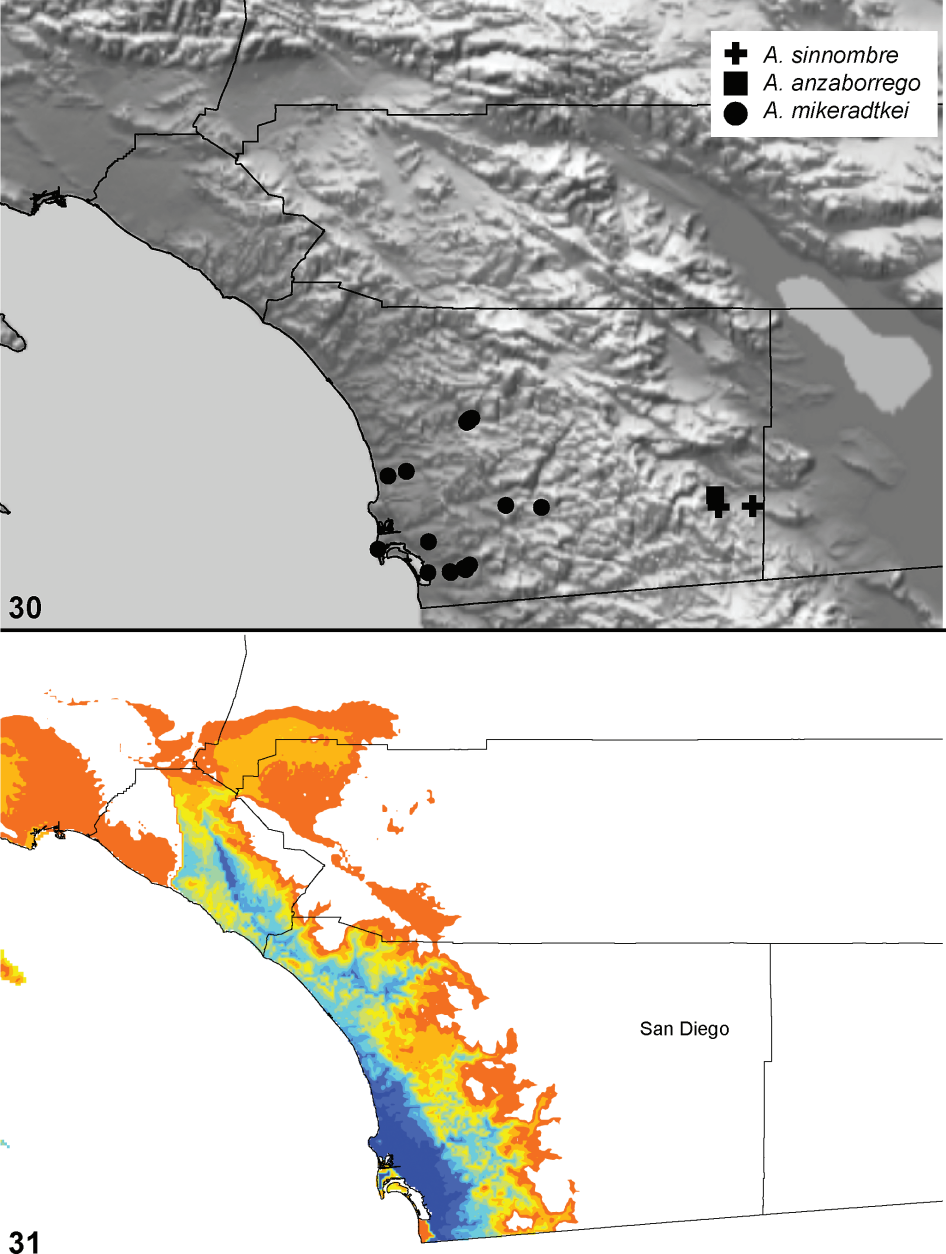
*Aptostichus sinnombre* sp. n., *Aptostichus anzaborrego* sp. n., *Aptostichus mikeradtkei* sp. n. **30** known distributions **31** predicted distribution of *Aptostichus mikeradtkei*; cooler colors–blue shades–represent areas of high probability of occurrence, warmer colors–yellow and orange shades–represent areas of low probability of occurrence.

*Aptostichus mikeradtkei* is distributed primarily along the coast in San Diego County (Map 30); the primary habitat type where it has been collected is characterized as coastal chaparral and shrub. Males appear to disperse early through mid-late winter during November-February. The DM for the species indicates that its distribution likely extends further up the coast and into Orange County.

#### Conservation status.

Based on its abundance and relatively large number of populations sampled, the conservation status of *Aptostichus mikeradtkei* is likely secure.

#### Species concept applied.

Morphological.

### Aptostichus edwardabbeyi sp. n.

‘The Desert Solitare Trapdoor Spider’

urn:lsid:zoobank.org:act:45A106AE-A41F-4872-BFEB-8BF551A0E070

http://species-id.net/wiki/Aptostichus_edwardabbeyi

**Figures 257–261.**
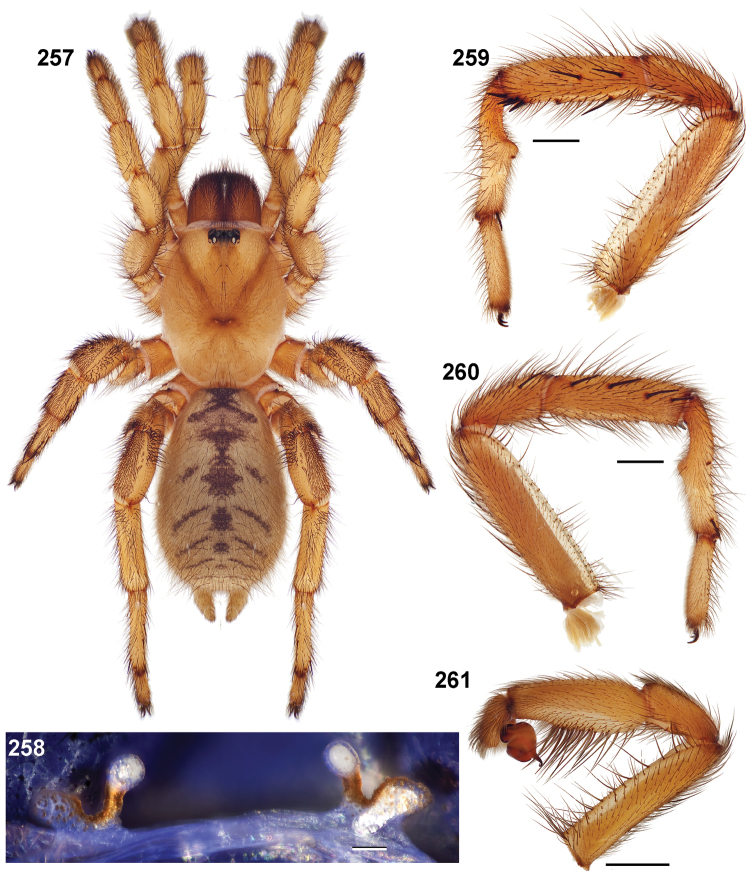
*Aptostichus edwardabbeyi* sp. n. **257, 258** habitus and cleared spermathecae of female paratype from Santa Cruz Co., Arizona (MY2279); scale bar = 0.1mm [806086] **259–261** male holotype (AP402) [806706]; scale bar = 1.0mm **259** retrolateral aspect, leg I [806078] **260** prolateral aspect, leg I [806076] **261** retrolateral aspect, pedipalp [806072].

Figures 257–261 Map 1

#### Types.

Male holotype (AP402) from Arizona, Cochise County, Chiricahua National Monument, 32.0044, -109.3561^4^, 1650m, coll. V. LeMay 14.xi.1968; male paratype (AP403) from Arizona, Pima County, Tucson, 32.224, -110.0955^7^, 700m, coll. 8.ii.1947; female paratype (MY2279) from Arizona, Santa Cruz County, 31.725, -110.879^3^, coll. 6.v.1997. Male types deposited in AMNH, female paratype in AUMNH.

#### Etymology.

The specific epithet is a patronym in honor of author and environmentalist Edward P. Abbey (1927-1989).

#### Diagnosis.

Males can be diagnosed on the basis of a unique conformation of the distal most spination pattern of tibia I which consists of 4-7 short spines that overlap (Fig. 259). This spination pattern is most similar to *Aptostichus cahuilla*, however *Aptostichus edwardabbeyi* males are lighter in coloration and are larger. Known only from southeastern Arizona.

#### Description of male holotype.

*Specimen preparation and condition*. Specimen collected live, wandering, preserved in 70% EtOH. Coloration faded, specimen in relatively poor condition. Pedipalp, leg I left side removed, stored in vial with specimen. *General coloration*. Carapace, chelicerae, legs yellowish red 5YR 4/6. Abdomen strong brown 7.5YR 4/6, light mottled markings dorsally. *Cephalothorax*. Carapace 5.00 long, 4.40 wide, lightly hirsute with intermingled thin white, black setae; stout black bristles along fringe; surface smooth, pars cephalica elevated. Fringe, posterior margin with black bristles. Foveal groove deep, only slightly procurved. Eyes on low mound. AER slightly procurved, PER slightly recurved. PME, AME subequal diameter. Sternum moderately setose, STRl 2.80, STRw 2.38. Posterior sternal sigilla large, positioned towards center but not contiguous, anterior sigilla pairs small, oval, marginal. Chelicerae with distinct anterior tooth row comprising 6 teeth, posterior margin with patch of small denticles. Palpal endites with patch of small cuspules on proximal, inner margin, labium with 3 cuspules, LBw 0.80, LBl 0.43. Rastellum consists of 6 stout spines not on prominent mound, one spine offset prolaterally. *Abdomen*. Setose, heavy black setae intermingled with fine black setae. *Legs*. Leg I: 4.81, 3.30, 3.20, 2.10, 1.75; leg IV: 4.81, 2.28. Light tarsal scopulae on all legs, light scopulae on metatarsus I, II. Tarsus I with single, slightly staggered row of 12 trichobothria. Leg I spination pattern illustrated in Figures 259, 260; TSp 4, TSr 3, TSrd 4. *Pedipalp*. Articles slender, lacking distinct spines (Fig. 261). PTw 0.85, PTl 2.23, Bl 1.09. Embolus slender, tapering sharply toward tip, lacking serrations (Fig. 261).

**Variation (2).** Cl 4.60-5.00, Cw 3.95-4.40, STRl 2.75-2.8, STRw 2.15-2.38, LBw 0.80-0.82, LBl 0.43-0.43, leg I: 4.60-4.85, 3.16-3.30, 3.00-3.20, 1.70-2.10, 1.63-1.75; PTl 2.13-2.23, PTw 0.85-0.94, Bl 1.02-1.09, TSp 4-4, TSr 3-3, TSrd 4-8.

#### Description of female paratype.

*Specimen preparation and condition*. Female collected at night, wandering, prepared in same manner as male holotype. Genital plate removed, cleared in trypsin, stored in microvial with specimen. *General coloration*. Carapace, legs, chelicerae, strong brown 7.5YR 4/6. Abdomen brown dorsally 7.5YR 4/3, distinct mottled chevron marking pattern (Fig. 257). *Cephalothorax*. Carapace 5.25 long, 4.55 wide, lightly hirsute with fine black setae; generally smooth surface, pars cephalica moderately elevated. Fringe lacks setae. Foveal groove deep, slightly procurved. Eye group slightly elevated on low mound. AER slightly procurved, PER slightly recurved. PME-AME subequal diameter. Sternum widest at coxae II/III, moderately setose, STRl 3.16, STRw 2.64. Three pairs of sternal sigilla anterior pairs small in size, oval, marginal; posterior pair large, oval, mesially positioned but not continuous. Chelicerae anterior tooth row comprising 6 teeth with posterior margin denticle patch. Palpal endites with 37 cuspules concentrated at the inner (promargin) posterior heel; labium with 4 cuspules, LBw 1.07, LBl 0.60. Rastellum consists of 8 very stout spines not positioned on mound, one spine offset prolaterally; fringe of short spines along distal promargin extending upward from rastellum. *Abdomen*. Moderately setose. PLS all 3 segments with spigots. Terminal segment 1/2 length of medial segment, 2 enlarged spigots visible at tip. PMS single segment, with spigots, short with rounded terminus. *Legs*. Anterior two pairs noticeably more slender than posterior pairs. Leg I 11.46 long. Tarsus I with 15 trichobothria arranged in three staggered rows. Legs I, II with moderately heavy scopulae on tarsus, metatarsus; light scopulae on distal aspect tarsus legs III, IV. PTLs 12, TBs 4. Rudimentary preening comb on retrolateral distal surface, tarsus-metatarsus joint, of metatarsus III, IV. *Spermathecae*. 2 simple spermathecal bulbs with elongate curved stalk; basal extension small (Fig. 258).

#### Material examined.

Known only from the type material.

#### GenBank accession.

16S-tRNAval-12S: JX103301

#### Distribution and natural history.

Known only from a few specimens from the type locality in Cochise County, Arizona.

#### Conservation status.

Undetermined but likely to be evaluated as vulnerable or imperiled as a consequence of its rarity in collections and restricted distribution.

#### Species concept applied.

Morphological.

### Aptostichus anzaborrego sp. n.

‘The Anza-Borrego Desert State Park Trapdoor Spider’

urn:lsid:zoobank.org:act:5A3F1065-2EB3-4589-8F08-CE9726C17B71

http://species-id.net/wiki/Aptostichus_anzaborrego

**Figures 262–265.**
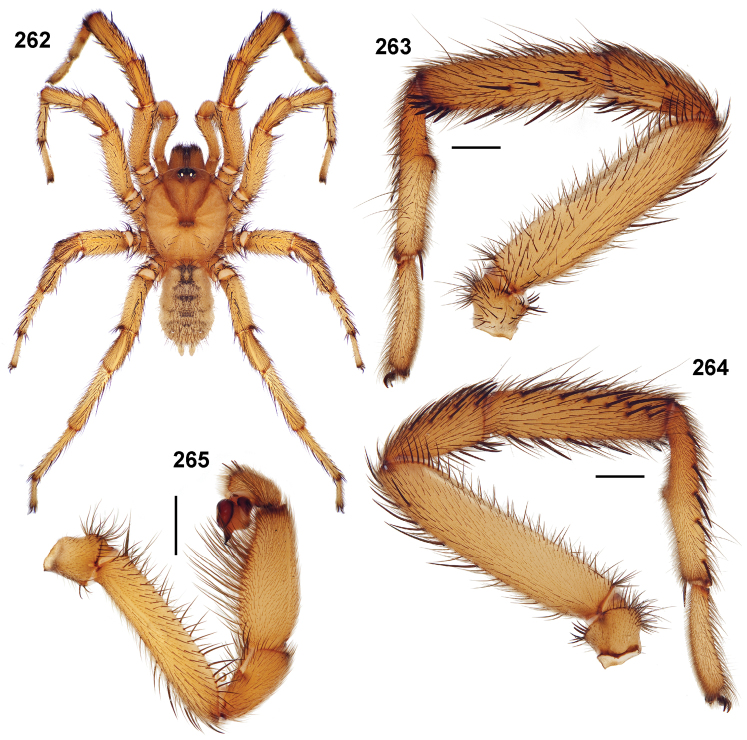
*Aptostichus anzaborrego* sp. n., male holotype from San Diego County (AP1199); scale bars = 1.0mm. **262** habitus [806106] **263** retrolateral aspect, leg I [806102] **264** prolateral aspect, leg I [806098] **265** retrolateral aspect, pedipalp [806104].

Figures 262–265 Map 30

#### Types.

Male holotype and male paratypes (AP1199) from California, San Diego County, Anza-Borrego Desert State Park, Indian Gorge, ~4.8km W of Hwy S-2, just W of Torote Canyon, 32.8685, -116.2380^1^, 351m, coll. M. Hedin 27-29.xi.2002; deposited in AUMNH.

#### Etymology.

The specific epithet is a noun taken in apposition from the type locality, Anza-Borrego Desert State Park.

#### Diagnosis.

Males can be diagnosed on the basis of a unique conformation of the retrolateral distal-most spination pattern of tibia I, which comprises 8-10 short overlapping spines (Fig. 263). This spination pattern is most similar to *Aptostichus cahuilla*, however *Aptostichus anzaborrego* males are lighter in coloration, have very light dorsal abdominal markings (Fig. 262), and have numerous spines on the prolateral surface tibia and patella I (Fig. 264).

#### Description of male holotype.

*Specimen preparation and condition*. Specimen collected live, wandering, preserved in 80% EtOH. Coloration and specimen in relatively good condition. Pedipalp, leg I left side removed, stored in vial with specimen. *General coloration*. Carapace, chelicerae, strong brown 7.5YR 4/6. Abdomen brown 7.5YR 5/4, light dorsal markings (Fig. 262). *Cephalothorax*. Carapace 5.15 long, 4.50 wide, very lightly hirsute with intermingled thin white, black setae; stout black bristles along fringe; surface smooth, pars cephalica elevated. Fringe, posterior margin with black bristles. Foveal groove deep, straight. Eyes elevated on high mound. AER slightly procurved, PER strongly recurved. PME, AME subequal diameter. Sternum moderately setose, STRl 3.00, STRw 2.50. Posterior sternal sigilla small in size, positioned towards margin, not contiguous, anterior sigilla pairs small, oval, marginal. Chelicerae with distinct anterior tooth row comprising 6 teeth, posterior margin with patch of small denticles. Palpal endites with patch of small cuspules on proximal, inner margin, labium with 2 cuspules, LBw 0.87, LBl 0.44. Rastellum consists of 7 stout spines not on prominent mound, two spines offset prolaterally. *Abdomen*. Setose, heavy black setae intermingled with fine black setae. *Legs*. Leg I: 5.45, 3.90, 3.68, 2.26, 1.75; leg IV: 5.10, 2.60. Tarsus I, swollen distally, tarsus IV straight. Light tarsal scopulae on all legs, light scopulae on metatarsus I, II. Tarsus I with single, slightly staggered row of 15 trichobothria. Leg I spination pattern illustrated in Figures 263, 264; TSp 9, TSr 7, TSrd 7. *Pedipalp*. Articles slender, lacking distinct spines (Fig. 265). PTw 1.04, PTl 2.48, Bl 1.29. Embolus slender, tapering sharply toward tip, lacking serrations (Fig. 265).

**Variation (2).** Cl 4.88-5.15, Cw 4.24-4.50, STRl 2.88-3.00, STRw 2.38-2.50, LBw 0.85-0.87, LBl 0.44-0.51, leg I: 4.75-5.45, 3.41-3.9, 3.26-3.68, 2.08-2.26, 1.55-1.75; leg IV: 4.60-5.10, 2.17-2.60; PTl 2.17-2.48, PTw 1.02-1.04, Bl 1.19-1.29, TSp 5-9, TSr 2-7, TSrd 7-9.

#### Description of female.

Known only from male specimens.

#### Material examined.

Known only from the type material.

#### Distribution and natural history.

*Aptostichus anzaborrego* is known from only two specimens from the Anza-Borrego Desert State Park, Colorado Desert habitat, in San Diego County. Based on the limited data available males appear to disperse during the winter months (November).

#### Conservation status.

The conservation status of *Aptostichus anzaborrego* is likely imperiled given its very restricted distribution and rarity in collections.

#### Species concept applied.

Morphological.

### Aptostichus sinnombre sp. n.

‘The No-name Trapdoor Spider’

urn:lsid:zoobank.org:act:2ADC46DE-8C5E-4D67-B8A7-C60522405025

http://species-id.net/wiki/Aptostichus_sinnombre

Figures 266–273 Map 30

#### Types.

Male holotype (MY3761) from California, San Diego County, Anza-Borrego Desert State Park, Indian Gorge, 0.4km W mouth of Torote Canyon, 32.86923, -116.23740^1^, 350m, coll. M. Hedin 20.ii.2009; male paratype (MY3823) from near type locality, mouth of canyon Sin Nombre, 32.8584, -116.1473^1^, 200m, coll. M. Hedin 13.iii.2010; deposited in AUMNH.

#### Etymology.

The specific epithet is a noun in apposition taken from name of canyon near the type locality, Sin Nombre.

#### Diagnosis.

**Figures 266–272.**
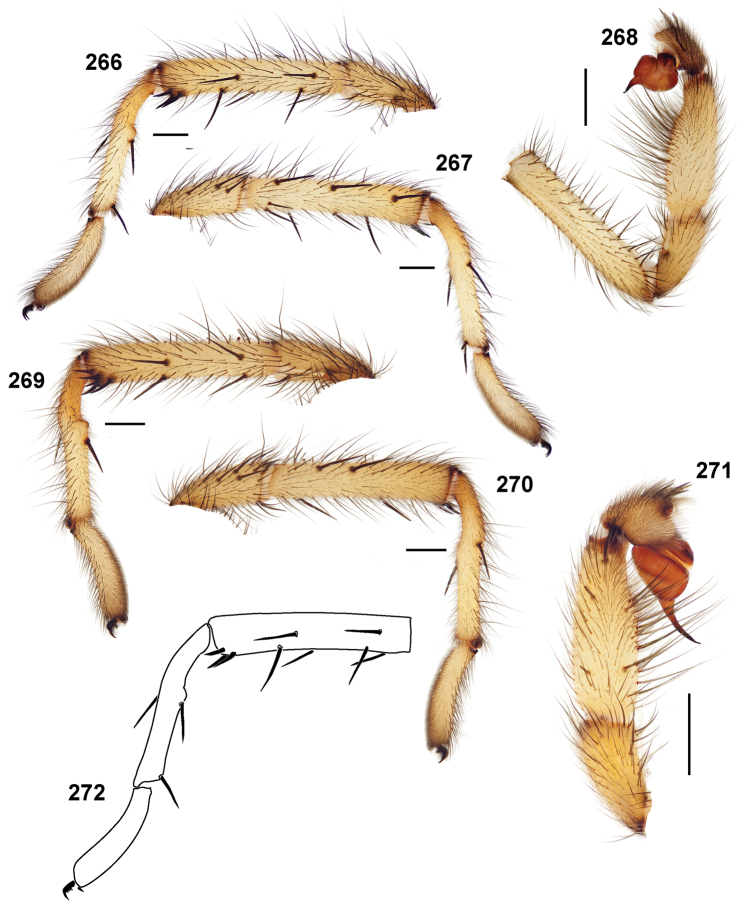
*Aptostichus sinnombre* sp. n., male specimens from San Diego County; scale bars = 1.0mm. **266–268** male holotype (MY3761) **266** retrolateral aspect, leg I [806546] **267** prolateral aspect, leg I [806550] **268** retrolateral aspect, pedipalp [806552] **269–271** male paratype (MY3823) **269 **retrolateral aspect, leg I [806554] **270** prolateral aspect, leg I [806558] **271** retrolateral aspect, pedipalp [806560] **272** line drawing, spination pattern, leg I tibia and metatarsus of holotype.

Males can be differentiated from all other *Aptostichus* species on the basis of a unique conformation of the retrolateral distal-most spination pattern of tibia I, which comprises 3-6 spines arranged in a distinct cluster, a low rounded metatarsal mating apophysis that bears a single strong spine, and a convex shaped (curved) tarsus (Figs 266, 269, 272); all other known species lack this combination.

#### Description of male holotype.

**Figure 273.**
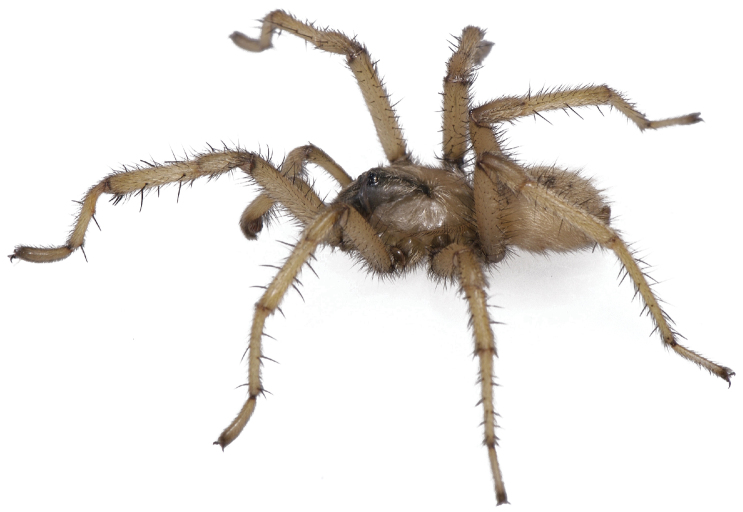
Live photograph, *Aptostichus sinnombre* sp. n. male holotype (MY3761); note leg I, right hand side missing.

*Specimen preparation and condition*. Specimen collected live, wandering, preserved in 80% EtOH. Coloration and specimen in relatively good condition. Pedipalp, leg I left side removed, stored in vial with specimen; legs right side removed for tissue storage. *General coloration*. Carapace, chelicerae, yellowish brown 10YR 5/4. Abdomen light yellowish brown 10YR 6/4, light dorsal chevron markings (Fig. 273). *Cephalothorax*. Carapace 5.75 long, 5.05 wide, very hirsute with intermingled thin white, black setae; stout black bristles along fringe; surface smooth, pars cephalica elevated. Fringe, posterior margin lacks black bristles. Foveal groove deep, straight. Eyes elevated on low mound. AER slightly procurved, PER strongly recurved. PME’s smaller in diameter than AME’s. Sternum moderately setose, STRl 3.10, STRw 2.67. Posterior sternal sigilla moderate in size, positioned towards center, not contiguous, anterior sigilla pairs small, oval, marginal. Chelicerae with distinct anterior tooth row comprising 7 teeth, posterior margin with patch of small denticles. Palpal endites with patch of small cuspules on proximal, inner margin, labium lacks cuspules, LBw 0.97, LBl 0.65. Rastellum consists of 3 spines not on prominent mound. *Abdomen*. Setose, heavy black setae intermingled with fine black setae. *Legs*. Legs long; Leg I: 6.88, 5.19, 4.35, 2.88, 1.90; leg IV: 6.19, 3.05. Tarsus I, swollen distally, tarsus IV straight. Heavy short scopulae on tarsi I, II; lighter on legs III, IV. Tarsus I with single, slightly staggered row of 13 trichobothria. Leg I spination pattern illustrated in Figures 266, 267, 269, 270, 272; TSp 4, TSr 4, TSrd 3. Knob-like metatarsal apophysis bearing a single large spine. *Pedipalp*. Articles slender (Fig. 268), distal prolateral spine on tibia (Fig. 271). PTw 0.85, PTl 2.57, Bl 1.45. Embolus slender, tapering sharply toward tip, lacking serrations (Fig. 271).

**Variation (2).** Cl 5.25-5.75, Cw 4.70-5.05, STRl 3.10-3.13, STRw 2.57-2.67, LBw 0.90-0.97, LBl 0.56-0.65, leg I: 6.38-6.88, 4.70-5.19, 4.15-4.35, 2.67-2.88, 1.75-1.90; leg IV: 5.88-6.19, 2.92-3.05; PTl 2.38-2.57, PTw 0.85-0.85, Bl 1.45-1.53, TSp 2-4, TSr 4-4, TSrd 3-6.

#### Description of female.

Known only from male specimens.

#### Material examined.

Known only from the type material.

#### Distribution and natural history.

*Aptostichus sinnombre* is known from only two specimens from the Anza-Borrego Desert State Park, Colorado Desert habitat, in San Diego County and is sympatric with *Aptostichus anzaborrego*. Males appear to disperse during late winter months (February, March), based on the limited data available.

#### Conservation status.

The conservation status of *Aptostichus sinnombre* is likely imperiled given its restricted distribution and rarity in collections.

#### Species concept applied.

Morphological/Phylogenetic.

##### The *Simus* species group

**Included Species.**

*Aptostichus simus* Chamberlin, 1917

*Aptostichus satleri* sp. n.

*Aptostichus elisabethae* sp. n.

*Aptostichus fornax* sp. n.

*Aptostichus lucerne* sp. n.

*Aptostichus bonoi* sp. n.

*Aptostichus fisheri* sp. n.

*Aptostichus cajalco* sp. n.

### Aptostichus simus

Chamberlin, 1917

‘The Southern Coastal Dune Trapdoor Spider’

urn:lsid:zoobank.org:act:D5D006A2-9041-4118-8EE2-C49B2CCC02D9

http://species-id.net/wiki/Aptostichus_simus

**Figures 274–280.**
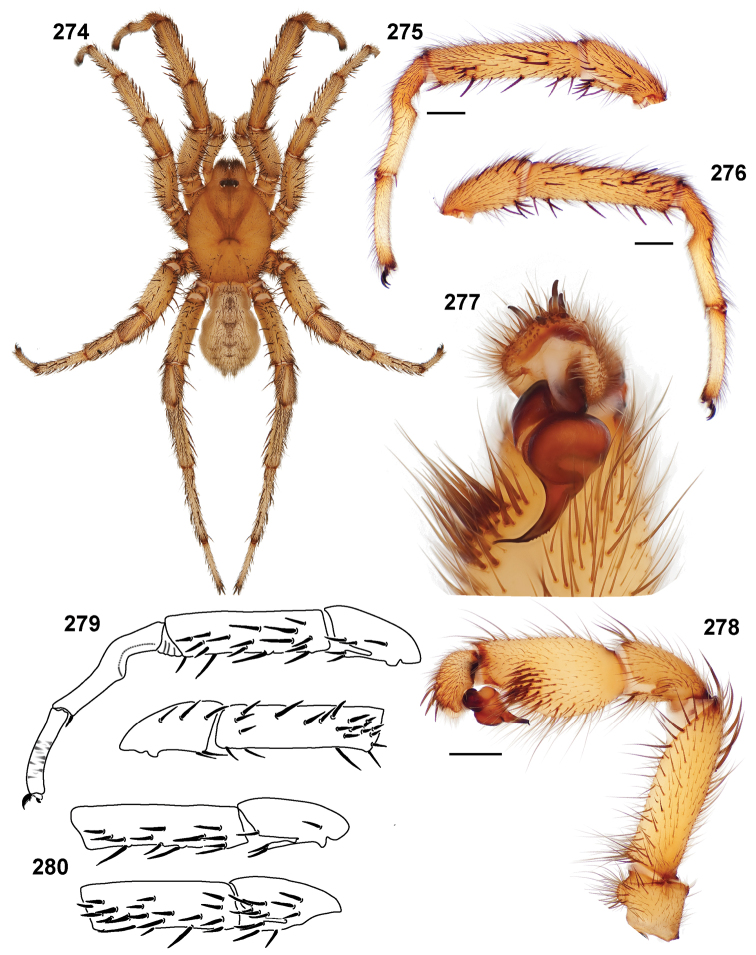
*Aptostichus simus* Chamberlin, 1917 male specimens from San Diego County; scale bars = 1.0mm. **274** habitus (AP1209) [806521] **275–278** secondary sexual characteristics (AP819) **275** retrolateral aspect, leg I [806525] **276** prolateral aspect, leg I [806529] **277** ventral view, pedipalp bulb [806531] **278** retrolateral aspect, pedipalp [806533] **279, 280** line drawings, leg I articles **279** retrolateral and prolateral aspect of specimen from San Diego County, Imperial Beach **280** retrolateral aspect, tibia and patella, in descending from San Diego County, Imperial Beach and Santa Barbara County, Carpinteria State Beach.

**Map 32.**
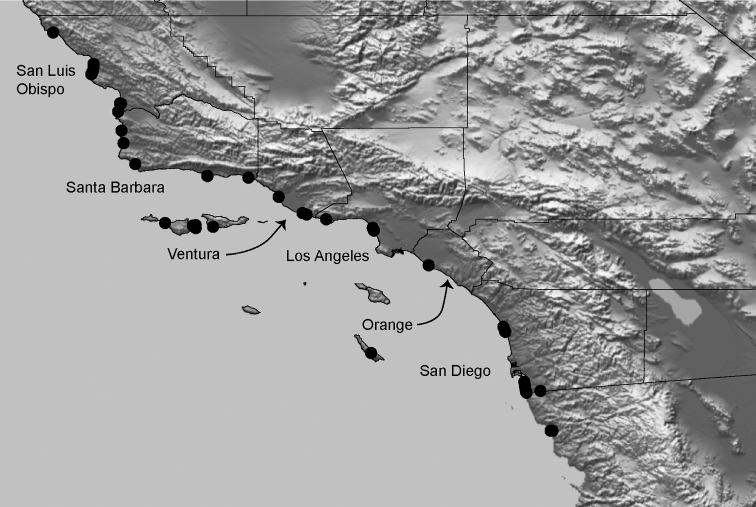
Known distribution of *Aptostichus simus*. Monterey County localities not shown.

Figures 274 [Fig F73] –284 Map 32

Aptostichus simus Chamberlin, 1917: 36. female holotype (No. 9), California, San Diego County Co., Silverstrand State Beach, 32.6346, -117.1400^4^, 1m, coll. G.R. Crotch, in MCZ, examined.

#### Diagnosis.

Males of *Aptostichus simus* can be distinguished from species in the *Simus* species group by having a serrated embolus (Figs 50, 277) and lacking both elongate ventral tibia I spines and ventral tarsal spines (Figs 275, 276, 279). Females can be easily recognized by having a very large (> 150), sharply delineated patch of endite cuspules (Fig. 39, 283). The species is the only member of the *Simus* group that has been collected from coastal dune habitat.

#### Descriptions.

Described by Chamberlin (1917: 36).

#### Material examined:

**Mexico: Baja California:** El Descanso, Descanso Dunes, 32.2074, -116.9125^5^, 22m, D Weissman, V Lee 3.iii.1992 [AP63, 89, 2♀, CAS]; **United States: California: Los Angeles County:** San Clemente Island, 32.878, -118.464^6^, 500m, J Scott 1.vi.1938 [AP093, 1♀, CAS]; S Huntington Beach, 33.6354, -117.9656^3^, 3m, W Gertsch, W Ivie 28.ix.1961 [AP628, 1♀, 1♂, AMNH]; Dunes on beach just W Los Angeles Airport, 33.9361, -118.4391^5^, 10m, W Icenogle 30.viii.1973 [AP073, 1♀, AMNH]; Los Angeles, El Segundo Sand Dunes, 33.939, -118.439^4^, 35m, J George 9.xii.1987 [AP441, 442, 2♂, CAS]; Playa Del Rey Beach, sand dunes between intersection 66th & Pacific & Pacific & Argonaut St, 33.959, -118.4491^3^, 4m, M Ramirez, H David 6.xi.1982 [AP58, 78, 611, 2juv, 1♀, 1♂, AMNH]; Malibu, Broad Beach, 34.0338, -118.8517^3^, 4m, M Ramirez [AP622, 626, 2♀, CAS]; **Monterey Co.:** Salinas River St Beach, 36.7831, -121.7944^1^, 3m, J Bond 6.v.1997 [AP757, 1♀, AMNH], [AP777, 1264, 1♀, 1juv, AUMNH]; Moss Landing St Beach, 36.8086, -121.7883^1^, 3m, J Bond 14.v.1997 [AP624, 794, 807, 808, 3♀, 1juv, AUMNH], [AP796, 1♀, AMNH]; Moss Landing St Beach, beachside dunes, 36.8091, -121.7883^1^, 1m, J Bond, W Bond 27.vii.2008 [MY3749, 1♀, AUMNH]; Moss Landing St Beach, 36.8109, -121.7894^1^, 6m, J Bond 17.iii.2005 [MY3080, 1♀, AUMNH], [MY3081, 1♀, FMNH], 8.xii.2005 [MY3466, 1♀, AUMNH], A Stockman, P Marek 30.i.2006 [MY3534, 1♀, FMNH]; **San Diego Co.:** Border Field St Park, S Monument Blvd, just N international border, 32.5351, -117.1191^1^, 16m, USGS-BRD San Diego Sta. 16.i.2003 [AP917, 1♂, CAS], 1.ii.2000 [AP918, 1♀, CAS]; Border Field St Park, S Monument Blvd., 32.5422, -117.1234^1^, 2m, R Fisher 2.x.1997 [AP819, 1♀, 4♂, CAS]; Border Field St Beach, 32.5436, -117.1227^1^, 3m, J Bond 19.x.1998 [AP699, 701, 707 3♀, AUMNH]; Borderfield St Beach, 32.5453, -117.1249^3^, 2m, W Icenogle 26.viii.1971 [AP054, 94, 2♀, 96juv, CAS], M Ramirez. H David 21.viii.1982 [AP071, 2 eggsacs, AMNH]; Tijuana Estuary, N Monument Blvd., E of beach, 32.5457, -117.1243^1^, 2m, USGS-BRD San Diego Field Sta. 1.x.1998 [AP1188-1190, 4♂, CAS]; Tijuana Estuary, N of Monument Blvd., E beach, 32.5479, -117.1249^1^, 2m, USGS-BRD San Diego Sta. 26.ix.2002 [AP1182, 3♂, CAS], 1.vii.2002, [AP1183, 1♂, CAS], 1.ix.2002 [AP1184, 3♂, CAS], 1.viii.1998 [AP1185, 1♀, CAS], 1.x.1998 [AP1186, 2♂, CAS], 1.xi.2002 [AP1187, 3♂, CAS]; Tijuana Estuary, N of Monument Blvd., E beach, 32.5503, -117.1261^1^, 1♂, USGS-BRD San Diego Sta. 1.x.1998 [AP1181, 2♂, CAS]; Otay Mesa, E Spring Canyon, N Wruck Canyon, 32.5515, -116.9998^1^, 150m, R Fisher 1.viii.2002 [AP820, 2♂, CAS]; Imperial Beach, 32.5805, -117.1325^3^, 0m, S Johnson 5.x.1978 [AP087, 4♀, AMNH], W Icenogle 2.x.1978 [AP091, 1♀, 62juv, AMNH], [AP097, 1♀, 58j, AMNH], 1.xi.1978 [AP598, 1♀, UCR]; N end Imperial Beach, just N YMCA Camp, 32.5871, -117.1319^2^, 0m, M Hedin 12.xi.2005 [AP1209, 1♂, CAS]; N end Imperial Beach, ~0.8km N YMCA surf camp, 32.5929, -117.1321^2^, 1m, M Hedin 23.ix.2002 [MY667, 1♂, AUMNH]; 0.4km N Imperial Beach, 32.59351, -117.1321^3^, 3m W Icenogle 1.x.1977 [AP201, 657, 2♂, CAS]; Silverstrand St Beach, 32.6265, -117.139^3^, 4m, M Ramirez, H David 4.xi.1982 [AP064, AP1260, 2♀, AMNH]; Silver Strand St Beach, 32.6267, -117.1391^3^, 2m, Parrish 11.v.1963 [AP088, 1♀, AMNH]; J Bond 27.iii.96, [AP1256, 1257, 3♀, AUMNH]; 0.4km S Ponto St Beach, 33.0608, -117.3027^3^, 3m, W Icenogle 22.x.1970 [AP056, 1♀, 41juv, AMNH], 8.v.1970 [AP095, 2♀, CAS]; Encinitas, Ponto St Beach, 33.0668, -117.3065^3^, 4m, B Kaston 22.x.1970 [AP096, 1♀, 40juv, CAS]; Leucadia, sand dunes on beach, 33.0655, -117.3051^3^, 3m, B Kaston 30.i.1971 [AP065, 1♀, AMNH], 26.ix.70 [AP651, 1♀, 50+juv, AMNH]; Encinitas, Ponto St Beach, 33.0668, -117.3065^3^, 4m, B Kaston 22.x.1970 [AP055, 1♀, 20juv, CAS], W Icenogle 28.x.1970 [AP059, 28juv, AMNH], [AP067, 1♀, 15juv, CAS], [AP069, 1♀, CAS], [AP075, 1♀, 32juv, AMNH], 26.ix.1970 [AP082, 1♀, 83juv, CAS], [AP090, 1♀, 6juv, AMNH]; S Carlsbad St Beach, 33.1038, -117.3191^1^, 3m, J Bond 19.x.1998 [AP698, 709, 2♀, AUMNH]; **San Luis Obispo Co.:** Oso Flaco Lake, 35.0305, -120.6241^3^, 6m, M Irwin 22.vi.1965 [AP061, 4juv, AMNH]; Montana de Oro St Park, 35.2854, -120.8809^1^, 29m, A Stockman, A Bailey 8.vii.2009 [MY3704-3706, 3juv, AUMNH]; Morro Dunes, S end Morro Bay, 35.3058, -120.8728^1^, 20m, J Bond 6.xii.2005 [MY3436-3440, 4♀, 1juv, AUMNH]; N end Morro Bay, 35.3748, -120.8631^1^, 4m, J Bond 6.xii.2005 [MY3441-3443, 3♀, AUMNH]; Baywood, 35.3083, -120.8683^3^, 3m, P Sullivan 26.xi.1977 [AP621, 1♂, AMNH]; Morro Bay, beach N Morro Rock, 35.3739, -120.8613^1^, 0m, M Hedin, S Foldi 3.iv.2005 [AP1208, 1♀, AUMNH]; Estcro Bay, 35.3453, -120.8624^6^, 10m, 1.vi.1975 [AP068, 1♀, CAS]; isolated dune field just N San Simeon Bay, 35.6466, -121.211^1^, 3m, J Bond 6.xii.05 [MY3447, 3449, 2♀, AUMNH]; **Santa Barbara Co.:** Santa Rosa Island E of marsh road to E Pt, 33.9572, -119.9784^5^, 14m, M Ramirez, H David 9.viii.1994 [AP079, 80, 594, 3♀, 1juv, AMNH]; Santa Rosa Island, SE Anchorage, 33.9787, -120.0034^5^, 19m, M Ramirez, H David 30.viii.1988 [AP053, 1♀, 29juv, AMNH], [AP081, 1♀, AMNH], 9.viii.1994 [AP083, 1♀, AMNH], 1.vii.1987 [AP092, 1♀, CAS]; Santa Rosa Island, dunes Skunk Pt, 33.9821, -119.9793^4^, 15m, M Ramirez, H David 11.viii.1994 [AP050, 57, 1261, 2♀, 3juv, AMNH]; Santa Rosa Island, dunes E Sandy Pt, 34.0003, -120.243^4^, 24m, M Ramirez, H David 8.viii.1994 [AP052, 3juv, AMNH]; N Carpinteria St Beach, 34.393, -119.5239^3^, 3m, W Gertsch, W Ivie 27.ix.1961 [AP659, 1♀, 1♂, AMNH]; Coal Oil Pt, 34.4072, -119.8772^1^, 3m, J Bond, B Opell 21.xi.1998 [AP689, 697, 702, 3♀, AUMNH], [AP695, 700, 2♀, FMNH], [AP696, 1♀, AMNH], M Ramirez, H David 24.vi.82 [AP070, 72, 76, 86, 623, 4♀, 1eggsac, AMNH], J Bond 5.iv.1996 [AP721, 735, 744, 2♀, 1juv, AUMNH], [AP743, 1♀, FMNH]; Ocean Beach Co Park, W of Lompoc, W end HWY 246, 34.69, -120.6031^3^, 2m, W Icenogle 12.viii.1978 [AP074, 1♀, 62juv, AMNH]; Vandenberg Air Force Base, dunes mouth San Antonio Creek, 34.7989, -120.6199^4^, 11m, 25.v.1976 [AP051, 1juv, CAS]; Jalama Co Park, 34.5098, -120.5011^1^, 4m, J Bond, A Stockman, D Beamer 17.iii.2005 [MY3422-24, 2♀, 1juv, AUMNH]; Ocean Park Dunes, 34.69, -120.6031^1^, 2m, J Bond, A Stockman, D Beamer 17.iii.2005 [MY3425-27, 3♀, AUMNH]; Guadalupe-Nipomo Dunes Preserve, 34.9624, -120.6497^1^, 11m, J Bond, A Stockman, D Beamer 17.iii.05 [MY3428, 1♀, 15juv, AUMNH]; J Bond, A Stockman, D Beamer 17.iii.05 [MY3429, 1♀, AMNH]; J Bond, A Stockman, D Beamer 17.iii.05 [MY3430, 1juv, AUMNH]; Oso Flaco Lake Preserve, 35.0341, -120.6326^1^, 6m, J Bond 17.iii.2005 [MY3431-34, 4♀, AUMNH], [MY3435, 1♀, AMNH]; **Santa Cruz Co.:** Santa Cruz Island, Johnson’s Lee Beach, 33.9684, -119.8291^3^, 3m, 12.ix.1982 [AP077, 1♀, 7juv, AMNH], M Ramirez 15.i.1983 [AP653, 1♂, AMNH], 3.x.1987 [AP658, 1♀, 2♂, CAS]; **Ventura Co.:** Pt Mugu St Park, Sycamore Cove Beach, 34.0742, -119.0207^3^, 3m, M Ramirez [AP625, 627, 629, 3♀, CAS]; Pt Mugu, 34.0863, -119.05562^5^, 25m, D Boe 16.vi.1979 [AP596, 1♀, UCR]; McGrath St Beach, 34.2264, -119.2614^3^, 7m, M Ramirez, H David 25.vi.1982 [AP062, 1♀, 2juv, AMNH].

**Variation, males (5).** Cl 4.88-6.19, 5.61±0.21; Cw 4.31-5.19, 4.76±0.16; STRl 2.79-3.25, 3.01±0.09; STRw 2.40-2.85, 2.64±0.08; LBw 0.80-0.92, 0.84±0.02; LBl 0.45-0.63, 0.54±0.03; leg I: 5.00-5.22, 5.13±0.04; 3.69-4.06, 3.81±0.07; 3.13-3.50, 3.30±0.06; 2.06-2.44, 2.26±0.07; 1.50-1.86, 1.70±0.08; leg IV: 4.75-5.25, 5.03±0.08; 2.69-3.38, 3.10±0.12; PTl 1.68-1.95, 1.79±0.04; PTw 1.02-1.14, 1.09±0.02; Bl 0.80-0.86, 0.83±0.01; TSp 14-29, 20.20±2.62; TSr 12-21, 16.4±1.72; TSrd 0-0, 0±0.

**Variation, females (32).** Cl 4.81-8.75, 6.91±0.19; Cw 3.66-7.06, 5.70±0.15; STRl 2.64-4.88, 3.87±0.10; STRw 2.31-4.31, 3.39±0.09; LBw 0.81-1.64, 1.19±0.04; LBl 0.60-1.04, 0.82±0.02; Leg I: 10.53-19.50, 15.65±0.41; ANTd 4-5, 4.22±0.07; PTLs 15-29, 20.75±0.69; TBs 2-8, 5.66±0.32.

**Figures 281–283. F73:**
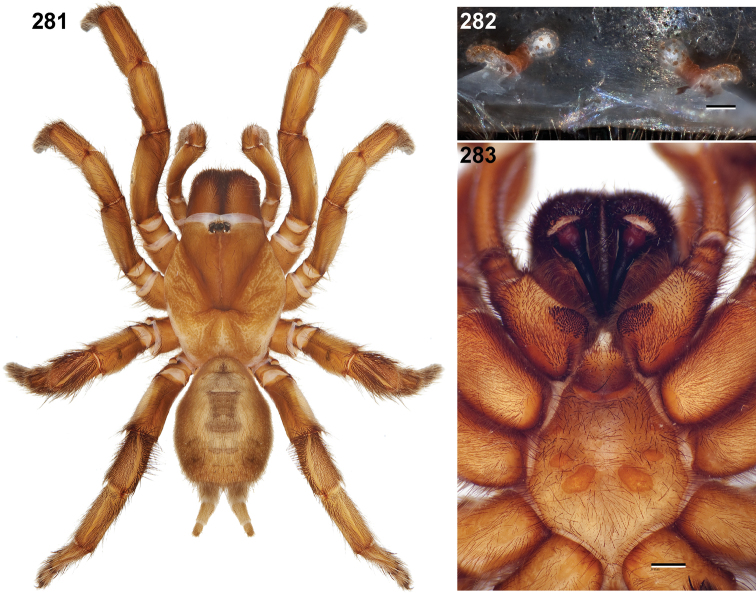
*Aptostichus simus* female specimens. **281** habitus, from San Diego County (AP067) **282** cleared spermathecae (MY3447), from San Luis Obispo County [806538]; scale bar = 0.1mm **283 **cephalothorax, ventral aspect, from San Diego County (AP095) [806542].

**Figure 284.**
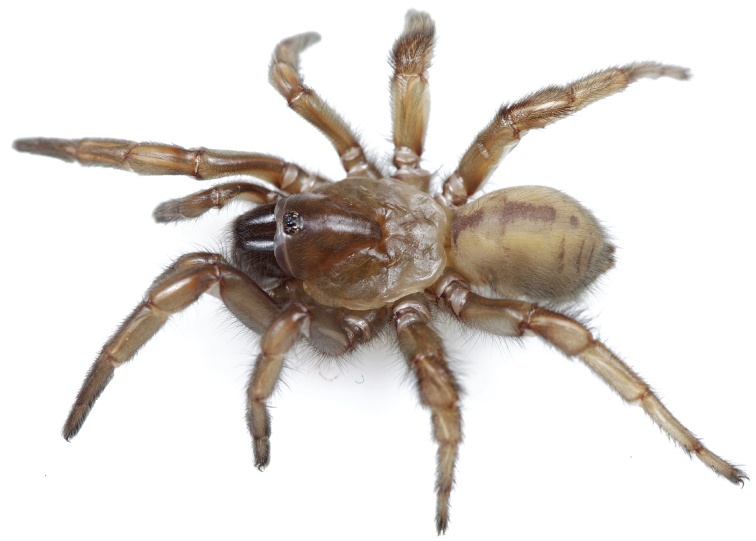
Live photograph, *Aptostichus simus* from Monterey County (AUMS022).

#### GenBank accessions.

16S: AF307955-AF307969; 16S-tRNAval-12S: EU570038, JX103367-JX103400.

#### Distribution and natural history.

Like *Aptostichus stephencolberti* and *Aptostichus miwok*, *Aptostichus simus* is distributed throughout the California coastal dune habitats. Specimens are known from Baja California Norte, northward along beaches in San Diego, Orange, Los Angeles, Ventura, Santa Barbara, and San Luis Obispo Counties; their distribution ends just north of Point Conception with two disjunct populations in Monterey County and on the Channel Islands just off the California coast (Map 32). Like other coastal dune species, *Aptostichus simus* is found in relatively deep burrows on the steep faces of sand dunes and at the base of coastal vegetation. Burrows comprise a thick silk lining and are covered by a very cryptic trapdoor constructed of silk and sand. Dune habitats disturbed by high concentrations of the invasive *Carpobrotus edulis* (ice plant) tend to lack *Aptostichus simus*.

#### Conservation status.

*Aptostichus simus* is generally abundant at some localities along the California coast but has been almost entirely extirpated from many beaches in Southern California. For example, the species is very rare at the type locality, Silver Strand State Beach. Moreover, the species is particularly vulnerable to invasive plant species (e.g., ice plant, *Carpobrotus edulis*) and is highly structured genetically across its distribution thus many populations contain unique alleles (Bond et al. 2001). Consequently, I would consider this species to be vulnerable or imperiled due to its low abundance at many localities and extreme structuring.

#### Species concept applied.

Morphological/Phylogenetic.

#### Remarks.

As discussed by Bond et al. (2001) the species hypothesis presented here likely represents 2-3 additional species, however, further work is needed before we fully understand the composition and limits of this taxon.

### Aptostichus satleri sp. n.

‘The Radical Satler Trapdoor Spider’

urn:lsid:zoobank.org:act:371C5A4E-2CFC-4F58-9C6B-152071123F2B

http://species-id.net/wiki/Aptostichus_satleri

Figures 285–291 Map 33

#### Types.

Male holotype (MY3825) and male paratypes (MY3826, 3827) from California, Kern County, Erskine Creek Rd., 5.6km E or intersection with Lake Isabella Blvd., E of Bodfish, 35.5689, -118.4383^1^, 925m, coll. J. Satler 8-29x.2010; holotype and paratype (3826) deposited in AUMNH, paratype (3827 in CAS).

#### Etymology.

The specific epithet is a patronym in honor of Jordan Satler, collector of this very unique species.

#### Diagnosis.

**Figures 285–291.**
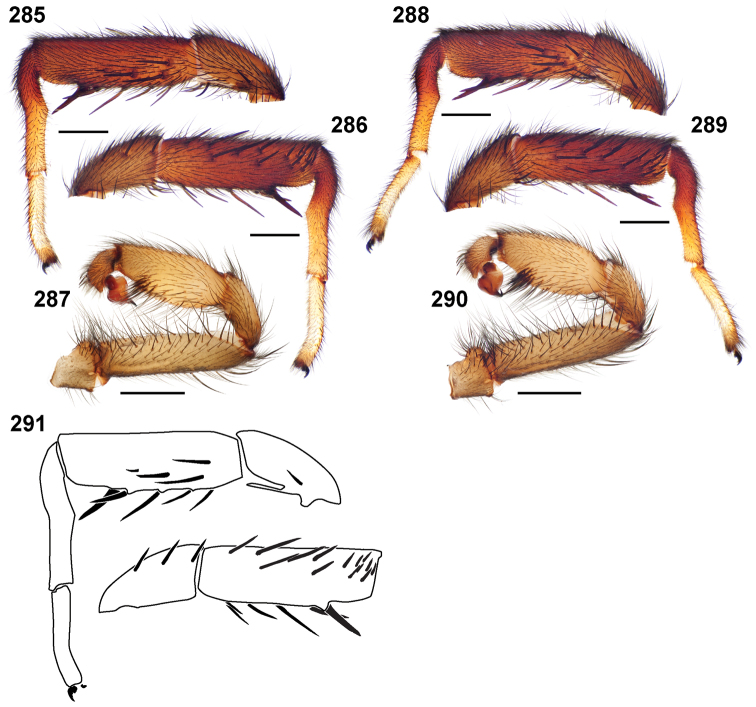
*Aptostichus satleri* sp. n. holotype (MY3825) and paratype (MY3826) males from Kern County; scale bars = 1.0mm. **285** retrolateral aspect, leg I [806495] **286** prolateral aspect, leg I [806499] **287** retrolateral aspect, pedipalp [806501] **288** retrolateral aspect, leg I [806503] **289** prolateral aspect, leg I [806507] **290** retrolateral aspect, pedipalp [806509] **291** line drawings of leg I spination pattern; retrolateral aspect of patella, tibia, metatarsus, tarsus; prolateral aspect of patella and tibia.

Males can be distinguished from all other known similar *Simus* group taxa (*Aptostichus lucerne*, *Aptostichus fornax*, and *Aptostichus elisabethae*) by virtue of their unique leg I spination pattern (Figs 285, 286, 288, 289, 291), darker coloration (non-desert adapted). The long leg I ventral tibial spine arrangement (Fig. 291) is similar to *Aptostichus elisabethae*, however, the palpal embolus of *Aptostichus satleri* is serrated whereas *Aptostichus elisabethae* is not. *Aptostichus fornax*, *Aptostichus lucerne*, and *Aptostichus elisabethae* are all much lighter in general coloration and lack distinct abdominal markings; *Aptostichus satleri* is much darker and has distinct chevron markings.

#### Description of male holotype.

*Specimen preparation and condition*. Specimen collected from pitfall trap, preserved in 80% EtOH; legs III, IV removed, stored as tissues. Coloration in generally pristine condition. Pedipalp, leg I left side removed, stored in vial with specimen. *General coloration*. Carapace, chelicerae, dark brown 10YR 3/3. Abdomen brown 10YR 5/3, with distinct chevron markings. *Cephalothorax*. Carapace 4.60 long, 3.76 wide, lightly hirsute; stout black bristles along fringe; surface smooth, pars cephalica elevated. Fringe, posterior margin with black bristles. Foveal groove deep, recurved. Eyes on low mound. AER slightly procurved, PER slightly recurved. PME, AME subequal diameter. Sternum moderately setose, STRl 2.53, STRw 2.17. Posterior sternal sigilla small in size, widely separated, anterior sigilla pairs small, oval, marginal. Chelicerae with distinct anterior tooth row comprising 4 teeth, posterior margin with patch of small denticles. Palpal endites, labium lack cuspules, LBw 0.68, LBl 0.51. Rastellum consists of 10 stout spines not on prominent mound. *Abdomen*. Setose, heavy black setae intermingled with fine black setae. *Legs*. Leg I: 4.16, 3.24, 2.43, 1.80, 1.25; leg IV: 4.45, 2.33. Tarsi curved; tarsus I with light pseudosegmentation. Very light (sparse) tarsal scopulae on all legs, light scopulae on metatarsus I, II. Tarsus I with single, slightly staggered row of 11 trichobothria. Leg I spination pattern illustrated in Figures 285, 286, 288, 289, 291; TSp 14, TSr 4, TSrd 0. *Pedipalp*. Articles stout, with distinct patch of distal prolateral tibial spines (Figs 287, 290). PTw 0.83, PTl 1.84, Bl 0.66. Embolus broad, tapering sharply toward tip, with serrations.

**Variation (3).** Cl 4.36-4.68, 4.55±0.10; Cw 3.56-3.76, 3.69±0.07; STRl 2.40-2.53, 2.48±0.04; STRw 2.08-2.17, 2.14±0.03; LBw 0.68-0.77, 0.71±0.03; LBl 0.37-0.51, 0.46±0.05; leg I: 3.88-4.20, 4.08±0.1; 3.01-3.24, 3.15±0.07; 2.43-2.43, 2.43±0.00; 1.78-1.80, 1.79±0.01; 1.25-1.28, 1.26±0.01; leg IV: 3.96-4.25, 4.14±0.09; 2.33-2.48, 2.41±0.04; PTl 1.80-1.84, 1.83±0.01; PTw 0.83-0.85, 0.84±0.01; Bl 0.66-0.68, 0.67±0.01; TSp 5-19, 12.67±4.10; TSr 4-12, 7.33±2.40; TSrd 0-0, 0±0.

#### Description of female.

Known only from male specimens.

#### Material examined.

**United States: California: Kern Co.:** Keyesville Recreation Area, off hwy 155, ~1.6km SW of Lake Isabella Dam, 35.6358, -118.4953^1^, 800m, J Satler 8–29.x.2010 [MY3825-3828, 4♂, AUMNH].

#### Distribution and natural history.

**Map 33.**
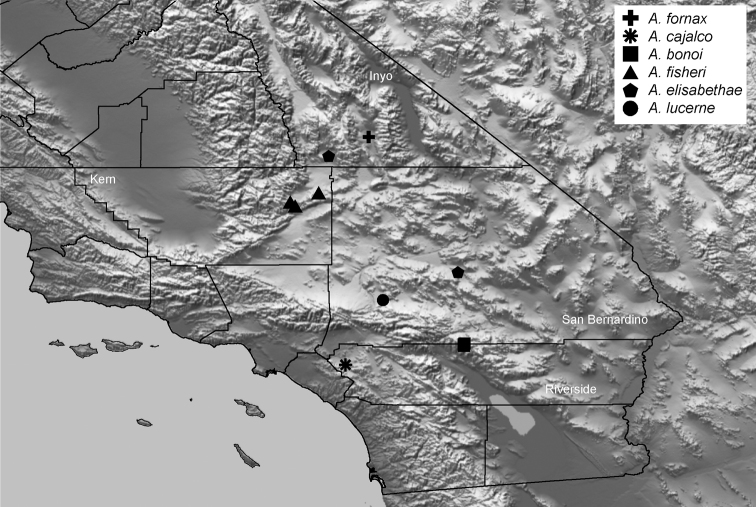
Distribution of *Aptostichus fornax*, *Aptostichus cajalco*, *Aptostichus chavezi*, *Aptostichus fisheri*, *Aptostichus elisabethae*, and *Aptostichus lucerne*.

Known only from the type locality in Kern County, which is characterized as Sierran Steppe-Mixed Forest (Map 33). Based on very limited data, collected from pitfall traps, males of this species appears to disperse during late October.

#### Conservation status.

The conservation status of *Aptostichus satleri* is undetermined.

#### Species concept applied.

Morphological.

#### Remarks.

This species came to my attention very late in the process of completing this revision thus very little is known about it. No known attempts have been made to locate and collect females at the type locality.

### Aptostichus elisabethae sp. n.

‘Elisabeth’s Desert Trapdoor Spider’

urn:lsid:zoobank.org:act:463C1178-9AC3-466E-86FE-73D289BEBDAB

http://species-id.net/wiki/Aptostichus_elisabethae

**Figures 292–297.**
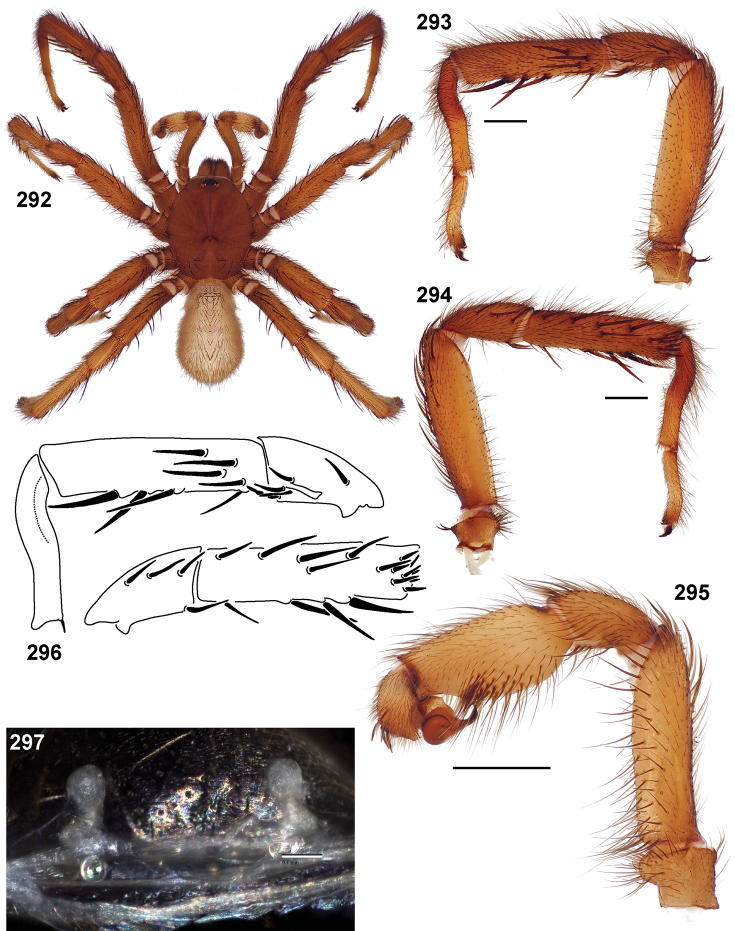
*Aptostichus elisabethae* sp. n. **292–295** male holotype (AP389) from San Bernardino County; scale bars = 1.0mm **292** habitus [806425] **293** retrolateral aspect, leg I [806429] **294** prolateral aspect, leg I [806431] **295** retrolateral aspect, pedipalp [806433] **296** line drawings of leg I spination pattern; retrolateral aspect patella, tibia, and metatarsus; prolateral aspect, patella and tibia **297** female paratype (AP1254), cleared spermathecae [806434]; scale bar = 0.1mm.

Figures 292–297 Maps 33

#### Types.

Male holotype (AP389) from California, San Bernardino County, Pisgah Crater, 34.7465, -116.3755^1^, 666m, coll. Norris & Heath 26.xi.1961 deposited in AMNH; female paratype (AP1254) from type locality, coll. J. Bond 27.i.1997; deposited in AUMNH.

#### Etymology.

The specific epithet is a patronym in honor of my beautiful daughter Elisabeth Morgen Bond.

#### Diagnosis.

Males are most similar to *Aptostichus satleri*, *Aptostichus lucerne* and *Aptostichus fornax* as a consequence of lacking any spines on the distal quadrant of the retrolateral aspect of the leg I tibia (Figs 293, 294, 296). They lack the larger retrolateral palpal tibial spines of *Aptostichus fornax* and the numerous prolateral tibia I spines (TSp) and embolus serrations of *Aptostichus lucerne* and *Aptostichus satleri*; *Aptostichus elisabethae* is much lighter in coloration and lacks the distinct abdominal markings (Fig. 292) of *Aptostichus satleri*. Females can be distinguished by having a sternum that is almost as long as it is wide and a rastellum composed of at least 8 large spines.

#### Description of male holotype.

*Specimen preparation and condition*. Specimen collected from pitfall trap, preserved in 80% EtOH. Coloration faded. Pedipalp, leg I left side removed, stored in vial with specimen. *General coloration*. Carapace, chelicerae, dark red 2.5YR 3/6. Abdomen very pale brown 10YR 7/4, lacking dorsal markings (Fig. 292). *Cephalothorax*. Carapace 3.84 long, 3.32 wide, glabrous; stout black bristles along fringe; surface smooth, pars cephalica elevated. Fringe, posterior margin with black bristles. Foveal groove deep, recurved. Eyes on low mound. AER slightly procurved, PER slightly recurved. PME’s smaller in diameter than AME’s. Sternum moderately setose, STRl 1.94, STRw 1.84. Posterior sternal sigilla moderate in size, widely separated, anterior sigilla pairs small, oval, marginal. Chelicerae with distinct anterior tooth row comprising 3 teeth, posterior margin with patch of small denticles. Palpal endites and labium lack cuspules, LBw 0.60, LBl 0.43. Rastellum consists of 10 stout spines not on prominent mound. *Abdomen*. Setose, heavy black setae intermingled with fine black setae. *Legs*. Leg I: 3.84, 3.24, 2.38, 1.52, 1.25; leg IV: 3.45, 2.05. Tarsi curved; tarsus I with light pseudosegmentation. Very light (sparse) tarsal scopulae on all legs, light scopulae on metatarsus I, II. Tarsus I with single, slightly staggered row of 9 trichobothria. Leg I spination pattern illustrated in Figures 293, 294, 296; TSp 14, TSr 4, TSrd 0. *Pedipalp*. Articles stout, with distinct patch of distal prolateral tibial spines (Fig. 295). PTw 0.75, PTl 1.53, Bl 0.68. Embolus broad, tapering sharply toward tip, lacking serrations.

**Variation (10).** Cl 3.75-4.69, 4.03±0.08; Cw 3.06-4.00, 3.29±0.08; STRl 1.83-2.37, 2.00±0.05; STRw 1.68-2.10, 1.82±0.04; LBw 0.53-0.62, 0.58±0.01; LBl 0.38-0.44, 0.41±0.01; leg I: 3.75-4.25, 3.87±0.05; 3.06-3.38, 3.14±0.03; 2.25-2.58, 2.35±0.03; 1.46-1.68, 1.52±0.02; 1.20-1.35, 1.25±0.01; leg IV: 3.27-3.81, 3.43±0.05; 1.86-2.38, 2.04±0.04; PTl 1.32-1.59, 1.43±0.03; PTw 0.68-0.84, 0.74±0.02; Bl 0.62-0.69, 0.65±0.01; TSp 8-16, 11.90±0.75; TSr 3-10, 6.2±0.61; TSrd 0-0, 0±0.

#### Description of female paratype.

*Specimen preparation and condition*. Female collected live from burrow, prepared in same manner as male holotype. Genital plate removed, cleared in trypsin, stored in microvial with specimen. *General coloration*. Carapace, legs, chelicerae, yellowish brown 10YR 5/4. Abdomen pale brown dorsally 10YR 6/3, very light chevron marking pattern. *Cephalothorax*. Carapace 3.44 long, 3.06 wide, generally glabrous, very sparse fine black setae; generally smooth surface, pars cephalica moderately elevated. Fringe lacks setae. Foveal groove deep, slightly procurved. Eye group slightly elevated on very low mound. AER slightly procurved, PER slightly recurved. PME’s smaller in diameter than AME’s. Sternum widest at coxae II/III, moderately setose, STRl 1.92, STRw 1.74. Three pairs of sternal sigilla anterior pairs small in size, oval, marginal; posterior pair moderate in size, oval, mesially positioned but not contiguous. Chelicerae anterior tooth row comprising 4 teeth with posterior margin denticle patch. Palpal endites with 45 cuspules concentrated at the inner (promargin) posterior heel; labium lacks cuspules, LBw 0.62, LBl 0.38. Rastellum consists of 8 stout spines not positioned on mound; fringe of short spines along distal promargin extending upward from rastellum. *Abdomen*. Moderately setose. PLS all 3 segments with spigots. Terminal segment 1/2 length of medial segment, 2 enlarged spigots visible at tip. PMS single segment, with spigots, short with rounded terminus. *Legs*. Anterior two pairs noticeably more slender than posterior pairs. Leg I 9.00 long. Tarsus I with 9 trichobothria arranged in wide row. Legs I, II with light scopulae on tarsus, metatarsus; light scopulae on distal aspect tarsus legs III, IV. PTLs 17, TBs 6. Rudimentary preening comb on retrolateral distal surface, tarsus-metatarsus joint, of metatarsus III, IV. *Spermathecae*. 2 simple spermathecal bulbs with short stalk; basal extension small (Fig. 297).

**Variation.** Known only from the type material.

#### Material examined.

**United States: California: San Bernardino Co.:** Pisgah Crater, 34.7465, -116.3755^1^, 666m, Norris, Heath 1.ii.1961-25.ii.1961, 26.xi.1961, 6.i.1963 [AP317-333, 389, 22♂, 1juv, AMNH]; J Bond 27.i.1997, [AP1254, 1258, 1♀, 1juv, AUMNH]; **Inyo Co.:** China Lake Naval Weapons Center, 35.9039, -117.6639^5^, 828m, G Pratt, C Pierce 15.ii.1997 [AP593, 1♂, UCR].

#### Distribution and natural history.

*Aptostichus elisabethae* is known from Mojave Desert habitat at two localities in San Bernardino and Inyo Counties (Map 33). Based on limited pitfall trap data males appear to disperse November-February. The habitat and terrain at the type locality, Pisgah Crater, is the most extreme I encountered for any *Aptostichus* species. The Pisgah Crater locality is the site of a young volcanic cinder cone from which basaltic lava flows once extended for a considerable distance out from the vent source (Fig. 6). Female burrows, during winter months are visible by way of small soil mounds at the burrow entrance. Like other species, we presume that these mounds are formed when individuals extend their burrows during the rainy reason. Despite observing a number of “mounds” at the type locality during one collecting expedition in January of 1997, I was only able to collect two specimens from their burrows. Burrows were deep (15–20 cm) and unusually complicated with numerous side chambers and long horizontal below ground extensions that were often >10cm in length.

#### Conservation status.

The conservation status of *Aptostichus elisabethae* would likely be considered vulnerable or imperiled; it is rare in collections, abundance is low, and it is relatively restricted in distribution (see comments below regarding Inyo County locality). The type locality is the site of a quarry (not presently active) where hectorite and volcanic cinders are mined.

#### Species concept applied.

Morphological.

#### Remarks.

I have included the single specimen from Inyo County as part of the *Aptostichus elisabethae* hypothesis. Although this specimen is similar to *Aptostichus elisabethae* it is sufficiently different to lead me to believe that if more specimens were available it would likely be considered a separate species. However, at this time I have conservatively decided to group these specimens as a single species. The female paratype, the only known female specimen collected for the species has very lightly sclerotized spermathecae and as such may not be fully mature.

### Aptostichus fornax sp. n.

‘The Furnace Trapdoor Spider’

urn:lsid:zoobank.org:act:50095886-C9E7-4098-84D7-ADB6911C7FCC

http://species-id.net/wiki/Aptostichus_fornax

**Figures 298–303.**
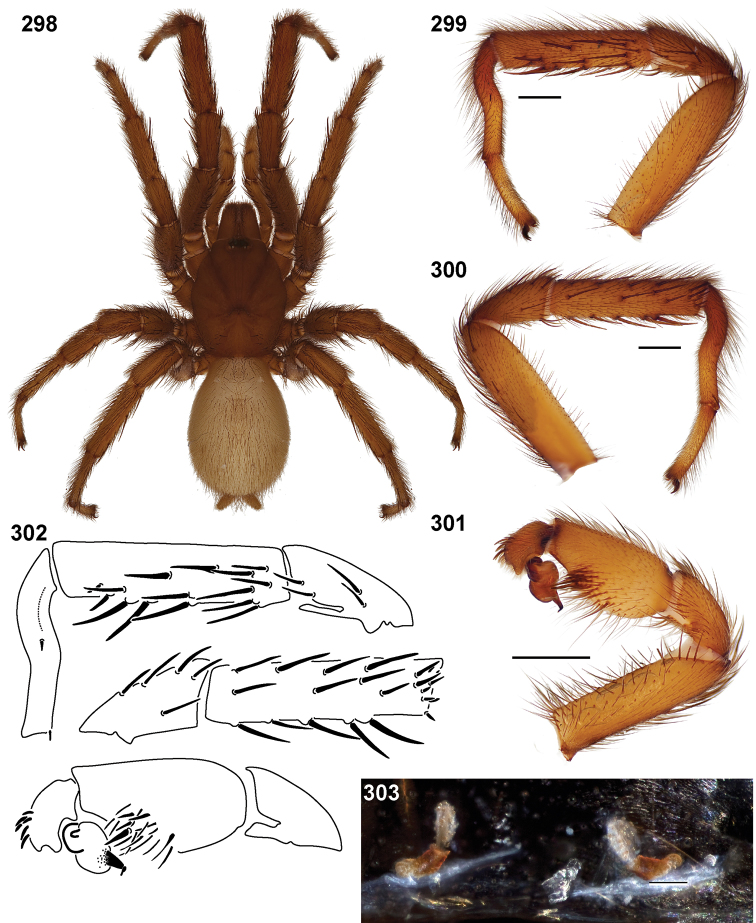
*Aptostichus fornax* sp. n. **298–302** male holotype (AP418) from Inyo County; scale bars = 1.0mm **298** habitus [806456] **299** retrolateral aspect, leg I [806460] **300** prolateral aspect, leg I [806462] **301** retrolateral aspect, pedipalp [806464] **302** line drawings of leg I spination pattern and pedipalp; retrolateral aspect patella, tibia, metatarsus; prolateral aspect patella and tibia; retrolateral aspect, pedipalp **303** female paratype (AP578), cleared spermathecae [806465]; scale bar = 0.1mm.

Figures 298–303 Map 33

#### Types.

Male holotype (AP418) from California, Inyo County, Panamint Valley sand dunes, 36.09167, -117.25917^4^, 317m, coll. D. Giuliani 17.ii.1972; female paratype (AP578) from type locality, coll. 27.ix.1974; holotype deposited in CAS, paratype in AMNH.

#### Etymology.

The specific epithet is a noun taken in apposition and refers to the hot climate in which this species is found. Fornax is a southern constellation; the name is Latin for furnace. In Roman mythology Fornax is the goddess of the hearth.

#### Diagnosis.

Males can be distinguished by a palpal tibia with a retrolateral patch of small spines and at least one large, stout spine (Fig. 303, 302) and numerous spines on the retrolateral surface of tibia II; *Aptostichus elisabethae* lacks the palpal spine and tibia II retrolateral spines. The enlarged palpal spine is shared bya number of *Sierra* species group taxa; however, these species lack the proximal - ventral metatarsus I excavation that differentially characterizes *Aptostichus fornax*. *Aptostichus fornax* specimens lack the serrated embolus of other *Simus* group species (*Aptostichus simus*, *Aptostichus lucerne*, *Aptostichus satleri*).

#### Description of male holotype.

*Specimen preparation and condition*. Specimen collected from pitfall trap, preserved in 70% EtOH. Coloration faded. Pedipalp, leg I left side removed, stored in vial with specimen. *General coloration*. Carapace, chelicerae, legs dark red 2.5YR 3/6. Abdomen uniform very pale brown 10YR 7/4, lacks distinct markings (Fig. 298). *Cephalothorax*. Carapace 4.8 long, 3.96 wide, glabrous, stout black bristles along fringe; surface smooth, pars cephalica elevated. Fringe, posterior margin with black bristles. Foveal groove deep, strongly recurved. Eyes on low mound. AER slightly procurved, PER slightly recurved. PME’s much smaller in size than AME’s. Sternum moderately setose, STRl 2.75, STRw 2.23. Posterior sternal sigilla medium in size, elongate, positioned centrally, not contiguous, anterior sigilla pairs small, sub-oval to elongate, marginal. Chelicerae with distinct anterior tooth row comprising 4 teeth, posterior margin with single row of small denticles. Palpal endites, labium, lacks cuspules, LBw 0.77, LBl 0.37. Rastellum consists of 8 stout spines arranged in continuous row. *Abdomen*. Setose, heavy black setae intermingled with fine black setae. *Legs*. Leg I: 4.50, 3.64, 2.82, 1.83, 1.50; leg IV: 4.2, 2.36. Light tarsal scopulae on legs I, II. Tarsus I with single, slightly staggered row of 12 trichobothria. Leg I spination pattern illustrated in Figures 299, 300, 302; TSp 17, TSr 12, TSrd 0. *Pedipalp*. Articles stout, palpal tibia width greater than ½ length, with one large spine situated among distinct patch of smaller spines (Figs 301, 302). Palpal bulb short, broad with slight curvature at tip, lacks serrations. PTw 0.92, PTl 1.70, Bl 0.62.

**Variation.**Known only from the type specimen.

#### Description of female paratype.

*Specimen preparation and condition*. Female presumed to have been collected live from burrow, prepared in same manner as male holotype; in very poor condition, abdomen destroyed. Genital plate in poor condition, removed, cleared in trypsin, stored in microvial with specimen. *General coloration*. Carapace, legs, chelicerae, dark reddish brown 2.5YR 2.5/3. *Cephalothorax*. Carapace 4.40 long, 3.32 wide, generally glabrous, very sparse fine black setae; generally smooth surface, pars cephalica moderately elevated. Fringe lacks setae. Foveal groove deep, slightly procurved. Eye group slightly elevated on very low mound. AER slightly procurved, PER slightly recurved. PME’s smaller in diameter than AME’s. Sternum widest at coxae II/III, moderately setose, STRl 2.45, STRw 1.88. Three pairs of sternal sigilla anterior pairs small in size, oval, marginal; posterior pair moderate in size, elongate, mesially positioned but not contiguous. Chelicerae anterior tooth row comprising 4 teeth with posterior margin denticle patch. Palpal endites with >75 cuspules concentrated at the inner (promargin) posterior heel; labium lacks cuspules, LBw 0.80, LBl 0.63. Rastellum consists of 17 stout spines not positioned on mound; fringe of short spines along distal promargin extending upward from rastellum. *Abdomen*. Moderately setose. PLS all 3 segments with spigots. Terminal segment 1/2 length of medial segment, 2 enlarged spigots visible at tip. PMS single segment, with spigots, short with rounded terminus. *Walking legs*. Anterior two pairs noticeably more slender than posterior pairs. Leg I 10.46 long. Tarsus I with 7 trichobothria arranged in staggered row. Legs I, II with light scopulae on tarsus, metatarsus; light scopulae on distal aspect tarsus legs III, IV. PTLs 23, TBs 4. Rudimentary preening comb on retrolateral distal surface, tarsus-metatarsus joint, of metatarsus IV; well developed, wide preening comb on leg III. *Spermathecae*. 2 short, heavily sclerotized, simple spermathecal bulbs; basal extension small (Fig. 303).

**Variation.**Known only from the type specimen.

#### Material examined.

Known only from the type material.

#### Distribution and natural history.

Known only from the type locality in Inyo County, characterized as Mojave Desert habitat.

#### Conservation status.

The conservation status of *Aptostichus fornax* is likely to be imperiled given its very restricted distribution in Death Valley National Park and paucity of specimens collected.

#### Species concept applied.

Morphological.

### Aptostichus lucerne sp. n.

‘Deadman’s Trapdoor Spider’

urn:lsid:zoobank.org:act:3A7C3DDF-6B7D-4EB9-B24B-A85760FB8EEE

http://species-id.net/wiki/Aptostichus_lucerne

Figures 304–310 Map 33

#### Types.

Male holotype (AP434) and paratype (AP433) from California, San Bernardino County, Deadman’s Point, locality presumed to be point E of Apple Valley, jnct. HWY’s 18 and 247, 34.47221, -117.122^3^, 922m, coll. E. Sleeper 25.x.1957; deposited in AMNH.

#### Etymology.

The specific epithet is a noun in apposition taken from the presumed type locality of Deadman’s Point in the Lucerne Valley.

#### Diagnosis.

**Figures 304–310.**
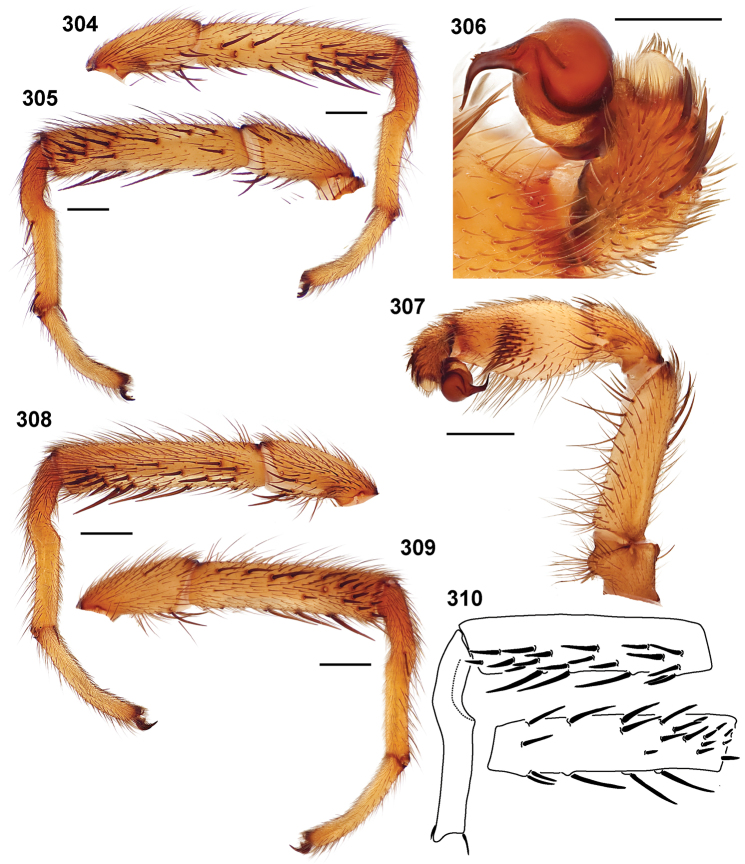
*Aptostichus lucerne* sp. n. from San Bernardino County. **304–307** male holotype (AP434); scale bars = 1.0mm and 0.5mm (**306**) **304** retrolateral aspect, of right leg, leg I [806479] **305** prolateral aspect, of right leg, leg I [806483] **306** oblique, ventral/retrolateral aspect of palpal bulb [806485] **307** retrolateral aspect, pedipalp [806487] **308, 309** male paratype **308** retrolateral aspect, leg I [806489] **309** prolateral aspect, leg I [806493] **310** line drawings of leg I spination pattern; retrolateral aspect tibia and metatarsus; prolateral aspect of tibia.

Males can be distinguished by having long ventral spines on tibia I like those of *Aptostichus elisabethae* (Figs 304, 305, 308, 309, 310). However, the tibia I prolateral spination is denser (TSp 17–19 vs. 8–16 in *Aptostichus elisabethae*) and the embolus of *Aptostichus lucerne* is serrated (Fig. 306), whereas that of *Aptostichus elisabethae* is not.

#### Description of male holotype.

*Specimen preparation and condition*. Specimen collected from pitfall trap, preserved in 70% EtOH. Coloration faded. Pedipalp, leg I left side, other legs, removed, stored in vial with specimen. *General coloration*. Carapace, chelicerae, legs yellowish red 5YR 4/6. Abdomen uniform light brown 7.5YR 6/4 dorsally, light dorsal chevron markings. *Cephalothorax*. Carapace 5.15 long, 4.40 wide, glabrous, stout black bristles along fringe; surface smooth, pars cephalica elevated. Fringe, posterior margin with black bristles. Foveal groove deep, strongly recurved. Eyes on low mound. AER slightly procurved, PER slightly recurved. PME’s smaller in diameter than AME. Sternum moderately setose, STRl 2.95, STRw 2.45. Posterior sternal sigilla medium in size, elongate, positioned posteriorly, not contiguous, anterior sigilla pairs small, elongate, marginal. Chelicerae with distinct anterior tooth row comprising 4 teeth, posterior margin with single row of small denticles. Palpal endites, labium lacks cuspules, LBw 0.77, LBl 0.43. Rastellum consists of 14 stout spines arranged across anterior margin of chelicerae. *Abdomen*. Setose, heavy black setae intermingled with fine black setae. *Legs*. Leg I: 5.44, 4.48, 3.68, 2.26, 1.55; leg IV: 4.55, 3.00. Light tarsal scopulae on legs I, II; light scopulate on metatarsus I. Tarsus I with single, slightly staggered row of 15 trichobothria. Leg I spination pattern illustrated in Figures 304, 305, 308, 309, 310; TSp 19, TSr 15, TSrd 0. *Pedipalp*. Articles stout, tibia short, width more than half length, with a distinct patch of medial/distal retrolateral spines (Fig. 307); PTw 1.04, PTl 1.84, Bl 0.82. Embolus stout dorsal - ventrally flattened with slight curvature at midpoint, and serrated distally (Fig. 306).

**Variation (2).** Cl 4.8-5.15, Cw 3.96-4.40, STRl 2.50-2.95, STRw 2.18-2.45, LBw 0.71-0.77, LBl 0.43-0.46, leg I: 4.65-5.44, 3.80-4.48, 3.01-3.68, 2.25-2.26, 1.40-1.55, leg IV: 4.52-5.20, 2.9-3.04, PTl 1.62-1.84, PTw 0.90-1.04, Bl 0.71-0.82, TSp 18-19, TSr 15-15, TSrd 0-0.

#### Description of female.

Known only from male specimens.

#### Material examined.

Known only from the type material.

#### Distribution and natural history.

*Aptostichus lucerne* is known from only the two male type specimens collected from the type locality in San Bernardino, characterized as Mojave Desert habitat.

#### Conservation status.

The conservation status of *Aptostichus lucerne* is likely to be critically imperiled or presumed extinct. The species has not been seen since the type specimens were collected in 1957. Efforts to collect *Aptostichus lucerne* over the past decade, from the putative type locality, have proved unsuccessful; the area has been highly impacted by development.

#### Species concept applied.

Morphological.

#### Remarks.

The locality label of the type specimens is somewhat dubious, labeled only as “Deadman Point, San Bernardino”, and thus requires collecting of more specimens to confirm that the Apple Valley junction determination as the type locality is correct.

### Aptostichus bonoi sp. n.

‘Bono’s Joshua Tree Trapdoor Spider’

urn:lsid:zoobank.org:act:6C112C6F-06FD-4526-BAD7-069C5B816793

http://species-id.net/wiki/Aptostichus_bonoi

**Figures 311–315.**
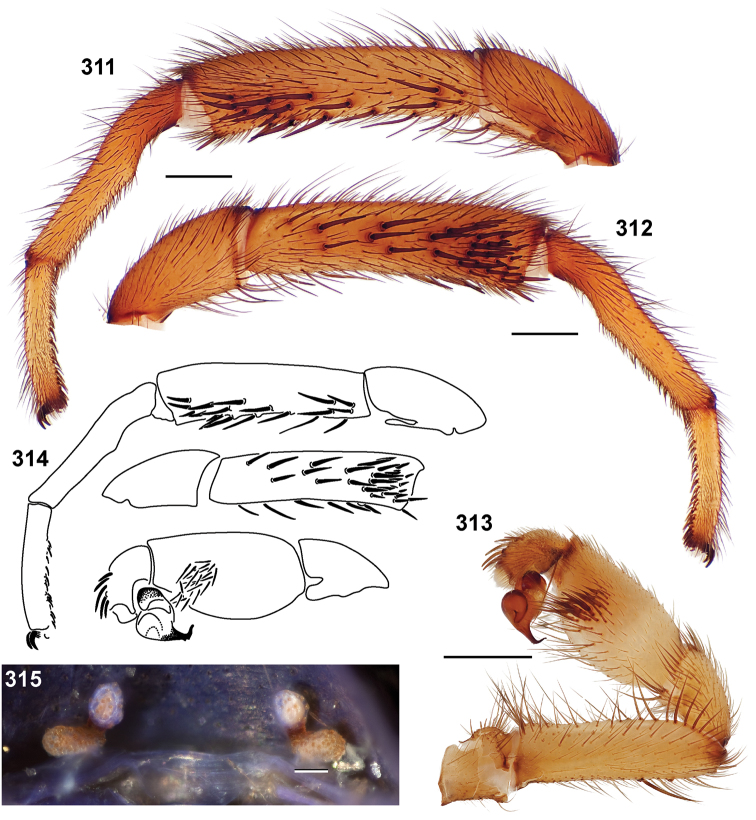
*Aptostichus bonoi* sp. n. **311–314** male holotype (AP399) from San Bernardino County; scale bars = 1.0mm **311** retrolateral aspect, leg I [806371] **312** prolateral aspect, leg I [806375] **313** retrolateral aspect, pedipalp [806377] **314** line drawings of leg I spination pattern and pedipalp; retrolateral aspect, leg I patella, tibia, metatarsus, tarsus; prolateral aspect tibia and patella; retrolateral aspect, pedipalp **315 **female paratype (AP901) from San Bernardino County, cleared spermathecae [806378]; scale bar = 0.1mm.

Figures 311–315 Map 33

#### Types.

Male holotype (AP399) from California, San Bernardino County, Joshua Tree National Park (JTNP), Lower Covington Flat, 34.0401, -116.3102^3^, 1433m, 28.ix.1962; deposited in AMNH. Female paratype (AP901) from JTNP, Upper Covington Flat, 34.0145, -116.3159^1^, coll. USGS-BRD San Diego Field Station 1.xi.2000; deposited in CAS.

#### Etymology.

The specific epithet is a patronym in honor of Bono from the Irish rock group U2 and in recognition of the Joshua Tree album released in March of 1987.

#### Diagnosis.

Males of *Aptostichus bonoi* and *Aptostichus fisheri* can be distinguished from all other species of *Aptostichus* by the presence of short, distinctive spines on the ventral surface of tarsus I (Figs 311, 312, 314) and by having only very light traces of scopulae. *Aptostichus bonoi* can be distinguished from *Aptostichus fisheri* by virtue of having many more spines on the retrolateral surface of tibia I (TSr = 14) than *Aptostichus fisheri* (TSr < 12; Figs 311, 314).

#### Description of male holotype.

*Specimen preparation and condition*. Specimen presumed to have been collected from pitfall trap, preserved in 70% EtOH. Coloration very faded. Pedipalp, leg I left side removed, other legs detached, stored in vial with specimen. *General coloration*. Carapace, chelicerae, legs yellowish red 5YR 4/6. Abdomen uniform light brown 7.5YR 6/3, dorsal chevron markings. *Cephalothorax*. Carapace 5.00 long, 4.35 wide, glabrous, stout black bristles along fringe; surface smooth, pars cephalica elevated. Fringe, posterior margin with black bristles. Foveal groove deep, strongly recurved. Eyes on low mound. AER slightly procurved, PER slightly recurved. PME, AME subequal diameter. Sternum moderately setose, STRl 2.75, STRw 2.48. Posterior sternal sigilla small, positioned away from margin but not in center, not contiguous, anterior sigilla pairs small, oval, marginal. Chelicerae with distinct anterior tooth row comprising 4 teeth, posterior margin with single row of small denticles. Palpal endites, labium, lacks cuspules, LBw 0.77, LBl 0.39. Rastellum consists of 12 stout spines not on prominent mound. *Abdomen*. Setose, heavy black setae intermingled with fine black setae. *Legs*. Leg I: 5.44, 4.00, 3.22, 1.95, 1.42; leg IV: 5.13, 2.76. Very light tarsal scopulae on legs I, II. Tarsus I with single, slightly staggered row of 11 trichobothria. Leg I spination pattern illustrated in Figures 311, 312, 314; TSp 29, TSr 14, TSrd 0; distinct short spines on ventral surface tarsus I. *Pedipalp*. Palpal tibia short, width more than half-length, with distinct patch of medial/distal retrolateral spines (Fig. 314). Palpal bulb short; embolus stout, dorsal-ventrally flattened with slight curvature at its midpoint, serrated distally (Fig. 313). PTw 0.90, PTl 1.60, Bl 0.77.

**Variation.** Known only from the type specimen.

#### Description of female paratype.

*Specimen preparation and condition*. Female presumed to have been collected live from burrow, prepared in same manner as male holotype. Genital plate removed, cleared in trypsin, stored in microvial with specimen. *General coloration*. Carapace, legs, chelicerae, dark reddish brown 2.5YR 2.5/4. Abdomen reddish brown, 5YR 4/3. *Cephalothorax*. Carapace 5.75 long, 4.80 wide, generally glabrous, very sparse fine black setae; generally smooth surface, pars cephalica moderately elevated. Fringe lacks setae. Foveal groove deep, straight. Posterior aspect carapace flat. Eye group slightly elevated on low mound. AER slightly procurved, PER slightly recurved. PME, AME subequal diameter. Sternum widest at coxae II/III, moderately setose, STRl 3.22, STRw 3.21. Three pairs of sternal sigilla anterior pairs small in size, oval, marginal; posterior pair moderate in size, elongate, mesially positioned but not contiguous. Chelicerae anterior tooth row comprising 4 teeth with posterior margin denticle patch. Palpal endites with 24 cuspules concentrated at the inner (promargin) posterior heel; labium lacks cuspules, LBw 1.02, LBl 0.60. Rastellum consists of 12 stout spines not positioned on mound; fringe of short spines along distal promargin extending upward from rastellum. *Abdomen*. Moderately setose. PLS all 3 segments with spigots. Terminal segment 1/2 length of medial segment, 2 enlarged spigots visible at tip. PMS single segment, with spigots, short with rounded terminus. *Legs*. Anterior two pairs noticeably more slender than posterior pairs. Leg I 12.55 long. Tarsus I with 7 trichobothria arranged in staggered row, distal aspect of row interspersed with setae. Legs I, II with light scopulae on tarsus, metatarsus; light scopulae on distal aspect tarsus legs III, IV. PTLs 30, TBs 7. Rudimentary preening comb on retrolateral distal surface, tarsus-metatarsus joint, of metatarsus IV; well developed, wide preening comb on leg III. *Spermathecae*. 2 short, heavily sclerotized, simple spermathecal bulbs; basal extension small (Fig. 315).

**Variation.**Known only from the type specimen.

#### Material examined.

**United States: California: Riverside Co.:** Joshua Tree Natl Park, Covington Flat, 34.0311, -116.3177^1^, 1554m, J Bond 8.xii.1997 [AP682, 1juv, AUMNH]; Joshua Tree Natl Park, Upper Covington Flat, 34.0145, -116.3159^1^, 949m, 1.xi.2000 USGS-BRD San Diego Sta. [AP901, 1♀, CAS]; **San Bernardino Co.:** Joshua Tree Natl Park, Lower Covington Flat, 34.0401, -116.3102^3^, 1433m, 28.ix.1962 [AP399, 1♂, AMNH].

#### Distribution and natural history.

Known only from the type locality in the Covington Flat area of Joshua Tree National Park (Map 33). The habitat is higher altitude Mojave Desert and is considerably more vegetated than lower altitude areas. Based on the limited data available, males disperse during late fall through early winter (September–November).

#### Species concept applied.

Morphological.

#### Conservation status.

The conservation status of *Aptostichus bonoi* is likely imperiled given its very restricted distribution and rarity in collections.

### Aptostichus fisheri sp. n.

‘Fisher’s Red Rock Trapdoor Spider’

urn:lsid:zoobank.org:act:A2224401-F628-4E54-B81B-80B95FB988C4

http://species-id.net/wiki/Aptostichus_fisheri

Figures 316–322 Maps 33

#### Types.

Male holotype (AP955) from California, Kern County, north Red Rock Canyon, 35.39752, -117.99797^1^, 865m, coll. 30.ix-3.x.2003 and male paratype (AP954) from east Dove Spring Canyon, 35.43813, -118.04607^1^, 1068m, coll. USGS-BRD San Diego Field Station 1.x.2003; deposited in CAS. Male paratype (AP407) from NE edge of El Paso Mountains, hills ~1.6km W HWY 395.

#### Etymology.

The specific epithet is a patronym in honor of Robert N. Fisher of the United States Geological Survey San Diego Field Station. Specimens collected through projects directed by Dr. Fisher have greatly enhanced our knowledge of *Aptostichus* species distributions.

#### Diagnosis.

Males of *Aptostichus fisheri* are distinguished in the diagnosis of *Aptostichus bonoi*.

#### Description of male holotype.

**Figures 316–322.**
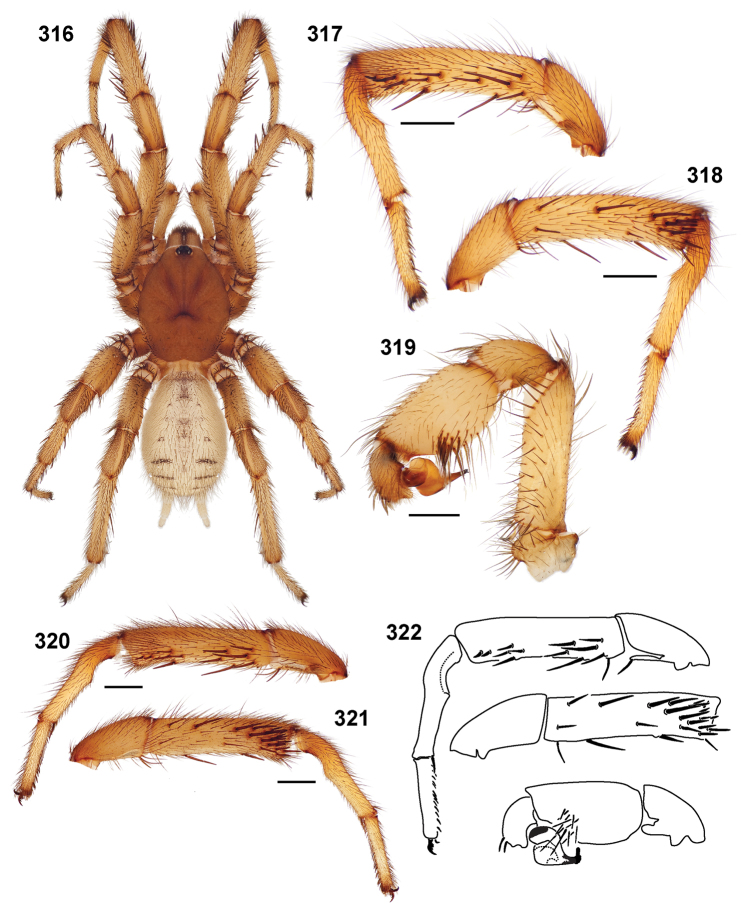
*Aptostichus fisheri* sp. n. from Kern County; scale bars = 1.0mm. **316** habitus of male paratype (AP954) [806438] **317–319** male holotype (AP955) **317** retrolateral aspect, leg I [806442] **318** prolateral aspect, leg I [806446] **319** retrolateral aspect, pedipalp [806448] **320, 321** male paratype (AP407) **320** retrolateral aspect, leg I [806450] **321** prolateral aspect, leg I [806454] **322** line drawings of leg I spination pattern and pedipalp; retrolateral aspect, patella, tibia, metatarsus, tarsus; prolateral aspect, tibia and patella; retrolateral aspect, pedipalp.

*Specimen preparation and condition*. Specimen collected from pitfall trap, preserved in 80% EtOH. Coloration lightly faded. Pedipalp, leg I left side removed, stored in vial with specimen; leg II right side missing from specimen. *General coloration*. Carapace, chelicerae, legs dark reddish brown 5YR 3/4. Abdomen uniform light brown 7.5YR 6/3, light dorsal chevron markings (Fig. 316). *Cephalothorax*. Carapace 3.76 long, 3.08 wide, glabrous, stout black bristles along fringe; surface smooth, pars cephalica elevated. Fringe, posterior margin with black bristles. Foveal groove deep, strongly recurved. Eyes on low mound. AER slightly procurved, PER slightly recurved. PME, AME subequal diameter. Sternum moderately setose, STRl 2.04, STRw 1.88. Posterior sternal sigilla small, positioned away from margin but not in center, not contiguous, anterior sigilla pairs small, oval, marginal. Chelicerae with distinct anterior tooth row comprising 4 teeth, posterior margin with single row of small denticles. Palpal endites, labium, lacks cuspules, LBw 0.60, LBl 0.29. Rastellum consists of 12 stout spines not on prominent mound. *Abdomen*. Setose, heavy black setae intermingled with fine black setae. *Legs*. Leg I: 3.92, 3.44, 2.55, 1.71, 1.25; leg IV: 3.68, 2.17. Very light tarsal scopulae on legs I, II. Tarsus I with single, slightly staggered row of 9 trichobothria. Leg I spination pattern illustrated in Figures 317, 318, 320-322; TSp 13, TSr 10, TSrd 0; distinct short spines on ventral surface tarsus I. *Pedipalp*. Palpal tibia short, width more than half-length, with distinct patch of medial/distal retrolateral spines (Fig. 319). Palpal bulb short; embolus stout, dorsal-ventrally flattened with slight curvature at its midpoint, serrated distally (Fig. 322). PTw 0.77, PTl 1.30, Bl 0.65.

**Variation (3).** Cl 3.72-4.12, 3.87±0.13; Cw 3.01-3.48, 3.19±0.15; STRl 2.00-2.18, 2.07±0.05; STRw 1.84-1.98, 1.90±0.04; LBw 0.56-0.68, 0.61±0.04; LBl 0.29-0.37, 0.32±0.03; leg I: 3.92-4.35, 4.06±0.14; 3.29-3.60, 3.44±0.09; 2.35-2.63, 2.51±0.08; 1.64-1.75, 1.70±0.03; 1.20-1.25, 1.23±0.02; leg IV: 3.56-4.08, 3.77±0.16; 2.17-2.36, 2.23±0.06; PTl 1.30-1.36, 1.32±0.02; PTw 0.71-0.77, 0.75±0.02; Bl 0.58-0.68, 0.64±0.03; TSp 13-21, 16.00±2.52; TSr 10-11, 10.33±0.33; TSrd 0-0, 0±0.

#### Description of female.

Known only from male specimens.

#### Material examined.

Known only from the type material.

#### Distribution and natural history.

*Aptostichus fisheri* is narrowly restricted to high altitude Mojave Desert habitat in Kern County. Males appear to disperse during the fall months of September and October.

#### Species concept applied.

Morphological.

#### Conservation status.

The conservation status of *Aptostichus fisheri* is likely imperiled given its very restricted distribution.

### Aptostichus cajalco sp. n.

‘The Cajalco Canyon Trapdoor Spider’

urn:lsid:zoobank.org:act:F5B5607A-CB36-41F9-99EB-8B0795ECF0BC

http://species-id.net/wiki/Aptostichus_cajalco

Figures 323 –331 Map 33

#### Types.

Male holotype and female paratype (MY3778) from California, Riverside County, 2.9km along Cajalco Road, west of Lake Matthews, 33.8256, -117.4957^1^, 265m, coll. 6.ix.1999 W. Icenogle; additional male paratypes (AP1239, 1236) from the type locality coll. W. Icenogle 9.ix.1999, 6.x.1999; deposited in AUMNH.

#### Etymology.

The specific epithet is a noun in apposition taken from the type locality, Cajalco Canyon.

#### Diagnosis.

Male and female *Aptostichus cajalco* can be diagnosed from all other *Simus* group species by having a round sternum that is approximately as wide as it is long; all other species have a sternum that is more typical (widest at coxae III-IV junction).

#### Description of male holotype.

**Figures 323–327.**
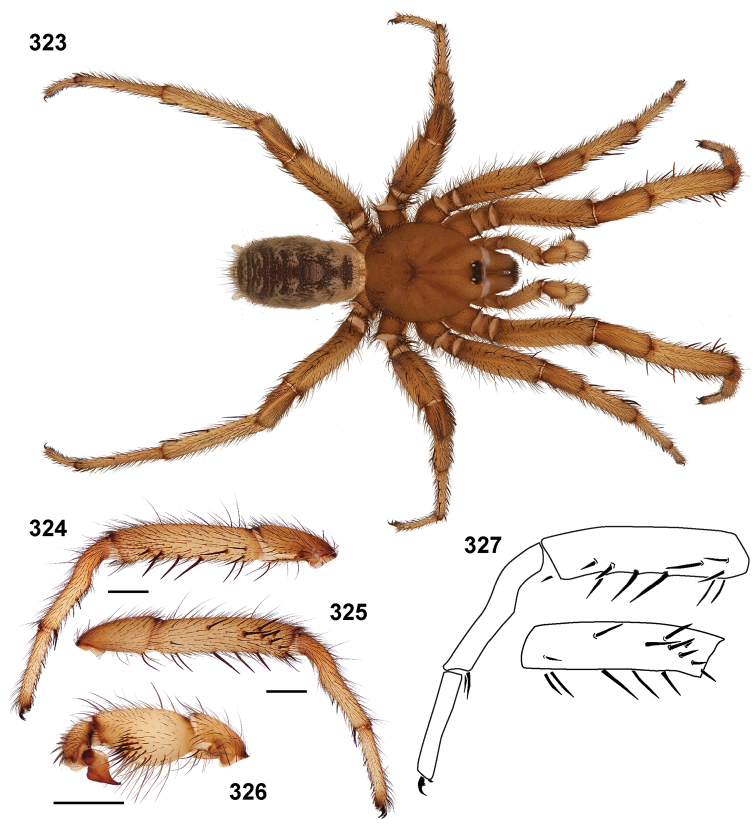
*Aptostichus cajalco* sp. n. male holotype (MY3778) from Riverside County; scale bars = 1.0mm. **323** habitus [806393] **324** retrolateral aspect, leg I [806395] **325** prolateral aspect, leg I [806397] **326** retrolateral aspect, pedipalp [806399] **327** line drawings of leg I spination pattern; retrolateral aspect, tibia, metatarsus, tarsus; tibia.

*Specimen preparation and condition*. Specimen collected from pitfall trap, preserved in 80% EtOH. Coloration in good condition. Pedipalp, leg I left side removed, stored in vial with specimen. *General coloration*. Carapace, chelicerae, legs yellowish red 5YR 4/6. Abdomen uniform brown 7.5YR 4/3, distinct dorsal mottled chevron markings (Fig. 323). *Cephalothorax*. Carapace 3.66 long, 3.04 wide, glabrous, stout black bristles along fringe; surface smooth, pars cephalica elevated. Fringe, posterior margin with black bristles. Foveal groove deep, strongly recurved. Eyes on low mound. AER slightly procurved, PER slightly recurved. PME, AME subequal diameter. Sternum moderately setose, appears round in shape, STRl 2.02, STRw 1.82. Posterior sternal sigilla small, positioned away from margin but not in center, not contiguous, anterior sigilla pairs small, oval, marginal. Chelicerae with distinct anterior tooth row comprising 4 teeth, posterior margin with single row of small denticles. Palpal endites, labium, lacks cuspules, LBw 0.60, LBl 0.29. Rastellum consists of ~8 stout spines not on prominent mound. *Abdomen*. Setose, heavy black setae intermingled with fine black setae. *Legs*. Leg I: 3.88, 3.20, 2.39, 1.68, 1.20; leg IV: 3.68, 2.20. Very light to no tarsal scopulae on legs I, II. Tarsus I with single, slightly staggered row of 11 trichobothria. Leg I spination pattern illustrated in Figures 324, 325, 327; TSp 8, TSr 5, TSrd 0; distinct short spines on ventral surface tarsus I. *Pedipalp*. Palpal tibia short, width more than half-length, with distinct patch of medial/distal retrolateral spines (Fig. 326). Palpal bulb short; embolus stout, dorsal-ventrally flattened with slight curvature at its midpoint, serrated distally. PTw 0.68, PTl 1.34, Bl 0.60.

**Variation (5).** Cl 3.63-4.08, 3.87±0.09; Cw 3.01-3.48, 3.20±0.1; STRl 1.98-2.25, 2.14±0.06; STRw 1.74-2.03, 1.91±0.06; LBw 0.60-0.68, 0.62±0.02; LBl 0.34-0.39, 0.36±0.01; leg I: 3.72-4.10, 3.87±0.06; 3.08-3.26, 3.15±0.03; 2.26-2.48, 2.36±0.04; 1.56-1.70, 1.63±0.03; 1.10-1.20, 1.15±0.02; leg IV: 3.57-4, 3.75±0.09; 2.08-2.29, 2.18±0.03; PTl 1.28-1.39, 1.31±0.02; PTw 0.66-0.75, 0.71±0.02; Bl 0.60-0.71, 0.66±0.02; TSp 8-11, 9.80±0.73; TSr 3-7, 5.20±0.8; TSrd 0-0, 0±0.

#### Description of female paratype.

**Figures 328–331.**
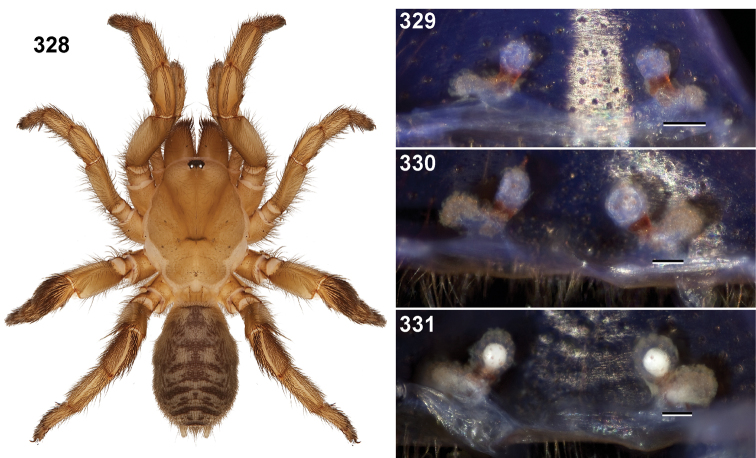
*Aptostichus cajalco* sp. n. female specimens from Riverside County. **328** habitus, female paratype (MY3778) [806382] **329–331** cleared spermathecae; scale bars = 0.1mm **329** MY3778 [806385] **330** AP668 [806386] **331** AP669 [806389].

*Specimen preparation and condition*. Female collected from pitfall trap, prepared in same manner as male holotype. Genital plate, removed, cleared in trypsin, stored in microvial with specimen. *General coloration*. Carapace, legs, chelicerae, strong brown, 7.5YR 4/6. Abdomen brown, 7.5YR 5/3 with distinct mottled striping (Fig. 328). *Cephalothorax*. Carapace 4.00 long, 3.44 wide, generally glabrous, very sparse fine black setae; generally smooth surface, pars cephalica moderately elevated. Fringe lacks setae. Foveal groove deep, straight. Eye group slightly elevated on low mound. AER slightly procurved, PER slightly recurved. PME-AME subequal diameter. Sternum round in appearance, moderately setose, STRl 2.10, STRw 1.93. Three pairs of sternal sigilla anterior pairs small in size, oval, marginal; posterior pair moderate in size, elongate, mesially positioned but not contiguous. Chelicerae anterior tooth row comprising 4 teeth with posterior margin denticle patch. Palpal endites with 24 cuspules concentrated at inner (promargin) posterior heel; labium lacks cuspules, LBw 0.68, LBl 0.43. Rastellum consists of 12 stout spines not positioned on mound; fringe of short spines along distal promargin extending upward from rastellum. *Abdomen*. Moderately setose. PLS all 3 segments with spigots. Terminal segment 1/2 length of medial segment, 2 enlarged spigots visible at tip. PMS single segment, with spigots, short with rounded terminus. *Legs*. Anterior two pairs noticeably more slender than posterior pairs. Leg I 8.15 long. Tarsus I with 7 trichobothria arranged in staggered row, distal aspect of row interspersed with setae. Legs I, II with light scopulae on tarsus, metatarsus; light scopulae on distal aspect tarsus legs III, IV. PTLs 27, TBs 7. Rudimentary preening comb on retrolateral distal surface, tarsus-metatarsus joint, of metatarsus III; well developed, wide preening comb on leg IV. *Spermathecae*. 2 short, heavily sclerotized, simple spermathecal bulbs; basal extension small (Figs 329–331).

**Variation (4).** Cl 4.00-6.25, 5.25±0.49; Cw 3.44-5.50, 4.51±0.43; STRl 2.10-3.36, 2.74±0.27; STRw 1.93-3.32, 2.67±0.31; LBw 0.68-1.02, 0.9±0.08; LBl 0.43-0.77, 0.61±0.08; Leg I: 8.15-13.68, 11.10±1.15; ANTd 4-4, 4.00±0; PTLs 27-36, 30.00±2.12; TBs 6-8, 7.00±0.41.

#### Material examined.

**United States: California: Riverside Co.:** 2.9km [road] W of Lake Mathews, Canyon just S Cajalco Rd, 33.8256, -117.4957^1^, 265m, W Icenogle 22.iv.2000 [AP360, 2♀, AUMNH], 6.viii.2000 [AP367, 368, 666, 2♂, 1♀, AUMNH], 23.vii.2000 [AP667, 2♀, AUMNH], 7.vii.2000 [AP668, 1♀, AUMNH], 8.vi.2000 [AP669, 1♀, 2juv, AUMNH], 18.viii.2000 [AP670, 2♂, AUMNH], 6.ix.1999 [AP1235-1239, 12♂, AUMNH], 19.vii.1999 [AP1240, 1♀, 2juv, AUMNH], 24.ix.2002 [MY670-673, 4♂, AUMNH], 6.ix.1999 [MY3778, 1♀, 1♂, AUMNH], J Bond 20.xi.1998 [AP694, 1♀, AUMNH].

#### Distribution and natural history.

*Aptostichus cajalco* is known only from the type locality, adjacent to “Cajalco Canyon” in Riverside County. The habitat is California coastal chaparral. Males disperse in late summer and early fall (August, September) before the winter rains. A single, highly degraded specimen resembling *Aptostichus cajalco* was collected in a pitfall trap from the vicinity of Yucaipa, California.

#### Species concept applied.

Morphological.

#### Conservation status.

As a consequence of its very restricted distribution and apparently low abundance the conservation status of *Aptostichus cajalco* would likely be chara-cterized as imperiled.

##### The *Sierra* species group

**Included species.**

*Aptostichus sierra* sp. n.

*Aptostichus huntington* sp. n.

*Aptostichus dorothealangeae* sp. n.

*Aptostichus chavezi* sp. n.

### Aptostichus sierra sp. n.

‘The Shaver Lake Trapdoor Spider’

urn:lsid:zoobank.org:act:AB21A3CF-506D-420D-A2EC-D09E4DEA445E

http://species-id.net/wiki/Aptostichus_sierra

**Figures 332–336.**
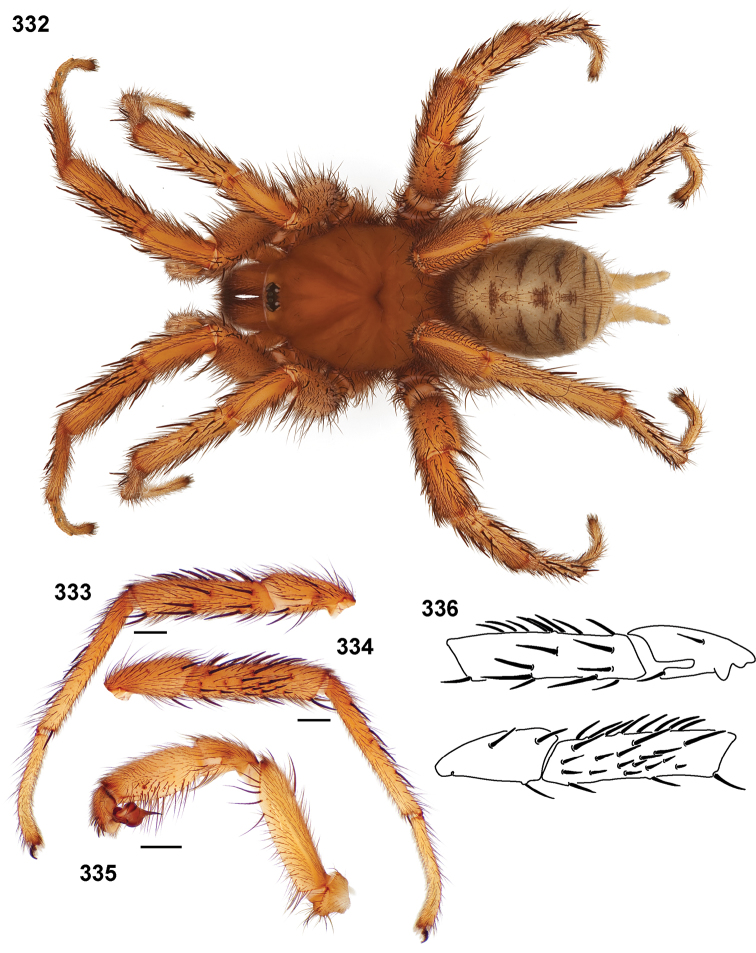
*Aptostichus sierra* sp. n. male holotype (AP400) from Fresno County; scale bars = 1.0mm. **332** habitus [806511] **333** retrolateral aspect, leg I [806515] **334** prolateral aspect, leg I [806517] **335** retrolateral aspect, pedipalp [806519] **336** line drawings of leg I tibia and patella spination patterns; retrolateral aspect; prolateral aspect.

**Map 34, 35.**
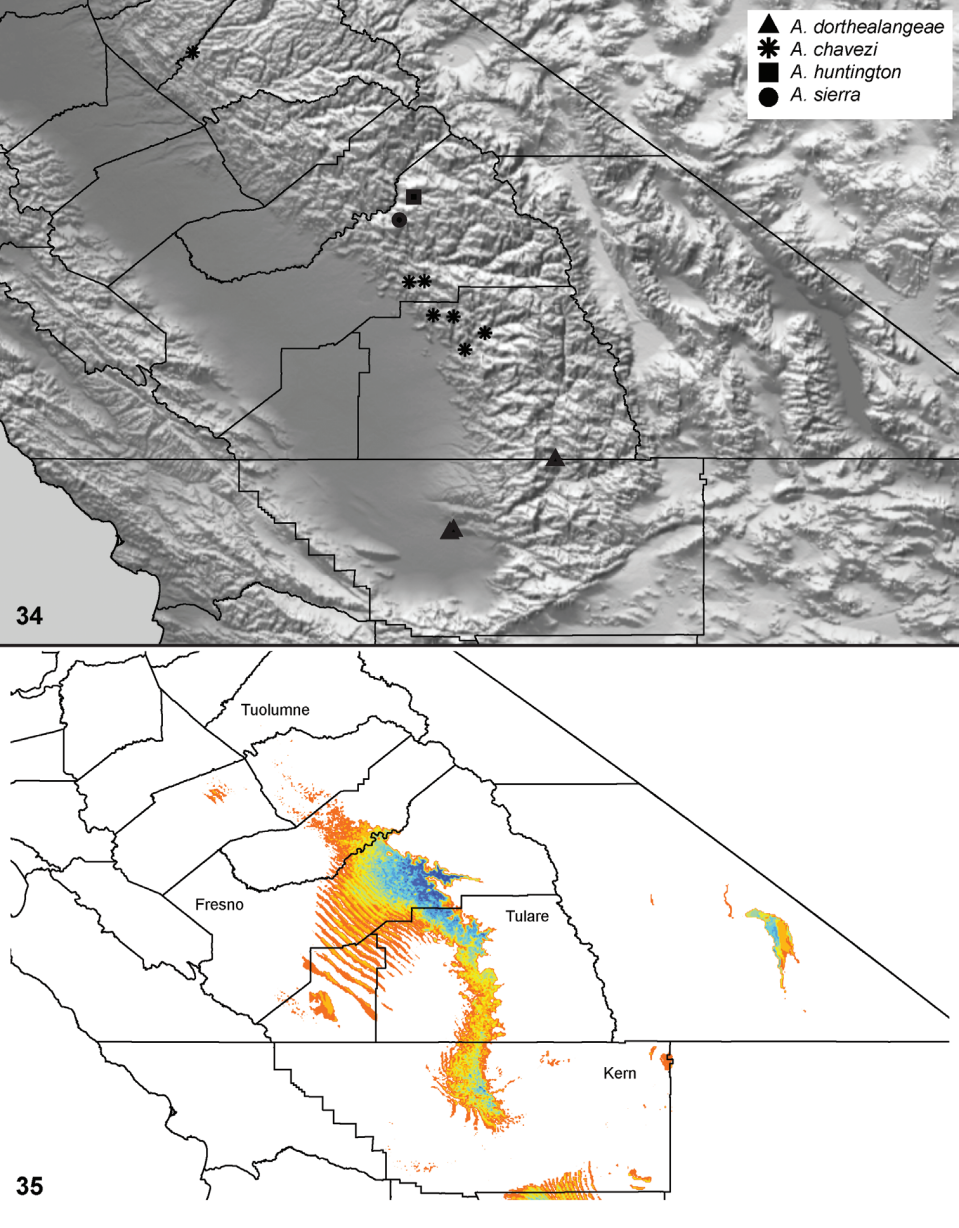
Distribution of *Sierra* group species. **34 k**nown distribution of *Aptostichus dorothealangeae*, *Aptostichus chavezi*, *Aptostichus huntington*, and *Aptostichus sierra* **35** predicted distribution of *Aptostichus chavezi*; cooler colors–blue shades–represent areas of high probability of occurrence, warmer colors–yellow and orange shades–represent areas of low probability of occurrence.

Figures 332–336 Map 34

#### Types.

Male holotype (AP400) from California, Fresno County, Shaver Lake, 37.1129, -119.3095^5^, 1660m, coll. 12.ix.1959; deposited in AMNH.

#### Etymology.

The specific epithet is a noun taken in apposition from the location of the type locality in the Sierra Nevada mountain range.

#### Diagnosis.

All known male specimens of the *Sierra* species group can be distinguished from all other species of *Aptostichus* by virtue of having a long slender metatarsus I which lacks a ventral/proximal excavated area. *Aptostichus sierra* males can be distinguished from others in the *Sierra* species group by virtue of their large size, by having a broader sternum, and unique leg I spination pattern (Figs 333, 334, 336).

#### Description of male holotype.

*Specimen preparation and condition*. Specimen presumed to have been collected from pitfall trap, preserved in 70% EtOH. Coloration faded. Pedipalp, leg I left side removed, stored in vial with specimen. Large megaspine on tibia pedipalp left side missing, intact on right side pedipalp tibia. *General coloration*. Carapace, chelicerae, legs yellowish red 5YR 4/6. Abdomen uniform brown 7.5YR 5/3 dorsally, mottled dorsal chevron pattern (Fig. 332). *Cephalothorax*. Carapace 5.52 long, 4.50 wide, glabrous with few fine white spines, fine black spines intermingled, stout black bristles along fringe; surface smooth, pars cephalica elevated. Fringe, posterior margin with black bristles. Foveal groove deep, moderately recurved. Eyes on low mound. AER slightly procurved, PER slightly recurved. PME, AME subequal diameter. Sternum moderately setose, STRl 3.07, STRw 2.25. Posterior sternal sigilla small, positioned marginally, not contiguous, anterior sigilla pairs very small, oval, marginal. Chelicerae with distinct anterior tooth row comprising 6 teeth, posterior margin with single row of small denticles. Palpal endites with patch of small cuspules on proximal, inner margin, labium lacks cuspules, LBw 0.31, LBl 0.77. Rastellum consists of 6 stout spines arranged along anterior margin, not on prominent mound. *Abdomen*. Setose, heavy black setae intermingled with fine black setae. *Legs*. Leg I: 4.90, 3.36, 3.92, 2.68, 0.00; leg IV: 5.20, 2.75. Tarsi strongly bent. Light tarsal scopulae on all legs I, II; metatarsus I with light scopulate distally. Tarsus I with single, slightly staggered row of 17 trichobothria. Leg I spination pattern illustrated in Figures 333, 334, 336; TSp 18 with numerous additional spines dorsally, TSr 5, TSrd 0. Tibia I long relative to femur I; distal retrolateral spines absent. Metatarsus not anteverted, lacks a distinct mid–ventral mating apophysis, proximal excavation of metatarsus I. Metatarsus, tarsus I long, tarsus I lacks spines (Figs 333, 334). *Pedipalp*. Palpal tibia short, width slightly less than half-length. Retrolateral surface has at least one large spine, with numerous smaller, ventral elongate spines. Palpal bulb very short (Bl/Cl 13.95), pyriform. Embolus intermediate width with slight curvature at midpoint, not serrated distally (Fig. 335). PTw 0.82, PTl 1.97, Bl 0.77.

**Variation.** Known only from the type specimen.

#### Description of female.

Known only from male specimens.

#### Material examined.

Known only from the type specimen.

#### Distribution and natural history.

*Aptostichus sierra* is known only from the type specimen from Fresno County, collected in a pitfall trap in September. The habitat type is characterized as Sierran Steppe, Mixed Coniferous Forest, and Alpine Meadow.

#### Conservation status.

The conservation status of *Aptostichus sierra* is likely to be characterized as imperiled due to its rarity and restricted distribution.

#### Species concept applied.

Morphological.

#### Remarks.

Despite extensive searching in the areas around the type locality over the past decade, I have been unable to recover a single *Aptostichus sierra* specimen. This species is either very rare or I have been unable to pinpoint its exact microhabitat.

### Aptostichus huntington sp. n.

‘The Huntington Lake Trapdoor Spider’

urn:lsid:zoobank.org:act:5F38BA70-A753-4C23-AC85-59A5144D125B

http://species-id.net/wiki/Aptostichus_huntington

**Figures 337–341.**
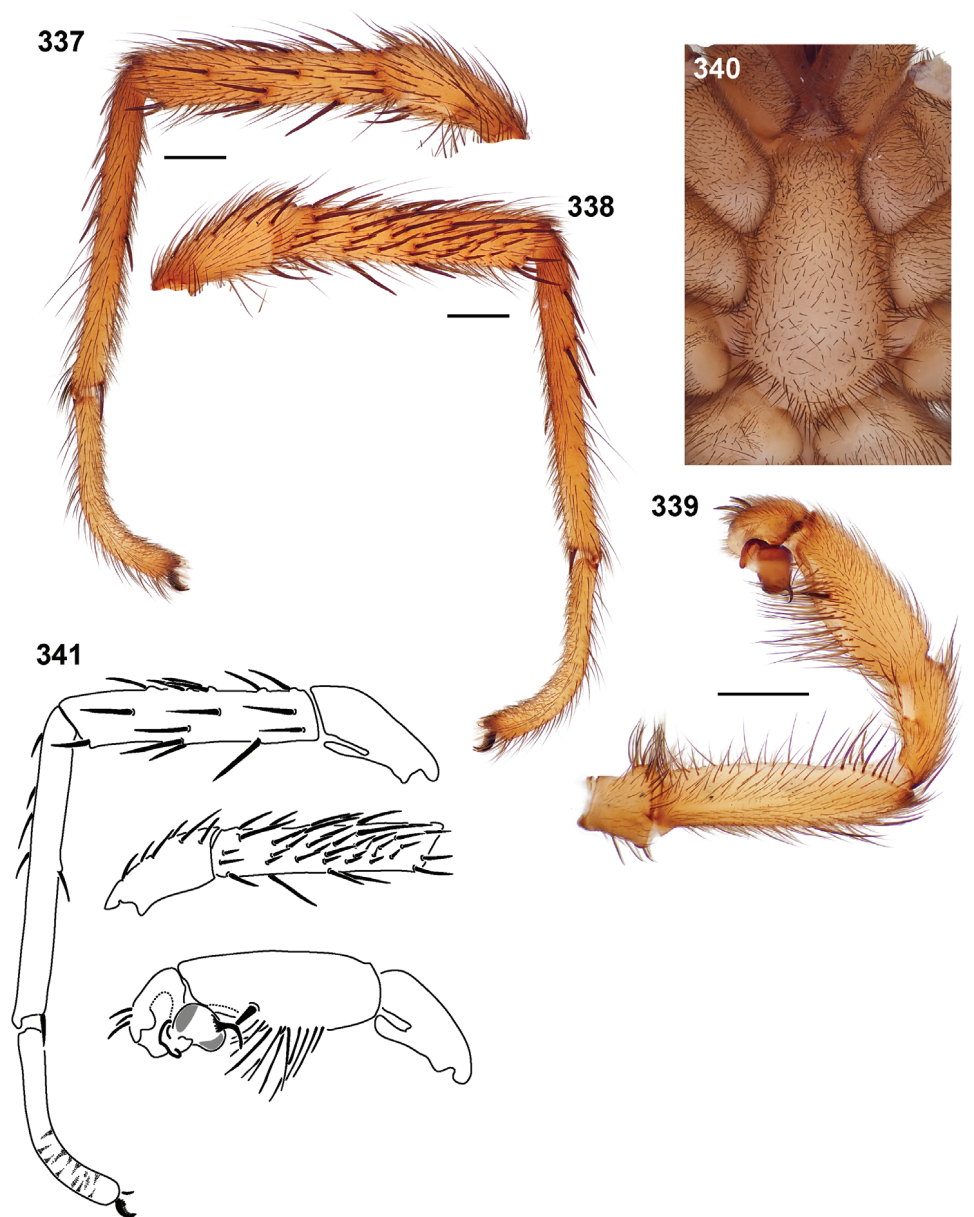
*Aptostichus huntington* sp. n. male holotype (AP408) from Fresno County; scale bars = 1.0mm. **337** retrolateral aspect, leg I [806469] **338** prolateral aspect, leg I [806473] **339** retrolateral aspect, pedipalp [806475] **340** sternum [806477] **341** line drawings of leg I and pedipalp spination patterns; retrolateral aspect patella, tibia, metatarsus, and tarsus; prolateral aspect tibia and patella; retrolateral aspect, pedipalp.

Figures 337–341 Map 34

#### Types.

Male holotype (AP408) and two male paratypes (AP408) from California, Fresno County, Billy Creek at Huntington Lake, 37.2379, -119.2295^4^, 2130m, coll. J. Halstead 21–28.viii.1984; deposited in CAS.

#### Etymology.

The specific epithet is a noun taken in apposition from the type locality of Huntington Lake.

#### Diagnosis.

Male of this species can be distinguished from others in the *Sierra* species group by their elongate sternum, strongly curved tarsus and unique tibia I prolateral spination pattern comprising > 31 spines.

#### Description of male holotype.

*Specimen preparation and condition*. Specimen presumed to have been collected from pitfall trap, preserved in 70%EtOH. Coloration faded; abdomen extremely faded, collapsed. Pedipalp, leg I left/right side removed, stored in vial with specimen. *General coloration*. Carapace, chelicerae, legs strong brown 7.5YR 4/6. Abdomen uniform light brown 7.5YR 6/3, with mottled chevron striping. *Cephalothorax*. Carapace 5.31 long, 4.00 wide, generally glabrous with very light white setae intermingled with few thin black setae, stout black bristles along fringe; surface smooth, pars cephalica elevated. Fringe, posterior margin with black bristles. Foveal groove deep, strongly recurved. Eyes on low mound. AER slightly procurved, PER slightly recurved. PME, AME subequal diameter. Sternum moderately setose, STRl 3.53, STRw 1.88; very thin. Posterior sternal sigilla small, positioned marginally, not contiguous, anterior sigilla pairs very small, oval, marginal. Chelicerae with distinct anterior tooth row comprising 5 teeth, posterior margin with single row of small denticles. Palpal endites with patch of small cuspules on proximal, inner margin, labium lacks cuspules, LBw 0.77, LBl 0.19. Rastellum consists of 8 stout spines not on prominent mound. *Abdomen*. Setose, heavy black setae intermingled with fine black setae. *Legs*. Leg I: 4.80, 3.48, 4.24, 2.56, 0.00; leg IV: 5.05, 2.90. Tarsi strongly bent. Light tarsal scopulae on all legs I, II; metatarsus I with light scopula distally. Tarsus I with single, slightly staggered row of 12 trichobothria. Leg I spination pattern illustrated in Figures 337, 338, 341; TSp 32, TSr 7, TSrd 1. Tibia I long relative to femur I, distal retrolateral spine group absent (Fig. 337). Metatarsus not anteverted, lacks distinct mid–ventral mating apophysis, proximal excavation (Figs 337, 338, 341). Metatarsus, tarsus I long. Strongly bent tarsus I lightly pseudosegmented. Tarsus I lacks ventral spines. *Pedipalp*. Palpal tibia short, width slightly less than half length (Figs 339, 341). Retrolateral surface tibia with large spine, numerous smaller, elongate ventral spines (Figs 339, 341). Palpal bulb very short (Bl/Cl 14.50). Embolus intermediate width, very thin distally, slight curvature at midpoint, not serrated distally. PTw 0.77, PTl 1.95, Bl 0.77.

**Variation (3).** Cl 5.31-5.75, 5.58±0.14; Cw 4.00-4.44, 4.27±0.14; STRl 3.51-3.81, 3.62±0.10; STRw 1.88-2.28, 2.08±0.12; LBw 0.74-0.77, 0.75±0.01; LBl 0.19-0.27, 0.23±0.02; leg I: 4.80-5.06, 4.93±0.08; 3.38-3.48, 3.43±0.03; 4.00-4.31, 4.18±0.09; 2.56-3.00, 2.77±0.13; 0-0, 0±0; leg IV: 5.05-5.44, 5.21±0.12; 2.90-3.44, 3.11±0.17; PTl 1.95-2.04, 1.98±0.03; PTw 0.74-0.78, 0.76±0.01; Bl 0.77-0.81, 0.79±0.01; TSp 32-39, 34.33±2.33; TSr 7-9, 7.67±0.67; TSrd 1-1, 1±0.

#### Description of female.

Known only from male specimens.

#### Material examined.

Known only from the type material.

#### Distribution and natural history.

*Aptostichus huntington* is known only from the type specimen from Fresno County, collected in a pitfall trap in September. The habitat type is characterized as Sierran Steppe, Mixed Coniferous Forest, and Alpine Meadow.

#### Conservation status.

The conservation status of *Aptostichus huntington* is likely to be characterized as imperiled due to its rarity and restricted distribution.

#### Species concept applied.

Morphological.

#### Remarks.

Like *Aptostichus sierra*, despite extensive searching in the areas around the type locality over the past decade, I have been unable to recover a single *Aptostichus huntington* specimen. This species is either very rare or I have been unable to pinpoint its exact microhabitat.

### Aptostichus dorothealangeae sp. n.

‘Dorothea Lange’s Trapdoor Spider’

urn:lsid:zoobank.org:act:215224F7-9FEC-4E7F-AA46-AD6684103F2C

http://species-id.net/wiki/Aptostichus_dorothealangeae

**Figures 342–346.**
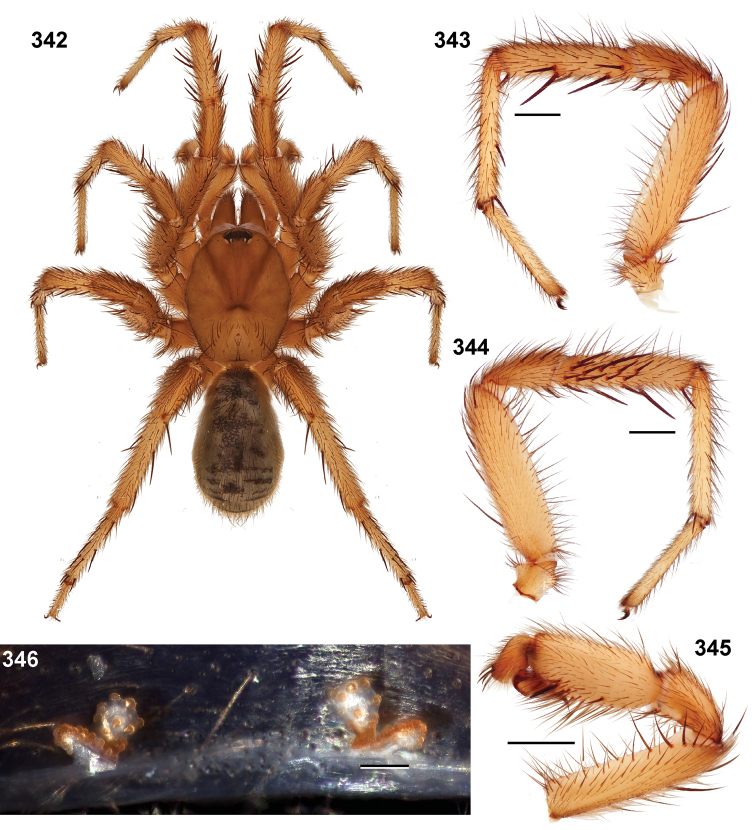
*Aptostichus dorothealangeae* sp. n. from Kern County. **342–345** male holotype (AP426); scale bars = 1.0mm **342** habitus [806412] **343** retrolateral aspect, leg I [806416] **344** prolateral aspect, leg I [806418] **345** retrolateral aspect, pedipalp [806420] **346** cleared spermathecae (AP732) [806421]; scale bar = 0.1mm.

Figures 342–346 Maps 34

#### Types.

Male holotype (AP426) and female paratype (AP460) from California, Kern Co., Bakersfield, South Bank of Kern River, 35.3947, -119.0313^5^, elev. 137m, coll. W. Icenogle 6.x.1971, 16.x.1971, deposited in AUMNH.

#### Etymology.

The specific epithet is a patronym in honor of the American photographer and photojournalist Dorothea Lange (1895–1965); best known for her photo documentation work for the Farm Security Administration during the American Great Depression.

#### Diagnosis.

*Aptostichus dorothealangeae* can be distinguished from all other species by having the characteristics described for all *Sierra* group species in the diagnosis of *Aptostichus sierra* and lacking a strongly curved tarsus I and by having a TSp spination pattern that is offset proximally (Fig. 344). Females can be recognized by having an elongate sternum and a spermathecal bulb arrangement very similar to that of *Aptostichus simus*; that is a short median stalk and a very short lateral base that does not form a secondary bulb. It is important to note that this feature may likewise distinguish *Aptostichus huntington* and *Aptostichus sierra* from other non *Sierra* group species, however females of the other two species are at present unknown. The disparity in size between *Aptostichus dorothealangeae* and the Sierran species (*Aptostichus dorothealangeae* is smaller; Cl < 4.50) might also be reflected in females of these two species and thus may prove a potentially useful distinguishing feature.

#### Description of male holotype.

*Specimen preparation and condition*. Specimen collected live from burrow, preserved in 70%EtOH. Coloration slightly faded. Pedipalp, leg I left side removed, stored in vial with specimen. *General coloration*. Carapace, chelicerae, legs strong brown 7.5YR 4/6. Abdomen brown, 7.5YR 4/2; distinct dorsal mottled striping pattern; purple tint along dorsal carapace fringe (Fig. 342). *Cephalothorax*. Carapace 3.88 long, 3.16 wide, lightly hirsute with thin white setae intermingled with thin black setae, stout black bristles along fringe; surface smooth, pars cephalica elevated. Fringe, posterior margin with black bristles. Foveal groove deep, strongly recurved. Eyes on low mound. AER slightly procurved, PER straight. PME, AME subequal diameter. Sternum moderately setose, STRl 2.38, STRw 1.68; elongate. Posterior sternal sigilla small, positioned laterally, anterior sigilla pairs small, oval, marginal. Chelicerae with distinct anterior tooth row comprising 5 teeth, posterior margin with single row of small denticles. Palpal endites with patch of small cuspules on proximal, inner margin, labium with 3 cuspules, LBw 0.63, LBl 0.26. Rastellum consists of 4 stout spines not on prominent mound. *Abdomen*. Setose, heavy black setae intermingled with fine black setae. *Legs*. Leg I: 3.60, 2.64, 2.88, 2.18, 0.00; leg IV: 3.88, 2.52. Light tarsal scopulae on all legs, light scopulae on metatarsus I. Tarsus I with single, slightly staggered row of 9 trichobothria. Leg I spination pattern illustrated in Figures 343, 344; TSp 14, TSr 4 , TSrd 1. Tibia I long relative to femur I, with numerous prolateral spines, few retrolateral spines. Distal retrolateral spine patch absent. Metatarsus not anteverted, distinct mid–ventral mating apophysis, proximal excavation. Metatarsus, tarsus I elongate. *Pedipalp*. Palpal tibia intermediate in length, width slightly less than half-length; PTw 0.71, PTl 1.65, Bl 0.60. Retrolateral surface bears 2 large spines (Fig. 345), surrounded by numerous smaller spines. Pyriform shaped palpal bulb short (Bl/Cl 15.46); embolus base stout, but tip very thin, slight midpoint curvature, not serrated distally.

**Variation (4).** Cl 3.88-4.50, 4.11±0.15; Cw 2.94-3.53, 3.21±0.12; STRl 2.22-2.52, 2.39±0.06; STRw 1.50-1.86, 1.69±0.07; LBw 0.54-0.68, 0.62±0.03; LBl 0.21-0.29, 0.25±0.02; leg I: 3.50-4.00, 3.68±0.11; 2.56-2.94, 2.69±0.08; 2.87-3.38, 3.02±0.12; 2.04-2.31, 2.15±0.06; 0-0, 0±0; leg IV: 3.63-4.19, 3.88±0.12; 2.38-2.63, 2.48±0.06; PTl 1.53-1.76, 1.63±0.05; PTw 0.67-0.77, 0.72±0.02; Bl 0.60-0.68, 0.63±0.02; TSp 12-16, 14.50±0.96; TSr 4-4, 4.00±0; TSrd 1-1, 1.00±0.

#### Description of female paratype.

*Specimen preparation and condition*. Female collected live from burrow, prepared in same manner as male holotype. Genital plate removed, cleared in trypsin, stored in microvial with specimen. Color slightly faded. Carapace, legs, chelicerae, strong brown, 7.5YR 5/6. Abdomen brown dorsally 7.5YR 4/2; distinct dorsal mottled striping. *Cephalothorax*. Carapace 4.32 long, 3.26 wide, glabrous with few fine black setae; generally smooth surface, pars cephalica moderately elevated. Fringe lacks setae. Foveal groove deep, recurved. Eye group slightly elevated on low mound. AER slightly procurved, PER slightly recurved. PME-AME subequal diameter. Sternum elongate, moderately setose, STRl 2.85, STRw 1.95. Three pairs of sternal sigilla, anterior pairs small, oval, marginal, posterior pair small, laterally positioned. Chelicerae anterior tooth row comprising 5 teeth with posterior margin denticle patch. Palpal endites with 16 cuspules concentrated at inner promargin posterior heel; labium with 3 cuspules, LBw 0.85, LBl 0.37. Rastellum consist of 4 stout spines not positioned on mound; fringe of short spines along distal promargin extending upward from rastellum. *Abdomen*. Moderately setose. PLS all 3 segments with spigots. Terminal segment 1/2 length of medial segment, 2 enlarged spigots visible at tip. PMS single segment, with spigots, short with rounded terminus. *Legs*. Anterior two pairs noticeably more slender than posterior pairs. Leg I 9.22 long. Tarsus I with single staggered row of 11 trichobothria. Legs I, II, with moderately heavy scopulae on tarsi, metatarsi; light scopulae on tarsi III, IV. PTLs 8, TBs 2. Rudimentary preening comb on retrolateral distal surface (at tarsus-metatarsus joint) of metatarsus IV, absent on III. *Spermathecae*. Heavily sclerotized, 2 simple spermathecal bulbs on short neck, arranged on small spermathecal base (Fig. 346).

**Variation (4).** Cl 4.00-5.31, 4.56±0.28; Cw 3.26-4.40, 3.68±0.25; STRl 2.64-3.41, 2.95±0.16; STRw 1.80-2.35, 2.03±0.12; LBw 0.82-1.02, 0.89±0.04; LBl 0.37-0.43, 0.4±0.02; Leg I: 8.91-11.31, 9.70±0.55; ANTd 5-6, 5.25±0.25; PTLs 7-10, 8.75±0.75; TBs 2-2, 2.00±0.

#### Material examined.

**United States: California: Kern Co.:** 4.8km N of Kernville, Headquarters Campground, 35.7968, -118.4495^4^, 867m, J Doyen 30.iv.1972 [AP084, 1♀, CAS]; Bakersfield, S Bank Kern River, 35.3947, -119.0313^5^, 137m, W Icenogle 23.vi.1970 [AP553, 556, 2♂, AUMNH], [AP564, 1♀, 3juv, CAS], 16.x.1970 [AP559, 560, 563, 5♂, 7♀, 20juv, AUMNH], 6.x.1971 [AP425, 426, 1♀, 1♂, 14juv, AUMNH], [AP558, 1♀, 4juv, AMNH]; S bank Kern River 0.4km from Manor St Bridge, 35.4067, -119.0118^1^, 127m, J Bond 31.iii.1996 [AP732, 1♀, AUMNH].

#### Distribution and natural history.

The distribution of *Aptostichus dorothealangeae* (Map 34) is similar to that of *Aptostichus dantrippi* (Map 12). As currently defined the species is likely distributed primarily throughout Kern County bordering the ranges that bound the central valley to the east and include the Tehachapi, Greenhorn, and Piute Mountains. *Aptostichus dorothealangeae* is mostly restricted to the South Valley Alluvium and Basinsand Sierran Steppe-Mixed Forest-Coniferous Forest-Alpine Meadow ecoregions of Kern County (Map 35). The only known male specimens were collected during late spring, early summer (May, June).

#### Species concept applied.

Morphological.

#### Conservation status.

The status of *Aptostichus dorothealangeae* is likely to be imperiled or vulnerable. The type locality, along the banks of the Kern River, in Bakersfield, California is disturbed and has been highly impacted by proximal development of the last quarter century; the species is known from relatively few specimens and is presumed to not be particularly abundant.

### Aptostichus chavezi sp. n.

‘The UFW Trapdoor Spider’

urn:lsid:zoobank.org:act:4587FAC9-CA3D-4596-88D2-85D280513CE3

http://species-id.net/wiki/Aptostichus_chavezi

**Figures 347–351.**
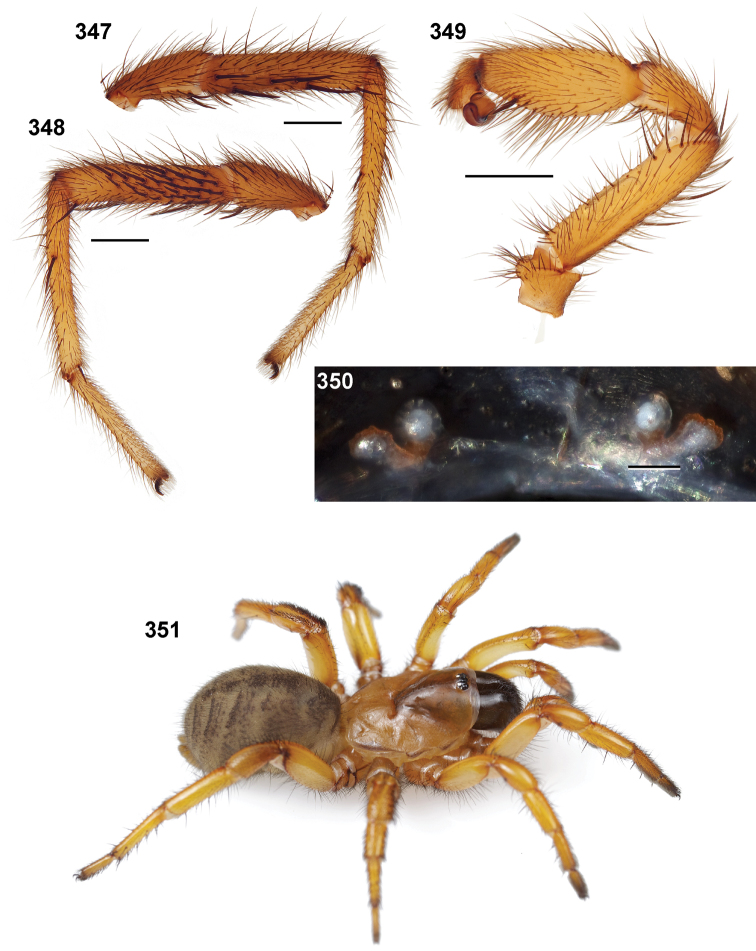
*Aptostichus chavezi* sp. n. from Tulare County. **347–349** male holotype (AP562); scale bars = 1.0mm **347** retrolateral aspect, right leg I [806401] **348** prolateral aspect, right leg I [806405] **349** retrolateral aspect, pedipalp [806407] **350** cleared spermathecae (AP1248) [806408]; scale bar = 0.1mm **351** female paratype (MY3774) from Tulare County, photographed in life.

Figures 347–351 Map 34, 35

#### Types.

Male holotype (AP562) from California, Tulare Co., Ash Mountain, Kaweah Power Station #3, 64km NE Visalia, 36.488, -118.837^5^, elev. 460m, coll. D. Burdick 3.iii.1983; female paratype (MY3774) from California, Tulare Co., HWY 245, ~22.4km N of Woodlake, near confluence of Cottonwood Creek & Rattlesnake Creek, 36.5856, -119.1220^1^, elev. 380m, coll. M. Hedin, D. Leavitt, J. Satler, J. Starrett 26.iii.2009; deposited in AUMNH.

#### Etymology.

The specific epithet is a patronym in honor of labor and civil rights leader César Chávez (1927–1993).

#### Diagnosis.

Male of *Aptostichus chavezi* can be distinguished from all other *Sierra* group species on the basis of a unique tibia I spination pattern that is most similar to that of its hypothesized and geographically proximate sister species *Aptostichus dorothealangeae*. *Aptostichus chavezi* males can be differentiated from *Aptostichus dorothealangeae* males by having a far greater number of spines on the prolateral surface of tibia I (Fig. 348; 26 vs. 12–16). Females of *Aptostichus chavezi* and *Aptostichus dorothealangeae* are very similar in appearance, however, *Aptostichus chavezi* specimens are, on average, larger (Cl 5.28 vs. 4.56) and tend to have more patella III prolateral spines (11-20 vs. 7-10). The known distributions of these two species do not overlap.

#### Description of male holotype.

*Specimen preparation and condition*. Specimen presumed collected from pitfall trap, preserved in 70%EtOH. Coloration slightly faded. Pedipalp, leg I right side removed, stored in vial with specimen; leg I left side missing. *General coloration*. Carapace, chelicerae, legs yellowish red 5YR 4/6. Abdomen brown, 7.5YR 4/3; distinct dorsal mottled striping pattern. *Cephalothorax*. Carapace 4.40 long, 3.47 wide, lightly hirsute with thin white setae intermingled with thin black setae, stout black bristles along fringe; surface smooth, pars cephalica elevated. Fringe, posterior margin with black bristles. Foveal groove deep, strongly recurved. Eyes on low mound. AER, slightly procurved, PER recurved. PME, AME subequal diameter. Sternum moderately setose, STRl 2.45, STRw 1.84; elongate. Posterior sternal sigilla small, positioned laterally, anterior sigilla pairs small, oval, marginal. Chelicerae with distinct anterior tooth row comprising 5 teeth, posterior margin with single row of small denticles. Palpal endites with patch of small cuspules on proximal, inner margin, labium with 2 cuspules, LBw 0.58, LBl 0.27. Rastellum consists of 4 stout spines not on prominent mound. *Abdomen*. Setose, heavy black setae intermingled with fine black setae. *Legs*. Leg I: 4.05, 3.04, 3.44, 2.40, 0.00; leg IV: 4.20, 2.64. Light tarsal scopulae on all legs, light scopulae on distal aspect metatarsus I. Tarsus I with single, slightly staggered row of 10 trichobothria. Leg I spination pattern illustrated in Figures 347, 348; TSp 14, TSr 4 , TSrd 1. Tibia I long relative to femur I, with numerous prolateral spines, few retrolateral spines. Distal retrolateral spine patch absent, single distal spine. Metatarsus not anteverted, lacks distinct mid–ventral mating apophysis, proximal excavation. Metatarsus, tarsus I elongate. *Pedipalp*. Palpal tibia intermediate in length, width slightly less than half-length; PTw 0.83, PTl 1.88, Bl 0.71. Retrolateral surface bears 1 large spine (Fig. 349), surrounded by few smaller spines. Pyriform shaped palpal bulb short (Bl/Cl 16.14); embolus base stout, but tip very thin, slight midpoint curvature, not serrated distally.

**Variation.** Known only from the type specimen.

#### Description of female paratype.

*Specimen preparation and condition*. Female collected live from burrow, prepared in same manner as male holotype. Genital plate removed, cleared in trypsin, stored in microvial with specimen; legs II-IV right-hand side removed for tissue storage. Color slightly faded. Carapace, legs, chelicerae, yellowish red 5YR 4/6; purplish tinting at carapace fringe (Fig. 351). Abdomen brown, 7.5YR 4/3; distinct dorsal mottled striping pattern. *Cephalothorax*. Carapace 6.06 long, 5.20 wide, glabrous with few fine black setae; generally smooth surface, pars cephalica moderately elevated. Fringe lacks setae. Foveal groove deep, slightly recurved. Eye group slightly elevated on low mound. AER slightly procurved, PER slightly recurved. PME-AME subequal diameter. Sternum elongate, moderately setose, STRl 3.40, STRw 2.73. Three pairs of sternal sigilla anterior pairs small, oval, marginal, posterior pair small, laterally positioned. Chelicerae anterior tooth row comprising 6 teeth with posterior margin denticle patch. Palpal endites with 22 cuspules concentrated at the inner promargin posterior heel; labium with 3 cuspules, LBw 1.02, LBl 0.51. Rastellum comprises of 6 stout spines not positioned on mound; fringe of short spines along distal promargin extending upward from rastellum. *Abdomen*. Moderately setose. PLS all 3 segments with spigots. Terminal segment 1/2 length of medial segment, 2 enlarged spigots visible at tip. PMS single segment, with spigots, short with rounded terminus. *Walking legs*. Anterior two pairs noticeably more slender than posterior pairs. Leg I 13.17 long. Tarsus I with single staggered row of 12 trichobothria. Legs I, II, with moderately heavy scopulae on tarsi, metatarsi; light scopulae on tarsi III, IV. PTLs 17, TBs 2. Preening combs on retrolateral distal surface (at tarsus-metatarsus joint) of metatarsus IV, III, highly reduced to absent. *Spermathecae*. Heavily sclerotized, 2 simple spermathecal bulbs on short neck, arranged on small spermathecal base (Fig. 350).

**Variation (5).** Cl 4.75-6.06, 5.28±0.25; Cw 3.88-5.20, 4.25±0.25; STRl 2.78-3.47, 3.12±0.13; STRw 2.00-2.73, 2.32±0.12; LBw 0.77-1.02, 0.92±0.05; LBl 0.36-0.51, 0.42±0.03; Leg I: 10.42-13.17, 11.39±0.56; ANTd 5-6, 5.8±0.2; PTLs 11-20, 15.20±1.56; TBs 2-4, 3.4±0.4.

#### Material examined:

**United States: California: Fresno Co.:** Elwood Rd, above Tretten Canyon, N Hwy 180, 36.7692, -119.2558^1^, 470m, M Hedin, D Leavitt, J Satler, J Starrett 26.iii.2009 [MY3773, 1juv, AUMNH]; Hopewell Rd, N of Clingans jct, Hwy 180, 36.7737, -119.1719^1^, 710m, M Hedin, D Leavitt, J Satler, J Starrett 26.iii.2009 [MY3772, 1juv, AUMNH]; **Tuolumne Co.:** 5km W Columbia, Crystal Palace Cave, 38.035, -120.4494^5^, 360m, W Elliott, A Grubbs, S Winterath 21.ii.1977 [AP557, 1♀, AMNH]; **Tulare Co.:** Hwy 198 S end Lake Kaweah, 36.3958, -118.9478^1^, 253m, J Bond 8.v.1997 [AP782, 783 2♀, CAS; AP784, 786, 787, 2♀, 1juv, AUMNH]; Ash Mountain, Kaweah Power Station #3 (64km NE Visalia), 36.488, -118.837^5^, 460m, D Burdick 10.vii.1983 [AP561, 562, 1juv, CAS], 3.iii.1983 [AP562, 1♂, CAS]; 9.6km [road] S Badger on Road J21, 36.5783, -119.0111^5^, 524m, W Icenogle 17.x.1973 [AP554, 1♀, AMNH]; Hwy 245, ~22.4km N of Woodlake, near confluence Cottonwood Crk & Rattlesnake Crk, 36.5856, -119.122^1^, 380m, M Hedin, D Leavitt, J Satler, J Starrett 26.iii.2009 [AP1248, MY3774, 2♀, AUMNH].

#### Distribution and natural history.

*Aptostichus chavezi* is distributed predominantly throughout Tulare and Fresno Counties (Fig. 34) in the Sierra Nevada foothills. The locality further to the north in Fresno County, not predicted in the DM (Fig. 35) may be a fifth, closely related *Sierra* group species. Because only a single specimen is known I have chosen to be conservative and include this specimen as part of the *Aptostichus chavezi* hypothesis until males become available. The habitat type throughout this species range is characterized as Sierran steppe, mixed and coniferous forest. Based on the single male specimen, it appears that males are late winter dispersers (March).

#### Conservation status.

Based on its widespread distribution and general abundance in collections, this species would likely be considered secure.

#### Species concept applied.

Morphological.

## Supplementary Material

XML Treatment for Euctenizidae

XML Treatment for Apomastinae

XML Treatment for Aptostichus

XML Treatment for Aptostichus atomarius

XML Treatment for Aptostichus stephencolberti

XML Treatment for Aptostichus angelinajolieae

XML Treatment for Aptostichus miwok

XML Treatment for Aptostichus stanfordianus

XML Treatment for Aptostichus dantrippi

XML Treatment for Aptostichus pennjillettei

XML Treatment for Aptostichus asmodaeus

XML Treatment for Aptostichus nateevansi

XML Treatment for Aptostichus chiricahua

XML Treatment for Aptostichus icenoglei

XML Treatment for Aptostichus cabrillo

XML Treatment for Aptostichus isabella

XML Treatment for Aptostichus muiri

XML Treatment for Aptostichus barackobamai

XML Treatment for Aptostichus hesperus

XML Treatment for Aptostichus hedinorum

XML Treatment for Aptostichus cahuilla

XML Treatment for Aptostichus killerdana

XML Treatment for Aptostichus serrano

XML Treatment for Aptostichus aguacaliente

XML Treatment for Aptostichus chemehuevi

XML Treatment for Aptostichus sarlacc

XML Treatment for Aptostichus derhamgiulianii

XML Treatment for Aptostichus mikeradtkei

XML Treatment for Aptostichus edwardabbeyi

XML Treatment for Aptostichus anzaborrego

XML Treatment for Aptostichus sinnombre

XML Treatment for Aptostichus simus

XML Treatment for Aptostichus satleri

XML Treatment for Aptostichus elisabethae

XML Treatment for Aptostichus fornax

XML Treatment for Aptostichus lucerne

XML Treatment for Aptostichus bonoi

XML Treatment for Aptostichus fisheri

XML Treatment for Aptostichus cajalco

XML Treatment for Aptostichus sierra

XML Treatment for Aptostichus huntington

XML Treatment for Aptostichus dorothealangeae

XML Treatment for Aptostichus chavezi

## Appendix 1

Locality data for *Aptostichus* specimens. (doi: 10.3897/zookeys.252.3588.app1) File format: Microsoft Excel comma delimited (.csv).

**Explanation note:** Locality data for *Aptostichus* specimens examined over the course of this study and listed in the material examined section that accompanies each species.

## Appendix 2

Type locality data. (doi: 10.3897/zookeys.252.3588.app2) File format: Microsoft Excel comma delimited (.csv).

**Explanation note:** Latitude and longitude data for the type localities for each *Aptostichus* species.

## Appendix 3

KML file containing *Aptostichus* locality data. (doi: 10.3897/zookeys.252.3588.app3) File format: Keyhole Markup Language (.kmz).

**Explanation note:** Locality data for all *Aptostichus* specimens.

## Appendix 4

Morphological data matrix. (doi: 10.3897/zookeys.252.3588.app4) File format: Nexus (.nex).

**Explanation note:** The morphological data matrix scored for all *Aptostichus* species and outgroup exemplar taxa.
